# THE CONCISE GUIDE TO PHARMACOLOGY 2019/20: Ion channels

**DOI:** 10.1111/bph.14749

**Published:** 2019-11-11

**Authors:** Stephen PH Alexander, Alistair Mathie, John A Peters, Emma L Veale, Jörg Striessnig, Eamonn Kelly, Jane F Armstrong, Elena Faccenda, Simon D Harding, Adam J Pawson, Joanna L Sharman, Christopher Southan, Jamie A Davies, CGTP Collaborators, Richard W Aldrich, Elvir Becirovic, Martin Biel, William A Catterall, Alex C Conner, Paul Davies, Markus Delling, Francesco Di Virgilio, Simonetta Falzoni, Chandy George, Steve AN Goldstein, Stephan Grissmer, Kotdaji Ha, Verena Hammelmann, Israel Hanukoglu, Mike Jarvis, Anders A Jensen, Leonard K Kaczmarek, Stephan Kellenberger, Charles Kennedy, Brian King, Joseph W Lynch, Edward Perez-Reyes, Leigh D Plant, Lachlan D Rash, Dejian Ren, Lucia G Sivilotti, Trevor G Smart, Terrance P Snutch, Jinbin Tian, Charlotte Van den Eynde, Joris Vriens, Aguan D Wei, Brenda T Winn, Heike Wulff, Haoxing Xu, Lixia Yue, Xiaoli Zhang, Michael Zhu

**Affiliations:** ^1^ School of Life Sciences University of Nottingham Medical School Nottingham NG7 2UH UK; ^2^ Medway School of Pharmacy The Universities of Greenwich and Kent at Medway Anson Building, Central Avenue, Chatham Maritime Chatham Kent ME4 4TB UK; ^3^ Neuroscience Division, Medical Education Institute, Ninewells Hospital and Medical School University of Dundee Dundee DD1 9SY UK; ^4^ Pharmacology and Toxicology, Institute of Pharmacy University of Innsbruck A-6020 Innsbruck Austria; ^5^ School of Physiology, Pharmacology and Neuroscience University of Bristol Bristol BS8 1TD UK; ^6^ Centre for Discovery Brain Science University of Edinburgh Edinburgh EH8 9XD UK; ^7^ Austin USA; ^8^ Munich Germany; ^9^ Seattle USA; ^10^ Birmingham UK; ^11^ Boston USA; ^12^ San Francisco USA; ^13^ Ferrara Italy; ^14^ Singapore Singapore; ^15^ Irvine USA; ^16^ Ulm Germany; ^17^ Ariel Israel; ^18^ Chicago USA; ^19^ Copenhagen Denmark; ^20^ New Haven USA; ^21^ Lausanne Switzerland; ^22^ Glasgow UK; ^23^ London UK; ^24^ Brisbane Australia; ^25^ Charlottesville USA; ^26^ Philadelphia USA; ^27^ Vancouver Canada; ^28^ Houston USA; ^29^ Leuven Belgium; ^30^ Davis USA; ^31^ Ann Arbor USA; ^32^ Farmington USA

## Abstract

The Concise Guide to PHARMACOLOGY 2019/20 is the fourth in this series of biennial publications. The Concise Guide provides concise overviews of the key properties of nearly 1800 human drug targets with an emphasis on selective pharmacology (where available), plus links to the open access knowledgebase source of drug targets and their ligands (http://www.guidetopharmacology.org/), which provides more detailed views of target and ligand properties. Although the Concise Guide represents approximately 400 pages, the material presented is substantially reduced compared to information and links presented on the website. It provides a permanent, citable, point‐in‐time record that will survive database updates. The full contents of this section can be found at http://onlinelibrary.wiley.com/doi/10.1111/bph.14749. Ion channels are one of the six major pharmacological targets into which the Guide is divided, with the others being: G protein‐coupled receptors, nuclear hormone receptors, catalytic receptors, enzymes and transporters. These are presented with nomenclature guidance and summary information on the best available pharmacological tools, alongside key references and suggestions for further reading. The landscape format of the Concise Guide is designed to facilitate comparison of related targets from material contemporary to mid‐2019, and supersedes data presented in the 2017/18, 2015/16 and 2013/14 Concise Guides and previous Guides to Receptors and Channels. It is produced in close conjunction with the International Union of Basic and Clinical Pharmacology Committee on Receptor Nomenclature and Drug Classification (NC‐IUPHAR), therefore, providing official IUPHAR classification and nomenclature for human drug targets, where appropriate.

1

### Conflict of interest

The authors state that there are no conflicts of interest to disclose.

### Overview

Ion channels are pore‐forming proteins that allow the flow of ions across membranes, either plasma membranes, or the membranes of intracellular organelles [http://www.ncbi.nlm.nih.gov/pubmed/2452140?dopt=AbstractPlus]. Many ion channels (such as most Na, K, Ca and some Cl channels) are gated by voltage but others (such as certain K and Cl channels, TRP channels, ryanodine receptors and IP_3_ receptors) are relatively voltage‐insensitive and are gated by second messengers and other intracellular and/or extracellular mediators. As such, there is some blurring of the boundaries between “ion channels” and “ligand‐gated channels” which are compiled separately in the Guide. Resolution of ion channel structures, beginning with K channels [http://www.ncbi.nlm.nih.gov/pubmed/9525859?dopt=AbstractPlus] then Cl channels [http://www.ncbi.nlm.nih.gov/pubmed/11796999?dopt=AbstractPlus] and most recently Na channels [http://www.ncbi.nlm.nih.gov/pubmed/21743477?dopt=AbstractPlus] has greatly improved understanding of the structural basis behind ion channel function. Many ion channels (*e.g.*, K, Na, Ca, HCN and TRP channels) share several structural similarities. These channels are thought to have evolved from a common ancestor and have been classfied together as the “voltage‐gated‐like (VGL) ion channel chanome” (see [http://www.ncbi.nlm.nih.gov/pubmed/16382097?dopt=AbstractPlus]). Other ion channels, however, such as Cl channels, aquaporins and connexins, have completely different structural properties to the VGL channels, having evolved quite separately.

Currently, ion channels (including ligand‐gated ion channels) represent the second largest target for existing drugs after G protein‐coupled receptors [http://www.ncbi.nlm.nih.gov/pubmed/17139284?dopt=AbstractPlus]. However, the advent of novel, faster screening techniques for compounds acting on ion channels [http://www.ncbi.nlm.nih.gov/pubmed/18356919?dopt=AbstractPlus] suggests that these proteins represent promising targets for the development of additional, novel therapeutic agents for the near future.

### Family structure

S143 Ligand‐gated ion channels

S144 5‐HT_3_ receptors

S146 Acid‐sensing (proton‐gated) ion channels (ASICs)

S148 Epithelial sodium channel (ENaC)

S149 GABA_A_ receptors

S155 Glycine receptors

S158 Ionotropic glutamate receptors

S164 IP_3_ receptor

S165 Nicotinic acetylcholine receptors

S168 P2X receptors

S170 ZAC

S171 Voltage‐gated ion channels

S171 CatSper and Two‐Pore channels

S173 Cyclic nucleotide‐regulated channels

S175 Potassium channels

S175 Calcium‐ and sodium‐activated potassium channels

S178 Inwardly rectifying potassium channels

S182 Two P domain potassium channels

S185 Voltage‐gated potassium channels

S189 Ryanodine receptors

S190 Transient Receptor Potential channels

S204 Voltage‐gated calcium channels

S207 Voltage‐gated proton channel

S208 Voltage‐gated sodium channels

S210 Other ion channels

S210 Aquaporins

S212 Chloride channels

S213 ClC family

S215 CFTR

S216 Calcium activated chloride channel

S217 Maxi chloride channel

S218 Volume regulated chloride channels

S219 Connexins and Pannexins

S221 Piezo channels

S222 Sodium leak channel, non‐selective

– http://www.guidetopharmacology.org/GRAC/FamilyDisplayForward?familyId=976

– http://www.guidetopharmacology.org/GRAC/FamilyDisplayForward?familyId=977

## http://www.guidetopharmacology.org/GRAC/FamilyDisplayForward?familyId=697

### Overview

Ligand‐gated ion channels (LGICs) are integral membrane proteins that contain a pore which allows the regulated flow of selected ions across the plasma membrane. Ion flux is passive and driven by the electrochemical gradient for the permeant ions. These channels are open, or gated, by the binding of a neurotransmitter to an orthosteric site(s) that triggers a conformational change that results in the conducting state. Modulation of gating can occur by the binding of endogenous, or exogenous, modulators to allosteric sites. LGICs mediate fast synaptic transmission, on a millisecond time scale, in the nervous system and at the somatic neuromuscular junction. Such transmission involves the release of a neurotransmitter from a pre‐synaptic neurone and the subsequent activation of post‐synaptically located receptors that mediate a rapid, phasic, electrical signal (the excitatory, or inhibitory, post‐synaptic potential). However, in addition to their traditional role in phasic neurotransmission, it is now established that some LGICs mediate a tonic form of neuronal regulation that results from the activation of extra‐synaptic receptors by ambient levels of neurotransmitter. The expression of some LGICs by nonexcitable cells is suggestive of additional functions.

By convention, the LGICs comprise the excitatory, cationselective, nicotinic acetylcholine [http://www.ncbi.nlm.nih.gov/pubmed/20055696?dopt=AbstractPlus, http://www.ncbi.nlm.nih.gov/pubmed/18723036?dopt=AbstractPlus], 5‐HT3 [http://www.ncbi.nlm.nih.gov/pubmed/18761359?dopt=AbstractPlus, http://www.ncbi.nlm.nih.gov/pubmed/20621123?dopt=AbstractPlus], ionotropic glutamate [http://www.ncbi.nlm.nih.gov/pubmed/18765242?dopt=AbstractPlus, http://www.ncbi.nlm.nih.gov/pubmed/20716669?dopt=AbstractPlus] and P2X receptors [http://www.ncbi.nlm.nih.gov/pubmed/18657557?dopt=AbstractPlus, http://www.ncbi.nlm.nih.gov/pubmed/18851707?dopt=AbstractPlus] and the inhibitory, anion‐selective, GABAA [http://www.ncbi.nlm.nih.gov/pubmed/19828786?dopt=AbstractPlus, http://www.ncbi.nlm.nih.gov/pubmed/18790874?dopt=AbstractPlus] and glycine receptors [http://www.ncbi.nlm.nih.gov/pubmed/18721822?dopt=AbstractPlus, http://www.ncbi.nlm.nih.gov/pubmed/21557733?dopt=AbstractPlus]. The nicotinic acetylcholine, 5‐HT_3_, GABA_A_ and glycine receptors (and an additional zinc‐activated channel) are pentameric structures and are frequently referred to as the Cysloop receptors due to the presence of a defining loop of residues formed by a disulphide bond in the extracellular domain of their constituent subunits [http://www.ncbi.nlm.nih.gov/pubmed/20096941?dopt=AbstractPlus, http://www.ncbi.nlm.nih.gov/pubmed/20849671?dopt=AbstractPlus]. However, the prokaryotic ancestors of these receptors contain no such loop and the term pentameric ligand‐gated ion channel (pLGIC) is gaining acceptance in the literature [http://www.ncbi.nlm.nih.gov/pubmed/19646860?dopt=AbstractPlus]. The ionotropic glutamate and P2X receptors are tetrameric and trimeric structures, respectively. Multiple genes encode the subunits of LGICs and the majority of these receptors are heteromultimers. Such combinational diversity results, within eachclass of LGIC,in a widerangeof receptors withdiffering pharmacological and biophysical properties and varying patternsof expression within the nervous system and other tissues. The LGICs thus present attractive targets for new therapeutic agents with improved discrimination between receptor isoforms and a reduced propensity for off‐target effects. The development of novel, faster screening techniques for compounds acting on LGICs [http://www.ncbi.nlm.nih.gov/pubmed/18356919?dopt=AbstractPlus] will greatly aid in the development of such agents.

## http://www.guidetopharmacology.org/GRAC/FamilyDisplayForward?familyId=68

### Overview

The 5‐HT_3_ receptor (**nomenclature as agreed by the NC‐IUPHAR Subcommittee on 5‐Hydroxytryptamine (serotonin) receptors** [http://www.ncbi.nlm.nih.gov/pubmed/7938165?dopt=AbstractPlus]) is a ligand‐gated ion channel of the Cys‐loop family that includes the zinc‐activated channels, nicotinic acetylcholine, GABA_A_ and strychnine‐sensitive glycine receptors. The receptor exists as a pentamer of 4TM subunits that form an intrinsic cation selective channel [http://www.ncbi.nlm.nih.gov/pubmed/18761359?dopt=AbstractPlus]. Five human 5‐HT_3_ receptor subunits have been cloned and homo‐oligomeric assemblies of 5‐HT_3_A and hetero‐oligomeric assemblies of 5‐HT_3_A and 5‐HT_3_B subunits have been characterised in detail. The 5‐HT_3_C (https://www.genenames.org/data/gene‐symbol‐report/#!/hgnc_id/HGNC:24003, http://www.uniprot.org/uniprot/Q8WXA8), 5‐HT_3_D (https://www.genenames.org/data/gene‐symbol‐report/#!/hgnc_id/HGNC:24004, http://www.uniprot.org/uniprot/Q70Z44) and 5‐HT_3_E (https://www.genenames.org/data/gene‐symbol‐report/#!/hgnc_id/HGNC:24005, http://www.uniprot.org/uniprot/A5X5Y0) subunits [http://www.ncbi.nlm.nih.gov/pubmed/14597179?dopt=AbstractPlus, http://www.ncbi.nlm.nih.gov/pubmed/12801637?dopt=AbstractPlus], like the 5‐HT_3_B subunit, do not form functional homomers, but are reported to assemble with the 5‐HT_3_A subunit to influence its functional expression rather than pharmacological profile [http://www.ncbi.nlm.nih.gov/pubmed/19012743?dopt=AbstractPlus, http://www.ncbi.nlm.nih.gov/pubmed/17392525?dopt=AbstractPlus, http://www.ncbi.nlm.nih.gov/pubmed/20522555?dopt=AbstractPlus]. 5‐HT_3_A, ‐C, ‐D, and ‐E subunits also interact with the chaperone RIC‐3 which predominantly enhances the surface expression of homomeric 5‐HT_3_A receptor [http://www.ncbi.nlm.nih.gov/pubmed/20522555?dopt=AbstractPlus]. The co‐expression of 5‐HT_3_A and 5‐HT_3_C‐E subunits has been demonstrated in human colon [http://www.ncbi.nlm.nih.gov/pubmed/21192076?dopt=AbstractPlus]. A recombinant hetero‐oligomeric 5‐HT_3_AB receptor has been reported to contain two copies of the 5‐HT_3_A subunit and three copies of the 5‐HT_3_B subunit in theorderB‐B‐A‐B‐A[http://www.ncbi.nlm.nih.gov/pubmed/16116092?dopt=AbstractPlus], butthis is inconsistent with recent reports which show at least one A‐A interface [http://www.ncbi.nlm.nih.gov/pubmed/20409468?dopt=AbstractPlus, http://www.ncbi.nlm.nih.gov/pubmed/21708905?dopt=AbstractPlus]. The 5‐HT_3_B subunit imparts distinctive biophysical properties upon hetero‐oligomeric 5‐HT_3_AB versus homo‐oligomeric 5‐HT_3_A recombinant receptors [http://www.ncbi.nlm.nih.gov/pubmed/9950429?dopt=AbstractPlus, http://www.ncbi.nlm.nih.gov/pubmed/10521471?dopt=AbstractPlus, http://www.ncbi.nlm.nih.gov/pubmed/10854267?dopt=AbstractPlus, http://www.ncbi.nlm.nih.gov/pubmed/18597859?dopt=AbstractPlus, http://www.ncbi.nlm.nih.gov/pubmed/12867984?dopt=AbstractPlus, http://www.ncbi.nlm.nih.gov/pubmed/16194573?dopt=AbstractPlus, http://www.ncbi.nlm.nih.gov/pubmed/12623220?dopt=AbstractPlus], influences the potency of channel blockers, but generally has only a modest effect upon the apparent affinity of agonists, or the affinity of antagonists ([http://www.ncbi.nlm.nih.gov/pubmed/11489465?dopt=AbstractPlus], but see [http://www.ncbi.nlm.nih.gov/pubmed/14625088?dopt=AbstractPlus, http://www.ncbi.nlm.nih.gov/pubmed/19131665?dopt=AbstractPlus, http://www.ncbi.nlm.nih.gov/pubmed/10521471?dopt=AbstractPlus]) which may be explained by the orthosteric binding site residing at an interface formed between 5‐HT_3_A subunits [http://www.ncbi.nlm.nih.gov/pubmed/20409468?dopt=AbstractPlus, http://www.ncbi.nlm.nih.gov/pubmed/21708905?dopt=AbstractPlus]. However, 5‐HT_3_A and 5‐HT_3_AB receptors differ in their allosteric regulation by some general anaesthetic agents, small alcohols and indoles [http://www.ncbi.nlm.nih.gov/pubmed/18187416?dopt=AbstractPlus, http://www.ncbi.nlm.nih.gov/pubmed/17360702?dopt=AbstractPlus, http://www.ncbi.nlm.nih.gov/pubmed/16081679?dopt=AbstractPlus]. The potential diversity of 5‐HT_3_ receptors is increased by alternative splicing of the genes HTR3A and E [http://www.ncbi.nlm.nih.gov/pubmed/11111833?dopt=AbstractPlus, http://www.ncbi.nlm.nih.gov/pubmed/7683998?dopt=AbstractPlus, http://www.ncbi.nlm.nih.gov/pubmed/21345729?dopt=AbstractPlus, http://www.ncbi.nlm.nih.gov/pubmed/18466097?dopt=AbstractPlus, http://www.ncbi.nlm.nih.gov/pubmed/17392525?dopt=AbstractPlus]. In addition, the use of tissue‐specific promoters driving expression from differenttranscriptional start sites has beenreported for the*HTR3A, HTR3B,HTR3D* and *HTR3E* genes, which could result in 5‐HT_3_ subunits harbouring different N‐termini [http://www.ncbi.nlm.nih.gov/pubmed/18597859?dopt=AbstractPlus, http://www.ncbi.nlm.nih.gov/pubmed/21345729?dopt=AbstractPlus, http://www.ncbi.nlm.nih.gov/pubmed/17010535?dopt=AbstractPlus]. To date, inclusion of the 5‐HT_3_A subunit appears imperative for 5‐HT_3_ receptor function.

**Table:** 

Nomenclature	http://www.guidetopharmacology.org/GRAC/ObjectDisplayForward?objectId=378	http://www.guidetopharmacology.org/GRAC/ObjectDisplayForward?objectId=379
Subunits	http://www.guidetopharmacology.org/GRAC/ObjectDisplayForward?objectId=373, http://www.guidetopharmacology.org/GRAC/ObjectDisplayForward?objectId=374	http://www.guidetopharmacology.org/GRAC/ObjectDisplayForward?objectId=373
Selective agonists	–	http://www.guidetopharmacology.org/GRAC/LigandDisplayForward?ligandId=2287‐http://www.guidetopharmacology.org/GRAC/LigandDisplayForward?ligandId=2287 [http://www.ncbi.nlm.nih.gov/pubmed/8848005?dopt=AbstractPlus, http://www.ncbi.nlm.nih.gov/pubmed/9950429?dopt=AbstractPlus, http://www.ncbi.nlm.nih.gov/pubmed/9463477?dopt=AbstractPlus, http://www.ncbi.nlm.nih.gov/pubmed/7565620?dopt=AbstractPlus, http://www.ncbi.nlm.nih.gov/pubmed/10204690?dopt=AbstractPlus], http://www.guidetopharmacology.org/GRAC/LigandDisplayForward?ligandId=218 [http://www.ncbi.nlm.nih.gov/pubmed/8848005?dopt=AbstractPlus, http://www.ncbi.nlm.nih.gov/pubmed/9950429?dopt=AbstractPlus, http://www.ncbi.nlm.nih.gov/pubmed/9463477?dopt=AbstractPlus, http://www.ncbi.nlm.nih.gov/pubmed/7565620?dopt=AbstractPlus], http://www.guidetopharmacology.org/GRAC/LigandDisplayForward?ligandId=4316 [http://www.ncbi.nlm.nih.gov/pubmed/8891244?dopt=AbstractPlus] – Rat, http://www.guidetopharmacology.org/GRAC/LigandDisplayForward?ligandId=2284 [http://www.ncbi.nlm.nih.gov/pubmed/8848005?dopt=AbstractPlus]
Antagonists	–	http://www.guidetopharmacology.org/GRAC/LigandDisplayForward?ligandId=7351 (p*K*_i_ 8.4) [http://www.ncbi.nlm.nih.gov/pubmed/21486038?dopt=AbstractPlus], http://www.guidetopharmacology.org/GRAC/LigandDisplayForward?ligandId=241 (p*K*_i_ 6–6.4) [http://www.ncbi.nlm.nih.gov/pubmed/11489465?dopt=AbstractPlus, http://www.ncbi.nlm.nih.gov/pubmed/8818349?dopt=AbstractPlus]
Selective antagonists	–	http://www.guidetopharmacology.org/GRAC/LigandDisplayForward?ligandId=7486 (p*K*_i_ 10.5) [http://www.ncbi.nlm.nih.gov/pubmed/20724042?dopt=AbstractPlus], http://www.guidetopharmacology.org/GRAC/LigandDisplayForward?ligandId=2296 (p*K*_i_ 9.5) [http://www.ncbi.nlm.nih.gov/pubmed/17652911?dopt=AbstractPlus], (http://www.guidetopharmacology.org/GRAC/LigandDisplayForward?ligandId=2289 (p*K*_i_ 9) [http://www.ncbi.nlm.nih.gov/pubmed/11489465?dopt=AbstractPlus], http://www.guidetopharmacology.org/GRAC/LigandDisplayForward?ligandId=2300 (p*K*_i_ ∼8.6–8.8) [http://www.ncbi.nlm.nih.gov/pubmed/8818349?dopt=AbstractPlus, http://www.ncbi.nlm.nih.gov/pubmed/7565620?dopt=AbstractPlus], http://www.guidetopharmacology.org/GRAC/LigandDisplayForward?ligandId=260 (p*K*_i_ 8.5–8.8) [http://www.ncbi.nlm.nih.gov/pubmed/9463477?dopt=AbstractPlus, http://www.ncbi.nlm.nih.gov/pubmed/7565620?dopt=AbstractPlus], http://www.guidetopharmacology.org/GRAC/LigandDisplayForward?ligandId=2290 (p*K*_i_ ∼7.8–8.3) [http://www.ncbi.nlm.nih.gov/pubmed/11489465?dopt=AbstractPlus, http://www.ncbi.nlm.nih.gov/pubmed/8818349?dopt=AbstractPlus, http://www.ncbi.nlm.nih.gov/pubmed/7565620?dopt=AbstractPlus]
Channel blockers	http://www.guidetopharmacology.org/GRAC/LigandDisplayForward?ligandId=2291 (pIC_50_ 4.2) [http://www.ncbi.nlm.nih.gov/pubmed/21059362?dopt=AbstractPlus], http://www.guidetopharmacology.org/GRAC/LigandDisplayForward?ligandId=2366 (pIC_50_ 2.5) [http://www.ncbi.nlm.nih.gov/pubmed/21059362?dopt=AbstractPlus], http://www.guidetopharmacology.org/GRAC/LigandDisplayForward?ligandId=1862 (pIC_50_ 2.4) [http://www.ncbi.nlm.nih.gov/pubmed/21059362?dopt=AbstractPlus]	http://www.guidetopharmacology.org/GRAC/LigandDisplayForward?ligandId=2291 (pIC_50_ 5) [http://www.ncbi.nlm.nih.gov/pubmed/21505038?dopt=AbstractPlus], http://www.guidetopharmacology.org/GRAC/LigandDisplayForward?ligandId=4323 (pIC_50_ 4.9) [http://www.ncbi.nlm.nih.gov/pubmed/10381768?dopt=AbstractPlus], http://www.guidetopharmacology.org/GRAC/LigandDisplayForward?ligandId=2298 (pIC_50_ 4.7) [http://www.ncbi.nlm.nih.gov/pubmed/21505038?dopt=AbstractPlus], http://www.guidetopharmacology.org/GRAC/LigandDisplayForward?ligandId=2366 (pIC_50_ 3.3) [http://www.ncbi.nlm.nih.gov/pubmed/21505038?dopt=AbstractPlus], http://www.guidetopharmacology.org/GRAC/LigandDisplayForward?ligandId=1862 (pIC_50_ 3.1) [http://www.ncbi.nlm.nih.gov/pubmed/21505038?dopt=AbstractPlus]
Labelled ligands	–	http://www.guidetopharmacology.org/GRAC/LigandDisplayForward?ligandId=2305 (Antagonist) (p*K*_d_ 9.8) [http://www.ncbi.nlm.nih.gov/pubmed/7565620?dopt=AbstractPlus], http://www.guidetopharmacology.org/GRAC/LigandDisplayForward?ligandId=2303 (Antagonist) (p*K*_d_ 8.6–9.3) [http://www.ncbi.nlm.nih.gov/pubmed/17652911?dopt=AbstractPlus, http://www.ncbi.nlm.nih.gov/pubmed/9463477?dopt=AbstractPlus], http://www.guidetopharmacology.org/GRAC/LigandDisplayForward?ligandId=2292 (Antagonist) (p*K*_d_ 8.9) [http://www.ncbi.nlm.nih.gov/pubmed/11489465?dopt=AbstractPlus, http://www.ncbi.nlm.nih.gov/pubmed/8818349?dopt=AbstractPlus], http://www.guidetopharmacology.org/GRAC/LigandDisplayForward?ligandId=4074 (Antagonist) (p*K*_d_ 8.7) [http://www.ncbi.nlm.nih.gov/pubmed/8994113?dopt=AbstractPlus], http://www.guidetopharmacology.org/GRAC/LigandDisplayForward?ligandId=4087 (Antagonist) (p*K*_d_ 8.5) [http://www.ncbi.nlm.nih.gov/pubmed/8419547?dopt=AbstractPlus]
Functional Characteristics	γ= 0.4‐0.8 pS [+ 5‐HT3B, γ= 16 pS]; inwardly rectifying current [+ 5‐HT3B, rectification reduced]; n_H_ 2‐3 [+ 5‐HT3B 1‐2]; relative permeability to divalent cations reduced by co‐expression of the 5‐HT3B subunit	γ= 0.4‐0.8 pS [+ 5‐HT3B, γ= 16 pS]; inwardly rectifying current [+ 5‐HT3B, rectification reduced]; n_H_ 2‐3 [+ 5‐HT3B 1‐2]; relative permeability to divalent cations reduced by co‐expression of the 5‐HT3B subunit

### Subunits

**Table:** 

Nomenclature	http://www.guidetopharmacology.org/GRAC/ObjectDisplayForward?objectId=373	http://www.guidetopharmacology.org/GRAC/ObjectDisplayForward?objectId=374	http://www.guidetopharmacology.org/GRAC/ObjectDisplayForward?objectId=375	http://www.guidetopharmacology.org/GRAC/ObjectDisplayForward?objectId=376	http://www.guidetopharmacology.org/GRAC/ObjectDisplayForward?objectId=377
HGNC, UniProt	https://www.genenames.org/data/gene‐symbol‐report/#!/hgnc_id/HGNC:5297, http://www.uniprot.org/uniprot/P46098	https://www.genenames.org/data/gene‐symbol‐report/#!/hgnc_id/HGNC:5298, http://www.uniprot.org/uniprot/O95264	https://www.genenames.org/data/gene‐symbol‐report/#!/hgnc_id/HGNC:24003, http://www.uniprot.org/uniprot/Q8WXA8	https://www.genenames.org/data/gene‐symbol‐report/#!/hgnc_id/HGNC:24004, http://www.uniprot.org/uniprot/Q70Z44	https://www.genenames.org/data/gene‐symbol‐report/#!/hgnc_id/HGNC:24005, http://www.uniprot.org/uniprot/A5X5Y0
Functional Characteristics	γ= 0.4‐0.8 pS [+ 5‐HT_3_B, γ= 16 pS]; inwardly rectifying current [+ 5‐HT_3_B, rectification reduced]; nH 2‐3 [+ 5‐HT_3_B 1‐2]; relative permeability to divalent cations reduced by co‐expression of the 5‐HT3B subunit	γ= 0.4‐0.8 pS [+ 5‐HT_3_B, γ= 16 pS]; inwardly rectifying current [+ 5‐HT_3_B, rectification reduced]; nH 2‐3 [+ 5‐HT_3_B 1‐2]; relative permeability to divalent cations reduced by co‐expression of the 5‐HT3B subunit	–	–	–

### Comments

Quantitative data in the table refer to homooligomeric assemblies of the human 5‐HT_3_A subunit, or the receptor native to human tissues. Significant changes introduced by co‐expression of the 5‐HT_3_B subunit are indicated in parenthesis. Although not a selective antagonist, http://www.guidetopharmacology.org/GRAC/LigandDisplayForward?ligandId=5458 displays multimodal and subunit‐dependent antagonism of 5‐HT_3_ receptors [http://www.ncbi.nlm.nih.gov/pubmed/19131665?dopt=AbstractPlus]. Similarly, http://www.guidetopharmacology.org/GRAC/LigandDisplayForward?ligandId=4323, http://www.guidetopharmacology.org/GRAC/LigandDisplayForward?ligandId=2298, http://www.guidetopharmacology.org/GRAC/LigandDisplayForward?ligandId=4051, http://www.guidetopharmacology.org/GRAC/LigandDisplayForward?ligandId=2366 and http://www.guidetopharmacology.org/GRAC/LigandDisplayForward?ligandId=1862 are not selective for 5‐HT_3_ receptors (*e.g.*[http://www.ncbi.nlm.nih.gov/pubmed/21059362?dopt=AbstractPlus]). The anti‐malarial drugs http://www.guidetopharmacology.org/GRAC/LigandDisplayForward?ligandId=4252and http://www.guidetopharmacology.org/GRAC/LigandDisplayForward?ligandId=2510 exerta modestly more potent block of 5‐HT_3_A versus 5‐HT_3_AB receptor‐mediated responses [http://www.ncbi.nlm.nih.gov/pubmed/18311193?dopt=AbstractPlus]. Knownbetterasa partial agonistof nicotinic acetylcholine α4β2 receptors, http://www.guidetopharmacology.org/GRAC/LigandDisplayForward?ligandId=5459 is also an agonist of the 5‐HT_3_A receptor [http://www.ncbi.nlm.nih.gov/pubmed/21775477?dopt=AbstractPlus]. Human [http://www.ncbi.nlm.nih.gov/pubmed/8848005?dopt=AbstractPlus, http://www.ncbi.nlm.nih.gov/pubmed/7565620?dopt=AbstractPlus], rat [http://www.ncbi.nlm.nih.gov/pubmed/7509203?dopt=AbstractPlus], mouse [http://www.ncbi.nlm.nih.gov/pubmed/1718042?dopt=AbstractPlus], guinea‐pig [http://www.ncbi.nlm.nih.gov/pubmed/9463477?dopt=AbstractPlus] ferret [http://www.ncbi.nlm.nih.gov/pubmed/10884508?dopt=AbstractPlus] and canine [http://www.ncbi.nlm.nih.gov/pubmed/16647053?dopt=AbstractPlus] orthologues of the 5‐HT_3_A receptor subunit have been cloned that exhibit intraspecies variations in receptor pharmacology. Notably, most ligands display significantly reduced affinities at the guinea‐pig 5‐HT_3_ receptor in comparison with other species. In addition to the agents listed in the table, native and recombinant 5‐HT_3_ receptors are subject to allosteric modulation by extracellular divalent cations, alcohols, several general anaesthetics and 5‐hydroxy‐ and halidesubstituted indoles (see reviews [http://www.ncbi.nlm.nih.gov/pubmed/8936343?dopt=AbstractPlus, http://www.ncbi.nlm.nih.gov/pubmed/17073663?dopt=AbstractPlus, http://www.ncbi.nlm.nih.gov/pubmed/17373882?dopt=AbstractPlus, http://www.ncbi.nlm.nih.gov/pubmed/20621123?dopt=AbstractPlus]).

### Further reading on 5‐HT_3_ receptors

Andrews PL *et al.* (2014) Nausea and the quest for the perfect anti‐emetic. *Eur. J. Pharmacol.* **722**: 108‐21 https://www.ncbi.nlm.nih.gov/pubmed/24157981?dopt=AbstractPlus

Fakhfouri G *et al.* (2015) From Chemotherapy‐Induced Emesis to Neuroprotection: Therapeutic Opportunities for 5‐HT_3_ Receptor Antagonists. *Mol. Neurobiol.* **52**: 1670‐1679 https://www.ncbi.nlm.nih.gov/pubmed/25377794?dopt=AbstractPlus

Gupta D *et al.* (2016) 5‐HT3 receptors: Target for new antidepressant drugs. *Neurosci Biobehav Rev* **64**: 311‐25 https://www.ncbi.nlm.nih.gov/pubmed/26976353?dopt=AbstractPlus

Hoyer D *et al.* (1994) International Union of Pharmacology classification of receptors for 5‐hydroxytryptamine (Serotonin). *Pharmacol. Rev.* **46**: 157‐203 https://www.ncbi.nlm.nih.gov/pubmed/7938165?dopt=AbstractPlus

Lochner M *et al.* (2015) A review of fluorescent ligands for studying 5‐HT_3_ receptors. *Neuropharmacology* **98**: 31‐40 https://www.ncbi.nlm.nih.gov/pubmed/25892507?dopt=AbstractPlus

Rojas C *et al.* (2014) Molecular mechanisms of 5‐HT(3) and NK(1) receptor antagonists in prevention of emesis. *Eur. J. Pharmacol.* **722**: 26‐37 https://www.ncbi.nlm.nih.gov/pubmed/24184669?dopt=AbstractPlus

## http://www.guidetopharmacology.org/GRAC/FamilyDisplayForward?familyId=118

### Overview

Acid‐sensing ion channels (ASICs, **nomenclatureas agreed by NC‐IUPHAR** [http://www.ncbi.nlm.nih.gov/pubmed/25287517?dopt=AbstractPlus]) are members of a Na^+^ channel superfamily that includes the epithelial Na^+^ channel (ENaC), the FMRF‐amide activated channel (FaNaC) of invertebrates, the degenerins (DEG) of *Caenorhabitis elegans*, channels in *Drosophila melanogaster* and ’orphan’ channels that include BLINaC [http://www.ncbi.nlm.nih.gov/pubmed/10457052?dopt=AbstractPlus] and INaC [http://www.ncbi.nlm.nih.gov/pubmed/10767424?dopt=AbstractPlus] that have also been named BASICs, for bile acidactivated ion channels [http://www.ncbi.nlm.nih.gov/pubmed/24365967?dopt=AbstractPlus]. ASIC subunits contain two TM domains and assemble as homo‐ or hetero‐trimers [http://www.ncbi.nlm.nih.gov/pubmed/24507937?dopt=AbstractPlus, http://www.ncbi.nlm.nih.gov/pubmed/19641589?dopt=AbstractPlus, http://www.ncbi.nlm.nih.gov/pubmed/17882215?dopt=AbstractPlus] to form proton‐gated, voltage‐insensitive, Na^+^ permeable, channels (reviewed in [http://www.ncbi.nlm.nih.gov/pubmed/25585135?dopt=AbstractPlus, http://www.ncbi.nlm.nih.gov/pubmed/23783197?dopt=AbstractPlus]). Splice variants of ASIC1 [termed ASIC1a (ASIC, ASICα, BNaC2α) [http://www.ncbi.nlm.nih.gov/pubmed/9062189?dopt=AbstractPlus], ASIC1b (ASICβ, BNaC2β) [http://www.ncbi.nlm.nih.gov/pubmed/9707631?dopt=AbstractPlus] and ASIC1b2 (ASICβ2) [http://www.ncbi.nlm.nih.gov/pubmed/11588592?dopt=AbstractPlus]; note that ASIC1a is also permeable to Ca^2+^] and ASIC2 [termed ASIC2a (MDEG1, BNaC1α, BNC1α) [http://www.ncbi.nlm.nih.gov/pubmed/9037075?dopt=AbstractPlus, http://www.ncbi.nlm.nih.gov/pubmed/8626462?dopt=AbstractPlus, http://www.ncbi.nlm.nih.gov/pubmed/8631835?dopt=AbstractPlus] and ASIC2b (MDEG2, BNaC1β) [http://www.ncbi.nlm.nih.gov/pubmed/9368048?dopt=AbstractPlus]] have been cloned. Unlike ASIC2a (listed in table), heterologous expression of ASIC2b alone does not support H^+^‐gated currents. A third member, ASIC3 (DRASIC, TNaC1) [http://www.ncbi.nlm.nih.gov/pubmed/9261094?dopt=AbstractPlus], has been identified. A fourth mammalian member of the family (ASIC4/SPASIC) does not support a proton‐gated channel in heterologous expression systems and is reported to downregulate the expression of ASIC1a and ASIC3 [http://www.ncbi.nlm.nih.gov/pubmed/10923674?dopt=AbstractPlus, http://www.ncbi.nlm.nih.gov/pubmed/18662336?dopt=AbstractPlus, http://www.ncbi.nlm.nih.gov/pubmed/10852210?dopt=AbstractPlus, http://www.ncbi.nlm.nih.gov/pubmed/25828470?dopt=AbstractPlus]. ASIC channels are primarily expressed in central and peripheral neurons including nociceptors where they participate in neuronal sensitivity to acidosis. They have also been detected in taste receptor cells (ASIC13), photoreceptors and retinal cells (ASIC1‐3), cochlear hair cells (ASIC1b), testis (hASIC3), pituitary gland (ASIC4), lung epithelial cells (ASIC1a and ‐3), urothelial cells, adipose cells (ASIC3), vascular smooth muscle cells (ASIC1‐3), immune cells (ASIC1,‐3 and ‐4) and bone (ASIC1‐3). A neurotransmitter‐like function of protons has been suggested, involving postsynaptically located ASICs of the CNS in functions such as learning and fear perception [http://www.ncbi.nlm.nih.gov/pubmed/24889629?dopt=AbstractPlus, http://www.ncbi.nlm.nih.gov/pubmed/24952644?dopt=AbstractPlus, http://www.ncbi.nlm.nih.gov/pubmed/19945383?dopt=AbstractPlus], responses to focal ischemia [http://www.ncbi.nlm.nih.gov/pubmed/17127388?dopt=AbstractPlus] and to axonal degeneration in autoimmune inflammation in a mouse model of multiple sclerosis [http://www.ncbi.nlm.nih.gov/pubmed/17994101?dopt=AbstractPlus], as well as seizures [http://www.ncbi.nlm.nih.gov/pubmed/18536711?dopt=AbstractPlus] and pain [http://www.ncbi.nlm.nih.gov/pubmed/22094702?dopt=AbstractPlus, http://www.ncbi.nlm.nih.gov/pubmed/21508231?dopt=AbstractPlus, http://www.ncbi.nlm.nih.gov/pubmed/18923424?dopt=AbstractPlus, http://www.ncbi.nlm.nih.gov/pubmed/23034652?dopt=AbstractPlus]. Heterologously expressed heteromultimers form ion channels with differences in kinetics, ion selectivity, pH‐ sensitivity and sensitivity to blockers that resemble some of the native proton activated currents recorded from neurones [http://www.ncbi.nlm.nih.gov/pubmed/10842183?dopt=AbstractPlus, http://www.ncbi.nlm.nih.gov/pubmed/18256271?dopt=AbstractPlus, http://www.ncbi.nlm.nih.gov/pubmed/10829030?dopt=AbstractPlus, http://www.ncbi.nlm.nih.gov/pubmed/9368048?dopt=AbstractPlus].

**Table:** 

Nomenclature	http://www.guidetopharmacology.org/GRAC/ObjectDisplayForward?objectId=684	http://www.guidetopharmacology.org/GRAC/ObjectDisplayForward?objectId=685
HGNC, UniProt	https://www.genenames.org/data/gene‐symbol‐report/#!/hgnc_id/HGNC:100, http://www.uniprot.org/uniprot/P78348	https://www.genenames.org/data/gene‐symbol‐report/#!/hgnc_id/HGNC:99, http://www.uniprot.org/uniprot/Q16515
Endogenous activators	Extracellular http://www.guidetopharmacology.org/GRAC/LigandDisplayForward?ligandId=2346 ^+^ (ASIC1a) (pEC_50_ ∼6.2–6.8), Extracellular http://www.guidetopharmacology.org/GRAC/LigandDisplayForward?ligandId=2346 ^+^ (ASIC1b) (pEC_50_ ∼5.1–6.2)	Extracellular http://www.guidetopharmacology.org/GRAC/LigandDisplayForward?ligandId=2346 ^+^ (pEC_50_ ∼4.1–5)
Channel blockers	http://www.guidetopharmacology.org/GRAC/LigandDisplayForward?ligandId=10306 (ASIC1a) (pIC_50_ ∼9.3) [http://www.ncbi.nlm.nih.gov/pubmed/28320947?dopt=AbstractPlus], http://www.guidetopharmacology.org/GRAC/LigandDisplayForward?ligandId=4292 (ASIC1a) (pIC_50_ 9), http://www.guidetopharmacology.org/GRAC/LigandDisplayForward?ligandId=10305 (ASIC1a) (pIC_50_ ∼8.5) [http://www.ncbi.nlm.nih.gov/pubmed/28327374?dopt=AbstractPlus], http://www.guidetopharmacology.org/GRAC/LigandDisplayForward?ligandId=566 (ASIC1a) (pIC_50_ ∼8.2), http://www.guidetopharmacology.org/GRAC/LigandDisplayForward?ligandId=10308 (ASIC1a) (pIC_50_ ∼7.3) [http://www.ncbi.nlm.nih.gov/pubmed/23034652?dopt=AbstractPlus], http://www.guidetopharmacology.org/GRAC/LigandDisplayForward?ligandId=10308 (ASIC1b) (pIC_50_ ∼7) [http://www.ncbi.nlm.nih.gov/pubmed/23624383?dopt=AbstractPlus], http://www.guidetopharmacology.org/GRAC/LigandDisplayForward?ligandId=10307 (ASIC1a & ASIC1b) (pIC_50_ ∼6.5) [http://www.ncbi.nlm.nih.gov/pubmed/29134638?dopt=AbstractPlus], http://www.guidetopharmacology.org/GRAC/LigandDisplayForward?ligandId=2525 (ASIC1b) (pIC_50_ ∼5.8), http://www.guidetopharmacology.org/GRAC/LigandDisplayForward?ligandId=4116 (ASIC1a) (pIC_50_ ∼5.7) [http://www.ncbi.nlm.nih.gov/pubmed/16061325?dopt=AbstractPlus] – Rat, http://www.guidetopharmacology.org/GRAC/LigandDisplayForward?ligandId=2525 (ASIC1a) (pIC_50_ ∼5.4), http://www.guidetopharmacology.org/GRAC/LigandDisplayForward?ligandId=2421 (ASIC1a) (pIC_50_ 5), http://www.guidetopharmacology.org/GRAC/LigandDisplayForward?ligandId=4145 (ASIC1a) (pIC_50_ 5), http://www.guidetopharmacology.org/GRAC/LigandDisplayForward?ligandId=4186 (ASIC1a) (pIC_50_ 5), http://www.guidetopharmacology.org/GRAC/LigandDisplayForward?ligandId=4262 (ASIC1a) (pIC_50_ ∼4.9), http://www.guidetopharmacology.org/GRAC/LigandDisplayForward?ligandId=2421 (ASIC1b) (pIC_50_ 4.6–4.7), http://www.guidetopharmacology.org/GRAC/LigandDisplayForward?ligandId=4194 (ASIC1a) (pIC_50_ 3.5) [http://www.ncbi.nlm.nih.gov/pubmed/11588175?dopt=AbstractPlus] – Rat, http://www.guidetopharmacology.org/GRAC/LigandDisplayForward?ligandId=2713 (ASIC1a) (pIC_50_ ∼3.5), http://www.guidetopharmacology.org/GRAC/LigandDisplayForward?ligandId=2476 (ASIC1a) (pIC_50_ ∼3.2)	http://www.guidetopharmacology.org/GRAC/LigandDisplayForward?ligandId=10307 (pIC_50_ ∼6.1) [http://www.ncbi.nlm.nih.gov/pubmed/29134638?dopt=AbstractPlus], http://www.guidetopharmacology.org/GRAC/LigandDisplayForward?ligandId=2421 (pIC_50_ 4.6), http://www.guidetopharmacology.org/GRAC/LigandDisplayForward?ligandId=4116 (pIC_50_ ∼4.5), http://www.guidetopharmacology.org/GRAC/LigandDisplayForward?ligandId=4262 (pIC_50_ ∼4.2), http://www.guidetopharmacology.org/GRAC/LigandDisplayForward?ligandId=2440 (pIC_50_ ∼3)
Labelled ligands	http://www.guidetopharmacology.org/GRAC/LigandDisplayForward?ligandId=4073 (ASIC1a) (p*K*_d_ 9.7)	–
Functional Characteristics	**ASIC1a**: γ= 14pS P_Na_/P_K_ = 5‐13, P_Na_/P_Ca_ =2.5 rapid activation rate (5.8‐13.7 ms), rapid inactivation rate (1.2‐4 s) @ pH 6.0, slow recovery (5.3‐13s) @ pH 7.4 **ASIC1b**: γ= 19 pS P_Na_/P_K_ =14.0, P_Na_ PCa rapid activation rate (9.9 ms), rapid inactivation rate (0.9‐1.7 s) @ pH 6.0, slow recovery (4.4‐7.7 s) @ pH 7.4	= γ10.4‐13.4 pS P_Na_/PK =10, P_Na_/PCa = 20 rapid activation rate, moderate inactivation rate (3.3‐5.5 s) @ pH 5
Comments	ASIC1a and ASIC1b are activated by the heteromeric Texas coral snake toxin MitTx, with pEC_50_ values of 8 and 7.6 respectively [http://www.ncbi.nlm.nih.gov/pubmed/22094702?dopt=AbstractPlus].	ASIC2 is also blocked by diarylamidines

**Table:** 

Nomenclature	http://www.guidetopharmacology.org/GRAC/ObjectDisplayForward?objectId=686
HGNC, UniProt	https://www.genenames.org/data/gene‐symbol‐report/#!/hgnc_id/HGNC:101, http://www.uniprot.org/uniprot/Q9UHC3
Endogenous activators	Extracellular http://www.guidetopharmacology.org/GRAC/LigandDisplayForward?ligandId=2346 ^+^ (transient component) (pEC_50_ ∼6.2–6.7), Extracellular http://www.guidetopharmacology.org/GRAC/LigandDisplayForward?ligandId=2346 ^+^ (sustained component) (pEC_50_ ∼3.5–4.3)
Activators	http://www.guidetopharmacology.org/GRAC/LigandDisplayForward?ligandId=4203 (largly non‐desensitizing; at pH 7.4) (pEC_50_ ∼3), http://www.guidetopharmacology.org/GRAC/LigandDisplayForward?ligandId=4137 (at pH 7.4) (pEC_50_ ∼2.9), http://www.guidetopharmacology.org/GRAC/LigandDisplayForward?ligandId=4127 (at pH 7.4) (pEC_50_ ∼2)
Channel blockers	http://www.guidetopharmacology.org/GRAC/LigandDisplayForward?ligandId=4135 (transient component only) (pIC_50_ 7.2), http://www.guidetopharmacology.org/GRAC/LigandDisplayForward?ligandId=10307 (pIC_50_ ∼6.5) [http://www.ncbi.nlm.nih.gov/pubmed/29134638?dopt=AbstractPlus], http://www.guidetopharmacology.org/GRAC/LigandDisplayForward?ligandId=4116 (pIC_50_ 6) [http://www.ncbi.nlm.nih.gov/pubmed/22778804?dopt=AbstractPlus], http://www.guidetopharmacology.org/GRAC/LigandDisplayForward?ligandId=4262 (transient component) (pIC_50_ ∼5.6), http://www.guidetopharmacology.org/GRAC/LigandDisplayForward?ligandId=2421 (transient component only ‐ sustained component enhanced by 200*μ*M amiloride at pH 4) (pIC_50_ 4.2–4.8), http://www.guidetopharmacology.org/GRAC/LigandDisplayForward?ligandId=2426 (pIC_50_ 4.4), http://www.guidetopharmacology.org/GRAC/LigandDisplayForward?ligandId=566 (pIC_50_ 4.2), http://www.guidetopharmacology.org/GRAC/LigandDisplayForward?ligandId=4139 (sustained component) (pIC_50_ 4) [http://www.ncbi.nlm.nih.gov/pubmed/11588175?dopt=AbstractPlus], http://www.guidetopharmacology.org/GRAC/LigandDisplayForward?ligandId=2714 (sustained component) (pIC_50_ 4), http://www.guidetopharmacology.org/GRAC/LigandDisplayForward?ligandId=4306 (sustained component) (pIC_50_ 3.6)
Functional Characteristics	γ= 13‐15 pS; biphasic response consisting of rapidly inactivating transient and sustained components; very rapid activation (<5 ms) and inactivation (0.4 s); fast recovery (0.4‐0.6 s) @ pH 7.4, transient component partially inactivated at pH 7.2
Comments	ASIC3 is activated by Mit‐Toxin (pEC_50_ 6.1) [http://www.ncbi.nlm.nih.gov/pubmed/22094702?dopt=AbstractPlus].

### Comments

http://www.guidetopharmacology.org/GRAC/LigandDisplayForward?ligandId=4292 (PcTx1) inhibits ASIC1a by increasing the affinity toH+and promoting channel desensitization [http://www.ncbi.nlm.nih.gov/pubmed/15955877?dopt=AbstractPlus, http://www.ncbi.nlm.nih.gov/pubmed/10829030?dopt=AbstractPlus]. PcTx1 has little effect on ASIC2a, ASIC3 or ASIC1a expressed as a heteromultimer with either ASIC2a, or ASIC3 but does inhibit ASIC1a expressed as a heteromultimer with ASIC2b [http://www.ncbi.nlm.nih.gov/pubmed/21715637?dopt=AbstractPlus]. PcTx1 and http://www.guidetopharmacology.org/GRAC/LigandDisplayForward?ligandId=10305 potentiate ASIC1b currents [http://www.ncbi.nlm.nih.gov/pubmed/16505147?dopt=AbstractPlus, http://www.ncbi.nlm.nih.gov/pubmed/28327374?dopt=AbstractPlus]. ASIC1containing homo‐ and heteromers are inhibited by Mambalgins, toxins contained in the black mamba venom, which induce in ASIC1a an acidic shift of the pH dependence of activation [http://www.ncbi.nlm.nih.gov/pubmed/23034652?dopt=AbstractPlus]. http://www.guidetopharmacology.org/GRAC/LigandDisplayForward?ligandId=10306 is highly selective for ASIC1a with very little activity at ASIC1b. It inhibits channel activation and is very slowly reversible [http://www.ncbi.nlm.nih.gov/pubmed/28320941?dopt=AbstractPlus]. http://www.guidetopharmacology.org/GRAC/LigandDisplayForward?ligandId=4135 most potently blocks homomeric ASIC3 channels, but also ASIC2b+ASIC3, ASIC1b+ASIC3, and ASIC1a+ASIC3 heteromeric channels with IC_50_ values of 117 nM, 900 nM and 2 *μ*M, respectively. http://www.guidetopharmacology.org/GRAC/LigandDisplayForward?ligandId=4135 has no effect on ASIC1a or ASIC2a+ASIC3, however, it does potentiate ASIC1b and ASIC2a homomers in the low micromolar range(1‐10*μ*M) [http://www.ncbi.nlm.nih.gov/pubmed/15044953?dopt=AbstractPlus, http://www.ncbi.nlm.nih.gov/pubmed/17113616?dopt=AbstractPlus, http://www.ncbi.nlm.nih.gov/pubmed/29134638?dopt=AbstractPlus]. http://www.guidetopharmacology.org/GRAC/LigandDisplayForward?ligandId=4135however also inhibits voltage‐gated Na^+^ channels [http://www.ncbi.nlm.nih.gov/pubmed/21943094?dopt=AbstractPlus, http://www.ncbi.nlm.nih.gov/pubmed/22972919?dopt=AbstractPlus]. IC_50_ value for http://www.guidetopharmacology.org/GRAC/LigandDisplayForward?ligandId=4116 was determined using high throughput electrophysiology on human ASIC3 expressed in HEK293 cells [http://www.ncbi.nlm.nih.gov/pubmed/22778804?dopt=AbstractPlus]. The pEC_50_ values for proton activation of ASIC channels are influenced by numerous factors including extracellular di‐ and poly‐valent ions, Zn^2+^, protein kinase C and serine proteases (reviewed in [http://www.ncbi.nlm.nih.gov/pubmed/25287517?dopt=AbstractPlus, http://www.ncbi.nlm.nih.gov/pubmed/23783197?dopt=AbstractPlus]). Rapid acidification is required for activation of ASIC1 and ASIC3 due to fast inactivation/desensitization. pEC_50_ values for H^+^‐activation of either transient, or sustained, currents mediated by ASIC3 vary in the literature and may reflect species and/or methodological differences [http://www.ncbi.nlm.nih.gov/pubmed/9886053?dopt=AbstractPlus, http://www.ncbi.nlm.nih.gov/pubmed/9744806?dopt=AbstractPlus, http://www.ncbi.nlm.nih.gov/pubmed/9261094?dopt=AbstractPlus]. The transient ASIC current component is Na^+^‐selective (PNa/PK of about 10) [http://www.ncbi.nlm.nih.gov/pubmed/9261094?dopt=AbstractPlus, http://www.ncbi.nlm.nih.gov/pubmed/25114023?dopt=AbstractPlus] whereas the sustained current component that is observed with ASIC3 and some ASIC heteromers is non‐selective between Na^+^ and K^+^ [http://www.ncbi.nlm.nih.gov/pubmed/9744806?dopt=AbstractPlus]. The reducing agents dithiothreitol (DTT) and glutathione (GSH) increase ASIC1a currents expressed in CHO cells and ASIC‐like currents in sensory ganglia and central neurons [http://www.ncbi.nlm.nih.gov/pubmed/16085050?dopt=AbstractPlus, http://www.ncbi.nlm.nih.gov/pubmed/16707785?dopt=AbstractPlus] whereas oxidation, through the formation of intersubunit disulphide bonds, reduces currents mediated by ASIC1a [http://www.ncbi.nlm.nih.gov/pubmed/19218436?dopt=AbstractPlus]. ASIC1a is also irreversibly modulated by extracellular serine proteases, such as trypsin, through proteolytic cleavage [http://www.ncbi.nlm.nih.gov/pubmed/16282326?dopt=AbstractPlus]. Non‐steroidal anti‐inflammatory drugs (NSAIDs) are direct inhibitors of ASIC currents (reviewed in [http://www.ncbi.nlm.nih.gov/pubmed/25613302?dopt=AbstractPlus]). Extracellular Zn^2+^ potentiates proton activation of homomeric and heteromeric channels incorporating ASIC2a, but not homomeric ASIC1a or ASIC3 channels [http://www.ncbi.nlm.nih.gov/pubmed/11457851?dopt=AbstractPlus]. However, removal of contaminating Zn^2+^ by chelation reveals a high affinity block of homomeric ASIC1a and heteromeric ASIC1a+ASIC2 channels by Zn^2+^ indicating complex biphasic actions of the divalent [http://www.ncbi.nlm.nih.gov/pubmed/15470133?dopt=AbstractPlus]. Nitric oxide potentiates submaximal currents activated by H^+^ mediated by ASIC1a, ASIC1b, ASIC2a and ASIC3 [http://www.ncbi.nlm.nih.gov/pubmed/18045919?dopt=AbstractPlus]. Ammonium ions activate ASIC channels (most likely ASIC1a) in midbrain dopaminergic neurones: that may be relevant to neuronal disorders associated with hyperammonemia [http://www.ncbi.nlm.nih.gov/pubmed/16847263?dopt=AbstractPlus]. The positive modulation of homomeric, heteromeric and native ASIC channels by the peptide http://www.guidetopharmacology.org/GRAC/LigandDisplayForward?ligandId=4195 and related substances, such as neuropeptides FF and SF, is reviewed in detail in [http://www.ncbi.nlm.nih.gov/pubmed/25592215?dopt=AbstractPlus]. Inflammatory conditions and particular pro‐inflammatory mediators such as http://www.guidetopharmacology.org/GRAC/LigandDisplayForward?ligandId=2391 induce overexpression of ASIC‐encoding genes and enhance ASIC currents [http://www.ncbi.nlm.nih.gov/pubmed/18923424?dopt=AbstractPlus, http://www.ncbi.nlm.nih.gov/pubmed/12486159?dopt=AbstractPlus, http://www.ncbi.nlm.nih.gov/pubmed/17258862?dopt=AbstractPlus]. The sustained current component mediated by ASIC3 is potentiated by hypertonic solutions in a manner that is synergistic with the effect of http://www.guidetopharmacology.org/GRAC/LigandDisplayForward?ligandId=2391 [http://www.ncbi.nlm.nih.gov/pubmed/18923424?dopt=AbstractPlus]. ASIC3 is partially activated by the lipids lysophosphatidylcholine (LPC) and arachidonic acid [http://www.ncbi.nlm.nih.gov/pubmed/26772186?dopt=AbstractPlus]. Mit‐Toxin, which is contained in the venom of the Texas coral snake, activates several ASIC subtypes [http://www.ncbi.nlm.nih.gov/pubmed/22094702?dopt=AbstractPlus]. Selective activation of ASIC3 by GMQ at a site separate from the proton binding site is potentiated by mild acidosis and reduced extracellular Ca^2+^ [http://www.ncbi.nlm.nih.gov/pubmed/20920791?dopt=AbstractPlus].

### Further reading on Acid‐sensing (proton‐gated) ion channels (ASICs)

Baron A *et al.* (2015) Pharmacology of acid‐sensing ion channels ‐ Physiological and therapeutical perspectives. *Neuropharmacology* **94**: 19‐35 https://www.ncbi.nlm.nih.gov/pubmed/25613302?dopt=AbstractPlus

Boscardin E *et al.* (2016) The function and regulation of acid‐sensing ion channels (ASICs) and the epithelial Na(+) channel (ENaC): IUPHAR Review 19. *Br. J. Pharmacol.* **173**: 2671‐701 https://www.ncbi.nlm.nih.gov/pubmed/27278329?dopt=AbstractPlus

Cristofori‐Armstrong B *et al.* (2017) Acid‐sensing ion channel (ASIC) structure and function: Insights from spider, snake and sea anemone venoms. *Neuropharmacology* **127**: 173‐184 https://www.ncbi.nlm.nih.gov/pubmed/28457973?dopt=AbstractPlus

Gründer S *et al.* (2015) Biophysical properties of acid‐sensing ion channels (ASICs). *Neuropharmacology* **94**: 9‐18 https://www.ncbi.nlm.nih.gov/pubmed/25585135?dopt=AbstractPlus

Hanukoglu I. (2017) ASIC and ENaC type sodium channels: conformational states and the structures of the ion selectivity filters. *FEBS J.* **284**: 525‐545 https://www.ncbi.nlm.nih.gov/pubmed/27580245?dopt=AbstractPlus

Kellenberger S *et al.* (2015) International Union of Basic and Clinical Pharmacology. XCI. structure, function, and pharmacology of acid‐sensing ion channels and the epithelial Na+ channel. *Pharmacol. Rev.* **67**: 1‐35 https://www.ncbi.nlm.nih.gov/pubmed/25287517?dopt=AbstractPlus

Rash LD. (2017) Acid‐Sensing Ion Channel Pharmacology, Past, Present, and Future …. *Adv. Pharmacol.* **79**: 35‐66 https://www.ncbi.nlm.nih.gov/pubmed/28528673?dopt=AbstractPlus

## http://www.guidetopharmacology.org/GRAC/FamilyDisplayForward?familyId=122

### Overview

The epithelial sodium channels (ENaC) are located on the apical membrane of epithelial cells in the distal kidney tubules, lung, respiratory tract, male and female reproductive tracts, sweat and salivary glands, placenta, colon and some other organs [http://www.ncbi.nlm.nih.gov/pubmed/7810611?dopt=AbstractPlus, http://www.ncbi.nlm.nih.gov/pubmed/7806569?dopt=AbstractPlus, http://www.ncbi.nlm.nih.gov/pubmed/26772908?dopt=AbstractPlus]. In these epithelia, ENaC allows flow of Na^+^ ions from the extracellular fluid in the lumen into the epithelial cell. Na^+^ ions are then pumped out of the cytoplasm into the interstitial fluid by the Na^+^/K^+^ ATPase located on the basolateral membrane [http://www.ncbi.nlm.nih.gov/pubmed/16139686?dopt=AbstractPlus]. As Na^+^ is one of the major electrolytes in the extracellular fluid (ECF), osmolarity change initiated by the Na^+^ flow is accompanied by a flow of water accompanying Na^+^ ions [http://www.ncbi.nlm.nih.gov/pubmed/18509340?dopt=AbstractPlus]. Thus, ENaC has a central role in the regulation of ECF volume and blood pressure, especially via its function in the kidney [http://www.ncbi.nlm.nih.gov/pubmed/25287517?dopt=AbstractPlus, http://www.ncbi.nlm.nih.gov/pubmed/25540145?dopt=AbstractPlus]. The expression of ENaC subunits, hence its activity, is regulated by the renin‐angotensin‐aldosterone system, and other factors that are involved in electrolyte homeostasis [http://www.ncbi.nlm.nih.gov/pubmed/8770001?dopt=AbstractPlus, http://www.ncbi.nlm.nih.gov/pubmed/22038262?dopt=AbstractPlus, http://www.ncbi.nlm.nih.gov/pubmed/25540145?dopt=AbstractPlus]. In the respiratory tract and female reproductive tract large segments of the tracts are covered by multi‐ciliated cells. In these cells ENaC has been shown to be located along the entire length of the cilia [http://www.ncbi.nlm.nih.gov/pubmed/22207244?dopt=AbstractPlus]. Cilial location greatly increases ENaC density per cell surface and allows ENaC to serve as a sensitive regulator of osmolarity of the periciliary fluid throughout the whole depth of the fluid bathing the cilia [http://www.ncbi.nlm.nih.gov/pubmed/22207244?dopt=AbstractPlus]. In contrast to ENaC, CFTR that is defective in cystic fibrosis is not located on non‐cilial cell‐surface [http://www.ncbi.nlm.nih.gov/pubmed/22207244?dopt=AbstractPlus]. Thus, ENaC function is also essential for the clearance of respiratory airways, transport of germ cells, fertilization, implantation and cell migration [http://www.ncbi.nlm.nih.gov/pubmed/22207244?dopt=AbstractPlus, http://www.ncbi.nlm.nih.gov/pubmed/8389146?dopt=AbstractPlus]. ENaC has beenrecently localized in the germinal epithelium of the testis, Sertoli cells, spermatozoa, along the epididymis ducts, and smooth muscle cells [http://www.ncbi.nlm.nih.gov/pubmed/29453757?dopt=AbstractPlus, http://www.ncbi.nlm.nih.gov/pubmed/30659401?dopt=AbstractPlus]. Evidence has been provided that rare mutations in ENaC are associated with female infertility [http://www.ncbi.nlm.nih.gov/pubmed/29885352?dopt=AbstractPlus].

**Table:** 

Nomenclature	http://www.guidetopharmacology.org/GRAC/ObjectDisplayForward?objectId=742
Subunits	http://www.guidetopharmacology.org/GRAC/ObjectDisplayForward?objectId=739, http://www.guidetopharmacology.org/GRAC/ObjectDisplayForward?objectId=738, http://www.guidetopharmacology.org/GRAC/ObjectDisplayForward?objectId=741
Activators	http://www.guidetopharmacology.org/GRAC/LigandDisplayForward?ligandId=4305 (pEC_50_ 5.9) [http://www.ncbi.nlm.nih.gov/pubmed/18326490?dopt=AbstractPlus]
Channel blockers	http://www.guidetopharmacology.org/GRAC/LigandDisplayForward?ligandId=4280 (pIC_50_ 8.1), http://www.guidetopharmacology.org/GRAC/LigandDisplayForward?ligandId=4145 (pIC_50_ ∼8), http://www.guidetopharmacology.org/GRAC/LigandDisplayForward?ligandId=2421 (pIC_50_ 6.7–7), http://www.guidetopharmacology.org/GRAC/LigandDisplayForward?ligandId=4329 (pIC_50_ ∼5.3) [http://www.ncbi.nlm.nih.gov/pubmed/8107805?dopt=AbstractPlus, http://www.ncbi.nlm.nih.gov/pubmed/14500741?dopt=AbstractPlus]

### Subunits

**Table:** 

Nomenclature	http://www.guidetopharmacology.org/GRAC/ObjectDisplayForward?objectId=738	http://www.guidetopharmacology.org/GRAC/ObjectDisplayForward?objectId=739	http://www.guidetopharmacology.org/GRAC/ObjectDisplayForward?objectId=741	http://www.guidetopharmacology.org/GRAC/ObjectDisplayForward?objectId=740
HGNC, UniProt	https://www.genenames.org/data/gene‐symbol‐report/#!/hgnc_id/HGNC:10599, http://www.uniprot.org/uniprot/P37088	https://www.genenames.org/data/gene‐symbol‐report/#!/hgnc_id/HGNC:10600, http://www.uniprot.org/uniprot/P51168	https://www.genenames.org/data/gene‐symbol‐report/#!/hgnc_id/HGNC:10602, http://www.uniprot.org/uniprot/P51170	https://www.genenames.org/data/gene‐symbol‐report/#!/hgnc_id/HGNC:10601, http://www.uniprot.org/uniprot/P51172

### Further reading on Epithelial sodium channel (ENaC)

Berman JM *et al.* (2015) A long isoform of the epithelial sodium channel alpha subunit forms a highly active channel. *Channels (Austin)* **9**: 30‐43 https://www.ncbi.nlm.nih.gov/pubmed/25517724?dopt=AbstractPlus

Boscardin E *et al.* (2016) The function and regulation of acid‐sensing ion channels (ASICs) and the epithelial Na(+) channel (ENaC): IUPHAR Review 19. *Br. J. Pharmacol.* **173**: 2671‐701 https://www.ncbi.nlm.nih.gov/pubmed/27278329?dopt=AbstractPlus

Bourque CW. (2008) Central mechanisms of osmosensation and systemic osmoregulation. *Nat. Rev. Neurosci.* **9**: 519‐31 https://www.ncbi.nlm.nih.gov/pubmed/18509340?dopt=AbstractPlus

Eaton DC *et al.* (2009) The contribution of epithelial sodium channels to alveolar function in health and disease. *Annu. Rev. Physiol.* **71**: 403‐23 https://www.ncbi.nlm.nih.gov/pubmed/18831683?dopt=AbstractPlus

Enuka Y *et al.* (2012) Epithelial sodium channels (ENaC) are uniformly distributed on motile cilia in the oviduct and the respiratory airways. *Histochem. Cell Biol.* **137**: 339‐53 https://www.ncbi.nlm.nih.gov/pubmed/22207244?dopt=AbstractPlus

Hanukoglu I. (2017) ASIC and ENaC type sodium channels: conformational states and the structures of the ion selectivity filters. *FEBS J.* **284**: 525‐545 https://www.ncbi.nlm.nih.gov/pubmed/27580245?dopt=AbstractPlus

Hanukoglu I *et al.* (2016) Epithelial sodium channel (ENaC) family: Phylogeny, structure‐function, tissue distribution, and associated inherited diseases. *Gene* **579**: 95‐132 https://www.ncbi.nlm.nih.gov/pubmed/26772908?dopt=AbstractPlus

Kashlan OB *et al.* (2012) Epithelial Na(+) channel regulation by cytoplasmic and extracellular factors. *Exp. Cell Res.* **318**: 1011‐9 https://www.ncbi.nlm.nih.gov/pubmed/22405998?dopt=AbstractPlus

Kellenberger S *et al.* (2015) International Union of Basic and Clinical Pharmacology. XCI. structure, function, and pharmacology of acid‐sensing ion channels and the epithelial Na+ channel. *Pharmacol. Rev.* **67**: 1‐35 https://www.ncbi.nlm.nih.gov/pubmed/25287517?dopt=AbstractPlus

Kleyman TR *et al.* (2009) ENaC at the cutting edge: regulation of epithelial sodium channels by proteases. *J. Biol. Chem.* **284**: 20447‐51 https://www.ncbi.nlm.nih.gov/pubmed/19401469?dopt=AbstractPlus

Palmer LG *et al.* (2012) Regulation and dysregulation of epithelial Na+ channels. *Clin. Exp. Nephrol.* **16**: 35‐43 https://www.ncbi.nlm.nih.gov/pubmed/22038262?dopt=AbstractPlus

Reddy MM *et al.* (2013) Status of fluid and electrolyte absorption in cystic fibrosis. *Cold Spring Harb Perspect Med* **3**: a009555 https://www.ncbi.nlm.nih.gov/pubmed/23284077?dopt=AbstractPlus

Rossier BC *et al.* (2015) Epithelial sodium transport and its control by aldosterone: the story of our internal environment revisited. *Physiol. Rev.* **95**: 297‐340 https://www.ncbi.nlm.nih.gov/pubmed/25540145?dopt=AbstractPlus

Soundararajan R *et al*. (2010) Role of epithelial sodium channels and their regulators in hypertension. *J. Biol. Chem.* **285**: 30363‐9 https://www.ncbi.nlm.nih.gov/pubmed/20624922?dopt=AbstractPlus

## http://www.guidetopharmacology.org/GRAC/FamilyDisplayForward?familyId=72

### Overview

The GABA_A_ receptor is a ligand‐gated ion channel of the Cys‐loop family that includes the nicotinic acetylcholine, 5‐HT_3_ and strychnine‐sensitive glycine receptors. GABA_A_ receptor‐mediated inhibition within the CNS occurs by fast synaptic transmission, sustained tonic inhibition and temporally intermediate events that have been termed ‘GABA_A_, slow’ [http://www.ncbi.nlm.nih.gov/pubmed/21145601?dopt=AbstractPlus]. GABA_A_ receptors exist as pentamers of 4TM subunits that form an intrinsic anion selective channel. Sequences of six α, three β, three γ, one *δ*, three *ρ*, one*∈*, one *π* and one ϑ GABA_A_ receptor subunits have been reported in mammals [http://www.ncbi.nlm.nih.gov/pubmed/18760291?dopt=AbstractPlus, http://www.ncbi.nlm.nih.gov/pubmed/18790874?dopt=AbstractPlus, http://www.ncbi.nlm.nih.gov/pubmed/17175817?dopt=AbstractPlus, http://www.ncbi.nlm.nih.gov/pubmed/23038269?dopt=AbstractPlus]. The *π*‐subunit is restricted to reproductive tissue. Alternatively spliced versions of many subunits exist (e.g. α4‐ and α6‐ (both not functional) α5‐, β2‐, β3‐ and γ2), along with RNA editing of the α3 subunit [http://www.ncbi.nlm.nih.gov/pubmed/19909284?dopt=AbstractPlus]. The three *ρ*‐subunits, (*ρ*1‐3) function as either homo‐or hetero‐oligomeric assemblies [http://www.ncbi.nlm.nih.gov/pubmed/15566397?dopt=AbstractPlus, http://www.ncbi.nlm.nih.gov/pubmed/11239575?dopt=AbstractPlus]. Receptors formed from *ρ*‐subunits, because of their distinctive pharmacology that includes insensitivity tobicuculline, benzodiazepines and barbiturates, have sometimes been termed GABA_C_ receptors [http://www.ncbi.nlm.nih.gov/pubmed/11239575?dopt=AbstractPlus], **but they are classified as GABA_A_ receptors by NC‐IUPHAR on the basis of structural and functional criteria** [http://www.ncbi.nlm.nih.gov/pubmed/9647870?dopt=AbstractPlus, http://www.ncbi.nlm.nih.gov/pubmed/18760291?dopt=AbstractPlus, http://www.ncbi.nlm.nih.gov/pubmed/18790874?dopt=AbstractPlus].

Many GABA_A_ receptor subtypes contain α‐, β‐ and γ‐subunits with the likely stoichiometry 2α.2β.1γ[http://www.ncbi.nlm.nih.gov/pubmed/12126658?dopt=AbstractPlus, http://www.ncbi.nlm.nih.gov/pubmed/18790874?dopt=AbstractPlus]. It is thought that the majority of GABA_A_ receptors harbour a single type of α‐ and β ‐subunit variant. The α1β2γ2 hetero‐oligomer constitutes the largest population of GABA_A_ receptors in the CNS, followed by the α2β3γ2 and α3β3γ2 isoforms. Receptors that incorporate the α4‐ α5‐or α6‐subunit, or the β1‐, γ1‐, γ3‐, *δ*‐, *∈*‐ and ϑ‐subunits, are less numerous, but they may nonetheless serve important functions. For example, extrasynaptically located receptors that contain α6‐ and *δ*‐subunits in cerebellar granule cells, or an α4‐ and *δ*‐subunit in dentate gyrus granule cells and thalamic neurones, mediate a tonic current that is important for neuronal excitability in response to ambient concentrations of GABA [http://www.ncbi.nlm.nih.gov/pubmed/19828786?dopt=AbstractPlus, http://www.ncbi.nlm.nih.gov/pubmed/15738957?dopt=AbstractPlus, http://www.ncbi.nlm.nih.gov/pubmed/15331240?dopt=AbstractPlus, http://www.ncbi.nlm.nih.gov/pubmed/15111008?dopt=AbstractPlus, http://www.ncbi.nlm.nih.gov/pubmed/24027497?dopt=AbstractPlus]. GABA binding occurs at the β+/α‐ subunit interface and the homologous γ+/α‐ subunits interface creates the benzodiazepinesite. A second site for benzodiazepine binding hasrecently been postulated to occur at the α+/β‐ interface ([http://www.ncbi.nlm.nih.gov/pubmed/21248110?dopt=AbstractPlus]; reviewed by [http://www.ncbi.nlm.nih.gov/pubmed/21189125?dopt=AbstractPlus]). The particular α‐and γ‐subunit isoforms exhibit marked effects on recognition and/or efficacy at the benzodiazepine site. Thus, receptors incorporating either α4‐ or α6‐subunits are not recognised by ‘classical’ benzodiazepines, such as http://www.guidetopharmacology.org/GRAC/LigandDisplayForward?ligandId=4193 (but see [http://www.ncbi.nlm.nih.gov/pubmed/20638393?dopt=AbstractPlus]). The trafficking, cell surface expression, internalisation and function of GABA_A_ receptors and their subunits are discussed in detail in several recent reviews [http://www.ncbi.nlm.nih.gov/pubmed/17083446?dopt=AbstractPlus, http://www.ncbi.nlm.nih.gov/pubmed/18382465?dopt=AbstractPlus, http://www.ncbi.nlm.nih.gov/pubmed/21555068?dopt=AbstractPlus, http://www.ncbi.nlm.nih.gov/pubmed/21742794?dopt=AbstractPlus] but one point worthy of note is that receptors incorporating the γ2 subunit (except when associated with α5) cluster at the postsynaptic membrane (but may distribute dynamically between synaptic and extrasynaptic locations), whereas as those incorporating the d subunit appear to be exclusively extrasynaptic.

**NC‐IUPHAR** [http://www.ncbi.nlm.nih.gov/pubmed/9647870?dopt=AbstractPlus, http://www.ncbi.nlm.nih.gov/pubmed/18790874?dopt=AbstractPlus] class the GABA_A_ receptors according to their subunit structure, pharmacology and receptor function. Currently, eleven native GABA_A_ receptors are classed as conclusively identified (*i.e*., α1β2γ2, α1βγ2, α3βγ2, α4βγ2, α4β2*δ*, α4β3*δ*, α5βγ2, α6βγ2, α6β2*δ*, α6β3*δ* and *ρ*) with further receptor isoforms occurring with high probability, or only tentatively [http://www.ncbi.nlm.nih.gov/pubmed/18760291?dopt=AbstractPlus, http://www.ncbi.nlm.nih.gov/pubmed/18790874?dopt=AbstractPlus]. It is beyond the scope of this Guide to discuss the pharmacology of individual GABA_A_ receptor isoforms in detail; such information can be gleaned in the reviews [http://www.ncbi.nlm.nih.gov/pubmed/9647870?dopt=AbstractPlus, http://www.ncbi.nlm.nih.gov/pubmed/12171573?dopt=AbstractPlus, http://www.ncbi.nlm.nih.gov/pubmed/15974965?dopt=AbstractPlus, http://www.ncbi.nlm.nih.gov/pubmed/12126658?dopt=AbstractPlus, http://www.ncbi.nlm.nih.gov/pubmed/12469353?dopt=AbstractPlus, http://www.ncbi.nlm.nih.gov/pubmed/17394533?dopt=AbstractPlus, http://www.ncbi.nlm.nih.gov/pubmed/18760291?dopt=AbstractPlus, http://www.ncbi.nlm.nih.gov/pubmed/18790874?dopt=AbstractPlus, http://www.ncbi.nlm.nih.gov/pubmed/17175817?dopt=AbstractPlus] and [http://www.ncbi.nlm.nih.gov/pubmed/21309116?dopt=AbstractPlus, http://www.ncbi.nlm.nih.gov/pubmed/18482097?dopt=AbstractPlus]. Agents that discriminate between α‐subunit isoforms are noted in the table and additional agents that demonstrate selectivity between receptor isoforms, for example via β‐subunit selectivity, are indicated in the text below. The distinctive agonist and antagonist pharmacology of *ρ* receptors is summarised in the table and additional aspects are reviewed in [http://www.ncbi.nlm.nih.gov/pubmed/15566397?dopt=AbstractPlus, http://www.ncbi.nlm.nih.gov/pubmed/20963487?dopt=AbstractPlus, http://www.ncbi.nlm.nih.gov/pubmed/21428815?dopt=AbstractPlus, http://www.ncbi.nlm.nih.gov/pubmed/11239575?dopt=AbstractPlus].

Several high‐resolution cryo‐electron microscopy structures have been described in which the full‐length human α1β3γ2L GABA_A_ receptor in lipid nanodiscs is bound to the channel‐blocker picrotoxin, the competitive antagonist bicuculline, the agonist GABA (γ‐aminobutyric acid), and the classical benzodiazepines alprazolam and diazepam [http://www.ncbi.nlm.nih.gov/pubmed/30602790?dopt=AbstractPlus].

**Table:** 

Nomenclature	http://www.guidetopharmacology.org/GRAC/ObjectDisplayForward?objectId=404	http://www.guidetopharmacology.org/GRAC/ObjectDisplayForward?objectId=405
HGNC, UniProt	https://www.genenames.org/data/gene‐symbol‐report/#!/hgnc_id/HGNC:4075, http://www.uniprot.org/uniprot/P14867	https://www.genenames.org/data/gene‐symbol‐report/#!/hgnc_id/HGNC:4076, http://www.uniprot.org/uniprot/P47869
Agonists	http://www.guidetopharmacology.org/GRAC/LigandDisplayForward?ligandId=4322 [GABA site], http://www.guidetopharmacology.org/GRAC/LigandDisplayForward?ligandId=4226 [GABA site], http://www.guidetopharmacology.org/GRAC/LigandDisplayForward?ligandId=4227 [GABA site], http://www.guidetopharmacology.org/GRAC/LigandDisplayForward?ligandId=4259 [GABA site], http://www.guidetopharmacology.org/GRAC/LigandDisplayForward?ligandId=4287 [GABA site]	http://www.guidetopharmacology.org/GRAC/LigandDisplayForward?ligandId=4322 [GABA site], http://www.guidetopharmacology.org/GRAC/LigandDisplayForward?ligandId=4226 [GABA site], http://www.guidetopharmacology.org/GRAC/LigandDisplayForward?ligandId=4227 [GABA site], http://www.guidetopharmacology.org/GRAC/LigandDisplayForward?ligandId=4259 [GABA site], http://www.guidetopharmacology.org/GRAC/LigandDisplayForward?ligandId=4287 [GABA site]
Selective antagonists	http://www.guidetopharmacology.org/GRAC/LigandDisplayForward?ligandId=2312 [GABA site], http://www.guidetopharmacology.org/GRAC/LigandDisplayForward?ligandId=4197 [GABA site]	http://www.guidetopharmacology.org/GRAC/LigandDisplayForward?ligandId=2312 [GABA site], http://www.guidetopharmacology.org/GRAC/LigandDisplayForward?ligandId=4197 [GABA site]
Channel blockers	http://www.guidetopharmacology.org/GRAC/LigandDisplayForward?ligandId=4320, http://www.guidetopharmacology.org/GRAC/LigandDisplayForward?ligandId=4051	http://www.guidetopharmacology.org/GRAC/LigandDisplayForward?ligandId=4320, http://www.guidetopharmacology.org/GRAC/LigandDisplayForward?ligandId=4051
Endogenous allosteric modulators	http://www.guidetopharmacology.org/GRAC/LigandDisplayForward?ligandId=4108 (Potentiation), http://www.guidetopharmacology.org/GRAC/LigandDisplayForward?ligandId=566 (Inhibition), http://www.guidetopharmacology.org/GRAC/LigandDisplayForward?ligandId=4321 (Potentiation)	http://www.guidetopharmacology.org/GRAC/LigandDisplayForward?ligandId=4108 (Potentiation), http://www.guidetopharmacology.org/GRAC/LigandDisplayForward?ligandId=566 (Inhibition), http://www.guidetopharmacology.org/GRAC/LigandDisplayForward?ligandId=4321 (Potentiation)
Allosteric modulators	http://www.guidetopharmacology.org/GRAC/LigandDisplayForward?ligandId=4192 [benzodiazepine site] (Antagonist) (p*K*_i_ 9.1) [http://www.ncbi.nlm.nih.gov/pubmed/9767648?dopt=AbstractPlus], http://www.guidetopharmacology.org/GRAC/LigandDisplayForward?ligandId=6963 (Positive) (p*K*_i_ 8.9) [http://www.ncbi.nlm.nih.gov/pubmed/2551039?dopt=AbstractPlus], http://www.guidetopharmacology.org/GRAC/LigandDisplayForward?ligandId=4193 [benzodiazepine site] (Positive) (p*K*_i_ 8.3) [http://www.ncbi.nlm.nih.gov/pubmed/8632757?dopt=AbstractPlus], http://www.guidetopharmacology.org/GRAC/LigandDisplayForward?ligandId=3364 [benzodiazepine site] (Positive) (p*K*_i_ 7.8) [http://www.ncbi.nlm.nih.gov/pubmed/2551039?dopt=AbstractPlus], http://www.guidetopharmacology.org/GRAC/LigandDisplayForward?ligandId=7111 [benzodiazepine site] (Positive) (pEC_50_ 7.4) [http://www.ncbi.nlm.nih.gov/pubmed/12408715?dopt=AbstractPlus], http://www.guidetopharmacology.org/GRAC/LigandDisplayForward?ligandId=4094 [benzodiazepine site] (Inverse agonist), http://www.guidetopharmacology.org/GRAC/LigandDisplayForward?ligandId=4095 [benzodiazepine site] (Inverse agonist), http://www.guidetopharmacology.org/GRAC/LigandDisplayForward?ligandId=4179 [benzodiazepine site] (Inverse agonist)	http://www.guidetopharmacology.org/GRAC/LigandDisplayForward?ligandId=4192 [benzodiazepine site] (Antagonist) (p*K*_i_ 9.1) [http://www.ncbi.nlm.nih.gov/pubmed/9767648?dopt=AbstractPlus], http://www.guidetopharmacology.org/GRAC/LigandDisplayForward?ligandId=6963 (Positive) (p*K*_i_ 8.8) [http://www.ncbi.nlm.nih.gov/pubmed/2551039?dopt=AbstractPlus], http://www.guidetopharmacology.org/GRAC/LigandDisplayForward?ligandId=4193 [benzodiazepine site] (Positive) (p*K*_i_ 8.3) [http://www.ncbi.nlm.nih.gov/pubmed/8632757?dopt=AbstractPlus], http://www.guidetopharmacology.org/GRAC/LigandDisplayForward?ligandId=7111 [benzodiazepine site] (Positive) (pEC_50_ 7.9) [http://www.ncbi.nlm.nih.gov/pubmed/12408715?dopt=AbstractPlus], http://www.guidetopharmacology.org/GRAC/LigandDisplayForward?ligandId=3364 [benzodiazepine site] (Positive) (p*K*_i_ 7.8) [http://www.ncbi.nlm.nih.gov/pubmed/2551039?dopt=AbstractPlus], http://www.guidetopharmacology.org/GRAC/LigandDisplayForward?ligandId=4094 [benzodiazepine site] (Inverse agonist), http://www.guidetopharmacology.org/GRAC/LigandDisplayForward?ligandId=4095 [benzodiazepine site] (Inverse agonist), http://www.guidetopharmacology.org/GRAC/LigandDisplayForward?ligandId=4179 [benzodiazepine site] (Inverse agonist)
Selective allosteric modulators	http://www.guidetopharmacology.org/GRAC/LigandDisplayForward?ligandId=4348 (Positive) (p*K*_i_ 7.4–7.7) [http://www.ncbi.nlm.nih.gov/pubmed/8719414?dopt=AbstractPlus, http://www.ncbi.nlm.nih.gov/pubmed/8813598?dopt=AbstractPlus], http://www.guidetopharmacology.org/GRAC/LigandDisplayForward?ligandId=4241 [benzodiazepine site] (Antagonist), http://www.guidetopharmacology.org/GRAC/LigandDisplayForward?ligandId=4347 [benzodiazepine site] (Antagonist), http://www.guidetopharmacology.org/GRAC/LigandDisplayForward?ligandId=4221 [benzodiazepine site] (Full agonist), http://www.guidetopharmacology.org/GRAC/LigandDisplayForward?ligandId=4277 [benzodiazepine site] (Full agonist)	http://www.guidetopharmacology.org/GRAC/LigandDisplayForward?ligandId=4241 [benzodiazepine site] (Partial agonist), http://www.guidetopharmacology.org/GRAC/LigandDisplayForward?ligandId=4327 [benzodiazepine site] (Partial agonist)
Labelled ligands	http://www.guidetopharmacology.org/GRAC/LigandDisplayForward?ligandId=4367 [benzodiazepine site] (Allosteric modulator, Antagonist), http://www.guidetopharmacology.org/GRAC/LigandDisplayForward?ligandId=4368 [benzodiazepine site] (Allosteric modulator, Antagonist), http://www.guidetopharmacology.org/GRAC/LigandDisplayForward?ligandId=4369 [anion channel] (Channel blocker), http://www.guidetopharmacology.org/GRAC/LigandDisplayForward?ligandId=4366 [benzodiazepine site] (Allosteric modulator, Mixed), http://www.guidetopharmacology.org/GRAC/LigandDisplayForward?ligandId=4360 [benzodiazepine site] (Allosteric modulator, Positive), http://www.guidetopharmacology.org/GRAC/LigandDisplayForward?ligandId=4083 [GABA site] (Antagonist), http://www.guidetopharmacology.org/GRAC/LigandDisplayForward?ligandId=4090 [GABA site] (Agonist), http://www.guidetopharmacology.org/GRAC/LigandDisplayForward?ligandId=4362 [benzodiazepine site] (Allosteric modulator, Positive)	http://www.guidetopharmacology.org/GRAC/LigandDisplayForward?ligandId=4367 [benzodiazepine site] (Allosteric modulator, Antagonist), http://www.guidetopharmacology.org/GRAC/LigandDisplayForward?ligandId=4368 [benzodiazepine site] (Allosteric modulator, Antagonist), http://www.guidetopharmacology.org/GRAC/LigandDisplayForward?ligandId=4369 [anion channel] (Channel blocker), http://www.guidetopharmacology.org/GRAC/LigandDisplayForward?ligandId=4366 [benzodiazepine site] (Allosteric modulator, Mixed), http://www.guidetopharmacology.org/GRAC/LigandDisplayForward?ligandId=4360 [benzodiazepine site] (Allosteric modulator, Full agonist), http://www.guidetopharmacology.org/GRAC/LigandDisplayForward?ligandId=4083 [GABA site] (Antagonist), http://www.guidetopharmacology.org/GRAC/LigandDisplayForward?ligandId=4090 [GABA site] (Agonist)
Comments	http://www.guidetopharmacology.org/GRAC/LigandDisplayForward?ligandId=566 is an endogenous allosteric regulator and causes potent inhibition of receptors formed from binary combinations of α and β subunits, incorporation of a *δ* or γ subunit causes a modest, or pronounced, reduction in inhibitory potency, respectively [http://www.ncbi.nlm.nih.gov/pubmed/9508826?dopt=AbstractPlus]	http://www.guidetopharmacology.org/GRAC/LigandDisplayForward?ligandId=566 is an endogenous allosteric regulator and causes potent inhibition of receptors formed from binary combinations of α and β subunits, incorporation of a *δ* or γ subunit causes a modest, or pronounced, reduction in inhibitory potency, respectively [http://www.ncbi.nlm.nih.gov/pubmed/9508826?dopt=AbstractPlus]

**Table:** 

Nomenclature	http://www.guidetopharmacology.org/GRAC/ObjectDisplayForward?objectId=406	http://www.guidetopharmacology.org/GRAC/ObjectDisplayForward?objectId=407
HGNC, UniProt	https://www.genenames.org/data/gene‐symbol‐report/#!/hgnc_id/HGNC:4077, http://www.uniprot.org/uniprot/P34903	https://www.genenames.org/data/gene‐symbol‐report/#!/hgnc_id/HGNC:4078, http://www.uniprot.org/uniprot/P48169
Agonists	http://www.guidetopharmacology.org/GRAC/LigandDisplayForward?ligandId=4322 [GABA site], http://www.guidetopharmacology.org/GRAC/LigandDisplayForward?ligandId=4226 [GABA site], http://www.guidetopharmacology.org/GRAC/LigandDisplayForward?ligandId=4227 [GABA site], http://www.guidetopharmacology.org/GRAC/LigandDisplayForward?ligandId=4259 [GABA site], http://www.guidetopharmacology.org/GRAC/LigandDisplayForward?ligandId=4287 [GABA site]	http://www.guidetopharmacology.org/GRAC/LigandDisplayForward?ligandId=4322 [GABA site], http://www.guidetopharmacology.org/GRAC/LigandDisplayForward?ligandId=4226 [GABA site], http://www.guidetopharmacology.org/GRAC/LigandDisplayForward?ligandId=4259 [GABA site], http://www.guidetopharmacology.org/GRAC/LigandDisplayForward?ligandId=4287 [GABA site] (low efficacy)
Selective agonists	–	http://www.guidetopharmacology.org/GRAC/LigandDisplayForward?ligandId=4227 [GABA site] (relatively high efficacy)
Selective antagonists	http://www.guidetopharmacology.org/GRAC/LigandDisplayForward?ligandId=2312 [GABA site], http://www.guidetopharmacology.org/GRAC/LigandDisplayForward?ligandId=4197 [GABA site]	http://www.guidetopharmacology.org/GRAC/LigandDisplayForward?ligandId=2312 [GABA site], http://www.guidetopharmacology.org/GRAC/LigandDisplayForward?ligandId=4197 [GABA site]
Channel blockers	http://www.guidetopharmacology.org/GRAC/LigandDisplayForward?ligandId=4320, http://www.guidetopharmacology.org/GRAC/LigandDisplayForward?ligandId=4051	http://www.guidetopharmacology.org/GRAC/LigandDisplayForward?ligandId=4320, http://www.guidetopharmacology.org/GRAC/LigandDisplayForward?ligandId=4051
Endogenous allosteric modulators	http://www.guidetopharmacology.org/GRAC/LigandDisplayForward?ligandId=4108 (Potentiation), http://www.guidetopharmacology.org/GRAC/LigandDisplayForward?ligandId=566 (Inhibition), http://www.guidetopharmacology.org/GRAC/LigandDisplayForward?ligandId=4321 (Potentiation)	http://www.guidetopharmacology.org/GRAC/LigandDisplayForward?ligandId=4108 (Potentiation), http://www.guidetopharmacology.org/GRAC/LigandDisplayForward?ligandId=566 (Inhibition), http://www.guidetopharmacology.org/GRAC/LigandDisplayForward?ligandId=4321 (Potentiation)
Allosteric modulators	http://www.guidetopharmacology.org/GRAC/LigandDisplayForward?ligandId=4192 [benzodiazepine site] (Antagonist) (p*K*_i_ 9) [http://www.ncbi.nlm.nih.gov/pubmed/9767648?dopt=AbstractPlus], http://www.guidetopharmacology.org/GRAC/LigandDisplayForward?ligandId=6963 (Positive) (p*K*_i_ 8.7) [http://www.ncbi.nlm.nih.gov/pubmed/2551039?dopt=AbstractPlus], http://www.guidetopharmacology.org/GRAC/LigandDisplayForward?ligandId=4193 [benzodiazepine site] (Positive) (p*K*_i_ 7.8) [http://www.ncbi.nlm.nih.gov/pubmed/8632757?dopt=AbstractPlus], http://www.guidetopharmacology.org/GRAC/LigandDisplayForward?ligandId=3364 [benzodiazepine site] (Positive) (p*K*_i_ 7.8) [http://www.ncbi.nlm.nih.gov/pubmed/2551039?dopt=AbstractPlus], http://www.guidetopharmacology.org/GRAC/LigandDisplayForward?ligandId=7111 [benzodiazepine site] (Positive) (pEC_50_ 7.2) [http://www.ncbi.nlm.nih.gov/pubmed/12408715?dopt=AbstractPlus]^,^ http://www.guidetopharmacology.org/GRAC/LigandDisplayForward?ligandId=4095 [benzodiazepine site] (Inverse agonist), http://www.guidetopharmacology.org/GRAC/LigandDisplayForward?ligandId=4179 [benzodiazepine site] (Inverse agonist)	–
Selective allosteric modulators	http://www.guidetopharmacology.org/GRAC/LigandDisplayForward?ligandId=4094 [benzodiazepine site] (higher affinity), http://www.guidetopharmacology.org/GRAC/LigandDisplayForward?ligandId=4241 [benzodiazepine site] (Partial agonist), http://www.guidetopharmacology.org/GRAC/LigandDisplayForward?ligandId=4297 [benzodiazepine site] (Inverse agonist), http://www.guidetopharmacology.org/GRAC/LigandDisplayForward?ligandId=4326 [benzodiazepine site] (Partial agonist), http://www.guidetopharmacology.org/GRAC/LigandDisplayForward?ligandId=4327 [benzodiazepine site] (Partial agonist)	http://www.guidetopharmacology.org/GRAC/LigandDisplayForward?ligandId=4296 [benzodiazepine site] (Full agonist), http://www.guidetopharmacology.org/GRAC/LigandDisplayForward?ligandId=4146 [benzodiazepine site] (Full agonist)
Labelled ligands	http://www.guidetopharmacology.org/GRAC/LigandDisplayForward?ligandId=4367 [benzodiazepine site] (Allosteric modulator, Antagonist), http://www.guidetopharmacology.org/GRAC/LigandDisplayForward?ligandId=4368 [benzodiazepine site] (Allosteric modulator, Antagonist), http://www.guidetopharmacology.org/GRAC/LigandDisplayForward?ligandId=4369 [anion channel] (Channel blocker), http://www.guidetopharmacology.org/GRAC/LigandDisplayForward?ligandId=4366 [benzodiazepine site] (Allosteric modulator, Mixed), http://www.guidetopharmacology.org/GRAC/LigandDisplayForward?ligandId=4360 [benzodiazepine site] (Allosteric modulator, Full agonist), http://www.guidetopharmacology.org/GRAC/LigandDisplayForward?ligandId=4083 [GABA site] (Antagonist), http://www.guidetopharmacology.org/GRAC/LigandDisplayForward?ligandId=4090 [GABA site] (Agonist)	http://www.guidetopharmacology.org/GRAC/LigandDisplayForward?ligandId=4367 [benzodiazepine site] (Allosteric modulator, Partial agonist), http://www.guidetopharmacology.org/GRAC/LigandDisplayForward?ligandId=4368 [benzodiazepine site] (Allosteric modulator, Antagonist), http://www.guidetopharmacology.org/GRAC/LigandDisplayForward?ligandId=4369 [anion channel] (Channel blocker), http://www.guidetopharmacology.org/GRAC/LigandDisplayForward?ligandId=4366 [benzodiazepine site] (Allosteric modulator, Mixed), http://www.guidetopharmacology.org/GRAC/LigandDisplayForward?ligandId=4365 [benzodiazepine site] (Allosteric modulator, Full agonist), http://www.guidetopharmacology.org/GRAC/LigandDisplayForward?ligandId=4083 [GABA site] (Antagonist), http://www.guidetopharmacology.org/GRAC/LigandDisplayForward?ligandId=4090 [GABA site] (Agonist)
Comments	http://www.guidetopharmacology.org/GRAC/LigandDisplayForward?ligandId=566 is an endogenous allosteric regulator and causes potent inhibition of receptors formed from binary combinations of α and β subunits, incorporation of a *δ* or γ subunit causes a modest, or pronounced, reduction in inhibitory potency, respectively [http://www.ncbi.nlm.nih.gov/pubmed/9508826?dopt=AbstractPlus]	http://www.guidetopharmacology.org/GRAC/LigandDisplayForward?ligandId=3364 and http://www.guidetopharmacology.org/GRAC/LigandDisplayForward?ligandId=4193 are not active at this subunit. http://www.guidetopharmacology.org/GRAC/LigandDisplayForward?ligandId=566 is an endogenous allosteric regulator and causes potent inhibition of receptors formed from binary combinations of α and β subunits, incorporation of a *δ* or γ subunit causes a modest, or pronounced, reduction in inhibitory potency, respectively [http://www.ncbi.nlm.nih.gov/pubmed/9508826?dopt=AbstractPlus]. http://www.guidetopharmacology.org/GRAC/LigandDisplayForward?ligandId=4365 selectively labels α4‐subunit‐containing receptors in the presence of a saturating concentration of a ‘classical’ benzodiazepine (*e.g.* http://www.guidetopharmacology.org/GRAC/LigandDisplayForward?ligandId=3364)

**Table:** 

Nomenclature	http://www.guidetopharmacology.org/GRAC/ObjectDisplayForward?objectId=408	http://www.guidetopharmacology.org/GRAC/ObjectDisplayForward?objectId=409
HGNC, UniProt	https://www.genenames.org/data/gene‐symbol‐report/#!/hgnc_id/HGNC:4079, http://www.uniprot.org/uniprot/P31644	https://www.genenames.org/data/gene‐symbol‐report/#!/hgnc_id/HGNC:4080, http://www.uniprot.org/uniprot/Q16445
Agonists	http://www.guidetopharmacology.org/GRAC/LigandDisplayForward?ligandId=4322 [GABA site], http://www.guidetopharmacology.org/GRAC/LigandDisplayForward?ligandId=4226 [GABA site], http://www.guidetopharmacology.org/GRAC/LigandDisplayForward?ligandId=4227 [GABA site], http://www.guidetopharmacology.org/GRAC/LigandDisplayForward?ligandId=4259 [GABA site], http://www.guidetopharmacology.org/GRAC/LigandDisplayForward?ligandId=4287 [GABA site]	http://www.guidetopharmacology.org/GRAC/LigandDisplayForward?ligandId=4322 [GABA site], http://www.guidetopharmacology.org/GRAC/LigandDisplayForward?ligandId=4226 [GABA site], http://www.guidetopharmacology.org/GRAC/LigandDisplayForward?ligandId=4259 [GABA site], http://www.guidetopharmacology.org/GRAC/LigandDisplayForward?ligandId=4287 [GABA site] (low efficacy)
Selective agonists	–	http://www.guidetopharmacology.org/GRAC/LigandDisplayForward?ligandId=4227 [GABA site] (relatively high efficacy)
Selective antagonists	http://www.guidetopharmacology.org/GRAC/LigandDisplayForward?ligandId=2312 [GABA site], http://www.guidetopharmacology.org/GRAC/LigandDisplayForward?ligandId=4197 [GABA site]	http://www.guidetopharmacology.org/GRAC/LigandDisplayForward?ligandId=2312 [GABA site], http://www.guidetopharmacology.org/GRAC/LigandDisplayForward?ligandId=4197 [GABA site]
Channel blockers	http://www.guidetopharmacology.org/GRAC/LigandDisplayForward?ligandId=4320, http://www.guidetopharmacology.org/GRAC/LigandDisplayForward?ligandId=4051	http://www.guidetopharmacology.org/GRAC/LigandDisplayForward?ligandId=4320, http://www.guidetopharmacology.org/GRAC/LigandDisplayForward?ligandId=4051
Endogenous allosteric modulators	http://www.guidetopharmacology.org/GRAC/LigandDisplayForward?ligandId=4108 (Potentiation), http://www.guidetopharmacology.org/GRAC/LigandDisplayForward?ligandId=566 (Inhibition), http://www.guidetopharmacology.org/GRAC/LigandDisplayForward?ligandId=4321 (Potentiation)	http://www.guidetopharmacology.org/GRAC/LigandDisplayForward?ligandId=4108 (Potentiation), http://www.guidetopharmacology.org/GRAC/LigandDisplayForward?ligandId=566 (Inhibition), http://www.guidetopharmacology.org/GRAC/LigandDisplayForward?ligandId=4321 (Potentiation)
Allosteric modulators	http://www.guidetopharmacology.org/GRAC/LigandDisplayForward?ligandId=4192 [benzodiazepine site] (Antagonist) (p*K*_i_ 9.2) [http://www.ncbi.nlm.nih.gov/pubmed/9767648?dopt=AbstractPlus], http://www.guidetopharmacology.org/GRAC/LigandDisplayForward?ligandId=4193 [benzodiazepine site] (Positive) (p*K*_i_ 8.3) [http://www.ncbi.nlm.nih.gov/pubmed/8632757?dopt=AbstractPlus], http://www.guidetopharmacology.org/GRAC/LigandDisplayForward?ligandId=7111 [benzodiazepine site] (Positive) (pEC_50_ 8) [http://www.ncbi.nlm.nih.gov/pubmed/12408715?dopt=AbstractPlus]^,^ http://www.guidetopharmacology.org/GRAC/LigandDisplayForward?ligandId=4094 [benzodiazepine site] (Inverse agonist), http://www.guidetopharmacology.org/GRAC/LigandDisplayForward?ligandId=4179 [benzodiazepine site] (Inverse agonist)	http://www.guidetopharmacology.org/GRAC/LigandDisplayForward?ligandId=4192 [benzodiazepine site] (Partial agonist) (p*K*_i_ 6.8) [http://www.ncbi.nlm.nih.gov/pubmed/9767648?dopt=AbstractPlus], http://www.guidetopharmacology.org/GRAC/LigandDisplayForward?ligandId=4146 [benzodiazepine site] (Full agonist)
Selective allosteric modulators	http://www.guidetopharmacology.org/GRAC/LigandDisplayForward?ligandId=4095 [benzodiazepine site] (Inverse agonist), http://www.guidetopharmacology.org/GRAC/LigandDisplayForward?ligandId=4238 [benzodiazepine site] (Inverse agonist), http://www.guidetopharmacology.org/GRAC/LigandDisplayForward?ligandId=4241 [benzodiazepine site] (Partial agonist), http://www.guidetopharmacology.org/GRAC/LigandDisplayForward?ligandId=4257 [benzodiazepine site] (Inverse agonist), http://www.guidetopharmacology.org/GRAC/LigandDisplayForward?ligandId=4299 [benzodiazepine site] (Inverse agonist), http://www.guidetopharmacology.org/GRAC/LigandDisplayForward?ligandId=4301 [benzodiazepine site] (Inverse agonist)	http://www.guidetopharmacology.org/GRAC/LigandDisplayForward?ligandId=4296 [benzodiazepine site] (Full agonist)
Labelled ligands	http://www.guidetopharmacology.org/GRAC/LigandDisplayForward?ligandId=4364 [benzodiazepine site] (Selective Binding) (p*K*_d_ 9.2) [http://www.ncbi.nlm.nih.gov/pubmed/9353361?dopt=AbstractPlus] – Rat, http://www.guidetopharmacology.org/GRAC/LigandDisplayForward?ligandId=4367 [benzodiazepine site] (Allosteric modulator, Antagonist), http://www.guidetopharmacology.org/GRAC/LigandDisplayForward?ligandId=4368 [benzodiazepine site] (Allosteric modulator, Antagonist), http://www.guidetopharmacology.org/GRAC/LigandDisplayForward?ligandId=4369 [anion channel] (Channel blocker), http://www.guidetopharmacology.org/GRAC/LigandDisplayForward?ligandId=4366 [benzodiazepine site] (Allosteric modulator, Mixed), http://www.guidetopharmacology.org/GRAC/LigandDisplayForward?ligandId=4363 [benzodiazepine site] (Allosteric modulator, Inverse agonist), http://www.guidetopharmacology.org/GRAC/LigandDisplayForward?ligandId=4360 [benzodiazepine site] (Allosteric modulator, Full agonist), http://www.guidetopharmacology.org/GRAC/LigandDisplayForward?ligandId=4083 [GABA site] (Antagonist), http://www.guidetopharmacology.org/GRAC/LigandDisplayForward?ligandId=4090 [GABA site] (Agonist)	http://www.guidetopharmacology.org/GRAC/LigandDisplayForward?ligandId=4367 [benzodiazepine site] (Allosteric modulator, Partial agonist), http://www.guidetopharmacology.org/GRAC/LigandDisplayForward?ligandId=4368 [benzodiazepine site] (Allosteric modulator, Antagonist), http://www.guidetopharmacology.org/GRAC/LigandDisplayForward?ligandId=4369 [anion channel] (Channel blocker), http://www.guidetopharmacology.org/GRAC/LigandDisplayForward?ligandId=4366 [benzodiazepine site] (Allosteric modulator, Mixed), http://www.guidetopharmacology.org/GRAC/LigandDisplayForward?ligandId=4365 [benzodiazepine site] (Allosteric modulator, Full agonist), http://www.guidetopharmacology.org/GRAC/LigandDisplayForward?ligandId=4083 [GABA site] (Antagonist), http://www.guidetopharmacology.org/GRAC/LigandDisplayForward?ligandId=4090 [GABA site] (Agonist)
Comments	http://www.guidetopharmacology.org/GRAC/LigandDisplayForward?ligandId=566 is an endogenous allosteric regulator and causes potent inhibition of receptors formed from binary combinations of α and β subunits, incorporation of a *δ* or γ subunit causes a modest, or pronounced, reduction in inhibitory potency, respectively [http://www.ncbi.nlm.nih.gov/pubmed/9508826?dopt=AbstractPlus]	http://www.guidetopharmacology.org/GRAC/LigandDisplayForward?ligandId=3364 and http://www.guidetopharmacology.org/GRAC/LigandDisplayForward?ligandId=4193 are not active at this subunit. http://www.guidetopharmacology.org/GRAC/LigandDisplayForward?ligandId=566 is an endogenous allosteric regulator and causes potent inhibition of receptors formed from binary combinations of α and β subunits, incorporation of a *δ* or γ subunit causes a modest, or pronounced, reduction in inhibitory potency, respectively [http://www.ncbi.nlm.nih.gov/pubmed/9508826?dopt=AbstractPlus]. http://www.guidetopharmacology.org/GRAC/LigandDisplayForward?ligandId=4365 selectively labels α6‐subunit‐containing receptors in the presence of a saturating concentration of a ‘classical’ benzodiazepine (*e.g.* http://www.guidetopharmacology.org/GRAC/LigandDisplayForward?ligandId=3364)

**Table:** 

Nomenclature	http://www.guidetopharmacology.org/GRAC/ObjectDisplayForward?objectId=410	http://www.guidetopharmacology.org/GRAC/ObjectDisplayForward?objectId=411	http://www.guidetopharmacology.org/GRAC/ObjectDisplayForward?objectId=412
HGNC, UniProt	https://www.genenames.org/data/gene‐symbol‐report/#!/hgnc_id/HGNC:4081, http://www.uniprot.org/uniprot/P18505	https://www.genenames.org/data/gene‐symbol‐report/#!/hgnc_id/HGNC:4082, http://www.uniprot.org/uniprot/P47870	https://www.genenames.org/data/gene‐symbol‐report/#!/hgnc_id/HGNC:4083, http://www.uniprot.org/uniprot/P28472
Channel blockers	http://www.guidetopharmacology.org/GRAC/LigandDisplayForward?ligandId=4320, http://www.guidetopharmacology.org/GRAC/LigandDisplayForward?ligandId=4051	http://www.guidetopharmacology.org/GRAC/LigandDisplayForward?ligandId=4320, http://www.guidetopharmacology.org/GRAC/LigandDisplayForward?ligandId=4051	http://www.guidetopharmacology.org/GRAC/LigandDisplayForward?ligandId=4320, http://www.guidetopharmacology.org/GRAC/LigandDisplayForward?ligandId=4051
Allosteric modulators	–	–	http://www.guidetopharmacology.org/GRAC/LigandDisplayForward?ligandId=7336 (Binding) (pIC_50_ 5.5) [http://www.ncbi.nlm.nih.gov/pubmed/8773466?dopt=AbstractPlus]
Comments	http://www.guidetopharmacology.org/GRAC/LigandDisplayForward?ligandId=566 is an endogenous allosteric regulator and causes potent inhibition of receptors formed from binary combinations of α and β subunits, incorporation of a *δ* or γ subunit causes a modest, or pronounced, reduction in inhibitory potency, respectively [http://www.ncbi.nlm.nih.gov/pubmed/9508826?dopt=AbstractPlus]		

**Table:** 

Nomenclature	http://www.guidetopharmacology.org/GRAC/ObjectDisplayForward?objectId=413	http://www.guidetopharmacology.org/GRAC/ObjectDisplayForward?objectId=414	http://www.guidetopharmacology.org/GRAC/ObjectDisplayForward?objectId=415
HGNC, UniProt	https://www.genenames.org/data/gene‐symbol‐report/#!/hgnc_id/HGNC:4086, http://www.uniprot.org/uniprot/Q8N1C3	https://www.genenames.org/data/gene‐symbol‐report/#!/hgnc_id/HGNC:4087, http://www.uniprot.org/uniprot/P18507	https://www.genenames.org/data/gene‐symbol‐report/#!/hgnc_id/HGNC:4088, http://www.uniprot.org/uniprot/Q99928
Channel blockers	http://www.guidetopharmacology.org/GRAC/LigandDisplayForward?ligandId=4320, http://www.guidetopharmacology.org/GRAC/LigandDisplayForward?ligandId=4051	http://www.guidetopharmacology.org/GRAC/LigandDisplayForward?ligandId=4320, http://www.guidetopharmacology.org/GRAC/LigandDisplayForward?ligandId=4051	http://www.guidetopharmacology.org/GRAC/LigandDisplayForward?ligandId=4320, http://www.guidetopharmacology.org/GRAC/LigandDisplayForward?ligandId=4051
Comments	http://www.guidetopharmacology.org/GRAC/LigandDisplayForward?ligandId=566 is an endogenous allosteric regulator and causes potent inhibition of receptors formed from binary combinations of α and β subunits, incorporation of a *δ* or γ subunit causes a modest, or pronounced, reduction in inhibitory potency, respectively [http://www.ncbi.nlm.nih.gov/pubmed/9508826?dopt=AbstractPlus]		

**Table:** 

Nomenclature	http://www.guidetopharmacology.org/GRAC/ObjectDisplayForward?objectId=416	http://www.guidetopharmacology.org/GRAC/ObjectDisplayForward?objectId=417	http://www.guidetopharmacology.org/GRAC/ObjectDisplayForward?objectId=418	http://www.guidetopharmacology.org/GRAC/ObjectDisplayForward?objectId=419
HGNC, UniProt	https://www.genenames.org/data/gene‐symbol‐report/#!/hgnc_id/HGNC:4084, http://www.uniprot.org/uniprot/O14764	https://www.genenames.org/data/gene‐symbol‐report/#!/hgnc_id/HGNC:4085, http://www.uniprot.org/uniprot/P78334	https://www.genenames.org/data/gene‐symbol‐report/#!/hgnc_id/HGNC:14454, http://www.uniprot.org/uniprot/Q9UN88	https://www.genenames.org/data/gene‐symbol‐report/#!/hgnc_id/HGNC:4089, http://www.uniprot.org/uniprot/O00591
Selective agonists	http://www.guidetopharmacology.org/GRAC/LigandDisplayForward?ligandId=4322 [GABA site]	–	–	–
Channel blockers	http://www.guidetopharmacology.org/GRAC/LigandDisplayForward?ligandId=4320, http://www.guidetopharmacology.org/GRAC/LigandDisplayForward?ligandId=4051	http://www.guidetopharmacology.org/GRAC/LigandDisplayForward?ligandId=4320, http://www.guidetopharmacology.org/GRAC/LigandDisplayForward?ligandId=4051	http://www.guidetopharmacology.org/GRAC/LigandDisplayForward?ligandId=4320, http://www.guidetopharmacology.org/GRAC/LigandDisplayForward?ligandId=4051	http://www.guidetopharmacology.org/GRAC/LigandDisplayForward?ligandId=4320, http://www.guidetopharmacology.org/GRAC/LigandDisplayForward?ligandId=4051
Comments	http://www.guidetopharmacology.org/GRAC/LigandDisplayForward?ligandId=566 is an endogenous allosteric regulator and causes potent inhibition of receptors formed from binary combinations of α and β subunits, incorporation of a *δ* or γ subunit causes a modest, or pronounced, reduction in inhibitory potency, respectively			

**Table:** 

Nomenclature	http://www.guidetopharmacology.org/GRAC/ObjectDisplayForward?objectId=420	http://www.guidetopharmacology.org/GRAC/ObjectDisplayForward?objectId=421	http://www.guidetopharmacology.org/GRAC/ObjectDisplayForward?objectId=422
HGNC, UniProt	https://www.genenames.org/data/gene‐symbol‐report/#!/hgnc_id/HGNC:4090, http://www.uniprot.org/uniprot/P24046	https://www.genenames.org/data/gene‐symbol‐report/#!/hgnc_id/HGNC:4091, http://www.uniprot.org/uniprot/P28476	https://www.genenames.org/data/gene‐symbol‐report/#!/hgnc_id/HGNC:17969, http://www.uniprot.org/uniprot/A8MPY1
Agonists	http://www.guidetopharmacology.org/GRAC/LigandDisplayForward?ligandId=4226 [GABA site] (Partial agonist), http://www.guidetopharmacology.org/GRAC/LigandDisplayForward?ligandId=4259 [GABA site] (Partial agonist)	http://www.guidetopharmacology.org/GRAC/LigandDisplayForward?ligandId=4226 [GABA site] (Partial agonist), http://www.guidetopharmacology.org/GRAC/LigandDisplayForward?ligandId=4259 [GABA site] (Partial agonist)	http://www.guidetopharmacology.org/GRAC/LigandDisplayForward?ligandId=4226 [GABA site] (Partial agonist), http://www.guidetopharmacology.org/GRAC/LigandDisplayForward?ligandId=4259 [GABA site] (Partial agonist)
Selective agonists	http://www.guidetopharmacology.org/GRAC/LigandDisplayForward?ligandId=4067 [GABA site], http://www.guidetopharmacology.org/GRAC/LigandDisplayForward?ligandId=4107 [GABA site]	http://www.guidetopharmacology.org/GRAC/LigandDisplayForward?ligandId=4067 [GABA site], http://www.guidetopharmacology.org/GRAC/LigandDisplayForward?ligandId=4107 [GABA site]	http://www.guidetopharmacology.org/GRAC/LigandDisplayForward?ligandId=4067 [GABA site], http://www.guidetopharmacology.org/GRAC/LigandDisplayForward?ligandId=4107 [GABA site]
Antagonists	http://www.guidetopharmacology.org/GRAC/LigandDisplayForward?ligandId=4322 [GABA site], http://www.guidetopharmacology.org/GRAC/LigandDisplayForward?ligandId=4227 [GABA site], http://www.guidetopharmacology.org/GRAC/LigandDisplayForward?ligandId=4287 [GABA site]	http://www.guidetopharmacology.org/GRAC/LigandDisplayForward?ligandId=4322 [GABA site], http://www.guidetopharmacology.org/GRAC/LigandDisplayForward?ligandId=4227 [GABA site], http://www.guidetopharmacology.org/GRAC/LigandDisplayForward?ligandId=4287 [GABA site]	http://www.guidetopharmacology.org/GRAC/LigandDisplayForward?ligandId=4322 [GABA site], http://www.guidetopharmacology.org/GRAC/LigandDisplayForward?ligandId=4227 [GABA site], http://www.guidetopharmacology.org/GRAC/LigandDisplayForward?ligandId=4287 [GABA site]
Selective antagonists	http://www.guidetopharmacology.org/GRAC/LigandDisplayForward?ligandId=4096 [GABA site], http://www.guidetopharmacology.org/GRAC/LigandDisplayForward?ligandId=4099 [GABA site], http://www.guidetopharmacology.org/GRAC/LigandDisplayForward?ligandId=4328 [GABA site], http://www.guidetopharmacology.org/GRAC/LigandDisplayForward?ligandId=4143 [GABA site]	http://www.guidetopharmacology.org/GRAC/LigandDisplayForward?ligandId=4096 [GABA site], http://www.guidetopharmacology.org/GRAC/LigandDisplayForward?ligandId=4099 [GABA site], http://www.guidetopharmacology.org/GRAC/LigandDisplayForward?ligandId=4328 [GABA site], http://www.guidetopharmacology.org/GRAC/LigandDisplayForward?ligandId=4143 [GABA site]	http://www.guidetopharmacology.org/GRAC/LigandDisplayForward?ligandId=4096 [GABA site], http://www.guidetopharmacology.org/GRAC/LigandDisplayForward?ligandId=4099 [GABA site], http://www.guidetopharmacology.org/GRAC/LigandDisplayForward?ligandId=4328 [GABA site], http://www.guidetopharmacology.org/GRAC/LigandDisplayForward?ligandId=4143 [GABA site]
Channel blockers	http://www.guidetopharmacology.org/GRAC/LigandDisplayForward?ligandId=4320, http://www.guidetopharmacology.org/GRAC/LigandDisplayForward?ligandId=4051	http://www.guidetopharmacology.org/GRAC/LigandDisplayForward?ligandId=4320, http://www.guidetopharmacology.org/GRAC/LigandDisplayForward?ligandId=4051	http://www.guidetopharmacology.org/GRAC/LigandDisplayForward?ligandId=4320, http://www.guidetopharmacology.org/GRAC/LigandDisplayForward?ligandId=4051
Comments	http://www.guidetopharmacology.org/GRAC/LigandDisplayForward?ligandId=2312 is not active at this subunit	http://www.guidetopharmacology.org/GRAC/LigandDisplayForward?ligandId=2312 is not active at this subunit	http://www.guidetopharmacology.org/GRAC/LigandDisplayForward?ligandId=2312 is not active at this subunit

### Comments

The potency and efficacy of many GABA agonists vary between GABA_A_ receptor isoforms [http://www.ncbi.nlm.nih.gov/pubmed/12171573?dopt=AbstractPlus, http://www.ncbi.nlm.nih.gov/pubmed/23385381?dopt=AbstractPlus, http://www.ncbi.nlm.nih.gov/pubmed/12469353?dopt=AbstractPlus]. For example, http://www.guidetopharmacology.org/GRAC/LigandDisplayForward?ligandId=4322 is a partial agonist at receptors with the subunit composition α4β3γ2, but elicits currents in excess of those evoked by GABA at the α4β3*δ* receptor where GABA itself is a low efficacy agonist [http://www.ncbi.nlm.nih.gov/pubmed/14645489?dopt=AbstractPlus, http://www.ncbi.nlm.nih.gov/pubmed/12145096?dopt=AbstractPlus]. The antagonists http://www.guidetopharmacology.org/GRAC/LigandDisplayForward?ligandId=2312 and http://www.guidetopharmacology.org/GRAC/LigandDisplayForward?ligandId=4197 differ in their ability to suppress spontaneous openings of the GABA_A_ receptor, the former being more effective [http://www.ncbi.nlm.nih.gov/pubmed/10455284?dopt=AbstractPlus]. The presence of the γ subunit within the heterotrimeric complex reduces the potency and efficacy of agonists [http://www.ncbi.nlm.nih.gov/pubmed/16272218?dopt=AbstractPlus]. The GABA_A_ receptor contains distinct allosteric sites that bind barbiturates and endogenous (*e.g.*, http://www.guidetopharmacology.org/GRAC/LigandDisplayForward?ligandId=4108‐http://www.guidetopharmacology.org/GRAC/LigandDisplayForward?ligandId=4108) and synthetic (*e.g.*, http://www.guidetopharmacology.org/GRAC/LigandDisplayForward?ligandId=5461) neuroactive steroids in a diastereo‐ or enantio‐selective manner [http://www.ncbi.nlm.nih.gov/pubmed/15959466?dopt=AbstractPlus, http://www.ncbi.nlm.nih.gov/pubmed/17531325?dopt=AbstractPlus, http://www.ncbi.nlm.nih.gov/pubmed/17560657?dopt=AbstractPlus, http://www.ncbi.nlm.nih.gov/pubmed/19199916?dopt=AbstractPlus]. Picrotoxinin and TBPS act at an allosteric site within the chloride channel pore to negatively regulate channel activity; negative allosteric regulation by http://www.guidetopharmacology.org/GRAC/LigandDisplayForward?ligandId=5462 derivatives also involves the http://www.guidetopharmacology.org/GRAC/LigandDisplayForward?ligandId=2291 site, whereas positive allosteric regulation by such compounds is proposed to occur at a distinct locus. Many intravenous (*e.g.*, http://www.guidetopharmacology.org/GRAC/LigandDisplayForward?ligandId=5463, http://www.guidetopharmacology.org/GRAC/LigandDisplayForward?ligandId=5464) and inhalational (*e.g*., http://www.guidetopharmacology.org/GRAC/LigandDisplayForward?ligandId=2401, http://www.guidetopharmacology.org/GRAC/LigandDisplayForward?ligandId=2505) anaesthetics and alcohols also exert a regulatory influence upon GABA_A_ receptor activity [http://www.ncbi.nlm.nih.gov/pubmed/18201756?dopt=AbstractPlus, http://www.ncbi.nlm.nih.gov/pubmed/21194017?dopt=AbstractPlus]. Specific amino acid residues within GABA_A_ receptor α‐ and β‐subunits that influence allosteric regulation by anaesthetic and non‐anaesthetic compounds havebeen identified [http://www.ncbi.nlm.nih.gov/pubmed/16126282?dopt=AbstractPlus, http://www.ncbi.nlm.nih.gov/pubmed/17560657?dopt=AbstractPlus]. Photoaffinity labelling of distinct amino acid residues within purified GABA_A_ receptors by the etomidate derivative, http://www.guidetopharmacology.org/GRAC/LigandDisplayForward?ligandId=5404, has also been demonstrated [http://www.ncbi.nlm.nih.gov/pubmed/17093081?dopt=AbstractPlus] and this binding subject to positive allosteric regulation by anaesthetic steroids [http://www.ncbi.nlm.nih.gov/pubmed/19282280?dopt=AbstractPlus]. An array of natural products including flavonoid and terpenoid compounds exert varied actions at GABA_A_ receptors (reviewed in detail in [http://www.ncbi.nlm.nih.gov/pubmed/15974965?dopt=AbstractPlus]).

In addition to the agents listed in the table, modulators of GABA_A_ receptor activity that exhibit subunit dependent activity include: http://www.guidetopharmacology.org/GRAC/LigandDisplayForward?ligandId=5465 [negative allosteric modulator selective for β1‐ versus β2‐, or β3‐subunit‐containing receptors [http://www.ncbi.nlm.nih.gov/pubmed/15100159?dopt=AbstractPlus]]; fragrent dioxane derivatives [positive allosteric modulators selective for β1‐ versus β2‐, or β3‐subunit‐containing receptors [http://www.ncbi.nlm.nih.gov/pubmed/20511229?dopt=AbstractPlus]]; http://www.guidetopharmacology.org/GRAC/LigandDisplayForward?ligandId=5466, http://www.guidetopharmacology.org/GRAC/LigandDisplayForward?ligandId=5463, http://www.guidetopharmacology.org/GRAC/LigandDisplayForward?ligandId=5467, http://www.guidetopharmacology.org/GRAC/LigandDisplayForward?ligandId=2593, http://www.guidetopharmacology.org/GRAC/LigandDisplayForward?ligandId=5468, http://www.guidetopharmacology.org/GRAC/LigandDisplayForward?ligandId=5469, http://www.guidetopharmacology.org/GRAC/LigandDisplayForward?ligandId=5470 [positive allosteric modulators with selectivity for β2/β3‐ over β1‐subunit‐containing receptors [http://www.ncbi.nlm.nih.gov/pubmed/18585399?dopt=AbstractPlus, http://www.ncbi.nlm.nih.gov/pubmed/20718740?dopt=AbstractPlus, http://www.ncbi.nlm.nih.gov/pubmed/12126658?dopt=AbstractPlus]]; http://www.guidetopharmacology.org/GRAC/LigandDisplayForward?ligandId=5467 [intrinsic efficacy, *i.e*., potentiation, or inhibition, is dependent upon the identity of the γ1‐3‐, *δ*‐, or *∈*‐subunit co‐assembed with α1‐ and β1‐subunits [http://www.ncbi.nlm.nih.gov/pubmed/12367611?dopt=AbstractPlus]]; http://www.guidetopharmacology.org/GRAC/LigandDisplayForward?ligandId=2421 [selective blockade of receptors containing an α6‐subunit [http://www.ncbi.nlm.nih.gov/pubmed/12021393?dopt=AbstractPlus]]; http://www.guidetopharmacology.org/GRAC/LigandDisplayForward?ligandId=4839 [selective blockade of receptors containing an α6subunit co‐assembled with β2/β3‐, but not β1‐subunit [http://www.ncbi.nlm.nih.gov/pubmed/12126658?dopt=AbstractPlus]]; http://www.guidetopharmacology.org/GRAC/LigandDisplayForward?ligandId=2434 [potentiates responses mediated by α1β3γ2L receptors, weakly inhibits α6β3γ2L receptors, and strongly blocks α6β3*δ* and α4β3*δ* receptors [http://www.ncbi.nlm.nih.gov/pubmed/12145096?dopt=AbstractPlus, http://www.ncbi.nlm.nih.gov/pubmed/9203639?dopt=AbstractPlus]]; http://www.guidetopharmacology.org/GRAC/LigandDisplayForward?ligandId=2299 [selectively potentiates responses mediated by α4β3*δ* and α6β3*δ* receptors versus receptors in which β2 replaces β3, or γ replaces *δ* [http://www.ncbi.nlm.nih.gov/pubmed/16814864?dopt=AbstractPlus], but see also [http://www.ncbi.nlm.nih.gov/pubmed/17591542?dopt=AbstractPlus]]; http://www.guidetopharmacology.org/GRAC/LigandDisplayForward?ligandId=4183 and http://www.guidetopharmacology.org/GRAC/LigandDisplayForward?ligandId=4184 [selectively potentiate responses mediated by *δ*‐subunitcontaining receptors [http://www.ncbi.nlm.nih.gov/pubmed/18762200?dopt=AbstractPlus]]. It should be noted that the apparent selectivity of some positive allosteric modulators (*e.g*., neurosteroids such as http://www.guidetopharmacology.org/GRAC/LigandDisplayForward?ligandId=4108 for *δ*‐subunit‐containing receptors (*e.g.*, α1β3*δ*) may be a consequence of the unusually low efficacy of GABA at this receptor isoform [http://www.ncbi.nlm.nih.gov/pubmed/19828786?dopt=AbstractPlus, http://www.ncbi.nlm.nih.gov/pubmed/14645489?dopt=AbstractPlus].

### Further reading on GABA_A_ receptors

Atack JR. (2008) GABA(A) receptor subtype‐selective efficacy: TPA023, an alpha2/alpha3 selective non‐sedating anxiolytic and alpha5IA, an alpha5 selective cognition enhancer. *CNS Neurosci Ther* **14**: 25‐35 https://www.ncbi.nlm.nih.gov/pubmed/18482097?dopt=AbstractPlus

Braat S *et al.* (2015) The GABA_A_ Receptor as a Therapeutic Target for Neurodevelopmental Disorders. *Neuron* **86**: 1119‐30 https://www.ncbi.nlm.nih.gov/pubmed/26050032?dopt=AbstractPlus

Calvo DJ *et al.* (2016) Dynamic Regulation of the GABA_A_ Receptor Function by Redox Mechanisms. *Mol. Pharmacol.* **90**: 326‐33 https://www.ncbi.nlm.nih.gov/pubmed/27439531?dopt=AbstractPlus

Masiulis S *et al.* (2019) GABA_A receptor signalling mechanisms revealed by structural pharmacology. *Nature* **565**: 454‐459 https://www.ncbi.nlm.nih.gov/pubmed/30602790?dopt=AbstractPlus

Mele M *et al.* (2016) Role of GABA_A R trafficking in the plasticity of inhibitory synapses. *J. Neurochem.* **139**: 997‐1018 https://www.ncbi.nlm.nih.gov/pubmed/27424566?dopt=AbstractPlus

## http://www.guidetopharmacology.org/GRAC/FamilyDisplayForward?familyId=73

### Overview

The inhibitory glycine receptor (**nomenclature as agreed by the NC‐IUPHAR Subcommittee on Glycine Receptors**) is a member of the Cys‐loop superfamily of transmittergated ion channels that includes the zinc activated channels, GABA_A_, nicotinic acetylcholine and 5‐HT_3_ receptors [http://www.ncbi.nlm.nih.gov/pubmed/18721822?dopt=AbstractPlus]. The receptor is expressed either as a homo‐pentamer of α subunits, or a complex now thought to harbour 2α and 3β subunits [http://www.ncbi.nlm.nih.gov/pubmed/16805771?dopt=AbstractPlus, http://www.ncbi.nlm.nih.gov/pubmed/15748848?dopt=AbstractPlus], that contain an intrinsic anion channel. Four differentially expressed isoforms of the α‐subunit (α1‐α4) and one variant of the β‐subunit (β1, https://www.genenames.org/data/gene‐symbol‐report/#!/hgnc_id/HGNC:4329, http://www.uniprot.org/uniprot/P48167) have been identified by genomic and cDNA cloning. Further diversity originates from alternative splicing of the primary gene transcripts for α1 (α1^INS^ and α1^del^), α2 (α2A and α2B), α3 (α3S and α3L) and β (βΔ7) subunits and by mRNA editing of the α2 and α3 subunit [http://www.ncbi.nlm.nih.gov/pubmed/19210758?dopt=AbstractPlus, http://www.ncbi.nlm.nih.gov/pubmed/15895087?dopt=AbstractPlus, http://www.ncbi.nlm.nih.gov/pubmed/17145751?dopt=AbstractPlus]. Both α2 splicing and α3 mRNA editing can produce subunits (*i.e.*, α2B and α3P185L) with enhanced agonist sensitivity. Predominantly, the mature form of the receptor contains α1 (or α3) and β subunits while the immature form is mostly composed of only α2 subunits. RNA transcripts encoding the α4‐subunit have not been detected in adult humans. The N‐terminal domain of the α‐subunit contains both the agonist and http://www.guidetopharmacology.org/GRAC/LigandDisplayForward?ligandId=347 binding sites that consist of several discontinuous regions of amino acids. Inclusion of the β‐subunit in the pentameric glycine receptor contributes to agonist binding, reduces single channel conductance and alters pharmacology. The β‐subunit also anchors the receptor, via an amphipathic sequence within the large intracellular loop region, to gephyrin. The latter is a cytoskeletal attachment protein that binds to a number of subsynaptic proteins involved in cytoskeletal structure and thus clusters and anchors hetero‐oligomeric receptors to the synapse [http://www.ncbi.nlm.nih.gov/pubmed/16807723?dopt=AbstractPlus, http://www.ncbi.nlm.nih.gov/pubmed/17504238?dopt=AbstractPlus, http://www.ncbi.nlm.nih.gov/pubmed/11283747?dopt=AbstractPlus]. G‐protein βγ subunits enhance the open state probability of native and recombinant glycine receptors by association with domains within the large intracellular loop [http://www.ncbi.nlm.nih.gov/pubmed/17040914?dopt=AbstractPlus, http://www.ncbi.nlm.nih.gov/pubmed/12858180?dopt=AbstractPlus]. Intracellular chloride concentration modulates the kinetics of native and recombinant glycine receptors [http://www.ncbi.nlm.nih.gov/pubmed/18987182?dopt=AbstractPlus]. Intracellular Ca^2+^ appears to increase native and recombinant glycine receptor affinity, prolonging channel open events, by a mechanism that does not involve phosphorylation [http://www.ncbi.nlm.nih.gov/pubmed/11144365?dopt=AbstractPlus].

**Table:** 

Nomenclature	http://www.guidetopharmacology.org/GRAC/ObjectDisplayForward?objectId=423	http://www.guidetopharmacology.org/GRAC/ObjectDisplayForward?objectId=424	http://www.guidetopharmacology.org/GRAC/ObjectDisplayForward?objectId=425	http://www.guidetopharmacology.org/GRAC/ObjectDisplayForward?objectId=426
HGNC, UniProt	https://www.genenames.org/data/gene‐symbol‐report/#!/hgnc_id/HGNC:4326, http://www.uniprot.org/uniprot/P23415	https://www.genenames.org/data/gene‐symbol‐report/#!/hgnc_id/HGNC:4327, http://www.uniprot.org/uniprot/P23416	https://www.genenames.org/data/gene‐symbol‐report/#!/hgnc_id/HGNC:4328, http://www.uniprot.org/uniprot/O75311	https://www.genenames.org/data/gene‐symbol‐report/#!/hgnc_id/HGNC:31715, http://www.uniprot.org/uniprot/Q5JXX5
Selective agonists (potency order)	http://www.guidetopharmacology.org/GRAC/LigandDisplayForward?ligandId=727 > http://www.guidetopharmacology.org/GRAC/LigandDisplayForward?ligandId=2365 > http://www.guidetopharmacology.org/GRAC/LigandDisplayForward?ligandId=2379	http://www.guidetopharmacology.org/GRAC/LigandDisplayForward?ligandId=727 > http://www.guidetopharmacology.org/GRAC/LigandDisplayForward?ligandId=2365 > http://www.guidetopharmacology.org/GRAC/LigandDisplayForward?ligandId=2379	http://www.guidetopharmacology.org/GRAC/LigandDisplayForward?ligandId=727 > http://www.guidetopharmacology.org/GRAC/LigandDisplayForward?ligandId=2365 > http://www.guidetopharmacology.org/GRAC/LigandDisplayForward?ligandId=2379	–
Selective antagonists	http://www.guidetopharmacology.org/GRAC/LigandDisplayForward?ligandId=4201 (pIC_50_ 6.1), http://www.guidetopharmacology.org/GRAC/LigandDisplayForward?ligandId=4290 (p*K*_i_ 5.7), http://www.guidetopharmacology.org/GRAC/LigandDisplayForward?ligandId=2514 (pIC_50_ 5.5), http://www.guidetopharmacology.org/GRAC/LigandDisplayForward?ligandId=2366 (pIC_50_ 4.7), http://www.guidetopharmacology.org/GRAC/LigandDisplayForward?ligandId=260 (p*K*_i_ 4.1), http://www.guidetopharmacology.org/GRAC/LigandDisplayForward?ligandId=2367 (pIC_50_ 3.5), http://www.guidetopharmacology.org/GRAC/LigandDisplayForward?ligandId=746 (weak inhibition), http://www.guidetopharmacology.org/GRAC/LigandDisplayForward?ligandId=4289, http://www.guidetopharmacology.org/GRAC/LigandDisplayForward?ligandId=347	http://www.guidetopharmacology.org/GRAC/LigandDisplayForward?ligandId=731 (pIC_50_ 7), http://www.guidetopharmacology.org/GRAC/LigandDisplayForward?ligandId=733 (pIC_50_ 6.7), http://www.guidetopharmacology.org/GRAC/LigandDisplayForward?ligandId=746 (pIC_50_ 6), http://www.guidetopharmacology.org/GRAC/LigandDisplayForward?ligandId=4201 (pIC_50_ 5.6), http://www.guidetopharmacology.org/GRAC/LigandDisplayForward?ligandId=4290 (p*K*_i_ 5.3), http://www.guidetopharmacology.org/GRAC/LigandDisplayForward?ligandId=2366 (pIC_50_ 5.1), http://www.guidetopharmacology.org/GRAC/LigandDisplayForward?ligandId=260 (p*K*_i_ 4.9), http://www.guidetopharmacology.org/GRAC/LigandDisplayForward?ligandId=2367 (pIC_50_ 4.2), http://www.guidetopharmacology.org/GRAC/LigandDisplayForward?ligandId=2361 (pIC_50_ 3.7), http://www.guidetopharmacology.org/GRAC/LigandDisplayForward?ligandId=4289, http://www.guidetopharmacology.org/GRAC/LigandDisplayForward?ligandId=347	http://www.guidetopharmacology.org/GRAC/LigandDisplayForward?ligandId=731 (pIC_50_ 7.3), http://www.guidetopharmacology.org/GRAC/LigandDisplayForward?ligandId=746 (pIC_50_ 7), http://www.guidetopharmacology.org/GRAC/LigandDisplayForward?ligandId=733 (pIC_50_ 7), http://www.guidetopharmacology.org/GRAC/LigandDisplayForward?ligandId=4072 (pIC_50_ 5.2), http://www.guidetopharmacology.org/GRAC/LigandDisplayForward?ligandId=2514 (pIC_50_ 4.5), http://www.guidetopharmacology.org/GRAC/LigandDisplayForward?ligandId=347	–
Channel blockers	http://www.guidetopharmacology.org/GRAC/LigandDisplayForward?ligandId=1862 (pIC_50_ 5.1–6.2), http://www.guidetopharmacology.org/GRAC/LigandDisplayForward?ligandId=2368 (pIC_50_ 5.9) [http://www.ncbi.nlm.nih.gov/pubmed/8090751?dopt=AbstractPlus], http://www.guidetopharmacology.org/GRAC/LigandDisplayForward?ligandId=4286 (pIC_50_ 5.3), http://www.guidetopharmacology.org/GRAC/LigandDisplayForward?ligandId=2291 (pIC_50_ 5.3), http://www.guidetopharmacology.org/GRAC/LigandDisplayForward?ligandId=4051 (pIC_50_ 5.2)	http://www.guidetopharmacology.org/GRAC/LigandDisplayForward?ligandId=2291 (pIC_50_ 6.4), http://www.guidetopharmacology.org/GRAC/LigandDisplayForward?ligandId=4051 (pIC_50_ 5.6), http://www.guidetopharmacology.org/GRAC/LigandDisplayForward?ligandId=1862 (pIC_50_ 4.9–5.4), http://www.guidetopharmacology.org/GRAC/LigandDisplayForward?ligandId=4286 (pIC_50_ 4.9), http://www.guidetopharmacology.org/GRAC/LigandDisplayForward?ligandId=2368 (pIC_50_ >4.7) [http://www.ncbi.nlm.nih.gov/pubmed/8090751?dopt=AbstractPlus]	http://www.guidetopharmacology.org/GRAC/LigandDisplayForward?ligandId=2291 (pIC_50_ 6.4), http://www.guidetopharmacology.org/GRAC/LigandDisplayForward?ligandId=1862 (pIC_50_ 5.7), http://www.guidetopharmacology.org/GRAC/LigandDisplayForward?ligandId=4286 (pIC_50_ 5.2), http://www.guidetopharmacology.org/GRAC/LigandDisplayForward?ligandId=4051 (block is weaker when β subunit is co‐expressed)	–
Endogenous allosteric modulators	http://www.guidetopharmacology.org/GRAC/LigandDisplayForward?ligandId=566 (Potentiation) (pEC_50_ 7.4), http://www.guidetopharmacology.org/GRAC/LigandDisplayForward?ligandId=4164 (Inhibition) (pIC_50_ 4.8–5.4), http://www.guidetopharmacology.org/GRAC/LigandDisplayForward?ligandId=566 (Inhibition) (pIC_50_ 4.8), Extracellular http://www.guidetopharmacology.org/GRAC/LigandDisplayForward?ligandId=2346 (Inhibition)	http://www.guidetopharmacology.org/GRAC/LigandDisplayForward?ligandId=566 (Potentiation) (pEC_50_ 6.3), http://www.guidetopharmacology.org/GRAC/LigandDisplayForward?ligandId=4164 (Inhibition) (pIC_50_ 4.8), http://www.guidetopharmacology.org/GRAC/LigandDisplayForward?ligandId=566 (Inhibition) (pIC_50_ 3.4)	http://www.guidetopharmacology.org/GRAC/LigandDisplayForward?ligandId=4164 (Inhibition) (pIC_50_ 5), http://www.guidetopharmacology.org/GRAC/LigandDisplayForward?ligandId=566 (Inhibition) (pIC_50_ 3.8)	–
Selective allosteric modulators	http://www.guidetopharmacology.org/GRAC/LigandDisplayForward?ligandId=2364 (Potentiation) (pEC_50_ 7.4), http://www.guidetopharmacology.org/GRAC/LigandDisplayForward?ligandId=731 (Potentiation) (pEC_50_ 6.6), http://www.guidetopharmacology.org/GRAC/LigandDisplayForward?ligandId=2424 (Potentiation) (pEC_50_ ∼5.5)	http://www.guidetopharmacology.org/GRAC/LigandDisplayForward?ligandId=2424 (Potentiation) (pEC_50_ ∼6)	http://www.guidetopharmacology.org/GRAC/LigandDisplayForward?ligandId=2424 (Potentiation) (pEC_50_ ∼5.3)	–
Labelled ligands	http://www.guidetopharmacology.org/GRAC/LigandDisplayForward?ligandId=2360 (Antagonist)	http://www.guidetopharmacology.org/GRAC/LigandDisplayForward?ligandId=2360 (Antagonist)	http://www.guidetopharmacology.org/GRAC/LigandDisplayForward?ligandId=2360 (Antagonist)	–
Functional Characteristics	γ = 86 pS (main state); (+ β = 44 pS)	γ = 111 pS (main state); (+ β = 54 pS)	γ = 105 pS (main state); (+ β = 48)	–

**Table:** 

Nomenclature	http://www.guidetopharmacology.org/GRAC/ObjectDisplayForward?objectId=427
HGNC, UniProt	https://www.genenames.org/data/gene‐symbol‐report/#!/hgnc_id/HGNC:4329, http://www.uniprot.org/uniprot/P48167
Selective antagonists	http://www.guidetopharmacology.org/GRAC/LigandDisplayForward?ligandId=2514 (when co‐expressed with the α1 subunit) (pIC_50_ 5.9), http://www.guidetopharmacology.org/GRAC/LigandDisplayForward?ligandId=4290 (when co‐expressed with the α1 subunit) (p*K*_i_ 5.6), http://www.guidetopharmacology.org/GRAC/LigandDisplayForward?ligandId=260 (when co‐expressed with the α2 subunit) (p*K*_i_ 5.3), http://www.guidetopharmacology.org/GRAC/LigandDisplayForward?ligandId=4290 (when co‐expressed with the α2 subunit) (p*K*_i_ 5), http://www.guidetopharmacology.org/GRAC/LigandDisplayForward?ligandId=2514 (when co‐expressed with the α3 subunit) (pIC_50_ 4.9), http://www.guidetopharmacology.org/GRAC/LigandDisplayForward?ligandId=2366 (when co‐expressed with the α2 subunit) (pIC_50_ 4.3), http://www.guidetopharmacology.org/GRAC/LigandDisplayForward?ligandId=2366 (when co‐expressed with the α1 subunit) (pIC_50_ 3.7), http://www.guidetopharmacology.org/GRAC/LigandDisplayForward?ligandId=4201 (when co‐expressed with the α1 subunit) (pIC_50_ >3.5), http://www.guidetopharmacology.org/GRAC/LigandDisplayForward?ligandId=4201 (when co‐expressed with the α2 subunit) (pIC_50_ >3.5)
Channel blockers	http://www.guidetopharmacology.org/GRAC/LigandDisplayForward?ligandId=1862 (when co‐expressed with the α2 subunit) (pIC_50_ 6.1–6.9), http://www.guidetopharmacology.org/GRAC/LigandDisplayForward?ligandId=1862 (when co‐expressed with the α1 subunit) (pIC_50_ 5.6–6.7), http://www.guidetopharmacology.org/GRAC/LigandDisplayForward?ligandId=1862 (when co‐expressed with the α3 subunit) (pIC_50_ 6.3), http://www.guidetopharmacology.org/GRAC/LigandDisplayForward?ligandId=2368 (when co‐expressed with the human α1 subunit) (pIC_50_ 5.6) [http://www.ncbi.nlm.nih.gov/pubmed/8090751?dopt=AbstractPlus] – Rat, http://www.guidetopharmacology.org/GRAC/LigandDisplayForward?ligandId=2368 (when co‐expressed with the human α2 subunit) (pIC_50_ 5.1) [http://www.ncbi.nlm.nih.gov/pubmed/8090751?dopt=AbstractPlus] – Rat, http://www.guidetopharmacology.org/GRAC/LigandDisplayForward?ligandId=2291 (when co‐expressed with the α3 subunit) (pIC_50_ 5.1), http://www.guidetopharmacology.org/GRAC/LigandDisplayForward?ligandId=4286 (when co‐expressed with the α1 subunit) (pIC_50_ 4.6), http://www.guidetopharmacology.org/GRAC/LigandDisplayForward?ligandId=4286 (when co‐expressed with the α3 subunit) (pIC_50_ 4.6), http://www.guidetopharmacology.org/GRAC/LigandDisplayForward?ligandId=2291 (when co‐expressed with the α1 subunit) (pIC_50_ 4.6), http://www.guidetopharmacology.org/GRAC/LigandDisplayForward?ligandId=4051 (when co‐expressed with the α2 subunit) (pIC_50_ 4.5), http://www.guidetopharmacology.org/GRAC/LigandDisplayForward?ligandId=4051 (when co‐expressed with the α1 subunit) (pIC_50_ 3.7)
Endogenous allosteric modulators	http://www.guidetopharmacology.org/GRAC/LigandDisplayForward?ligandId=566 (Inhibition) (pIC_50_ 4.9), http://www.guidetopharmacology.org/GRAC/LigandDisplayForward?ligandId=566 (Inhibition) (pIC_50_ 3.7)
Comments	Ligand interaction data for hetero‐oligomer receptors containing the β subunit are also listed under the α subunit

### Comments

Data in the table refer to homo‐oligomeric assemblies of the α‐subunit, significant changes introduced by coexpression of the β1 subunit are indicated in parenthesis. Not all glycine receptor ligands are listed within the table, but some that may be useful in distinguishing between glycine receptor isoforms are indicated (see detailed view pages for each subunit: http://www.guidetopharmacology.org/GRAC/ObjectDisplayForward?objectId=423&familyId=73&familyType=IC, http://www.guidetopharmacology.org/GRAC/ObjectDisplayForward?objectId=424&familyId=73&familyType=IC, http://www.guidetopharmacology.org/GRAC/ObjectDisplayForward?objectId=425&familyId=73&familyType=IC, http://www.guidetopharmacology.org/GRAC/ObjectDisplayForward?objectId=426&familyId=73&familyType=IC, http://www.guidetopharmacology.org/GRAC/ObjectDisplayForward?objectId=427&familyId=73&familyType=IC). http://www.guidetopharmacology.org/GRAC/LigandDisplayForward?ligandId=4290, http://www.guidetopharmacology.org/GRAC/LigandDisplayForward?ligandId=260 and http://www.guidetopharmacology.org/GRAC/LigandDisplayForward?ligandId=2367, for example, although not selective antagonists of glycine receptors, are included for this purpose. http://www.guidetopharmacology.org/GRAC/LigandDisplayForward?ligandId=347 is a potent and selective competitive glycine receptor antagonist with affinities in the range 5–15 nM. http://www.guidetopharmacology.org/GRAC/LigandDisplayForward?ligandId=2378 demonstrates comparable potency, but additionally blocks GABA_A_ receptors. There are conflicting reports concerning the ability of cannabinoids to inhibit [http://www.ncbi.nlm.nih.gov/pubmed/16107637?dopt=AbstractPlus], or potentiate and at high concentrations activate [http://www.ncbi.nlm.nih.gov/pubmed/19204413?dopt=AbstractPlus, http://www.ncbi.nlm.nih.gov/pubmed/19307742?dopt=AbstractPlus, http://www.ncbi.nlm.nih.gov/pubmed/16332990?dopt=AbstractPlus, http://www.ncbi.nlm.nih.gov/pubmed/21460829?dopt=AbstractPlus, http://www.ncbi.nlm.nih.gov/pubmed/18755158?dopt=AbstractPlus] glycine receptors. Nonetheless, cannabinoid analogues may hold promise in distinguishing between glycine receptor subtypes [http://www.ncbi.nlm.nih.gov/pubmed/18755158?dopt=AbstractPlus]. In addition, potentiation of glycine receptor activity by cannabinoids has been claimed to contribute to cannabis‐induced analgesia relying on Ser296/307 (α1/α3) in M3 [http://www.ncbi.nlm.nih.gov/pubmed/21460829?dopt=AbstractPlus]. Several analogues of http://www.guidetopharmacology.org/GRAC/LigandDisplayForward?ligandId=4259 and http://www.guidetopharmacology.org/GRAC/LigandDisplayForward?ligandId=5477 act as agonists and antagonists of both glycine and GABA_A_ receptors. http://www.guidetopharmacology.org/GRAC/LigandDisplayForward?ligandId=4051 acts as an allosteric inhibitor that appears to bind within the pore, and shows strong selectivity towards homomeric receptors. While its components, http://www.guidetopharmacology.org/GRAC/LigandDisplayForward?ligandId=2291 and http://www.guidetopharmacology.org/GRAC/LigandDisplayForward?ligandId=4286, have equal potencies at α1 receptors, their potencies at α2 and α3 receptors differ modestly and may allow some distinction between different receptor types [http://www.ncbi.nlm.nih.gov/pubmed/17714449?dopt=AbstractPlus]. Binding of picrotoxin within the pore has been demonstrated in the crystal structure of the related *C. elegans* GluCl Cys‐loop receptor [http://www.ncbi.nlm.nih.gov/pubmed/21572436?dopt=AbstractPlus]. In addition to the compounds listed in the table, numerous agents act as allosteric regulators of glycine receptors (comprehensively reviewed in [http://www.ncbi.nlm.nih.gov/pubmed/12413807?dopt=AbstractPlus, http://www.ncbi.nlm.nih.gov/pubmed/15383648?dopt=AbstractPlus, http://www.ncbi.nlm.nih.gov/pubmed/17692006?dopt=AbstractPlus, http://www.ncbi.nlm.nih.gov/pubmed/21557733?dopt=AbstractPlus]). Zn^2+^ acts through distinct binding sites of high‐ and low‐affinity to allosterically enhance channel function at low (<10 *μ*M) concentrations and inhibits responses at higher concentrations in a subunit selective manner [http://www.ncbi.nlm.nih.gov/pubmed/15905212?dopt=AbstractPlus]. The effect of Zn^2+^ is somewhat mimicked by Ni^2+^. Endogenous Zn^2+^ is essential for normal glycinergic neurotransmission mediated by α1 subunit‐containing receptors [http://www.ncbi.nlm.nih.gov/pubmed/17114051?dopt=AbstractPlus]. Elevation of intracellular Ca^2+^ produces fast potentiation of glycine receptor‐mediated responses. Dideoxyforskolin (4 *μ*M) and http://www.guidetopharmacology.org/GRAC/LigandDisplayForward?ligandId=1016 (0.2–5 *μ*M) both potentiate responses to low glycine concentrations (15 *μ*M), but act as inhibitors at higher glycine concentrations (100 *μ*M). Additional modulatory agents that enhance glycine receptor function include inhalational, and several intravenous general anaesthetics (*e.g.* http://www.guidetopharmacology.org/GRAC/LigandDisplayForward?ligandId=5478, http://www.guidetopharmacology.org/GRAC/LigandDisplayForward?ligandId=5464 and http://www.guidetopharmacology.org/GRAC/LigandDisplayForward?ligandId=5480) and certain neurosteroids. http://www.guidetopharmacology.org/GRAC/LigandDisplayForward?ligandId=2299 and higher order n‐alcohols also enhance glycine receptor function although whether this occurs by a direct allosteric action at the receptor [http://www.ncbi.nlm.nih.gov/pubmed/10908659?dopt=AbstractPlus], or through βγ subunits [http://www.ncbi.nlm.nih.gov/pubmed/20647311?dopt=AbstractPlus] is debated. Recent crystal structures of the bacterial homologue, GLIC, have identified transmembrane binding pockets for both anaesthetics [http://www.ncbi.nlm.nih.gov/pubmed/21248852?dopt=AbstractPlus] and alcohols [http://www.ncbi.nlm.nih.gov/pubmed/21730162?dopt=AbstractPlus]. Solvents inhaled as drugs of abuse (*e.g.* http://www.guidetopharmacology.org/GRAC/LigandDisplayForward?ligandId=5481, http://www.guidetopharmacology.org/GRAC/LigandDisplayForward?ligandId=5482) may act at sites that overlap with those recognising alcohols and volatile anaesthetics to produce potentiation of glycine receptor function. The function of glycine receptors formed as homomeric complexes of α1 or α2 subunits, or hetero‐oligomers of α1/β or α2/β subunits, is differentially affected by the 5‐HT_3_ receptor antagonist tropisetron (ICS 205‐930) which may evoke potentiation (which may occur within the femtomolar range at the homomeric glycine α1 receptor), or inhibition, depending upon the subunit composition of the receptor and the concentrations of the modulator and glycine employed. Potentiation and inhibition by tropeines involves different binding modes [http://www.ncbi.nlm.nih.gov/pubmed/19383091?dopt=AbstractPlus]. Additional tropeines, including http://www.guidetopharmacology.org/GRAC/LigandDisplayForward?ligandId=320, modulate glycine receptor activity.

### Further reading on Glycine receptors

Burgos CF *et al*. (2016) Structure and Pharmacologic Modulation of Inhibitory Glycine Receptors. *Mol. Pharmacol*. **90**: 318‐25 https://www.ncbi.nlm.nih.gov/pubmed/27401877?dopt=AbstractPlus

Dutertre S *et al*. (2012) Inhibitory glycine receptors: an update. *J. Biol. Chem*. **287**: 40216‐23 https://www.ncbi.nlm.nih.gov/pubmed/23038260?dopt=AbstractPlus

Lynch JW. (2004) Molecular structure and function of the glycine receptor chloride channel. *Physiol. Rev.* **84**: 1051‐95 https://www.ncbi.nlm.nih.gov/pubmed/15383648?dopt=AbstractPlus

Perkins DI *et al*. (2010) Molecular targets and mechanisms for ethanol action in glycine receptors. *Pharmacol. Ther*. **127**: 53‐65 https://www.ncbi.nlm.nih.gov/pubmed/20399807?dopt=AbstractPlus

Yevenes GE *et al*. (2011) Allosteric modulation of glycine receptors. *Br. J. Pharmacol*. **164**: 224‐36 https://www.ncbi.nlm.nih.gov/pubmed/21557733?dopt=AbstractPlus

## http://www.guidetopharmacology.org/GRAC/FamilyDisplayForward?familyId=75

### Overview

The ionotropic glutamate receptors comprise members of the NMDA (N‐methyl‐D‐aspartate), AMPA (α‐amino3‐hydroxy‐5‐methyl‐4‐isoxazoleproprionic acid) and kainate receptor classes, named originally according to their preferred, synthetic, agonist [http://www.ncbi.nlm.nih.gov/pubmed/10049997?dopt=AbstractPlus, http://www.ncbi.nlm.nih.gov/pubmed/18765242?dopt=AbstractPlus, http://www.ncbi.nlm.nih.gov/pubmed/20716669?dopt=AbstractPlus]. Receptor heterogeneity within each class arises from the homo‐oligomeric, or hetero‐oligomeric, assembly of distinct subunits into cation‐selective tetramers. Each subunit of the tetrameric complex comprises an extracellular amino terminal domain (ATD), an extracellular ligand binding domain (LBD), three transmembrane domains composed of three membrane spans (M1, M3 and M4), a channel lining re‐entrant ‘p‐loop’ (M2) located between M1 and M3 and an intracellular carboxy‐ terminal domain (CTD) [http://www.ncbi.nlm.nih.gov/pubmed/20491632?dopt=AbstractPlus, http://www.ncbi.nlm.nih.gov/pubmed/22974439?dopt=AbstractPlus, http://www.ncbi.nlm.nih.gov/pubmed/16554805?dopt=AbstractPlus, http://www.ncbi.nlm.nih.gov/pubmed/21080238?dopt=AbstractPlus, http://www.ncbi.nlm.nih.gov/pubmed/20716669?dopt=AbstractPlus]. The X‐ray structure of a homomeric ionotropic glutamate receptor (GluA2 – see below) has recently been solved at 3.6Å resolution [http://www.ncbi.nlm.nih.gov/pubmed/19946266?dopt=AbstractPlus] and although providing the most complete structural information current available may not representative of the subunit arrangement of, for example, the heteromeric NMDA receptors [http://www.ncbi.nlm.nih.gov/pubmed/21677647?dopt=AbstractPlus]. It is beyond the scope of this supplement to discuss the pharmacology of individual ionotropic glutamate receptor isoforms in detail; such information can be gleaned from [http://www.ncbi.nlm.nih.gov/pubmed/17962328?dopt=AbstractPlus, http://www.ncbi.nlm.nih.gov/pubmed/15494561?dopt=AbstractPlus, http://www.ncbi.nlm.nih.gov/pubmed/10049997?dopt=AbstractPlus, http://www.ncbi.nlm.nih.gov/pubmed/17622578?dopt=AbstractPlus, http://www.ncbi.nlm.nih.gov/pubmed/18793656?dopt=AbstractPlus, 434, http://www.ncbi.nlm.nih.gov/pubmed/15731895?dopt=AbstractPlus, http://www.ncbi.nlm.nih.gov/pubmed/21395862?dopt=AbstractPlus, http://www.ncbi.nlm.nih.gov/pubmed/23686171?dopt=AbstractPlus, http://www.ncbi.nlm.nih.gov/pubmed/17088105?dopt=AbstractPlus, http://www.ncbi.nlm.nih.gov/pubmed/20716669?dopt=AbstractPlus, http://www.ncbi.nlm.nih.gov/pubmed/23376022?dopt=AbstractPlus]. Agents that discriminate between subunit isoforms are, where appropriate, noted in the tables and additional compounds that distinguish between receptor isoforms are indicated in the text below.

**The classification of glutamate receptor subunits has been re‐addressed by NC‐IUPHAR** [http://www.ncbi.nlm.nih.gov/pubmed/18655795?dopt=AbstractPlus]. The scheme developed recommends a nomenclature for ionotropic glutamate receptor subunits that is adopted here.

### AMPA and Kainate receptors

AMPA receptors assemble as homomers, or heteromers, that may be drawn from GluA1, GluA2, GluA3 and GluA4 subunits. Transmembrane AMPA receptor regulatory proteins (TARPs) of class I (i.e. γ2, γ3, γ4 and γ8) act, with variable stoichiometry, as auxiliary subunits to AMPA receptors and influence their trafficking, single channel conductance gating and pharmacology (reviewed in [http://www.ncbi.nlm.nih.gov/pubmed/18026130?dopt=AbstractPlus, http://www.ncbi.nlm.nih.gov/pubmed/21521608?dopt=AbstractPlus, http://www.ncbi.nlm.nih.gov/pubmed/18514334?dopt=AbstractPlus, http://www.ncbi.nlm.nih.gov/pubmed/20134027?dopt=AbstractPlus]). Functional kainate receptors can be expressed as homomers of GluK1, GluK2 or GluK3 subunits. GluK1‐3 subunits are also capable of assembling into heterotetramers (*e.g*. GluK1/K2; [http://www.ncbi.nlm.nih.gov/pubmed/16361114?dopt=AbstractPlus, http://www.ncbi.nlm.nih.gov/pubmed/20850188?dopt=AbstractPlus, http://www.ncbi.nlm.nih.gov/pubmed/16847640?dopt=AbstractPlus]). Two additional kainate receptor subunits, GluK4 and GluK5, when expressed individually, form high affinity binding sites for http://www.guidetopharmacology.org/GRAC/LigandDisplayForward?ligandId=4231, but lack function, but can form heteromers when expressed with GluK1‐3 subunits (*e.g*. GluK2/K5; reviewed in [http://www.ncbi.nlm.nih.gov/pubmed/18793656?dopt=AbstractPlus, http://www.ncbi.nlm.nih.gov/pubmed/20850188?dopt=AbstractPlus, http://www.ncbi.nlm.nih.gov/pubmed/16847640?dopt=AbstractPlus]). Kainate receptors may also exhibit ‘metabotropic’ functions [http://www.ncbi.nlm.nih.gov/pubmed/16361114?dopt=AbstractPlus, http://www.ncbi.nlm.nih.gov/pubmed/17981346?dopt=AbstractPlus]. As found for AMPA receptors, kainate receptors are modulated by auxiliary subunits (Neto proteins, [http://www.ncbi.nlm.nih.gov/pubmed/21709676?dopt=AbstractPlus, http://www.ncbi.nlm.nih.gov/pubmed/20850188?dopt=AbstractPlus]). An important function difference between AMPA and kainate receptors is that the latter require extracellular Na+ and Cl‐ for their activation [http://www.ncbi.nlm.nih.gov/pubmed/19822544?dopt=AbstractPlus, http://www.ncbi.nlm.nih.gov/pubmed/21713670?dopt=AbstractPlus]. RNA encoding the GluA2 subunit undergoes extensive RNA editing in which the codon encoding a p‐loop glutamine residue (Q) is converted to one encoding arginine (R). This Q/R site strongly influences the biophysical properties of the receptor. Recombinant AMPA receptors lacking RNA edited GluA2 subunits are: (1) permeable to Ca^2+^; (2) blocked by intracellular polyamines at depolarized potentials causing inward rectification (the latter being reduced by TARPs); (3) blocked by extracellular argiotoxin and Joro spider toxins and (4) demonstrate higher channel conductances than receptors containing the edited form of GluA2 [http://www.ncbi.nlm.nih.gov/pubmed/17582328?dopt=AbstractPlus, http://www.ncbi.nlm.nih.gov/pubmed/12850211?dopt=AbstractPlus]. GluK1 and GluK2, but not other kainate receptor subunits, are similarly edited and broadly similar functional characteristics apply to kainate receptors lacking either an RNA edited GluK1, or GluK2, subunit [http://www.ncbi.nlm.nih.gov/pubmed/16361114?dopt=AbstractPlus, http://www.ncbi.nlm.nih.gov/pubmed/20850188?dopt=AbstractPlus]. Native AMPA and kainate receptors displaying differential channel conductances, Ca^2+^ permeabilites and sensitivity to block by intracellular polyamines have been identified [http://www.ncbi.nlm.nih.gov/pubmed/16713244?dopt=AbstractPlus, http://www.ncbi.nlm.nih.gov/pubmed/17582328?dopt=AbstractPlus, http://www.ncbi.nlm.nih.gov/pubmed/17275103?dopt=AbstractPlus]. GluA1‐4 can exist as two variants generated by alternative splicing (termed ‘flip’ and ‘flop’) that differ in their desensitization kinetics and their desensitization in the presence of cyclothiazide which stabilises the nondesensitized state. TARPs also stabilise the non‐desensitized conformation of AMPA receptors and facilitate the action of http://www.guidetopharmacology.org/GRAC/LigandDisplayForward?ligandId=4167 [http://www.ncbi.nlm.nih.gov/pubmed/18514334?dopt=AbstractPlus]. Splice variants of GluK1‐3 also exist which affects their trafficking [http://www.ncbi.nlm.nih.gov/pubmed/16361114?dopt=AbstractPlus, http://www.ncbi.nlm.nih.gov/pubmed/20850188?dopt=AbstractPlus].

**Table:** 

Nomenclature	http://www.guidetopharmacology.org/GRAC/ObjectDisplayForward?objectId=444	http://www.guidetopharmacology.org/GRAC/ObjectDisplayForward?objectId=445	http://www.guidetopharmacology.org/GRAC/ObjectDisplayForward?objectId=446	http://www.guidetopharmacology.org/GRAC/ObjectDisplayForward?objectId=447
HGNC, UniProt	https://www.genenames.org/data/gene‐symbol‐report/#!/hgnc_id/HGNC:4571, http://www.uniprot.org/uniprot/P42261	https://www.genenames.org/data/gene‐symbol‐report/#!/hgnc_id/HGNC:4572, http://www.uniprot.org/uniprot/P42262	https://www.genenames.org/data/gene‐symbol‐report/#!/hgnc_id/HGNC:4573, http://www.uniprot.org/uniprot/P42263	https://www.genenames.org/data/gene‐symbol‐report/#!/hgnc_id/HGNC:4574, http://www.uniprot.org/uniprot/P48058
Agonists	(http://www.guidetopharmacology.org/GRAC/LigandDisplayForward?ligandId=4070, http://www.guidetopharmacology.org/GRAC/LigandDisplayForward?ligandId=4131	(http://www.guidetopharmacology.org/GRAC/LigandDisplayForward?ligandId=4070, http://www.guidetopharmacology.org/GRAC/LigandDisplayForward?ligandId=4131	(http://www.guidetopharmacology.org/GRAC/LigandDisplayForward?ligandId=4070, http://www.guidetopharmacology.org/GRAC/LigandDisplayForward?ligandId=4131	(http://www.guidetopharmacology.org/GRAC/LigandDisplayForward?ligandId=4070, http://www.guidetopharmacology.org/GRAC/LigandDisplayForward?ligandId=4131
Selective antagonists	http://www.guidetopharmacology.org/GRAC/LigandDisplayForward?ligandId=4141, http://www.guidetopharmacology.org/GRAC/LigandDisplayForward?ligandId=4209, http://www.guidetopharmacology.org/GRAC/LigandDisplayForward?ligandId=4210 (active isomer, non‐competitive), http://www.guidetopharmacology.org/GRAC/LigandDisplayForward?ligandId=4264, http://www.guidetopharmacology.org/GRAC/LigandDisplayForward?ligandId=4245	http://www.guidetopharmacology.org/GRAC/LigandDisplayForward?ligandId=4141, http://www.guidetopharmacology.org/GRAC/LigandDisplayForward?ligandId=4209, http://www.guidetopharmacology.org/GRAC/LigandDisplayForward?ligandId=4210 (active isomer, non‐competitive), http://www.guidetopharmacology.org/GRAC/LigandDisplayForward?ligandId=4264, http://www.guidetopharmacology.org/GRAC/LigandDisplayForward?ligandId=4245	http://www.guidetopharmacology.org/GRAC/LigandDisplayForward?ligandId=4141, http://www.guidetopharmacology.org/GRAC/LigandDisplayForward?ligandId=4209, http://www.guidetopharmacology.org/GRAC/LigandDisplayForward?ligandId=4210 (active isomer, non‐competitive), http://www.guidetopharmacology.org/GRAC/LigandDisplayForward?ligandId=4264, http://www.guidetopharmacology.org/GRAC/LigandDisplayForward?ligandId=4245	http://www.guidetopharmacology.org/GRAC/LigandDisplayForward?ligandId=4141, http://www.guidetopharmacology.org/GRAC/LigandDisplayForward?ligandId=4209, http://www.guidetopharmacology.org/GRAC/LigandDisplayForward?ligandId=4210 (active isomer, non‐competitive), http://www.guidetopharmacology.org/GRAC/LigandDisplayForward?ligandId=4264, http://www.guidetopharmacology.org/GRAC/LigandDisplayForward?ligandId=4245
Channel blockers	extracellular http://www.guidetopharmacology.org/GRAC/LigandDisplayForward?ligandId=4138, extracellular http://www.guidetopharmacology.org/GRAC/LigandDisplayForward?ligandId=4229 (selective for channels lacking GluA2)	extracellular http://www.guidetopharmacology.org/GRAC/LigandDisplayForward?ligandId=4138	extracellular http://www.guidetopharmacology.org/GRAC/LigandDisplayForward?ligandId=4138, extracellular http://www.guidetopharmacology.org/GRAC/LigandDisplayForward?ligandId=4229 (selective for channels lacking GluA2)	extracellular http://www.guidetopharmacology.org/GRAC/LigandDisplayForward?ligandId=4138, extracellular http://www.guidetopharmacology.org/GRAC/LigandDisplayForward?ligandId=4229 (selective for channels lacking GluA2)
Allosteric modulators	http://www.guidetopharmacology.org/GRAC/LigandDisplayForward?ligandId=4248 (Positive) (pEC_50_ 5.8) [http://www.ncbi.nlm.nih.gov/pubmed/11406188?dopt=AbstractPlus], http://www.guidetopharmacology.org/GRAC/LigandDisplayForward?ligandId=4249 (Positive) (pEC_50_ 5.2) [http://www.ncbi.nlm.nih.gov/pubmed/11406188?dopt=AbstractPlus], http://www.guidetopharmacology.org/GRAC/LigandDisplayForward?ligandId=4167 (Positive) (pEC_50_ 4.7) [http://www.ncbi.nlm.nih.gov/pubmed/11406188?dopt=AbstractPlus], http://www.guidetopharmacology.org/GRAC/LigandDisplayForward?ligandId=4165 (Positive), http://www.guidetopharmacology.org/GRAC/LigandDisplayForward?ligandId=4166 (Positive), http://www.guidetopharmacology.org/GRAC/LigandDisplayForward?ligandId=4219 (Positive), http://www.guidetopharmacology.org/GRAC/LigandDisplayForward?ligandId=4251 (Positive), http://www.guidetopharmacology.org/GRAC/LigandDisplayForward?ligandId=4304 (Positive), http://www.guidetopharmacology.org/GRAC/LigandDisplayForward?ligandId=4133 (Positive), http://www.guidetopharmacology.org/GRAC/LigandDisplayForward?ligandId=4288 (Positive)	http://www.guidetopharmacology.org/GRAC/LigandDisplayForward?ligandId=4249 (Positive) (pEC_50_ 6.8) [http://www.ncbi.nlm.nih.gov/pubmed/11406188?dopt=AbstractPlus], http://www.guidetopharmacology.org/GRAC/LigandDisplayForward?ligandId=4248 (Positive) (pEC_50_ 6.7) [http://www.ncbi.nlm.nih.gov/pubmed/11406188?dopt=AbstractPlus], http://www.guidetopharmacology.org/GRAC/LigandDisplayForward?ligandId=4167 (Positive) (pEC_50_ 5.7) [http://www.ncbi.nlm.nih.gov/pubmed/11406188?dopt=AbstractPlus], http://www.guidetopharmacology.org/GRAC/LigandDisplayForward?ligandId=4165 (Positive), http://www.guidetopharmacology.org/GRAC/LigandDisplayForward?ligandId=4166 (Positive), http://www.guidetopharmacology.org/GRAC/LigandDisplayForward?ligandId=4219 (Positive), http://www.guidetopharmacology.org/GRAC/LigandDisplayForward?ligandId=4251 (Positive), http://www.guidetopharmacology.org/GRAC/LigandDisplayForward?ligandId=4304 (Positive), http://www.guidetopharmacology.org/GRAC/LigandDisplayForward?ligandId=4133 (Positive), http://www.guidetopharmacology.org/GRAC/LigandDisplayForward?ligandId=4288 (Positive)	http://www.guidetopharmacology.org/GRAC/LigandDisplayForward?ligandId=4249 (Positive) (pEC_50_ 5.8) [http://www.ncbi.nlm.nih.gov/pubmed/11406188?dopt=AbstractPlus], http://www.guidetopharmacology.org/GRAC/LigandDisplayForward?ligandId=4248 (Positive) (pEC_50_ 5.7) [http://www.ncbi.nlm.nih.gov/pubmed/11406188?dopt=AbstractPlus], http://www.guidetopharmacology.org/GRAC/LigandDisplayForward?ligandId=4167 (Positive) (pEC_50_ 4.9) [http://www.ncbi.nlm.nih.gov/pubmed/11406188?dopt=AbstractPlus], http://www.guidetopharmacology.org/GRAC/LigandDisplayForward?ligandId=4165 (Positive), http://www.guidetopharmacology.org/GRAC/LigandDisplayForward?ligandId=4166 (Positive), http://www.guidetopharmacology.org/GRAC/LigandDisplayForward?ligandId=4219 (Positive), http://www.guidetopharmacology.org/GRAC/LigandDisplayForward?ligandId=4251 (Positive), http://www.guidetopharmacology.org/GRAC/LigandDisplayForward?ligandId=4304 (Positive), http://www.guidetopharmacology.org/GRAC/LigandDisplayForward?ligandId=4133 (Positive), http://www.guidetopharmacology.org/GRAC/LigandDisplayForward?ligandId=4288 (Positive)	http://www.guidetopharmacology.org/GRAC/LigandDisplayForward?ligandId=4248 (Positive) (pEC_50_ 6.7) [http://www.ncbi.nlm.nih.gov/pubmed/11406188?dopt=AbstractPlus], http://www.guidetopharmacology.org/GRAC/LigandDisplayForward?ligandId=4249 (Positive) (pEC_50_ 6.7) [http://www.ncbi.nlm.nih.gov/pubmed/11406188?dopt=AbstractPlus], http://www.guidetopharmacology.org/GRAC/LigandDisplayForward?ligandId=4167 (Positive) (pEC_50_ 5.4) [http://www.ncbi.nlm.nih.gov/pubmed/11406188?dopt=AbstractPlus], http://www.guidetopharmacology.org/GRAC/LigandDisplayForward?ligandId=4165 (Positive), http://www.guidetopharmacology.org/GRAC/LigandDisplayForward?ligandId=4166 (Positive), http://www.guidetopharmacology.org/GRAC/LigandDisplayForward?ligandId=4219 (Positive), http://www.guidetopharmacology.org/GRAC/LigandDisplayForward?ligandId=4251 (Positive), http://www.guidetopharmacology.org/GRAC/LigandDisplayForward?ligandId=4304 (Positive), http://www.guidetopharmacology.org/GRAC/LigandDisplayForward?ligandId=4133 (Positive), http://www.guidetopharmacology.org/GRAC/LigandDisplayForward?ligandId=4288 (Positive)
Labelled ligands	http://www.guidetopharmacology.org/GRAC/LigandDisplayForward?ligandId=4077 (Agonist), http://www.guidetopharmacology.org/GRAC/LigandDisplayForward?ligandId=4081 (Antagonist)	http://www.guidetopharmacology.org/GRAC/LigandDisplayForward?ligandId=4077 (Agonist), http://www.guidetopharmacology.org/GRAC/LigandDisplayForward?ligandId=4081 (Antagonist)	http://www.guidetopharmacology.org/GRAC/LigandDisplayForward?ligandId=4077, http://www.guidetopharmacology.org/GRAC/LigandDisplayForward?ligandId=4081	http://www.guidetopharmacology.org/GRAC/LigandDisplayForward?ligandId=4077 (Agonist), http://www.guidetopharmacology.org/GRAC/LigandDisplayForward?ligandId=4081
Comments	http://www.guidetopharmacology.org/GRAC/LigandDisplayForward?ligandId=4288 and http://www.guidetopharmacology.org/GRAC/LigandDisplayForward?ligandId=4133 are examples of pyrrolidinones. http://www.guidetopharmacology.org/GRAC/LigandDisplayForward?ligandId=4167, http://www.guidetopharmacology.org/GRAC/LigandDisplayForward?ligandId=4304, and http://www.guidetopharmacology.org/GRAC/LigandDisplayForward?ligandId=4219 are examples of benzothiadiazides. http://www.guidetopharmacology.org/GRAC/LigandDisplayForward?ligandId=4165 and http://www.guidetopharmacology.org/GRAC/LigandDisplayForward?ligandId=4166 are examples of benzylpiperidines. http://www.guidetopharmacology.org/GRAC/LigandDisplayForward?ligandId=4248, http://www.guidetopharmacology.org/GRAC/LigandDisplayForward?ligandId=4249 and http://www.guidetopharmacology.org/GRAC/LigandDisplayForward?ligandId=4251 are examples of biarylpropylsulfonamides. Also blocked by intracellular polyamines.			

**Table:** 

Nomenclature	http://www.guidetopharmacology.org/GRAC/ObjectDisplayForward?objectId=448	http://www.guidetopharmacology.org/GRAC/ObjectDisplayForward?objectId=449
HGNC, UniProt	https://www.genenames.org/data/gene‐symbol‐report/#!/hgnc_id/HGNC:4575, http://www.uniprot.org/uniprot/Q9ULK0	https://www.genenames.org/data/gene‐symbol‐report/#!/hgnc_id/HGNC:4576, http://www.uniprot.org/uniprot/O43424

### Comments

GluD1 and GluD2 comprise, on the basis of sequence homology, an ‘orphan’ class of ionotropic glutamate receptor subunit. They do not form a functional receptor when expressed solely, or in combination with other ionotropic glutamate receptor subunits, in transfected cells [http://www.ncbi.nlm.nih.gov/pubmed/12725908?dopt=AbstractPlus]. However, GluD2 subunits bind http://www.guidetopharmacology.org/GRAC/LigandDisplayForward?ligandId=4171 and http://www.guidetopharmacology.org/GRAC/LigandDisplayForward?ligandId=727 and GluD2 subunits carrying the mutation A654T form a spontaneously open channel that is closed by http://www.guidetopharmacology.org/GRAC/LigandDisplayForward?ligandId=4171 [http://www.ncbi.nlm.nih.gov/pubmed/17715062?dopt=AbstractPlus].

**Table:** 

Nomenclature	http://www.guidetopharmacology.org/GRAC/ObjectDisplayForward?objectId=450	http://www.guidetopharmacology.org/GRAC/ObjectDisplayForward?objectId=451	http://www.guidetopharmacology.org/GRAC/ObjectDisplayForward?objectId=452	http://www.guidetopharmacology.org/GRAC/ObjectDisplayForward?objectId=453	http://www.guidetopharmacology.org/GRAC/ObjectDisplayForward?objectId=454
HGNC, UniProt	https://www.genenames.org/data/gene‐symbol‐report/#!/hgnc_id/HGNC:4579, http://www.uniprot.org/uniprot/P39086	https://www.genenames.org/data/gene‐symbol‐report/#!/hgnc_id/HGNC:4580, http://www.uniprot.org/uniprot/Q13002	https://www.genenames.org/data/gene‐symbol‐report/#!/hgnc_id/HGNC:4581, http://www.uniprot.org/uniprot/Q13003	https://www.genenames.org/data/gene‐symbol‐report/#!/hgnc_id/HGNC:4582, http://www.uniprot.org/uniprot/Q16099	https://www.genenames.org/data/gene‐symbol‐report/#!/hgnc_id/HGNC:4583, http://www.uniprot.org/uniprot/Q16478
Agonists	http://www.guidetopharmacology.org/GRAC/LigandDisplayForward?ligandId=4185 [http://www.ncbi.nlm.nih.gov/pubmed/11160654?dopt=AbstractPlus] – Rat, http://www.guidetopharmacology.org/GRAC/LigandDisplayForward?ligandId=4317 [http://www.ncbi.nlm.nih.gov/pubmed/10821708?dopt=AbstractPlus], http://www.guidetopharmacology.org/GRAC/LigandDisplayForward?ligandId=4231 [http://www.ncbi.nlm.nih.gov/pubmed/9849663?dopt=AbstractPlus], (http://www.guidetopharmacology.org/GRAC/LigandDisplayForward?ligandId=4069, (http://www.guidetopharmacology.org/GRAC/LigandDisplayForward?ligandId=4071, http://www.guidetopharmacology.org/GRAC/LigandDisplayForward?ligandId=4112, http://www.guidetopharmacology.org/GRAC/LigandDisplayForward?ligandId=4140, http://www.guidetopharmacology.org/GRAC/LigandDisplayForward?ligandId=4181	http://www.guidetopharmacology.org/GRAC/LigandDisplayForward?ligandId=4185 [http://www.ncbi.nlm.nih.gov/pubmed/11160654?dopt=AbstractPlus] – Rat, http://www.guidetopharmacology.org/GRAC/LigandDisplayForward?ligandId=4181 [http://www.ncbi.nlm.nih.gov/pubmed/8809152?dopt=AbstractPlus], http://www.guidetopharmacology.org/GRAC/LigandDisplayForward?ligandId=4317 [http://www.ncbi.nlm.nih.gov/pubmed/8996224?dopt=AbstractPlus] – Rat, http://www.guidetopharmacology.org/GRAC/LigandDisplayForward?ligandId=4231 [http://www.ncbi.nlm.nih.gov/pubmed/8809152?dopt=AbstractPlus, http://www.ncbi.nlm.nih.gov/pubmed/9849663?dopt=AbstractPlus]	http://www.guidetopharmacology.org/GRAC/LigandDisplayForward?ligandId=4317 [http://www.ncbi.nlm.nih.gov/pubmed/18578478?dopt=AbstractPlus] – Rat, http://www.guidetopharmacology.org/GRAC/LigandDisplayForward?ligandId=4231 (low potency) [http://www.ncbi.nlm.nih.gov/pubmed/18578478?dopt=AbstractPlus] – Rat, http://www.guidetopharmacology.org/GRAC/LigandDisplayForward?ligandId=4185	http://www.guidetopharmacology.org/GRAC/LigandDisplayForward?ligandId=4317, http://www.guidetopharmacology.org/GRAC/LigandDisplayForward?ligandId=4181, http://www.guidetopharmacology.org/GRAC/LigandDisplayForward?ligandId=4185, http://www.guidetopharmacology.org/GRAC/LigandDisplayForward?ligandId=4231	http://www.guidetopharmacology.org/GRAC/LigandDisplayForward?ligandId=4317, http://www.guidetopharmacology.org/GRAC/LigandDisplayForward?ligandId=4181, http://www.guidetopharmacology.org/GRAC/LigandDisplayForward?ligandId=4185, http://www.guidetopharmacology.org/GRAC/LigandDisplayForward?ligandId=4231
Selective agonists	http://www.guidetopharmacology.org/GRAC/LigandDisplayForward?ligandId=4246 [http://www.ncbi.nlm.nih.gov/pubmed/9849663?dopt=AbstractPlus]	–	–	–	–
Selective antagonists	http://www.guidetopharmacology.org/GRAC/LigandDisplayForward?ligandId=4102, http://www.guidetopharmacology.org/GRAC/LigandDisplayForward?ligandId=4123, http://www.guidetopharmacology.org/GRAC/LigandDisplayForward?ligandId=4247, http://www.guidetopharmacology.org/GRAC/LigandDisplayForward?ligandId=4250, http://www.guidetopharmacology.org/GRAC/LigandDisplayForward?ligandId=4258, http://www.guidetopharmacology.org/GRAC/LigandDisplayForward?ligandId=4274 (non‐competitive), http://www.guidetopharmacology.org/GRAC/LigandDisplayForward?ligandId=4333, http://www.guidetopharmacology.org/GRAC/LigandDisplayForward?ligandId=4334	http://www.guidetopharmacology.org/GRAC/LigandDisplayForward?ligandId=4102	–	–	–
Allosteric modulators	http://www.guidetopharmacology.org/GRAC/LigandDisplayForward?ligandId=4162 (Positive)	http://www.guidetopharmacology.org/GRAC/LigandDisplayForward?ligandId=4162 (Positive)	–	–	–
Labelled ligands	http://www.guidetopharmacology.org/GRAC/LigandDisplayForward?ligandId=4092 (Antagonist) (p*K*_d_ 7.7) [http://www.ncbi.nlm.nih.gov/pubmed/20837679?dopt=AbstractPlus], http://www.guidetopharmacology.org/GRAC/LigandDisplayForward?ligandId=4075 (Agonist), http://www.guidetopharmacology.org/GRAC/LigandDisplayForward?ligandId=4085 (Agonist)	http://www.guidetopharmacology.org/GRAC/LigandDisplayForward?ligandId=4085 (Agonist) [http://www.ncbi.nlm.nih.gov/pubmed/8996224?dopt=AbstractPlus] – Rat, http://www.guidetopharmacology.org/GRAC/LigandDisplayForward?ligandId=4075 (Agonist)	http://www.guidetopharmacology.org/GRAC/LigandDisplayForward?ligandId=4092 (Antagonist) (p*K*_d_ 6.3) [http://www.ncbi.nlm.nih.gov/pubmed/20837679?dopt=AbstractPlus], http://www.guidetopharmacology.org/GRAC/LigandDisplayForward?ligandId=4075 (Agonist), http://www.guidetopharmacology.org/GRAC/LigandDisplayForward?ligandId=4085 (Agonist)	http://www.guidetopharmacology.org/GRAC/LigandDisplayForward?ligandId=4075 (Agonist), http://www.guidetopharmacology.org/GRAC/LigandDisplayForward?ligandId=4085 (Agonist)	http://www.guidetopharmacology.org/GRAC/LigandDisplayForward?ligandId=4075 (Agonist), http://www.guidetopharmacology.org/GRAC/LigandDisplayForward?ligandId=4085 (Agonist)
Comments	–	Intracellular polyamines are subtype selective channel blockers (GluK3 ≫ GluK2)	http://www.guidetopharmacology.org/GRAC/LigandDisplayForward?ligandId=4181 and http://www.guidetopharmacology.org/GRAC/LigandDisplayForward?ligandId=4162 are inactive at the GluK3 subunit. Intracellular polyamines are subtype selective channel blockers (GluK3 ≫ GluK2)	–	–

### Comments

All AMPA receptors are additionally activated by http://www.guidetopharmacology.org/GRAC/LigandDisplayForward?ligandId=4231 (and http://www.guidetopharmacology.org/GRAC/LigandDisplayForward?ligandId=4181) with relatively low potency, (EC_50_ 100 *μ*M). Inclusion of TARPs within the receptor complex increases the potency and maximal effect of kainate [http://www.ncbi.nlm.nih.gov/pubmed/21521608?dopt=AbstractPlus, http://www.ncbi.nlm.nih.gov/pubmed/18514334?dopt=AbstractPlus]. AMPA is weak partial agonist at GluK1 and at heteromeric assemblies of GluK1/GluK2, GluK1/GluK5 and GluK2/GluK5 [http://www.ncbi.nlm.nih.gov/pubmed/18793656?dopt=AbstractPlus]. Quinoxalinediones such as http://www.guidetopharmacology.org/GRAC/LigandDisplayForward?ligandId=5475 and http://www.guidetopharmacology.org/GRAC/LigandDisplayForward?ligandId=4264 show limited selectivity between AMPA and kainate receptors. http://www.guidetopharmacology.org/GRAC/LigandDisplayForward?ligandId=4245 also has kainate (GluK1) receptor activity as has http://www.guidetopharmacology.org/GRAC/LigandDisplayForward?ligandId=4209 (GluK3 and GluK2/GluK3) [http://www.ncbi.nlm.nih.gov/pubmed/18793656?dopt=AbstractPlus]. http://www.guidetopharmacology.org/GRAC/LigandDisplayForward?ligandId=4141 is a potent competitive antagonist of AMPA receptors, has a weaker antagonist action at kainate receptors comprising GluK1 subunits, butis devoid of activity at kainate receptors formed from GluK2 or GluK2/GluK5 subunits. The pharmacological activity of http://www.guidetopharmacology.org/GRAC/LigandDisplayForward?ligandId=4141 resides with the (S)‐enantiomer. http://www.guidetopharmacology.org/GRAC/LigandDisplayForward?ligandId=4123 and http://www.guidetopharmacology.org/GRAC/LigandDisplayForward?ligandId=4334 may block GluK3, in addition to GluK1 [http://www.ncbi.nlm.nih.gov/pubmed/20837679?dopt=AbstractPlus, http://www.ncbi.nlm.nih.gov/pubmed/18761361?dopt=AbstractPlus]. (2S,4R)‐4‐methylglutamate (http://www.guidetopharmacology.org/GRAC/LigandDisplayForward?ligandId=4317) is equipotent in activating (and desensitising) GluK1 and GluK2 receptor isoforms and, via the induction of desensitisation at low concentrations, has beenused as a functional antagonist of kainate receptors. Both (2*S*,4*R*)‐4‐methylglutamate and http://www.guidetopharmacology.org/GRAC/LigandDisplayForward?ligandId=4246 have agonist activity at NMDA receptors. (2S,4R)‐4‐methylglutamate is also an inhibitor of the glutamate transporters EAAT1 and EAAT2.

### NMDA receptors

NMDA receptors assemble as obligate heteromers that may be drawn from GluN1, GluN2A, GluN2B, GluN2C, GluN2D, GluN3A and GluN3B subunits. Alternative splicing can generate eight isoforms of GluN1 with differing pharmacological properties. Various splice variants of GluN2B, 2C, 2D and GluN3A have also been reported. Activation of NMDA receptors containing GluN1 and GluN2 subunits requires the binding of two agonists, glutamate to the S1 and S2 regions of the GluN2 subunit and glycine to S1 and S2 regions of the GluN1 subunit [http://www.ncbi.nlm.nih.gov/pubmed/16474411?dopt=AbstractPlus, http://www.ncbi.nlm.nih.gov/pubmed/15701057?dopt=AbstractPlus]. The minimal requirement for efficient functional expression of NMDA receptors *in vitro* is a di‐heteromeric assembly of GluN1 and at least one GluN2 subunit variant, as a dimer of heterodimers arrangement in the extracellular domain [http://www.ncbi.nlm.nih.gov/pubmed/16281028?dopt=AbstractPlus, http://www.ncbi.nlm.nih.gov/pubmed/21677647?dopt=AbstractPlus, http://www.ncbi.nlm.nih.gov/pubmed/16554805?dopt=AbstractPlus]. However, more complex tri‐heteromeric assemblies, incorporating multiple subtypes of GluN2 subunit, or GluN3 subunits, can be generated*in vitro* and occur*in vivo*. The NMDA receptor channel commonly has a high relative permeability to Ca^2+^ and is blocked, in a voltage‐dependent manner, by Mg^2+^ such that at resting potentials the response is substantially inhibited.

**Table:** 

Nomenclature	http://www.guidetopharmacology.org/GRAC/ObjectDisplayForward?objectId=455	http://www.guidetopharmacology.org/GRAC/ObjectDisplayForward?objectId=456	http://www.guidetopharmacology.org/GRAC/ObjectDisplayForward?objectId=457
HGNC, UniProt	https://www.genenames.org/data/gene‐symbol‐report/#!/hgnc_id/HGNC:4584, http://www.uniprot.org/uniprot/Q05586	https://www.genenames.org/data/gene‐symbol‐report/#!/hgnc_id/HGNC:4585, http://www.uniprot.org/uniprot/Q12879	https://www.genenames.org/data/gene‐symbol‐report/#!/hgnc_id/HGNC:4586, http://www.uniprot.org/uniprot/Q13224
Endogenous agonists	http://www.guidetopharmacology.org/GRAC/LigandDisplayForward?ligandId=6511 [glutamate site], http://www.guidetopharmacology.org/GRAC/LigandDisplayForward?ligandId=4171 [glycine site], http://www.guidetopharmacology.org/GRAC/LigandDisplayForward?ligandId=3309 [glutamate site], http://www.guidetopharmacology.org/GRAC/LigandDisplayForward?ligandId=727 [glycine site]	http://www.guidetopharmacology.org/GRAC/LigandDisplayForward?ligandId=6511 [glutamate site] (GluN2D > GluN2C = GluN2B > GluN2A), http://www.guidetopharmacology.org/GRAC/LigandDisplayForward?ligandId=4171 [glycine site] (GluN2D > GluN2C > GluN2B > GluN2A), http://www.guidetopharmacology.org/GRAC/LigandDisplayForward?ligandId=3309 [glutamate site] (GluN2D = GluN2B > GluN2C = GluN2A), http://www.guidetopharmacology.org/GRAC/LigandDisplayForward?ligandId=727 [glycine site] (GluN2D > GluN2C > GluN2B > GluN2A)	
Agonists	http://www.guidetopharmacology.org/GRAC/LigandDisplayForward?ligandId=4066 [glycine site] (Partial agonist), (http://www.guidetopharmacology.org/GRAC/LigandDisplayForward?ligandId=4068 [glutamate site], http://www.guidetopharmacology.org/GRAC/LigandDisplayForward?ligandId=4268 [glutamate site], http://www.guidetopharmacology.org/GRAC/LigandDisplayForward?ligandId=4216 [glutamate site] (Partial agonist)	http://www.guidetopharmacology.org/GRAC/LigandDisplayForward?ligandId=4066 [glycine site] (Partial agonist), (http://www.guidetopharmacology.org/GRAC/LigandDisplayForward?ligandId=4068 [glutamate site] (GluN2D > GluN2C = GluN2B > GluN2A), http://www.guidetopharmacology.org/GRAC/LigandDisplayForward?ligandId=4268 [glutamate site] (GluN2D > GluN2C > GluN2B > GluN2A), http://www.guidetopharmacology.org/GRAC/LigandDisplayForward?ligandId=4216 [glutamate site] (GluN2B ≥ GluN2A ≥ GluN2D > GluN2C; partial agonist at GluN2A and GluN2C)	
Selective antagonists	http://www.guidetopharmacology.org/GRAC/LigandDisplayForward?ligandId=4240 [glycine site] (pIC_50_ 8.7) [http://www.ncbi.nlm.nih.gov/pubmed/8182696?dopt=AbstractPlus] – Rat, http://www.guidetopharmacology.org/GRAC/LigandDisplayForward?ligandId=4208 [glycine site] (p*K*_i_ 8.1–8.4) [http://www.ncbi.nlm.nih.gov/pubmed/10780999?dopt=AbstractPlus] – Rat, http://www.guidetopharmacology.org/GRAC/LigandDisplayForward?ligandId=4239 [glycine site] (pIC_50_ 8.1) [http://www.ncbi.nlm.nih.gov/pubmed/1534584?dopt=AbstractPlus] – Rat, http://www.guidetopharmacology.org/GRAC/LigandDisplayForward?ligandId=2361 [glycine site]	http://www.guidetopharmacology.org/GRAC/LigandDisplayForward?ligandId=2361 [glycine site], http://www.guidetopharmacology.org/GRAC/LigandDisplayForward?ligandId=4154 [glutamate site], http://www.guidetopharmacology.org/GRAC/LigandDisplayForward?ligandId=4208 [glycine site], http://www.guidetopharmacology.org/GRAC/LigandDisplayForward?ligandId=4239 [glycine site], http://www.guidetopharmacology.org/GRAC/LigandDisplayForward?ligandId=4240 [glycine site], http://www.guidetopharmacology.org/GRAC/LigandDisplayForward?ligandId=4243 [glutamate site], http://www.guidetopharmacology.org/GRAC/LigandDisplayForward?ligandId=4276 [glutamate site] (GluN2A > GluN2B (human), but weakly selective for rat GluN2A versus GluN2B) [http://www.ncbi.nlm.nih.gov/pubmed/11909726?dopt=AbstractPlus, http://www.ncbi.nlm.nih.gov/pubmed/14718249?dopt=AbstractPlus, http://www.ncbi.nlm.nih.gov/pubmed/16778008?dopt=AbstractPlus, http://www.ncbi.nlm.nih.gov/pubmed/16452656?dopt=AbstractPlus], http://www.guidetopharmacology.org/GRAC/LigandDisplayForward?ligandId=4332 [glutamate site] (GluN2D ≥ GluN2C > GluN2A ≥ GluN2B) [http://www.ncbi.nlm.nih.gov/pubmed/15801853?dopt=AbstractPlus], http://www.guidetopharmacology.org/GRAC/LigandDisplayForward?ligandId=4161 [glutamate site] (GluN2B > GluN2D = GluN2C = GluN2A), http://www.guidetopharmacology.org/GRAC/LigandDisplayForward?ligandId=4168 [glutamate site], http://www.guidetopharmacology.org/GRAC/LigandDisplayForward?ligandId=4170 [glutamate site] (GluN2A = GluN2B > GluN2C = GluN2D), http://www.guidetopharmacology.org/GRAC/LigandDisplayForward?ligandId=4155 [glutamate site]	
Channel blockers	–	http://www.guidetopharmacology.org/GRAC/LigandDisplayForward?ligandId=708 (GluN2A = GluN2B > GluN2C = GluN2D), http://www.guidetopharmacology.org/GRAC/LigandDisplayForward?ligandId=4260 (GluN2A = GluN2B ≫ GluN2C = GluN2D), http://www.guidetopharmacology.org/GRAC/LigandDisplayForward?ligandId=4128 (GluN2C = GluN2D ≥ GluN2B ≥ GluN2A), http://www.guidetopharmacology.org/GRAC/LigandDisplayForward?ligandId=2403, http://www.guidetopharmacology.org/GRAC/LigandDisplayForward?ligandId=4233, http://www.guidetopharmacology.org/GRAC/LigandDisplayForward?ligandId=4282	
Labelled ligands	http://www.guidetopharmacology.org/GRAC/LigandDisplayForward?ligandId=4088 [glycine site] (Antagonist) (p*K*_d_ ∼8.5) [http://www.ncbi.nlm.nih.gov/pubmed/9721050?dopt=AbstractPlus] – Rat, http://www.guidetopharmacology.org/GRAC/LigandDisplayForward?ligandId=4078 [glutamate site] (Selective Antagonist), http://www.guidetopharmacology.org/GRAC/LigandDisplayForward?ligandId=4079 [glycine site] (Antagonist), http://www.guidetopharmacology.org/GRAC/LigandDisplayForward?ligandId=4080 [glutamate site] (Antagonist), http://www.guidetopharmacology.org/GRAC/LigandDisplayForward?ligandId=4082 [glutamate site] (Selective Antagonist), http://www.guidetopharmacology.org/GRAC/LigandDisplayForward?ligandId=4086 [glycine site] (Antagonist), http://www.guidetopharmacology.org/GRAC/LigandDisplayForward?ligandId=4089 [cation channel] (Antagonist), http://www.guidetopharmacology.org/GRAC/LigandDisplayForward?ligandId=4084 [glycine site] (Agonist)	http://www.guidetopharmacology.org/GRAC/LigandDisplayForward?ligandId=4078 [glutamate site] (Antagonist), http://www.guidetopharmacology.org/GRAC/LigandDisplayForward?ligandId=4079 [glycine site] (Antagonist), http://www.guidetopharmacology.org/GRAC/LigandDisplayForward?ligandId=4080 [glutamate site] (Antagonist), http://www.guidetopharmacology.org/GRAC/LigandDisplayForward?ligandId=4082 [glutamate site] (Antagonist), http://www.guidetopharmacology.org/GRAC/LigandDisplayForward?ligandId=4086 [glycine site] (Antagonist), http://www.guidetopharmacology.org/GRAC/LigandDisplayForward?ligandId=4088 [glycine site] (Antagonist), http://www.guidetopharmacology.org/GRAC/LigandDisplayForward?ligandId=4089 [cation channel] (Channel blocker), http://www.guidetopharmacology.org/GRAC/LigandDisplayForward?ligandId=4084 [glycine site] (Agonist)	

**Table:** 

Nomenclature	http://www.guidetopharmacology.org/GRAC/ObjectDisplayForward?objectId=458	http://www.guidetopharmacology.org/GRAC/ObjectDisplayForward?objectId=459
HGNC, UniProt	https://www.genenames.org/data/gene‐symbol‐report/#!/hgnc_id/HGNC:4587, http://www.uniprot.org/uniprot/Q14957	https://www.genenames.org/data/gene‐symbol‐report/#!/hgnc_id/HGNC:4588, http://www.uniprot.org/uniprot/O15399
Endogenous agonists	http://www.guidetopharmacology.org/GRAC/LigandDisplayForward?ligandId=6511 [glutamate site] (GluN2D > GluN2C = GluN2B > GluN2A), http://www.guidetopharmacology.org/GRAC/LigandDisplayForward?ligandId=4171 [glycine site] (GluN2D > GluN2C > GluN2B > GluN2A), http://www.guidetopharmacology.org/GRAC/LigandDisplayForward?ligandId=3309 [glutamate site] (GluN2D = GluN2B > GluN2C = GluN2A), http://www.guidetopharmacology.org/GRAC/LigandDisplayForward?ligandId=727 [glycine site] (GluN2D > GluN2C > GluN2B > GluN2A)	
Agonists	(http://www.guidetopharmacology.org/GRAC/LigandDisplayForward?ligandId=4068 [glutamate site] (GluN2D > GluN2C = GluN2B > GluN2A), http://www.guidetopharmacology.org/GRAC/LigandDisplayForward?ligandId=4268 [glutamate site] (GluN2D > GluN2C > GluN2B > GluN2A), http://www.guidetopharmacology.org/GRAC/LigandDisplayForward?ligandId=4216 [glutamate site] (GluN2B ≥ GluN2A ≥ GluN2D > GluN2C; partial agonist at GluN2A and GluN2C)	
Selective antagonists	http://www.guidetopharmacology.org/GRAC/LigandDisplayForward?ligandId=2361 [glycine site], http://www.guidetopharmacology.org/GRAC/LigandDisplayForward?ligandId=4154 [glutamate site], http://www.guidetopharmacology.org/GRAC/LigandDisplayForward?ligandId=4208 [glycine site], http://www.guidetopharmacology.org/GRAC/LigandDisplayForward?ligandId=4239 [glycine site], http://www.guidetopharmacology.org/GRAC/LigandDisplayForward?ligandId=4240 [glycine site], http://www.guidetopharmacology.org/GRAC/LigandDisplayForward?ligandId=4243 [glutamate site], http://www.guidetopharmacology.org/GRAC/LigandDisplayForward?ligandId=4332 [glutamate site] (GluN2D ≥ GluN2C > GluN2A ≥ GluN2B) [http://www.ncbi.nlm.nih.gov/pubmed/15801853?dopt=AbstractPlus], http://www.guidetopharmacology.org/GRAC/LigandDisplayForward?ligandId=4161 [glutamate site] (GluN2B > GluN2D = GluN2C = GluN2A), http://www.guidetopharmacology.org/GRAC/LigandDisplayForward?ligandId=4168 [glutamate site], http://www.guidetopharmacology.org/GRAC/LigandDisplayForward?ligandId=4170 [glutamate site] (GluN2A = GluN2B > GluN2C = GluN2D), http://www.guidetopharmacology.org/GRAC/LigandDisplayForward?ligandId=4155 [glutamate site]	
Channel blockers	http://www.guidetopharmacology.org/GRAC/LigandDisplayForward?ligandId=4282 (pIC_50_ 7.1) [http://www.ncbi.nlm.nih.gov/pubmed/17303642?dopt=AbstractPlus], http://www.guidetopharmacology.org/GRAC/LigandDisplayForward?ligandId=4233 (pIC_50_ 6.2) [http://www.ncbi.nlm.nih.gov/pubmed/17303642?dopt=AbstractPlus], http://www.guidetopharmacology.org/GRAC/LigandDisplayForward?ligandId=4128 (GluN2C = GluN2D ≥ GluN2B ≥ GluN2A) (pIC_50_ 4.7) [http://www.ncbi.nlm.nih.gov/pubmed/17303642?dopt=AbstractPlus], http://www.guidetopharmacology.org/GRAC/LigandDisplayForward?ligandId=708 (GluN2A = GluN2B > GluN2C = GluN2D), http://www.guidetopharmacology.org/GRAC/LigandDisplayForward?ligandId=4260 (GluN2A = GluN2B ≫ GluN2C = GluN2D), http://www.guidetopharmacology.org/GRAC/LigandDisplayForward?ligandId=2403	http://www.guidetopharmacology.org/GRAC/LigandDisplayForward?ligandId=708 (GluN2A = GluN2B > GluN2C = GluN2D), http://www.guidetopharmacology.org/GRAC/LigandDisplayForward?ligandId=4260 (GluN2A = GluN2B ≫ GluN2C = GluN2D), http://www.guidetopharmacology.org/GRAC/LigandDisplayForward?ligandId=4128 (GluN2C = GluN2D ≥ GluN2B ≥ GluN2A), http://www.guidetopharmacology.org/GRAC/LigandDisplayForward?ligandId=2403, http://www.guidetopharmacology.org/GRAC/LigandDisplayForward?ligandId=4233, http://www.guidetopharmacology.org/GRAC/LigandDisplayForward?ligandId=4282
Labelled ligands	http://www.guidetopharmacology.org/GRAC/LigandDisplayForward?ligandId=4078 [glutamate site] (Antagonist), http://www.guidetopharmacology.org/GRAC/LigandDisplayForward?ligandId=4079 [glycine site] (Antagonist), http://www.guidetopharmacology.org/GRAC/LigandDisplayForward?ligandId=4080 [glutamate site] (Antagonist), http://www.guidetopharmacology.org/GRAC/LigandDisplayForward?ligandId=4082 [glutamate site] (Antagonist), http://www.guidetopharmacology.org/GRAC/LigandDisplayForward?ligandId=4086 [glycine site] (Antagonist), http://www.guidetopharmacology.org/GRAC/LigandDisplayForward?ligandId=4088 [glycine site] (Antagonist), http://www.guidetopharmacology.org/GRAC/LigandDisplayForward?ligandId=4089 [cation channel] (Channel blocker), http://www.guidetopharmacology.org/GRAC/LigandDisplayForward?ligandId=4084 [glycine site] (Agonist)	http://www.guidetopharmacology.org/GRAC/LigandDisplayForward?ligandId=4078 [glutamate site] (Antagonist), http://www.guidetopharmacology.org/GRAC/LigandDisplayForward?ligandId=4079 [glycine site] (Antagonist), http://www.guidetopharmacology.org/GRAC/LigandDisplayForward?ligandId=4080 [glutamate site] (Antagonist), http://www.guidetopharmacology.org/GRAC/LigandDisplayForward?ligandId=4082 [glutamate site] (Antagonist), http://www.guidetopharmacology.org/GRAC/LigandDisplayForward?ligandId=4086 [glycine site] (Selective Antagonist), http://www.guidetopharmacology.org/GRAC/LigandDisplayForward?ligandId=4088 [glycine site] (Antagonist), http://www.guidetopharmacology.org/GRAC/LigandDisplayForward?ligandId=4089 [cation channel] (Channel blocker), http://www.guidetopharmacology.org/GRAC/LigandDisplayForward?ligandId=4084 [glycine site] (Agonist)

**Table:** 

Nomenclature	http://www.guidetopharmacology.org/GRAC/ObjectDisplayForward?objectId=460	http://www.guidetopharmacology.org/GRAC/ObjectDisplayForward?objectId=461
HGNC, UniProt	https://www.genenames.org/data/gene‐symbol‐report/#!/hgnc_id/HGNC:16767, http://www.uniprot.org/uniprot/Q8TCU5	https://www.genenames.org/data/gene‐symbol‐report/#!/hgnc_id/HGNC:16768, http://www.uniprot.org/uniprot/O60391
Comments	See the main comments section below for information on the pharmacology of GluN3A and GluN3B subunits	

### Comments

Potency orders unreferenced in the table are from [http://www.ncbi.nlm.nih.gov/pubmed/17962328?dopt=AbstractPlus, http://www.ncbi.nlm.nih.gov/pubmed/17303642?dopt=AbstractPlus, http://www.ncbi.nlm.nih.gov/pubmed/17622578?dopt=AbstractPlus, http://www.ncbi.nlm.nih.gov/pubmed/8642401?dopt=AbstractPlus, http://www.ncbi.nlm.nih.gov/pubmed/17088105?dopt=AbstractPlus, http://www.ncbi.nlm.nih.gov/pubmed/20716669?dopt=AbstractPlus]. In addition to the glutamate and http://www.guidetopharmacology.org/GRAC/LigandDisplayForward?ligandId=727 binding sites documented in the table, physiologically important inhibitory modulatory sites exist for Mg^2+^, Zn^2+^, and protons [http://www.ncbi.nlm.nih.gov/pubmed/15494561?dopt=AbstractPlus, http://www.ncbi.nlm.nih.gov/pubmed/10049997?dopt=AbstractPlus, http://www.ncbi.nlm.nih.gov/pubmed/20716669?dopt=AbstractPlus]. Voltage‐independent inhibition by Zn^2+^ binding with high affinity within the ATD is highly subunit selective (GluN2A ≫ GluN2B > GluN2C ≥ GluN2D; [http://www.ncbi.nlm.nih.gov/pubmed/17088105?dopt=AbstractPlus, http://www.ncbi.nlm.nih.gov/pubmed/20716669?dopt=AbstractPlus]). The receptor is also allosterically modulated, in both positive and negative directions, by endogenous neuroactive steroids in a subunit dependent manner [http://www.ncbi.nlm.nih.gov/pubmed/16257494?dopt=AbstractPlus, http://www.ncbi.nlm.nih.gov/pubmed/11861317?dopt=AbstractPlus]. Tonic proton blockade of NMDA receptor function is alleviated by polyamines and the inclusion of exon 5 within GluN1 subunit splice variants, whereas the non‐competitive antagonists http://www.guidetopharmacology.org/GRAC/LigandDisplayForward?ligandId=5472 and http://www.guidetopharmacology.org/GRAC/LigandDisplayForward?ligandId=4163 increase the fraction of receptors blocked by protons at ambient concentration. Inclusion of exon 5 also abolishes potentiation by polyamines and inhibition by Zn^2+^ that occurs through binding in the ATD [http://www.ncbi.nlm.nih.gov/pubmed/9698310?dopt=AbstractPlus]. http://www.guidetopharmacology.org/GRAC/LigandDisplayForward?ligandId=5472, http://www.guidetopharmacology.org/GRAC/LigandDisplayForward?ligandId=4163, http://www.guidetopharmacology.org/GRAC/LigandDisplayForward?ligandId=86, http://www.guidetopharmacology.org/GRAC/LigandDisplayForward?ligandId=5473 and http://www.guidetopharmacology.org/GRAC/LigandDisplayForward?ligandId=4300 discriminate between recombinant NMDA receptors assembled from GluN1 and either GluN2A, or GluN2B, subunits by acting as selective, non‐competitive, antagonists of heterooligomers incorporating GluN2B through a binding site at the ATD GluN1/GluN2B subunit interface [http://www.ncbi.nlm.nih.gov/pubmed/21677647?dopt=AbstractPlus]. http://www.guidetopharmacology.org/GRAC/LigandDisplayForward?ligandId=4244 is a competitive antagonist that also displays selectivity for GluN2B over GluN2A subunit‐containing receptors. Similarly, http://www.guidetopharmacology.org/GRAC/LigandDisplayForward?ligandId=5474 is a photoaffinity label that interacts selectively with receptors incorporating GluN2B versus GluN2A, GluN2D and, to a lesser extent, GluN2C subunits. TCN 201 and TCN 213 have recently been shown to block GluN2A NMDA receptors selectively by a mechanism that involves allosteric inhibition of glycine binding to the GluN1 site [http://www.ncbi.nlm.nih.gov/pubmed/20810618?dopt=AbstractPlus, http://www.ncbi.nlm.nih.gov/pubmed/22579927?dopt=AbstractPlus, http://www.ncbi.nlm.nih.gov/pubmed/22553026?dopt=AbstractPlus, http://www.ncbi.nlm.nih.gov/pubmed/22022974?dopt=AbstractPlus]. In addition to influencing the pharmacological profile of the NMDA receptor, the identity of the GluN2 subunit co‐assembled with GluN1 is an important determinant of biophysical properties that include sensitivity to block by Mg^2+^, single‐channel conductance and maximal open probablity and channel deactivation time [http://www.ncbi.nlm.nih.gov/pubmed/15494561?dopt=AbstractPlus, http://www.ncbi.nlm.nih.gov/pubmed/15701057?dopt=AbstractPlus, http://www.ncbi.nlm.nih.gov/pubmed/19404260?dopt=AbstractPlus]. Incorporation of the GluN3A subunit into tri‐heteromers containing GluN1 and GluN2 subunits is associated with decreased single‐channel conductance, reduced permeability to Ca^2+^ and decreased susceptibility to block by Mg^2+^ [http://www.ncbi.nlm.nih.gov/pubmed/18654865?dopt=AbstractPlus, http://www.ncbi.nlm.nih.gov/pubmed/20097255?dopt=AbstractPlus]. Reduced permeability to Ca^2+^ has also been observed following the inclusion of GluN3B in tri‐heteromers. The expression of GluN3A, or GluN3B, with GluN1 alone forms, in *Xenopus laevis* oocytes, a cation channel with unique properties that include activation by http://www.guidetopharmacology.org/GRAC/LigandDisplayForward?ligandId=727 (but not http://www.guidetopharmacology.org/GRAC/LigandDisplayForward?ligandId=4268), lack of permeation by Ca^2+^ and resistance to blockade by Mg^2+^ and NMDA receptor antagonists [http://www.ncbi.nlm.nih.gov/pubmed/11823786?dopt=AbstractPlus]. The function of heteromers composed of GluN1 and GluN3A is enhanced by Zn^2+^, or glycine site antagonists, binding to the GluN1 subunit [http://www.ncbi.nlm.nih.gov/pubmed/18711142?dopt=AbstractPlus]. Zn^2+^ also directly activates such complexes. The co‐expression of GluN1, GluN3A and GluN3B appears to be required to form glycine‐activated receptors in mammalian cell hosts [http://www.ncbi.nlm.nih.gov/pubmed/17502428?dopt=AbstractPlus].

### Further reading on Ionotropic glutamate receptors

Filippini A *et al.* (2017) The Good and the Bad of Glutamate Receptor RNA Editing. *Mol. Neurobiol.* **54**: 6795‐6805 https://www.ncbi.nlm.nih.gov/pubmed/27766534?dopt=AbstractPlus

Greger IH *et al.* (2017) Structural and Functional Architecture of AMPA‐Type Glutamate Receptors and Their Auxiliary Proteins. *Neuron* **94**: 713‐730 https://www.ncbi.nlm.nih.gov/pubmed/28521126?dopt=AbstractPlus

Hackos DH *et al.* (2017) Diverse modes of NMDA receptor positive allosteric modulation: Mechanisms and consequences. *Neuropharmacology* **112**: 34‐45 https://www.ncbi.nlm.nih.gov/pubmed/27484578?dopt=AbstractPlus

Huettner JE. (2015) Glutamate receptor pores. *J. Physiol. (Lond.)* **593**: 49‐59 https://www.ncbi.nlm.nih.gov/pubmed/25556787?dopt=AbstractPlus

Iacobucci GJ *et al.* (2017) NMDA receptors: linking physiological output to biophysical operation. *Nat. Rev. Neurosci.* **18**: 236‐249 https://www.ncbi.nlm.nih.gov/pubmed/28303017?dopt=AbstractPlus

Krieger J *et al.* (2015) Structure, Dynamics, and Allosteric Potential of Ionotropic Glutamate Receptor N‐Terminal Domains. *Biophys. J.* **109**: 1136‐48 https://www.ncbi.nlm.nih.gov/pubmed/26255587?dopt=AbstractPlus

Lussier MP *et al.* (2015) Dynamic Regulation of N‐Methyl‐d‐aspartate (NMDA) and α‐Amino‐3‐hydroxy‐5‐methyl‐4‐isoxazolepropionic Acid (AMPA) Receptors by Posttranslational Modifications. *J. Biol. Chem.* **290**: 28596‐603 https://www.ncbi.nlm.nih.gov/pubmed/26453298?dopt=AbstractPlus

Mllerud S *et al.* (2017) Lessons from crystal structures of kainate receptors. *Neuropharmacology* **112**: 16‐28 https://www.ncbi.nlm.nih.gov/pubmed/27236079?dopt=AbstractPlus

Yuzaki M *et al.* (2017) A GluD Coming‐Of‐Age Story. *Trends Neurosci.* **40**: 138‐150 https://www.ncbi.nlm.nih.gov/pubmed/28110935?dopt=AbstractPlus

Zhou HX *et al.* (2017) Advancing NMDA Receptor Physiology by Integrating Multiple Approaches. *Trends Neurosci.* **40**: 129‐137 https://www.ncbi.nlm.nih.gov/pubmed/28187950?dopt=AbstractPlus

Zhuo M. (2017) Ionotropic glutamate receptors contribute to pain transmission and chronic pain. *Neuropharmacology* **112**: 228‐234 https://www.ncbi.nlm.nih.gov/pubmed/27543416?dopt=AbstractPlus

## http://www.guidetopharmacology.org/GRAC/FamilyDisplayForward?familyId=123

### Overview

The inositol 1,4,5‐trisphosphate receptors (IP_3_R) are ligand‐gated Ca^2+^‐release channels on intracellular Ca^2^+ store sites (such as the endoplasmic reticulum). They are responsible for the mobilization of intracellular Ca^2+^ stores and play an important role in intracellular Ca^2+^ signalling in a wide variety of cell types. Three different gene products (types I‐III) have been isolated, which assemble as large tetrameric structures. IP_3_Rs are closely associated with certain proteins: http://www.guidetopharmacology.org/GRAC/LigandDisplayForward?ligandId=2351 (https://www.genenames.org/data/gene‐symbol‐report/#!/hgnc_id/HGNC:1442 https://www.genenames.org/data/gene‐symbol‐report/#!/hgnc_id/HGNC:1445 https://www.genenames.org/data/gene‐symbol‐report/#!/hgnc_id/HGNC:1449, http://www.uniprot.org/uniprot/P62158) and FKBP (and calcineurin via FKBP). They are phosphorylated by PKA, PKC, PKG and CaMKII.

**Table:** 

Nomenclature	http://www.guidetopharmacology.org/GRAC/ObjectDisplayForward?objectId=743	http://www.guidetopharmacology.org/GRAC/ObjectDisplayForward?objectId=744	http://www.guidetopharmacology.org/GRAC/ObjectDisplayForward?objectId=745
HGNC, UniProt	https://www.genenames.org/data/gene‐symbol‐report/#!/hgnc_id/HGNC:6180, http://www.uniprot.org/uniprot/Q14643	https://www.genenames.org/data/gene‐symbol‐report/#!/hgnc_id/HGNC:6181, http://www.uniprot.org/uniprot/Q14571	https://www.genenames.org/data/gene‐symbol‐report/#!/hgnc_id/HGNC:6182, http://www.uniprot.org/uniprot/Q14573
Endogenous activators	cytosolic http://www.guidetopharmacology.org/GRAC/LigandDisplayForward?ligandId=1713 (< mM range), cytosolic http://www.guidetopharmacology.org/GRAC/LigandDisplayForward?ligandId=707 Concentration range: <7.5×10^−4^M, http://www.guidetopharmacology.org/GRAC/LigandDisplayForward?ligandId=4222 (endogenous; nM ‐ *μ*M range)	cytosolic http://www.guidetopharmacology.org/GRAC/LigandDisplayForward?ligandId=707 (nM range), http://www.guidetopharmacology.org/GRAC/LigandDisplayForward?ligandId=4222 (endogenous; nM ‐ *μ*M range)	cytosolic http://www.guidetopharmacology.org/GRAC/LigandDisplayForward?ligandId=707 (nM range), http://www.guidetopharmacology.org/GRAC/LigandDisplayForward?ligandId=4222 (endogenous; nM ‐ *μ*M range)
Activators	http://www.guidetopharmacology.org/GRAC/LigandDisplayForward?ligandId=4124 (pharmacological; nM range), http://www.guidetopharmacology.org/GRAC/LigandDisplayForward?ligandId=4223 (pharmacological; also activated by other InsP_3_ analogues)	http://www.guidetopharmacology.org/GRAC/LigandDisplayForward?ligandId=4124 (pharmacological; nM range), http://www.guidetopharmacology.org/GRAC/LigandDisplayForward?ligandId=4223 (pharmacological; also activated by other InsP_3_ analogues)	–
Antagonists	http://www.guidetopharmacology.org/GRAC/LigandDisplayForward?ligandId=2387 (*μ*M range), http://www.guidetopharmacology.org/GRAC/LigandDisplayForward?ligandId=407 (mM range), http://www.guidetopharmacology.org/GRAC/LigandDisplayForward?ligandId=2459 (*μ*M range), http://www.guidetopharmacology.org/GRAC/LigandDisplayForward?ligandId=4344 (*μ*M range)	http://www.guidetopharmacology.org/GRAC/LigandDisplayForward?ligandId=2459 (*μ*M range)	http://www.guidetopharmacology.org/GRAC/LigandDisplayForward?ligandId=2459 (*μ*M range)
Functional Characteristics	Ca^2+^: (P_Ba_/P_K_ 6) single‐channel conductance 70 pS (50 mM Ca^2+^)	Ca^2+^: single‐channel conductance 70 pS (50 mM Ca^2+^) 390 pS (220 mM Cs^+^)	Ca^2+^: single‐channel conductance 88 pS (55 mM Ba^2+^)
Comments	IP_3_R1 is also antagonised by calmodulin at high cytosolic Ca^2+^ concentrations	–	–

### Comments

The absence of a modulator of a particular isoform of receptor indicates that the action of that modulator has not been determined, not that it is without effect.

### Further reading on IP_3_ receptors

Berridge MJ. (2016) The Inositol Trisphosphate/Calcium Signaling Pathway in Health and Disease. *Physiol. Rev.* **96**: 1261‐96 https://www.ncbi.nlm.nih.gov/pubmed/27512009?dopt=AbstractPlus

Garcia MI *et al.* (2017) Cardiac inositol 1,4,5‐trisphosphate receptors. *Biochim. Biophys. Acta* **1864**: 907‐914 https://www.ncbi.nlm.nih.gov/pubmed/27884701?dopt=AbstractPlus

Mak DO *et al.* (2015) Inositol 1,4,5‐trisphosphate receptors in the endoplasmic reticulum: A single‐channel point of view. *Cell Calcium* **58**: 67‐78 https://www.ncbi.nlm.nih.gov/pubmed/25555684?dopt=AbstractPlus

Rossi AM *et al.* (2018) IP_3 receptors ‐ lessons from analyses *ex cellula*. *J. Cell. Sci.* **132**: https://www.ncbi.nlm.nih.gov/pubmed/30552138?dopt=AbstractPlus

Seo MD *et al.* (2015) Structural insights into endoplasmic reticulum stored calcium regulation by inositol 1,4,5‐trisphosphate and ryanodine receptors. *Biochim. Biophys. Acta* **1853**: 1980‐91 https://www.ncbi.nlm.nih.gov/pubmed/25461839?dopt=AbstractPlus

Thillaiappan NB *et al.* (2019) IP_3 receptors and Ca^2+^ entry. *Biochim Biophys Acta Mol Cell Res* **1866**: 1092‐1100 https://www.ncbi.nlm.nih.gov/pubmed/30448464?dopt=AbstractPlus

## http://www.guidetopharmacology.org/GRAC/FamilyDisplayForward?familyId=76

### Overview

Nicotinic acetylcholine receptors are members of the Cys‐loop family of transmitter‐gated ion channels that includes the GABA_A_, strychnine‐sensitive glycine and 5‐HT_3_ receptors [http://www.ncbi.nlm.nih.gov/pubmed/19126755?dopt=AbstractPlus, http://www.ncbi.nlm.nih.gov/pubmed/18723036?dopt=AbstractPlus, http://www.ncbi.nlm.nih.gov/pubmed/16554804?dopt=AbstractPlus, http://www.ncbi.nlm.nih.gov/pubmed/19721446?dopt=AbstractPlus, http://www.ncbi.nlm.nih.gov/pubmed/21787755?dopt=AbstractPlus]. All nicotinic receptors are pentamers in which each of the five subunits contains four α‐helical transmembrane domains. Genes encoding a total of 17 subunits (α1‐10, β1‐4, γ, *δ* and *ϵ*) have been identified [http://www.ncbi.nlm.nih.gov/pubmed/17651090?dopt=AbstractPlus]. All subunits with the exception of α8 (present in avian species) have been identified in mammals. All α subunits possess two tandem cysteine residues near to the site involved in acetylcholine binding, and subunits not named α lack these residues [http://www.ncbi.nlm.nih.gov/pubmed/18723036?dopt=AbstractPlus]. The orthosteric ligand binding site is formed by residues within at least three peptide domains on the α subunit (principal component), and three on the adjacent subunit (complementary component). nAChRs contain several allosteric modulatory sites. One such site, for positive allosteric modulators (PAMs) and allosteric agonists, has been proposed to reside within an intrasubunit cavity between the four transmembrane domains [http://www.ncbi.nlm.nih.gov/pubmed/21436053?dopt=AbstractPlus, http://www.ncbi.nlm.nih.gov/pubmed/18791069?dopt=AbstractPlus]; see also [http://www.ncbi.nlm.nih.gov/pubmed/21572436?dopt=AbstractPlus]). The high resolution crystal structure of the molluscan acetylcholine binding protein, a structural homologue of the extracellular binding domain of a nicotinic receptor pentamer, in complex with several nicotinic receptor ligands (*e.g.*[http://www.ncbi.nlm.nih.gov/pubmed/15046723?dopt=AbstractPlus]) and the crystal structure of the extracellular domain of the α1 subunit bound to http://www.guidetopharmacology.org/GRAC/LigandDisplayForward?ligandId=3964 at 1.94 Å resolution [http://www.ncbi.nlm.nih.gov/pubmed/17643119?dopt=AbstractPlus], has revealed the orthosteric binding site in detail (reviewed in [http://www.ncbi.nlm.nih.gov/pubmed/18262468?dopt=AbstractPlus, http://www.ncbi.nlm.nih.gov/pubmed/17651090?dopt=AbstractPlus, http://www.ncbi.nlm.nih.gov/pubmed/19576182?dopt=AbstractPlus, http://www.ncbi.nlm.nih.gov/pubmed/16554804?dopt=AbstractPlus]). Nicotinic receptors at the somatic neuromuscular junction of adult animals have the stoichiometry (α1)_2_β1*δϵ*, whereas an extrajunctional (α1)_2_β1γ*δ* receptor predominates in embryonic and denervated skeletal muscle and other pathological states. Other nicotinic receptors are assembled as combinations of α(2‐6) and β(2‐4) subunits. For α2, α3, α4 and β2 and β4 subunits, pairwise combinations of α and β (*e.g.* α3β4 and α4β2) are sufficient to form a functional receptor *in vitro*, but far more complex isoforms may exist *in vivo* (reviewed in [http://www.ncbi.nlm.nih.gov/pubmed/19481063?dopt=AbstractPlus, http://www.ncbi.nlm.nih.gov/pubmed/16876883?dopt=AbstractPlus, http://www.ncbi.nlm.nih.gov/pubmed/18723036?dopt=AbstractPlus]). There is strong evidence that the pairwise assembly of some α and β subunits can occur with variable stoichiometry [*e.g.* (α4)_2_(β2)_2_ or (α4)_3_(β2)_2_] which influences the biophysical and pharmacological properties of the receptor [http://www.ncbi.nlm.nih.gov/pubmed/18723036?dopt=AbstractPlus]. α5 and β3 subunits lack function when expressed alone, or pairwise, but participate in the formation of functional hetero‐oligomeric receptors when expressed as a third subunit with another α and β pair [e.g. α4α5αβ2, α4αβ2β3, α5α6β2, see [http://www.ncbi.nlm.nih.gov/pubmed/18723036?dopt=AbstractPlus] for further examples]. The α6 subunit can form a functional receptor when co‐expressed with β4 *in vitro*, but more efficient expression ensues from incorporation of a third partner, such as β3 [http://www.ncbi.nlm.nih.gov/pubmed/19498417?dopt=AbstractPlus]. The α7, α8, and α9 subunits form functional homo‐oligomers, but can also combine with a second subunit to constitute a hetero‐oligomeric assembly (*e.g.* α7β2 and α9α10). For functional expression of the α10 subunit, co‐assembly with α9 is necessary. The latter, along with the α10 subunit, appears to be largely confined to cochlear and vestibular hair cells. Comprehensive listings of nicotinic receptor subunit combinations identified from recombinant expression systems, or *in vivo*, are given in [http://www.ncbi.nlm.nih.gov/pubmed/18723036?dopt=AbstractPlus]. In addition, numerous proteins interact with nicotinic ACh receptors modifying their assembly, trafficking to and from the cell surface, and activation by ACh (reviewed by [http://www.ncbi.nlm.nih.gov/pubmed/20346921?dopt=AbstractPlus, http://www.ncbi.nlm.nih.gov/pubmed/20674046?dopt=AbstractPlus, http://www.ncbi.nlm.nih.gov/pubmed/18246096?dopt=AbstractPlus]).

The nicotinic receptor Subcommittee of **NC‐IUPHAR** has recommended a nomenclature and classification scheme for nicotinic acetylcholine (nACh) receptors based on the subunit composition of known, naturally‐ and/or heterologously‐expressed nACh receptor subtypes [http://www.ncbi.nlm.nih.gov/pubmed/10353988?dopt=AbstractPlus]. Headings for this table reflect abbreviations designating nACh receptor subtypes based on the predominant α subunit contained in that receptor subtype. An asterisk following the indicated α subunit denotes that other subunits are known to, or may, assemble with the indicated α subunit to form the designated nACh receptor subtype(s). Where subunit stoichiometries within a specific nACh receptor subtype are known, numbers of a particular subunit larger than 1 are indicated by a subscript following the subunit (enclosed in parentheses – see also [http://www.ncbi.nlm.nih.gov/pubmed/18655795?dopt=AbstractPlus]).

**Table:** 

Nomenclature	http://www.guidetopharmacology.org/GRAC/ObjectDisplayForward?objectId=462	http://www.guidetopharmacology.org/GRAC/ObjectDisplayForward?objectId=463	http://www.guidetopharmacology.org/GRAC/ObjectDisplayForward?objectId=464	http://www.guidetopharmacology.org/GRAC/ObjectDisplayForward?objectId=465
HGNC, UniProt	https://www.genenames.org/data/gene‐symbol‐report/#!/hgnc_id/HGNC:1955, http://www.uniprot.org/uniprot/P02708	https://www.genenames.org/data/gene‐symbol‐report/#!/hgnc_id/HGNC:1956, http://www.uniprot.org/uniprot/Q15822	https://www.genenames.org/data/gene‐symbol‐report/#!/hgnc_id/HGNC:1957, http://www.uniprot.org/uniprot/P32297	https://www.genenames.org/data/gene‐symbol‐report/#!/hgnc_id/HGNC:1958, http://www.uniprot.org/uniprot/P43681
Commonly used antagonists	(α1)_2_β1γ*δ* and (α1)_2_β1*δϵ*: http://www.guidetopharmacology.org/GRAC/LigandDisplayForward?ligandId=3964‐http://www.guidetopharmacology.org/GRAC/LigandDisplayForward?ligandId=3964 > http://www.guidetopharmacology.org/GRAC/LigandDisplayForward?ligandId=4001 > http://www.guidetopharmacology.org/GRAC/LigandDisplayForward?ligandId=4002 > http://www.guidetopharmacology.org/GRAC/LigandDisplayForward?ligandId=4003 > http://www.guidetopharmacology.org/GRAC/LigandDisplayForward?ligandId=2294 (IC_50_ = 43 ‐ 82 nM)	α2β2: http://www.guidetopharmacology.org/GRAC/LigandDisplayForward?ligandId=4006 (*K_B_* = 0.9 *μ*M), http://www.guidetopharmacology.org/GRAC/LigandDisplayForward?ligandId=2294 (*K_B_* = 1.4 *μ*M); α2β4: http://www.guidetopharmacology.org/GRAC/LigandDisplayForward?ligandId=4006 (*K_B_* = 3.6 *μ*M), http://www.guidetopharmacology.org/GRAC/LigandDisplayForward?ligandId=2294 (*K_B_* = 4.2 *μ*M)	α3β2: http://www.guidetopharmacology.org/GRAC/LigandDisplayForward?ligandId=4006 (*K_B_* = 1.6 *μ*M, IC_50_ = 2.0 *μ*M), http://www.guidetopharmacology.org/GRAC/LigandDisplayForward?ligandId=2294 (*K_B_* = 2.4 *μ*M); α3β4: http://www.guidetopharmacology.org/GRAC/LigandDisplayForward?ligandId=4006 (*K_B_* = 19 *μ*M, IC_50_ = 26 *μ*M), http://www.guidetopharmacology.org/GRAC/LigandDisplayForward?ligandId=2294 (*K_B_* = 2.2 *μ*M)	α4β2: http://www.guidetopharmacology.org/GRAC/LigandDisplayForward?ligandId=4006 (*K_B_* = 0.1 *μ*M; IC_50_ = 0.08 ‐ 0.9 *μ*M), http://www.guidetopharmacology.org/GRAC/LigandDisplayForward?ligandId=2294 (*K_B_* = 3.2 *μ*M, IC_50_ = 34 *μ*M); α4β4: http://www.guidetopharmacology.org/GRAC/LigandDisplayForward?ligandId=4006 (*K_B_* = 0.01 *μ*M, IC_50_ = 0.19 – 1.2 *μ*M), http://www.guidetopharmacology.org/GRAC/LigandDisplayForward?ligandId=2294 (*K_B_* = 0.2 *μ*M, IC_50_ = 50 *μ*M)
Selective agonists	http://www.guidetopharmacology.org/GRAC/LigandDisplayForward?ligandId=4004 (selective for (α1)_2_β1γ*δ*)	–	–	http://www.guidetopharmacology.org/GRAC/LigandDisplayForward?ligandId=5459 [http://www.ncbi.nlm.nih.gov/pubmed/16171993?dopt=AbstractPlus], http://www.guidetopharmacology.org/GRAC/LigandDisplayForward?ligandId=3994 [http://www.ncbi.nlm.nih.gov/pubmed/14698195?dopt=AbstractPlus], http://www.guidetopharmacology.org/GRAC/LigandDisplayForward?ligandId=3987 (α4β2) [http://www.ncbi.nlm.nih.gov/pubmed/12871651?dopt=AbstractPlus]
Selective antagonists	http://www.guidetopharmacology.org/GRAC/LigandDisplayForward?ligandId=3964, http://www.guidetopharmacology.org/GRAC/LigandDisplayForward?ligandId=3983, http://www.guidetopharmacology.org/GRAC/LigandDisplayForward?ligandId=3999, http://www.guidetopharmacology.org/GRAC/LigandDisplayForward?ligandId=4001, http://www.guidetopharmacology.org/GRAC/LigandDisplayForward?ligandId=3984 (selective for (α1)_2_β1*δϵ*)	–	http://www.guidetopharmacology.org/GRAC/LigandDisplayForward?ligandId=3973 (α3β4), http://www.guidetopharmacology.org/GRAC/LigandDisplayForward?ligandId=3970 (α3β2), http://www.guidetopharmacology.org/GRAC/LigandDisplayForward?ligandId=3982 (α3β2), http://www.guidetopharmacology.org/GRAC/LigandDisplayForward?ligandId=4011‐http://www.guidetopharmacology.org/GRAC/LigandDisplayForward?ligandId=4011 (α3β2), http://www.guidetopharmacology.org/GRAC/LigandDisplayForward?ligandId=4009‐http://www.guidetopharmacology.org/GRAC/LigandDisplayForward?ligandId=4009 (α3β2)	–
Channel blockers	http://www.guidetopharmacology.org/GRAC/LigandDisplayForward?ligandId=356 ((α1)_2_β1γ*δ* and (α1)_2_β1*δϵ*) (pIC_50_ ∼6), http://www.guidetopharmacology.org/GRAC/LigandDisplayForward?ligandId=3990 ((α1)_2_β1*δ*) (pIC_50_ ∼5.8)	http://www.guidetopharmacology.org/GRAC/LigandDisplayForward?ligandId=3963, http://www.guidetopharmacology.org/GRAC/LigandDisplayForward?ligandId=3990	http://www.guidetopharmacology.org/GRAC/LigandDisplayForward?ligandId=3990 (α3β4) (pIC_50_ 6.4), http://www.guidetopharmacology.org/GRAC/LigandDisplayForward?ligandId=3990 (α3β2) (pIC_50_ 5.1), http://www.guidetopharmacology.org/GRAC/LigandDisplayForward?ligandId=3986 (α3β4) [http://www.ncbi.nlm.nih.gov/pubmed/19389923?dopt=AbstractPlus], http://www.guidetopharmacology.org/GRAC/LigandDisplayForward?ligandId=4000 (α3β4) [http://www.ncbi.nlm.nih.gov/pubmed/17625074?dopt=AbstractPlus], http://www.guidetopharmacology.org/GRAC/LigandDisplayForward?ligandId=3963 (α3β2), http://www.guidetopharmacology.org/GRAC/LigandDisplayForward?ligandId=3963 (α3β4)	http://www.guidetopharmacology.org/GRAC/LigandDisplayForward?ligandId=3990 (α4β4) (pIC_50_ 5.3–6.5), http://www.guidetopharmacology.org/GRAC/LigandDisplayForward?ligandId=3990 (α4β2) (pIC_50_ 5.4–5.4), http://www.guidetopharmacology.org/GRAC/LigandDisplayForward?ligandId=3963 (α4β2) (pIC_50_ 4.5–5.2), http://www.guidetopharmacology.org/GRAC/LigandDisplayForward?ligandId=3963 (α4β4) (pIC_50_ 4), http://www.guidetopharmacology.org/GRAC/LigandDisplayForward?ligandId=3986 (α4β2) [http://www.ncbi.nlm.nih.gov/pubmed/19389923?dopt=AbstractPlus], http://www.guidetopharmacology.org/GRAC/LigandDisplayForward?ligandId=4000 (α4β2) [http://www.ncbi.nlm.nih.gov/pubmed/17625074?dopt=AbstractPlus]
Allosteric modulators	–	http://www.guidetopharmacology.org/GRAC/LigandDisplayForward?ligandId=3992 (Positive) [http://www.ncbi.nlm.nih.gov/pubmed/16738207?dopt=AbstractPlus]	–	http://www.guidetopharmacology.org/GRAC/LigandDisplayForward?ligandId=3992 (Positive) [http://www.ncbi.nlm.nih.gov/pubmed/16738207?dopt=AbstractPlus]
Selective allosteric modulators	–	–	–	http://www.guidetopharmacology.org/GRAC/LigandDisplayForward?ligandId=3993 (Positive) [http://www.ncbi.nlm.nih.gov/pubmed/21763685?dopt=AbstractPlus]
Labelled ligands	http://www.guidetopharmacology.org/GRAC/LigandDisplayForward?ligandId=3972 (Selective Antagonist), http://www.guidetopharmacology.org/GRAC/LigandDisplayForward?ligandId=3981 (Selective Antagonist)	http://www.guidetopharmacology.org/GRAC/LigandDisplayForward?ligandId=3976 (Agonist), http://www.guidetopharmacology.org/GRAC/LigandDisplayForward?ligandId=3977 (Agonist), http://www.guidetopharmacology.org/GRAC/LigandDisplayForward?ligandId=3979 (Agonist)	http://www.guidetopharmacology.org/GRAC/LigandDisplayForward?ligandId=3976 (Agonist), http://www.guidetopharmacology.org/GRAC/LigandDisplayForward?ligandId=3977 (Agonist), http://www.guidetopharmacology.org/GRAC/LigandDisplayForward?ligandId=3976 (Agonist), http://www.guidetopharmacology.org/GRAC/LigandDisplayForward?ligandId=3977 (Agonist), http://www.guidetopharmacology.org/GRAC/LigandDisplayForward?ligandId=3979 (Agonist)	http://www.guidetopharmacology.org/GRAC/LigandDisplayForward?ligandId=3976 (Agonist), http://www.guidetopharmacology.org/GRAC/LigandDisplayForward?ligandId=3977 (Agonist), http://www.guidetopharmacology.org/GRAC/LigandDisplayForward?ligandId=3979 (Agonist), http://www.guidetopharmacology.org/GRAC/LigandDisplayForward?ligandId=3976 (Agonist), http://www.guidetopharmacology.org/GRAC/LigandDisplayForward?ligandId=3977 (Agonist), http://www.guidetopharmacology.org/GRAC/LigandDisplayForward?ligandId=3977 (Agonist) – Rat, http://www.guidetopharmacology.org/GRAC/LigandDisplayForward?ligandId=3979 (Agonist)
Functional Characteristics	(α1)_2_βγ*δ*: P_Ca_/P_Na_ = 0.16 ‐ 0.2, *P*_f_ = 2.1 – 2.9%; (α1)_2_β*δϵ*: P_Ca_/P_Na_ = 0.65 – 1.38, *P*_f_ = 4.1 – 7.2%	α2β2: P_Ca_/P_Na_ 1.5	α3β2: P_Ca_/P_Na_ = 1.5; α3β4: P_Ca_/P_Na_ = 0.78 ‐ 1.1, *P*_f_ = 2.7 – 4.6%	α4β2: P_Ca_/P_Na_ = 1.65, *P*_f_ = 2.6 – 2.9%; α4β4: *P*_f_ = 1.5 – 3.0 %

**Table:** 

Nomenclature	http://www.guidetopharmacology.org/GRAC/ObjectDisplayForward?objectId=466	http://www.guidetopharmacology.org/GRAC/ObjectDisplayForward?objectId=467	http://www.guidetopharmacology.org/GRAC/ObjectDisplayForward?objectId=468
HGNC, UniProt	https://www.genenames.org/data/gene‐symbol‐report/#!/hgnc_id/HGNC:1959, http://www.uniprot.org/uniprot/P30532	https://www.genenames.org/data/gene‐symbol‐report/#!/hgnc_id/HGNC:15963, http://www.uniprot.org/uniprot/Q15825	https://www.genenames.org/data/gene‐symbol‐report/#!/hgnc_id/HGNC:1960, http://www.uniprot.org/uniprot/P36544
Commonly used antagonists	–	α6/α3β2β3 chimera: http://www.guidetopharmacology.org/GRAC/LigandDisplayForward?ligandId=4006 (IC_50_ = 1.1 *μ*M)	(α7)5: http://www.guidetopharmacology.org/GRAC/LigandDisplayForward?ligandId=4006 (IC_50_ = 8 ‐ 20 *μ*M); (α7)5: http://www.guidetopharmacology.org/GRAC/LigandDisplayForward?ligandId=2294 (IC_50_ = 3.1 *μ*M)
Selective agonists	–	–	http://www.guidetopharmacology.org/GRAC/LigandDisplayForward?ligandId=6926 (Partial agonist) [http://www.ncbi.nlm.nih.gov/pubmed/21919481?dopt=AbstractPlus, http://www.ncbi.nlm.nih.gov/pubmed/15293871?dopt=AbstractPlus], http://www.guidetopharmacology.org/GRAC/LigandDisplayForward?ligandId=7371 ([125I]α‐ bungarotoxin binding assay) [411], http://www.guidetopharmacology.org/GRAC/LigandDisplayForward?ligandId=3962 (allosteric) [http://www.ncbi.nlm.nih.gov/pubmed/21436053?dopt=AbstractPlus], http://www.guidetopharmacology.org/GRAC/LigandDisplayForward?ligandId=3995 ((α7)_5_) [http://www.ncbi.nlm.nih.gov/pubmed/17898229?dopt=AbstractPlus], http://www.guidetopharmacology.org/GRAC/LigandDisplayForward?ligandId=3998 ((α7)_5_) [http://www.ncbi.nlm.nih.gov/pubmed/16821801?dopt=AbstractPlus], http://www.guidetopharmacology.org/GRAC/LigandDisplayForward?ligandId=3997 ((α7)_5_) [http://www.ncbi.nlm.nih.gov/pubmed/18490160?dopt=AbstractPlus], http://www.guidetopharmacology.org/GRAC/LigandDisplayForward?ligandId=3988 ((α7)_5_) [http://www.ncbi.nlm.nih.gov/pubmed/15715459?dopt=AbstractPlus], http://www.guidetopharmacology.org/GRAC/LigandDisplayForward?ligandId=3969 ((α7)_5_) [http://www.ncbi.nlm.nih.gov/pubmed/19482012?dopt=AbstractPlus]
Selective antagonists	http://www.guidetopharmacology.org/GRAC/LigandDisplayForward?ligandId=3970, http://www.guidetopharmacology.org/GRAC/LigandDisplayForward?ligandId=3982, http://www.guidetopharmacology.org/GRAC/LigandDisplayForward?ligandId=4011, http://www.guidetopharmacology.org/GRAC/LigandDisplayForward?ligandId=4009	http://www.guidetopharmacology.org/GRAC/LigandDisplayForward?ligandId=3970 (α6β2*), http://www.guidetopharmacology.org/GRAC/LigandDisplayForward?ligandId=4010 (α6β2β3), http://www.guidetopharmacology.org/GRAC/LigandDisplayForward?ligandId=3985 (α6/α3β2β3 chimera)	http://www.guidetopharmacology.org/GRAC/LigandDisplayForward?ligandId=3964 ((α7)_5_), http://www.guidetopharmacology.org/GRAC/LigandDisplayForward?ligandId=4007 ((α7)_5_), http://www.guidetopharmacology.org/GRAC/LigandDisplayForward?ligandId=3965 ((α7)_5_), http://www.guidetopharmacology.org/GRAC/LigandDisplayForward?ligandId=4005 ((α7)_5_)
Channel blockers	–	http://www.guidetopharmacology.org/GRAC/LigandDisplayForward?ligandId=3990 (α6/α3β2β3 chimera) (pIC_50_ 5), http://www.guidetopharmacology.org/GRAC/LigandDisplayForward?ligandId=3963 (α6/α3β2β3 chimera) (pIC_50_ 4)	http://www.guidetopharmacology.org/GRAC/LigandDisplayForward?ligandId=3990 ((α7)_5_) (pIC_50_ 4.8)
Allosteric modulators	–	–	http://www.guidetopharmacology.org/GRAC/LigandDisplayForward?ligandId=3986 (Positive) [http://www.ncbi.nlm.nih.gov/pubmed/19389923?dopt=AbstractPlus], http://www.guidetopharmacology.org/GRAC/LigandDisplayForward?ligandId=3992 (Positive) [http://www.ncbi.nlm.nih.gov/pubmed/16738207?dopt=AbstractPlus], http://www.guidetopharmacology.org/GRAC/LigandDisplayForward?ligandId=4000 (Positive) [http://www.ncbi.nlm.nih.gov/pubmed/17625074?dopt=AbstractPlus]
Selective allosteric modulators	–	–	http://www.guidetopharmacology.org/GRAC/LigandDisplayForward?ligandId=3966 (Positive) [http://www.ncbi.nlm.nih.gov/pubmed/21084390?dopt=AbstractPlus], http://www.guidetopharmacology.org/GRAC/LigandDisplayForward?ligandId=3991 (Positive) [http://www.ncbi.nlm.nih.gov/pubmed/15858066?dopt=AbstractPlus]
Labelled ligands	–	http://www.guidetopharmacology.org/GRAC/LigandDisplayForward?ligandId=3977 (Agonist) – Chicken, http://www.guidetopharmacology.org/GRAC/LigandDisplayForward?ligandId=3975‐http://www.guidetopharmacology.org/GRAC/LigandDisplayForward?ligandId=3975 (Antagonist)	http://www.guidetopharmacology.org/GRAC/LigandDisplayForward?ligandId=3977 (Agonist), http://www.guidetopharmacology.org/GRAC/LigandDisplayForward?ligandId=3974 (Agonist) [http://www.ncbi.nlm.nih.gov/pubmed/17959745?dopt=AbstractPlus], http://www.guidetopharmacology.org/GRAC/LigandDisplayForward?ligandId=3971 (Agonist) [http://www.ncbi.nlm.nih.gov/pubmed/20674564?dopt=AbstractPlus], http://www.guidetopharmacology.org/GRAC/LigandDisplayForward?ligandId=3972 (Selective Antagonist) (p*K*_d_ 8.3–9.1), http://www.guidetopharmacology.org/GRAC/LigandDisplayForward?ligandId=3981 (Selective Antagonist) (p*K*_d_ 8.3–9.1), http://www.guidetopharmacology.org/GRAC/LigandDisplayForward?ligandId=3980 (Antagonist) (p*K*_d_ 8.7) – Rat
Functional Characteristics	–	–	P_Ca_/P_Na_ = 6.6‐20, *P*_f_ = 8.8 ‐ 11.4%

**Table:** 

Nomenclature	http://www.guidetopharmacology.org/GRAC/ObjectDisplayForward?objectId=752	http://www.guidetopharmacology.org/GRAC/ObjectDisplayForward?objectId=469	http://www.guidetopharmacology.org/GRAC/ObjectDisplayForward?objectId=470
HGNC, UniProt	–	https://www.genenames.org/data/gene‐symbol‐report/#!/hgnc_id/HGNC:14079, http://www.uniprot.org/uniprot/Q9UGM1	https://www.genenames.org/data/gene‐symbol‐report/#!/hgnc_id/HGNC:13800, http://www.uniprot.org/uniprot/Q9GZZ6
Commonly used antagonists	(α8)_5_: http://www.guidetopharmacology.org/GRAC/LigandDisplayForward?ligandId=3964 > http://www.guidetopharmacology.org/GRAC/LigandDisplayForward?ligandId=320 ≥ http://www.guidetopharmacology.org/GRAC/LigandDisplayForward?ligandId=2294 ≥ http://www.guidetopharmacology.org/GRAC/LigandDisplayForward?ligandId=347	(α9)_5_: http://www.guidetopharmacology.org/GRAC/LigandDisplayForward?ligandId=3964 > http://www.guidetopharmacology.org/GRAC/LigandDisplayForward?ligandId=4005 > http://www.guidetopharmacology.org/GRAC/LigandDisplayForward?ligandId=347 http://www.guidetopharmacology.org/GRAC/LigandDisplayForward?ligandId=260 > http://www.guidetopharmacology.org/GRAC/LigandDisplayForward?ligandId=2294; α9α10: http://www.guidetopharmacology.org/GRAC/LigandDisplayForward?ligandId=3964 > http://www.guidetopharmacology.org/GRAC/LigandDisplayForward?ligandId=260 = http://www.guidetopharmacology.org/GRAC/LigandDisplayForward?ligandId=347 > http://www.guidetopharmacology.org/GRAC/LigandDisplayForward?ligandId=2294	α9α10: http://www.guidetopharmacology.org/GRAC/LigandDisplayForward?ligandId=3964 > http://www.guidetopharmacology.org/GRAC/LigandDisplayForward?ligandId=260 = http://www.guidetopharmacology.org/GRAC/LigandDisplayForward?ligandId=347 > http://www.guidetopharmacology.org/GRAC/LigandDisplayForward?ligandId=2294
Selective antagonists	–	http://www.guidetopharmacology.org/GRAC/LigandDisplayForward?ligandId=3964 ((α9)_5_), http://www.guidetopharmacology.org/GRAC/LigandDisplayForward?ligandId=3964 (α9α10), http://www.guidetopharmacology.org/GRAC/LigandDisplayForward?ligandId=4008 (α9α10), http://www.guidetopharmacology.org/GRAC/LigandDisplayForward?ligandId=3996 ((α9)_5_), http://www.guidetopharmacology.org/GRAC/LigandDisplayForward?ligandId=3996 (α9α10), http://www.guidetopharmacology.org/GRAC/LigandDisplayForward?ligandId=2585 ((α9)_5_), http://www.guidetopharmacology.org/GRAC/LigandDisplayForward?ligandId=2585 (α9α10), http://www.guidetopharmacology.org/GRAC/LigandDisplayForward?ligandId=347 ((α9)_5_), http://www.guidetopharmacology.org/GRAC/LigandDisplayForward?ligandId=347 (α9α10)	http://www.guidetopharmacology.org/GRAC/LigandDisplayForward?ligandId=3964 (α9α10), http://www.guidetopharmacology.org/GRAC/LigandDisplayForward?ligandId=4008 (α9α10), http://www.guidetopharmacology.org/GRAC/LigandDisplayForward?ligandId=3996 (α9α10), http://www.guidetopharmacology.org/GRAC/LigandDisplayForward?ligandId=2585 (α9α10), http://www.guidetopharmacology.org/GRAC/LigandDisplayForward?ligandId=347 (α9α10)
Labelled ligands	http://www.guidetopharmacology.org/GRAC/LigandDisplayForward?ligandId=3977 ((α8)_5_) (p*K*_d_ 9.7), http://www.guidetopharmacology.org/GRAC/LigandDisplayForward?ligandId=3972 (native α8*) (p*K*_d_ 8.3), http://www.guidetopharmacology.org/GRAC/LigandDisplayForward?ligandId=3981 (native α8*) (p*K*_d_ 8.3)	http://www.guidetopharmacology.org/GRAC/LigandDisplayForward?ligandId=3980 (Antagonist) (p*K*_d_ 8.1), http://www.guidetopharmacology.org/GRAC/LigandDisplayForward?ligandId=3972 (Antagonist), http://www.guidetopharmacology.org/GRAC/LigandDisplayForward?ligandId=3981 (Antagonist)	http://www.guidetopharmacology.org/GRAC/LigandDisplayForward?ligandId=3980 (Antagonist) (p*K*_d_ 8.1)
Functional Characteristics	–	(α9)_5_: P_Ca_/P_Na_ = 9; α9α10: P_Ca_/P_Na_ = 9, *P*_f_ = 22%	α9α10: P_Ca_/P_Na_ = 9, *P*_f_ = 22%

**Table:** 

Nomenclature	http://www.guidetopharmacology.org/GRAC/ObjectDisplayForward?objectId=471 http://www.guidetopharmacology.org/GRAC/ObjectDisplayForward?objectId=471	http://www.guidetopharmacology.org/GRAC/ObjectDisplayForward?objectId=471 http://www.guidetopharmacology.org/GRAC/ObjectDisplayForward?objectId=472	http://www.guidetopharmacology.org/GRAC/ObjectDisplayForward?objectId=471 http://www.guidetopharmacology.org/GRAC/ObjectDisplayForward?objectId=473	http://www.guidetopharmacology.org/GRAC/ObjectDisplayForward?objectId=471 http://www.guidetopharmacology.org/GRAC/ObjectDisplayForward?objectId=474	http://www.guidetopharmacology.org/GRAC/ObjectDisplayForward?objectId=471 http://www.guidetopharmacology.org/GRAC/ObjectDisplayForward?objectId=475	http://www.guidetopharmacology.org/GRAC/ObjectDisplayForward?objectId=471 http://www.guidetopharmacology.org/GRAC/ObjectDisplayForward?objectId=476	http://www.guidetopharmacology.org/GRAC/ObjectDisplayForward?objectId=471 http://www.guidetopharmacology.org/GRAC/ObjectDisplayForward?objectId=477
HGNC, UniProt	https://www.genenames.org/data/gene‐symbol‐report/#!/hgnc_id/HGNC:1961, http://www.uniprot.org/uniprot/P11230	https://www.genenames.org/data/gene‐symbol‐report/#!/hgnc_id/HGNC:1962, http://www.uniprot.org/uniprot/P17787	https://www.genenames.org/data/gene‐symbol‐report/#!/hgnc_id/HGNC:1963, http://www.uniprot.org/uniprot/Q05901	https://www.genenames.org/data/gene‐symbol‐report/#!/hgnc_id/HGNC:1964, http://www.uniprot.org/uniprot/P30926	https://www.genenames.org/data/gene‐symbol‐report/#!/hgnc_id/HGNC:1967, http://www.uniprot.org/uniprot/P07510	https://www.genenames.org/data/gene‐symbol‐report/#!/hgnc_id/HGNC:1965, http://www.uniprot.org/uniprot/Q07001	https://www.genenames.org/data/gene‐symbol‐report/#!/hgnc_id/HGNC:1966, http://www.uniprot.org/uniprot/Q04844
Antagonists	–	–	–	–	–	http://www.guidetopharmacology.org/GRAC/LigandDisplayForward?ligandId=8883 (pIC_50_ 6.2–6.3) [http://www.ncbi.nlm.nih.gov/pubmed/11934578?dopt=AbstractPlus]	–
Comments	Ligand interaction data for hetero‐oligomeric receptors containing the β1 subunit are listed under the α1 subunits						

### Comments

Commonly used agonists of nACh receptors that display limited discrimination in functional assays between receptor subtypes include http://www.guidetopharmacology.org/GRAC/LigandDisplayForward?ligandId=5460, http://www.guidetopharmacology.org/GRAC/LigandDisplayForward?ligandId=5347, http://www.guidetopharmacology.org/GRAC/LigandDisplayForward?ligandId=3967, http://www.guidetopharmacology.org/GRAC/LigandDisplayForward?ligandId=5348, http://www.guidetopharmacology.org/GRAC/LigandDisplayForward?ligandId=2585 and the natural transmitter, http://www.guidetopharmacology.org/GRAC/LigandDisplayForward?ligandId=294 (ACh). A summary of their profile across differing receptors is provided in [http://www.ncbi.nlm.nih.gov/pubmed/16876883?dopt=AbstractPlus] and quantitative data across numerous assay systems are summarized in [http://www.ncbi.nlm.nih.gov/pubmed/16033252?dopt=AbstractPlus]. Quantitative data presented in the table for commonly used antagonists and channel blockers for human receptors studied under voltage‐clamp are from [http://www.ncbi.nlm.nih.gov/pubmed/8987816?dopt=AbstractPlus, http://www.ncbi.nlm.nih.gov/pubmed/8996215?dopt=AbstractPlus, http://www.ncbi.nlm.nih.gov/pubmed/18448138?dopt=AbstractPlus, http://www.ncbi.nlm.nih.gov/pubmed/11303054?dopt=AbstractPlus, http://www.ncbi.nlm.nih.gov/pubmed/11867382?dopt=AbstractPlus, http://www.ncbi.nlm.nih.gov/pubmed/16825297?dopt=AbstractPlus]. Type I PAMs increase peak agonist‐evoked responses but have little, or no, effect on the rate of desensitization of α7 nicotinic ACh receptors whereas type II PAMs also cause a large reduction in desensitization (reviewed in [http://www.ncbi.nlm.nih.gov/pubmed/21575610?dopt=AbstractPlus]).

### Further reading on Nicotinic acetylcholine receptors

Auerbach A. (2015) Agonist activation of a nicotinic acetylcholine receptor. *Neuropharmacology* **96**: 150‐6 https://www.ncbi.nlm.nih.gov/pubmed/25446670?dopt=AbstractPlus

Bertrand D *et al.* (2015) Therapeutic Potential of α7 Nicotinic Acetylcholine Receptors. *Pharmacol. Rev.* **67**: 1025‐73 https://www.ncbi.nlm.nih.gov/pubmed/26419447?dopt=AbstractPlus

Bouzat C *et al.* (2018) Nicotinic acetylcholine receptors at the single‐channel level. *Br. J. Pharmacol.* **175**: 1789‐1804 https://www.ncbi.nlm.nih.gov/pubmed/28261794?dopt=AbstractPlus

Chatzidaki A *et al.* (2015) Allosteric modulation of nicotinic acetylcholine receptors. *Biochem. Pharmacol.* **97**: 408‐417 https://www.ncbi.nlm.nih.gov/pubmed/26231943?dopt=AbstractPlus

Corradi J *et al.* (2016) Understanding the Bases of Function and Modulation of α7 Nicotinic Receptors: Implications for Drug Discovery. *Mol. Pharmacol.* **90**: 288‐99 https://www.ncbi.nlm.nih.gov/pubmed/27190210?dopt=AbstractPlus

Crespi A *et al.* (2018) Proteins and chemical chaperones involved in neuronal nicotinic receptor expression and function: an update. *Br. J. Pharmacol.* **175**: 1869‐1879 https://www.ncbi.nlm.nih.gov/pubmed/28294298?dopt=AbstractPlus

Dineley KT *et al.* (2015) Nicotinic ACh receptors as therapeutic targets in CNS disorders. *Trends Pharmacol. Sci.* **36**: 96‐108 https://www.ncbi.nlm.nih.gov/pubmed/25639674?dopt=AbstractPlus

Lukas RJ *et al.* (1999) International Union of Pharmacology. XX. Current status of the nomenclature for nicotinic acetylcholine receptors and their subunits. *Pharmacol. Rev.* **51**: 397‐401 https://www.ncbi.nlm.nih.gov/pubmed/10353988?dopt=AbstractPlus

Stokes C *et al.* (2015) Looking below the surface of nicotinic acetylcholine receptors. *Trends Pharmacol. Sci.* **36**: 514‐23 https://www.ncbi.nlm.nih.gov/pubmed/26067101?dopt=AbstractPlus

Wang J *et al.* (2018) Orthosteric and allosteric potentiation of heteromeric neuronal nicotinic acetylcholine receptors. *Br. J. Pharmacol.* **175**: 1805‐1821 https://www.ncbi.nlm.nih.gov/pubmed/28199738?dopt=AbstractPlus

Wu J *etal*. (2016)Heteromeric α7β2 Nicotinic Acetylcholine Receptors in the Brain. *Trends Pharmacol. Sci.* **37**: 562‐574 https://www.ncbi.nlm.nih.gov/pubmed/27179601?dopt=AbstractPlus

## http://www.guidetopharmacology.org/GRAC/FamilyDisplayForward?familyId=77

### Overview

P2X receptors (**nomenclature as agreed by the NC‐IUPHAR Subcommittee on P2X Receptors** [http://www.ncbi.nlm.nih.gov/pubmed/18655795?dopt=AbstractPlus, http://www.ncbi.nlm.nih.gov/pubmed/11171941?dopt=AbstractPlus]) have a trimeric topology [http://www.ncbi.nlm.nih.gov/pubmed/14523092?dopt=AbstractPlus, http://www.ncbi.nlm.nih.gov/pubmed/19641588?dopt=AbstractPlus, http://www.ncbi.nlm.nih.gov/pubmed/9606184?dopt=AbstractPlus] with two putative TM domains, gating primarily Na^+^, K^+^ and Ca^2+^, exceptionally Cl‐. The Nomenclature Subcommittee has recommended that for P2X receptors, structural criteria should be the initial criteria for nomenclature where possible. X‐ray crystallography indicates that functional P2X receptors are trimeric and three agonist molecules are required to bind to a single receptor in order to activate it [http://www.ncbi.nlm.nih.gov/pubmed/19641589?dopt=AbstractPlus, http://www.ncbi.nlm.nih.gov/pubmed/22535247?dopt=AbstractPlus, http://www.ncbi.nlm.nih.gov/pubmed/19641588?dopt=AbstractPlus, http://www.ncbi.nlm.nih.gov/pubmed/27626375?dopt=AbstractPlus]. Native receptors may occur as either homotrimers (*e.g.* P2X1 in smooth muscle) or heterotrimers (*e.g.* P2X2:P2X3 in the nodose ganglion [http://www.ncbi.nlm.nih.gov/pubmed/9364478?dopt=AbstractPlus], P2X1:P2X5 in mouse cortical astrocytes [http://www.ncbi.nlm.nih.gov/pubmed/18495881?dopt=AbstractPlus], and P2X2:P2X5 in mouse dorsal root ganglion, spinal cord and mid pons [http://www.ncbi.nlm.nih.gov/pubmed/22442090?dopt=AbstractPlus, http://www.ncbi.nlm.nih.gov/pubmed/24391538?dopt=AbstractPlus]. P2X2, P2X4 and P2X7 receptors have been shown to form functional homopolymers which, in turn, activate pores permeable to low molecular weight solutes [http://www.ncbi.nlm.nih.gov/pubmed/18851707?dopt=AbstractPlus]. The hemi‐channel pannexin‐1 has been implicated in the pore formation induced by P2X7 [http://www.ncbi.nlm.nih.gov/pubmed/19212823?dopt=AbstractPlus], but not P2X2 [http://www.ncbi.nlm.nih.gov/pubmed/18689682?dopt=AbstractPlus], receptor activation.

**Table:** 

Nomenclature	http://www.guidetopharmacology.org/GRAC/ObjectDisplayForward?objectId=478	http://www.guidetopharmacology.org/GRAC/ObjectDisplayForward?objectId=479	http://www.guidetopharmacology.org/GRAC/ObjectDisplayForward?objectId=480	http://www.guidetopharmacology.org/GRAC/ObjectDisplayForward?objectId=481	http://www.guidetopharmacology.org/GRAC/ObjectDisplayForward?objectId=482	http://www.guidetopharmacology.org/GRAC/ObjectDisplayForward?objectId=483	http://www.guidetopharmacology.org/GRAC/ObjectDisplayForward?objectId=484
HGNC, UniProt	https://www.genenames.org/data/gene‐symbol‐report/#!/hgnc_id/HGNC:8533, http://www.uniprot.org/uniprot/P51575	https://www.genenames.org/data/gene‐symbol‐report/#!/hgnc_id/HGNC:15459, http://www.uniprot.org/uniprot/Q9UBL9	https://www.genenames.org/data/gene‐symbol‐report/#!/hgnc_id/HGNC:8534, http://www.uniprot.org/uniprot/P56373	https://www.genenames.org/data/gene‐symbol‐report/#!/hgnc_id/HGNC:8535, http://www.uniprot.org/uniprot/Q99571	https://www.genenames.org/data/gene‐symbol‐report/#!/hgnc_id/HGNC:8536, http://www.uniprot.org/uniprot/Q93086	https://www.genenames.org/data/gene‐symbol‐report/#!/hgnc_id/HGNC:8538, http://www.uniprot.org/uniprot/O15547	https://www.genenames.org/data/gene‐symbol‐report/#!/hgnc_id/HGNC:8537, http://www.uniprot.org/uniprot/Q99572
Endogenous agonists	–	http://www.guidetopharmacology.org/GRAC/LigandDisplayForward?ligandId=1713 [http://www.ncbi.nlm.nih.gov/pubmed/18600475?dopt=AbstractPlus] – Rat	http://www.guidetopharmacology.org/GRAC/LigandDisplayForward?ligandId=1713 [http://www.ncbi.nlm.nih.gov/pubmed/12213051?dopt=AbstractPlus]	http://www.guidetopharmacology.org/GRAC/LigandDisplayForward?ligandId=1713 [http://www.ncbi.nlm.nih.gov/pubmed/12213051?dopt=AbstractPlus]	http://www.guidetopharmacology.org/GRAC/LigandDisplayForward?ligandId=1713 [http://www.ncbi.nlm.nih.gov/pubmed/12213051?dopt=AbstractPlus] – Rat	http://www.guidetopharmacology.org/GRAC/LigandDisplayForward?ligandId=1713 [http://www.ncbi.nlm.nih.gov/pubmed/12213051?dopt=AbstractPlus] – Rat	http://www.guidetopharmacology.org/GRAC/LigandDisplayForward?ligandId=1713 [http://www.ncbi.nlm.nih.gov/pubmed/12213051?dopt=AbstractPlus]
Agonists	http://www.guidetopharmacology.org/GRAC/LigandDisplayForward?ligandId=4093‐http://www.guidetopharmacology.org/GRAC/LigandDisplayForward?ligandId=4093, http://www.guidetopharmacology.org/GRAC/LigandDisplayForward?ligandId=1757, http://www.guidetopharmacology.org/GRAC/LigandDisplayForward?ligandId=4236‐http://www.guidetopharmacology.org/GRAC/LigandDisplayForward?ligandId=4236	–	http://www.guidetopharmacology.org/GRAC/LigandDisplayForward?ligandId=4093‐http://www.guidetopharmacology.org/GRAC/LigandDisplayForward?ligandId=4093, http://www.guidetopharmacology.org/GRAC/LigandDisplayForward?ligandId=1757	–	–	–	–
Antagonists	http://www.guidetopharmacology.org/GRAC/LigandDisplayForward?ligandId=4324 (pIC_50_ ∼8.9) [http://www.ncbi.nlm.nih.gov/pubmed/9614197?dopt=AbstractPlus], http://www.guidetopharmacology.org/GRAC/LigandDisplayForward?ligandId=4225 (pIC_50_ ∼8.5), http://www.guidetopharmacology.org/GRAC/LigandDisplayForward?ligandId=4266 (pIC_50_ ∼6.7), http://www.guidetopharmacology.org/GRAC/LigandDisplayForward?ligandId=4267 (pIC_50_ ∼6.3) [http://www.ncbi.nlm.nih.gov/pubmed/15072843?dopt=AbstractPlus]	http://www.guidetopharmacology.org/GRAC/LigandDisplayForward?ligandId=9545 (pIC_50_ 7–8) [http://www.ncbi.nlm.nih.gov/pubmed/23253448?dopt=AbstractPlus], http://www.guidetopharmacology.org/GRAC/LigandDisplayForward?ligandId=9546 (pIC_50_ 7–8) [http://www.ncbi.nlm.nih.gov/pubmed/23253448?dopt=AbstractPlus], http://www.guidetopharmacology.org/GRAC/LigandDisplayForward?ligandId=9539 (pIC_50_ ∼7) [http://www.ncbi.nlm.nih.gov/pubmed/23253448?dopt=AbstractPlus]	http://www.guidetopharmacology.org/GRAC/LigandDisplayForward?ligandId=4324 (pIC_50_ ∼8.9) [http://www.ncbi.nlm.nih.gov/pubmed/9614197?dopt=AbstractPlus], http://www.guidetopharmacology.org/GRAC/LigandDisplayForward?ligandId=9538 (pIC_50_ 8.9) [http://www.ncbi.nlm.nih.gov/pubmed/26686393?dopt=AbstractPlus], http://www.guidetopharmacology.org/GRAC/LigandDisplayForward?ligandId=9540 (pIC_50_ 8.5) [http://www.ncbi.nlm.nih.gov/pubmed/26686393?dopt=AbstractPlus], http://www.guidetopharmacology.org/GRAC/LigandDisplayForward?ligandId=4115 (pIC_50_ ∼7.5) [http://www.ncbi.nlm.nih.gov/pubmed/12482951?dopt=AbstractPlus]	http://www.guidetopharmacology.org/GRAC/LigandDisplayForward?ligandId=9541 (pIC_50_ 5–6) [http://www.ncbi.nlm.nih.gov/pubmed/26686393?dopt=AbstractPlus, http://www.ncbi.nlm.nih.gov/pubmed/23253448?dopt=AbstractPlus], http://www.guidetopharmacology.org/GRAC/LigandDisplayForward?ligandId=9543 (pIC_50_ 5–6) [http://www.ncbi.nlm.nih.gov/pubmed/26686393?dopt=AbstractPlus, http://www.ncbi.nlm.nih.gov/pubmed/23253448?dopt=AbstractPlus], http://www.guidetopharmacology.org/GRAC/LigandDisplayForward?ligandId=9542 (pIC_50_ 5–6) [http://www.ncbi.nlm.nih.gov/pubmed/26686393?dopt=AbstractPlus, http://www.ncbi.nlm.nih.gov/pubmed/23253448?dopt=AbstractPlus], http://www.guidetopharmacology.org/GRAC/LigandDisplayForward?ligandId=4790 (pIC_50_ 5–6) [http://www.ncbi.nlm.nih.gov/pubmed/26686393?dopt=AbstractPlus, http://www.ncbi.nlm.nih.gov/pubmed/23253448?dopt=AbstractPlus]	–	–	http://www.guidetopharmacology.org/GRAC/LigandDisplayForward?ligandId=9021 (p*K*_d_ 8.9) [http://www.ncbi.nlm.nih.gov/pubmed/18071294?dopt=AbstractPlus], http://www.guidetopharmacology.org/GRAC/LigandDisplayForward?ligandId=4121 (pIC_50_ ∼8), http://www.guidetopharmacology.org/GRAC/LigandDisplayForward?ligandId=4147 (pIC_50_ ∼8) [http://www.ncbi.nlm.nih.gov/pubmed/10860929?dopt=AbstractPlus], http://www.guidetopharmacology.org/GRAC/LigandDisplayForward?ligandId=4122 (pIC_50_ ∼7.7) [http://www.ncbi.nlm.nih.gov/pubmed/17471177?dopt=AbstractPlus, http://www.ncbi.nlm.nih.gov/pubmed/19558545?dopt=AbstractPlus, http://www.ncbi.nlm.nih.gov/pubmed/19464323?dopt=AbstractPlus], http://www.guidetopharmacology.org/GRAC/LigandDisplayForward?ligandId=4119 (pIC_50_ 7.4) [http://www.ncbi.nlm.nih.gov/pubmed/16982702?dopt=AbstractPlus], http://www.guidetopharmacology.org/GRAC/LigandDisplayForward?ligandId=2459 (pA2 = 7.4) (p*A*_2_ 7.4) [http://www.ncbi.nlm.nih.gov/pubmed/16487507?dopt=AbstractPlus], http://www.guidetopharmacology.org/GRAC/LigandDisplayForward?ligandId=4118 (pIC_50_ ∼6.9) [http://www.ncbi.nlm.nih.gov/pubmed/17471177?dopt=AbstractPlus], http://www.guidetopharmacology.org/GRAC/LigandDisplayForward?ligandId=7722 (p*A*_2_ 6.1) [36]
Selective antagonists	–	–	–	–	–	–	http://www.guidetopharmacology.org/GRAC/LigandDisplayForward?ligandId=7538 (p*K*_i_ 7.9) [http://www.ncbi.nlm.nih.gov/pubmed/23889535?dopt=AbstractPlus]
Allosteric modulators	–	–	–	–	–	–	http://www.guidetopharmacology.org/GRAC/LigandDisplayForward?ligandId=9021 (Negative) [http://www.ncbi.nlm.nih.gov/pubmed/18071294?dopt=AbstractPlus], http://www.guidetopharmacology.org/GRAC/LigandDisplayForward?ligandId=9022 (Negative) [http://www.ncbi.nlm.nih.gov/pubmed/18071294?dopt=AbstractPlus, http://www.ncbi.nlm.nih.gov/pubmed/18660826?dopt=AbstractPlus], http://www.guidetopharmacology.org/GRAC/LigandDisplayForward?ligandId=9022 (Positive) [http://www.ncbi.nlm.nih.gov/pubmed/18071294?dopt=AbstractPlus, http://www.ncbi.nlm.nih.gov/pubmed/18660826?dopt=AbstractPlus] – Rat, http://www.guidetopharmacology.org/GRAC/LigandDisplayForward?ligandId=5527 (https://www.genenames.org/data/gene‐symbol‐report/#!/hgnc_id/HGNC:1472, http://www.uniprot.org/uniprot/P49913) (Positive) [http://www.ncbi.nlm.nih.gov/pubmed/18765670?dopt=AbstractPlus], http://www.guidetopharmacology.org/GRAC/LigandDisplayForward?ligandId=6063 (Positive) [http://www.ncbi.nlm.nih.gov/pubmed/21262970?dopt=AbstractPlus], http://www.guidetopharmacology.org/GRAC/LigandDisplayForward?ligandId=10338 (Positive) [http://www.ncbi.nlm.nih.gov/pubmed/15383600?dopt=AbstractPlus]
Selective allosteric modulators	http://www.guidetopharmacology.org/GRAC/LigandDisplayForward?ligandId=6496 (Positive) [http://www.ncbi.nlm.nih.gov/pubmed/9632352?dopt=AbstractPlus]	–	–	http://www.guidetopharmacology.org/GRAC/LigandDisplayForward?ligandId=2373 (Positive) (pEC_50_ ∼6.6) [http://www.ncbi.nlm.nih.gov/pubmed/10460235?dopt=AbstractPlus] – Rat	–	–	http://www.guidetopharmacology.org/GRAC/LigandDisplayForward?ligandId=5953 (Negative) (pIC_50_ 5.2) [http://www.ncbi.nlm.nih.gov/pubmed/15210579?dopt=AbstractPlus], http://www.guidetopharmacology.org/GRAC/LigandDisplayForward?ligandId=4142 (Negative) [http://www.ncbi.nlm.nih.gov/pubmed/19309360?dopt=AbstractPlus, http://www.ncbi.nlm.nih.gov/pubmed/17031385?dopt=AbstractPlus], http://www.guidetopharmacology.org/GRAC/LigandDisplayForward?ligandId=4235 (Negative) [http://www.ncbi.nlm.nih.gov/pubmed/9113369?dopt=AbstractPlus, http://www.ncbi.nlm.nih.gov/pubmed/15210579?dopt=AbstractPlus], http://www.guidetopharmacology.org/GRAC/LigandDisplayForward?ligandId=2373 (Positive) [http://www.ncbi.nlm.nih.gov/pubmed/22506590?dopt=AbstractPlus]
Comments	–	–	–	–	–	–	Effects of the allosteric regulators at P2X7 receptors are species‐dependent.

### Comments

http://www.guidetopharmacology.org/GRAC/LigandDisplayForward?ligandId=4115 and http://www.guidetopharmacology.org/GRAC/LigandDisplayForward?ligandId=4298 also block the P2X2:P2X3 heteromultimer [http://www.ncbi.nlm.nih.gov/pubmed/16465177?dopt=AbstractPlus, http://www.ncbi.nlm.nih.gov/pubmed/12482951?dopt=AbstractPlus]. http://www.guidetopharmacology.org/GRAC/LigandDisplayForward?ligandId=4267, http://www.guidetopharmacology.org/GRAC/LigandDisplayForward?ligandId=4115 and http://www.guidetopharmacology.org/GRAC/LigandDisplayForward?ligandId=4298 are more than 10‐fold selective for P2X1 and P2X3 receptors, respectively.

Agonists listed show selectivity within recombinant P2X receptors of *ca*. one order of magnitude. http://www.guidetopharmacology.org/GRAC/LigandDisplayForward?ligandId=4121, http://www.guidetopharmacology.org/GRAC/LigandDisplayForward?ligandId=4122, http://www.guidetopharmacology.org/GRAC/LigandDisplayForward?ligandId=4119 and http://www.guidetopharmacology.org/GRAC/LigandDisplayForward?ligandId=4118 are at least 10‐fold selective for P2X7 receptors and show similar affinity across human and rodent receptors [http://www.ncbi.nlm.nih.gov/pubmed/17471177?dopt=AbstractPlus, http://www.ncbi.nlm.nih.gov/pubmed/19558545?dopt=AbstractPlus, http://www.ncbi.nlm.nih.gov/pubmed/19464323?dopt=AbstractPlus]. Several P2X receptors (particularly P2X1 and P2X3) may be inhibited by desensitisation using stable agonists (*e.g*. http://www.guidetopharmacology.org/GRAC/LigandDisplayForward?ligandId=4093‐http://www.guidetopharmacology.org/GRAC/LigandDisplayForward?ligandId=4093); http://www.guidetopharmacology.org/GRAC/LigandDisplayForward?ligandId=1728 and http://www.guidetopharmacology.org/GRAC/LigandDisplayForward?ligandId=1725 are non‐selective antagonists at rat and human P2X1‐3,5 and hP2X4, but not rP2X4,6,7 [http://www.ncbi.nlm.nih.gov/pubmed/8598206?dopt=AbstractPlus], and can also inhibit ATPase activity [http://www.ncbi.nlm.nih.gov/pubmed/7889301?dopt=AbstractPlus]. http://www.guidetopharmacology.org/GRAC/LigandDisplayForward?ligandId=4225 is inactive at rP2X2, an antagonist at rP2X3 (pIC_50_ 5.6) and enhances agonist responses at rP2X4 [http://www.ncbi.nlm.nih.gov/pubmed/10556935?dopt=AbstractPlus]. Antagonist potency of http://www.guidetopharmacology.org/GRAC/LigandDisplayForward?ligandId=4266 at recombinant P2X2, P2X3 and P2X5 is two orders of magnitude lower than that at P2X1 receptors [http://www.ncbi.nlm.nih.gov/pubmed/10193905?dopt=AbstractPlus]. The P2X7 receptor may be inhibited in a non‐competitive manner by the protein kinase inhibitors http://www.guidetopharmacology.org/GRAC/LigandDisplayForward?ligandId=4235 and http://www.guidetopharmacology.org/GRAC/LigandDisplayForward?ligandId=5953 [http://www.ncbi.nlm.nih.gov/pubmed/15210579?dopt=AbstractPlus], while the p38 MAP kinase inhibitor http://www.guidetopharmacology.org/GRAC/LigandDisplayForward?ligandId=4207 and the cyclic imide http://www.guidetopharmacology.org/GRAC/LigandDisplayForward?ligandId=4142 show a species‐dependent non‐competitive action [http://www.ncbi.nlm.nih.gov/pubmed/14634045?dopt=AbstractPlus, http://www.ncbi.nlm.nih.gov/pubmed/19309360?dopt=AbstractPlus, http://www.ncbi.nlm.nih.gov/pubmed/17031382?dopt=AbstractPlus, http://www.ncbi.nlm.nih.gov/pubmed/17031385?dopt=AbstractPlus].

The pH‐sensitive dye used in culture media, phenol red, is also reported to inhibit P2X1 and P2X3 containing channels [http://www.ncbi.nlm.nih.gov/pubmed/15778739?dopt=AbstractPlus]. Some recombinant P2X receptors expressed to high density bind http://www.guidetopharmacology.org/GRAC/LigandDisplayForward?ligandId=5406 and http://www.guidetopharmacology.org/GRAC/LigandDisplayForward?ligandId=5405‐http://www.guidetopharmacology.org/GRAC/LigandDisplayForward?ligandId=5405, although the latter can also bind to 5‐nucleotidase [http://www.ncbi.nlm.nih.gov/pubmed/8548175?dopt=AbstractPlus]. http://www.guidetopharmacology.org/GRAC/LigandDisplayForward?ligandId=5407 and http://www.guidetopharmacology.org/GRAC/LigandDisplayForward?ligandId=5408 have been used as high affinity antagonist radioligands for P2X3 (and P2X2/3) and P2X7 receptors, respectively [http://www.ncbi.nlm.nih.gov/pubmed/19558545?dopt=AbstractPlus]. Several high affinity radioligands for the P2X7 receptor have been recently synthesized [http://www.ncbi.nlm.nih.gov/pubmed/28374288?dopt=AbstractPlus, http://www.ncbi.nlm.nih.gov/pubmed/26386289?dopt=AbstractPlus, http://www.ncbi.nlm.nih.gov/pubmed/27765863?dopt=AbstractPlus]. http://www.guidetopharmacology.org/GRAC/LigandDisplayForward?ligandId=9540 has shown clinical efficacy in reducing refractory chronic cough [http://www.ncbi.nlm.nih.gov/pubmed/25467586?dopt=AbstractPlus].

### Further reading on P2X receptors

Di Virgilio F *et al.* (2017) The P2X7 Receptor in Infection and Inflammation. *Immunity* **47**: 15‐31 https://www.ncbi.nlm.nih.gov/pubmed/28723547?dopt=AbstractPlus

DiVirgilio F *etal*. (2018)TheElusive P2X7Macropore. *TrendsCellBiol.* **28**: 392‐404https://www.ncbi.nlm.nih.gov/pubmed/29439897?dopt=AbstractPlus

Habermacher C *et al.* (2016) Molecular structure and function of P2X receptors. *Neuropharmacology* **104**: 18‐30 https://www.ncbi.nlm.nih.gov/pubmed/26231831?dopt=AbstractPlus

Jacobson KA *et al.* (2016) Medicinal chemistry of adenosine, P2Y and P2X receptors. *Neuropharmacology* **104**: 31‐49 https://www.ncbi.nlm.nih.gov/pubmed/26686393?dopt=AbstractPlus

Khakh BS *et al.* (2001) International union of pharmacology. XXIV. Current status of the nomenclature and properties of P2X receptors and their subunits. *Pharmacol. Rev.* **53**: 107‐18 https://www.ncbi.nlm.nih.gov/pubmed/11171941?dopt=AbstractPlus

North RA. (2016) P2X receptors. *Philos. Trans. R. Soc. Lond., B, Biol. Sci.* **371**: https://www.ncbi.nlm.nih.gov/pubmed/27377721?dopt=AbstractPlus

Stokes L *et al.* (2017) P2X4 Receptor Function in the Nervous System and Current Breakthroughs in Pharmacology. *Front Pharmacol* **8**: 291 https://www.ncbi.nlm.nih.gov/pubmed/28588493?dopt=AbstractPlus

## http://www.guidetopharmacology.org/GRAC/FamilyDisplayForward?familyId=83

### Overview

The zinc‐activated channel (ZAC, **nomenclature as agreed by the NC‐IUPHAR Subcommittee for the Zinc Activated Channel**) is a member of the Cys‐loop family that includes the nicotinic ACh, 5‐HT_3_, GABA_A_ and strychnine‐sensitive glycine receptors [http://www.ncbi.nlm.nih.gov/pubmed/12381728?dopt=AbstractPlus, http://www.ncbi.nlm.nih.gov/pubmed/16083862?dopt=AbstractPlus, http://www.ncbi.nlm.nih.gov/pubmed/26872532?dopt=AbstractPlus]. The channel is likely to exist as a homopentamer of 4TM subunits that form an intrinsic cation selective channel equipermeable to Na^+^, K^+^ and Cs^+^, but impermeable to Ca^2+^ and Mg^2+^ [http://www.ncbi.nlm.nih.gov/pubmed/26872532?dopt=AbstractPlus]. ZAC displays constitutive activity that can be blocked by http://www.guidetopharmacology.org/GRAC/LigandDisplayForward?ligandId=2294 and high concentrations of Ca^2+^ [http://www.ncbi.nlm.nih.gov/pubmed/26872532?dopt=AbstractPlus]. Although denoted ZAC, the channel is more potently activated by protons and copper, with greater and lesser efficacy than zinc, respectively [http://www.ncbi.nlm.nih.gov/pubmed/26872532?dopt=AbstractPlus]. ZAC is present in the human, chimpanzee, dog, cow and opossum genomes, but is functionally absent from mouse, or rat, genomes [http://www.ncbi.nlm.nih.gov/pubmed/12381728?dopt=AbstractPlus, http://www.ncbi.nlm.nih.gov/pubmed/16083862?dopt=AbstractPlus].

**Table:** 

Nomenclature	http://www.guidetopharmacology.org/GRAC/ObjectDisplayForward?objectId=587
HGNC, UniProt	https://www.genenames.org/data/gene‐symbol‐report/#!/hgnc_id/HGNC:29504, http://www.uniprot.org/uniprot/Q401N2
Endogenous agonists	http://www.guidetopharmacology.org/GRAC/LigandDisplayForward?ligandId=2346 ^+^ [http://www.ncbi.nlm.nih.gov/pubmed/26872532?dopt=AbstractPlus], http://www.guidetopharmacology.org/GRAC/LigandDisplayForward?ligandId=4164 [http://www.ncbi.nlm.nih.gov/pubmed/26872532?dopt=AbstractPlus], http://www.guidetopharmacology.org/GRAC/LigandDisplayForward?ligandId=566 [http://www.ncbi.nlm.nih.gov/pubmed/12381728?dopt=AbstractPlus, http://www.ncbi.nlm.nih.gov/pubmed/26872532?dopt=AbstractPlus]
Antagonists	http://www.guidetopharmacology.org/GRAC/LigandDisplayForward?ligandId=2294 (pIC_50_ 5.2) [http://www.ncbi.nlm.nih.gov/pubmed/12381728?dopt=AbstractPlus], http://www.guidetopharmacology.org/GRAC/LigandDisplayForward?ligandId=707 (pIC_50_ 2) [http://www.ncbi.nlm.nih.gov/pubmed/26872532?dopt=AbstractPlus]
Functional Characteristics	Outwardly rectifying current (both constitutive and evoked by Zn^2+^)

### Comments

The ZAC subunit does not appear to exist in the mouse or rat genomes [http://www.ncbi.nlm.nih.gov/pubmed/12381728?dopt=AbstractPlus]. Although tabulated as an antagonist, it is possible that http://www.guidetopharmacology.org/GRAC/LigandDisplayForward?ligandId=2294 acts as a channel blocker. Antagonism by Ca^2+^ is voltage‐independent. ZAC is not activated (at 1 mM) by transition metals including Fe^2+^, Co^2+^, Ni^2+^, Cd^2+^, or Al^3+^ [http://www.ncbi.nlm.nih.gov/pubmed/26872532?dopt=AbstractPlus]. The concentration response relationship to Cu^2+^ is biphasic, with concentrations exceeding 30 *μ*M being associated with reduced activation [http://www.ncbi.nlm.nih.gov/pubmed/26872532?dopt=AbstractPlus].

### Further reading on ZAC

Collingridge GL *et al.* (2009) A nomenclature for ligand‐gated ion channels. *Neuropharmacology* **56**: 2‐5 https://www.ncbi.nlm.nih.gov/pubmed/18655795?dopt=AbstractPlus

Peralta FA *et al.* (2016) Zinc as Allosteric Ion Channel Modulator: Ionotropic Receptors as Metalloproteins. *Int J Mol Sci* **17**: https://www.ncbi.nlm.nih.gov/pubmed/27384555?dopt=AbstractPlus

Trattnig SM *et al.* (2016) Copper and protons directly activate the zinc‐activated channel. *Biochem. Pharmacol.* **103**: 109‐17 https://www.ncbi.nlm.nih.gov/pubmed/26872532?dopt=AbstractPlus

## http://www.guidetopharmacology.org/GRAC/FamilyDisplayForward?familyId=696

### Overview

The voltage‐gated ion channels and their structural relatives comprise a superfamily encoded by at least 143 genes in the human genome and are therefore one of the largest superfamilies of signal transduction proteins, following the G proteincoupled receptors and the protein kinases in number [http://www.ncbi.nlm.nih.gov/pubmed/16036917?dopt=AbstractPlus]. In addition to their prominence in signal transduction, these ion channels are also among the most common drug targets. As for other large protein superfamilies, understanding the molecular relationships among family members, developing a unified, rational nomenclature for the ion channel families and subfamilies, and assigning physiological functions and pharmacological significance to each family member has been an important challenge.

### Further reading on Voltage‐gated ion channels

Catterall WA *et al.* (2005) Introduction to the IUPHAR Compendium of Voltage‐Gated Ion Channels 2005 *Pharmacological Reviews* **57**: 385

## http://www.guidetopharmacology.org/GRAC/FamilyDisplayForward?familyId=70

### Overview

CatSper channels (CatSper1‐4, **nomenclature as agreed by NC‐IUPHAR** [http://www.ncbi.nlm.nih.gov/pubmed/16382101?dopt=AbstractPlus]) are putative 6TM, voltage‐gated, alkalinization‐activated calcium permeant channels that are presumed to assemble as a tetramer of α‐like subunits and mediate the current I_CatSper_ [http://www.ncbi.nlm.nih.gov/pubmed/16467839?dopt=AbstractPlus]. In mammals, CatSper subunits are structurally most closely related to individual domains of voltageactivated calcium channels (Cav) [http://www.ncbi.nlm.nih.gov/pubmed/11595941?dopt=AbstractPlus]. CatSper1 [http://www.ncbi.nlm.nih.gov/pubmed/11595941?dopt=AbstractPlus], CatSper2 [http://www.ncbi.nlm.nih.gov/pubmed/11675491?dopt=AbstractPlus] and CatSpers 3 and 4 [http://www.ncbi.nlm.nih.gov/pubmed/16107607?dopt=AbstractPlus, http://www.ncbi.nlm.nih.gov/pubmed/12932298?dopt=AbstractPlus, http://www.ncbi.nlm.nih.gov/pubmed/17227845?dopt=AbstractPlus], in common with a putative 2TM auxiliary CatSperβ protein [http://www.ncbi.nlm.nih.gov/pubmed/17478420?dopt=AbstractPlus] and two putative 1TM associated CatSperγ and CatSper*δ* proteins [http://www.ncbi.nlm.nih.gov/pubmed/21224844?dopt=AbstractPlus, http://www.ncbi.nlm.nih.gov/pubmed/19516020?dopt=AbstractPlus], are restricted to the testis and localised to the principle piece of sperm tail. The novel cross‐species CatSper channel inhibitor, http://www.guidetopharmacology.org/GRAC/LigandDisplayForward?ligandId=10266, has been proposed as a useful tool to aid characterisation of native CatSper channels [http://www.ncbi.nlm.nih.gov/pubmed/29723408?dopt=AbstractPlus].

Two‐pore channels (TPCs) are structurally related to CatSpers, CaVs and NaVs. TPCs have a 2x6TM structure with twice the number of TMs of CatSpers and half that of CaVs. There are three animal TPCs (TPC1‐TPC3). Humans have TPC1 and TPC2, but not TPC3. TPC1 and TPC2 are localized in endosomes and lysosomes [http://www.ncbi.nlm.nih.gov/pubmed/19387438?dopt=AbstractPlus]. TPC3 is also found on the plasma membrane and forms a voltage‐activated, non‐inactivating Na^+^ channel [http://www.ncbi.nlm.nih.gov/pubmed/25256615?dopt=AbstractPlus]. All the three TPCs are Na^+^‐selective under whole‐cell or whole‐organelle patch clamp recording [http://www.ncbi.nlm.nih.gov/pubmed/24776928?dopt=AbstractPlus, http://www.ncbi.nlm.nih.gov/pubmed/23394946?dopt=AbstractPlus, http://www.ncbi.nlm.nih.gov/pubmed/19211808?dopt=AbstractPlus]. The channels may also conduct Ca^2+^ [http://www.ncbi.nlm.nih.gov/pubmed/24277557?dopt=AbstractPlus].

**Table:** 

Nomenclature	http://www.guidetopharmacology.org/GRAC/ObjectDisplayForward?objectId=388	http://www.guidetopharmacology.org/GRAC/ObjectDisplayForward?objectId=389	http://www.guidetopharmacology.org/GRAC/ObjectDisplayForward?objectId=390	http://www.guidetopharmacology.org/GRAC/ObjectDisplayForward?objectId=391
HGNC, UniProt	https://www.genenames.org/data/gene‐symbol‐report/#!/hgnc_id/HGNC:17116, http://www.uniprot.org/uniprot/Q8NEC5	https://www.genenames.org/data/gene‐symbol‐report/#!/hgnc_id/HGNC:18810, http://www.uniprot.org/uniprot/Q96P56	https://www.genenames.org/data/gene‐symbol‐report/#!/hgnc_id/HGNC:20819, http://www.uniprot.org/uniprot/Q86XQ3	https://www.genenames.org/data/gene‐symbol‐report/#!/hgnc_id/HGNC:23220, http://www.uniprot.org/uniprot/Q7RTX7
Activators	CatSper1 is constitutively active, weakly facilitated by membrane depolarisation, strongly augmented by intracellular alkalinisation. In human, but not mouse, progesterone (EC50 8 nM) also potentiates the CatSper current (I_CatSper_). [http://www.ncbi.nlm.nih.gov/pubmed/21412339?dopt=AbstractPlus, http://www.ncbi.nlm.nih.gov/pubmed/21412338?dopt=AbstractPlus]	–	–	–
Channel blockers	http://www.guidetopharmacology.org/GRAC/LigandDisplayForward?ligandId=2432 (Inhibition) (pIC_50_ 5) [http://www.ncbi.nlm.nih.gov/pubmed/16467839?dopt=AbstractPlus] – Mouse, http://www.guidetopharmacology.org/GRAC/LigandDisplayForward?ligandId=4212 (pIC_50_ 4.7) [http://www.ncbi.nlm.nih.gov/pubmed/19718436?dopt=AbstractPlus], http://www.guidetopharmacology.org/GRAC/LigandDisplayForward?ligandId=2440 (Inhibition) (pIC_50_ 3.7) [http://www.ncbi.nlm.nih.gov/pubmed/16467839?dopt=AbstractPlus] – Mouse, http://www.guidetopharmacology.org/GRAC/LigandDisplayForward?ligandId=2476 (Inhibition) (pIC_50_ 3.5) [http://www.ncbi.nlm.nih.gov/pubmed/16467839?dopt=AbstractPlus] – Mouse	–	–	–
Selective channel blockers	http://www.guidetopharmacology.org/GRAC/LigandDisplayForward?ligandId=4269 (Inhibition) (pIC_50_ 5.7) [−80mV – 80mV] [http://www.ncbi.nlm.nih.gov/pubmed/21412339?dopt=AbstractPlus, http://www.ncbi.nlm.nih.gov/pubmed/21412338?dopt=AbstractPlus], http://www.guidetopharmacology.org/GRAC/LigandDisplayForward?ligandId=2522 (Inhibition) (pIC_50_ 4.4–4.5) [http://www.ncbi.nlm.nih.gov/pubmed/21412338?dopt=AbstractPlus]	–	–	–
Functional Characteristics	Calcium selective ion channel (Ba^2+^>Ca^2+^Mg^2+^Na^+^); quasilinear monovalent cation current in the absence of extracellular divalent cations; alkalinization shifts the voltage‐dependence of activation towards negative potentials [V @ pH 6.0 = +87 mV (mouse); V @ pH 7.5 = +11mV (mouse) or pH 7.4 = +85 mV (human)]; required for I_CatSper_ and male fertility (mouse and human)	Required for I_CatSper_ and male fertility (mouse and human)	Required for I_CatSper_ and male fertility (mouse)	Required for I_CatSper_ and male fertility (mouse)

**Table:** 

Nomenclature	http://www.guidetopharmacology.org/GRAC/ObjectDisplayForward?objectId=392	http://www.guidetopharmacology.org/GRAC/ObjectDisplayForward?objectId=393
HGNC, UniProt	https://www.genenames.org/data/gene‐symbol‐report/#!/hgnc_id/HGNC:18182, http://www.uniprot.org/uniprot/Q9ULQ1	https://www.genenames.org/data/gene‐symbol‐report/#!/hgnc_id/HGNC:20820, http://www.uniprot.org/uniprot/Q8NHX9
Activators	http://www.guidetopharmacology.org/GRAC/LigandDisplayForward?ligandId=2794 (pEC_50_ 6.5) [http://www.ncbi.nlm.nih.gov/pubmed/24776928?dopt=AbstractPlus]	http://www.guidetopharmacology.org/GRAC/LigandDisplayForward?ligandId=2794 (pEC_50_ 6.4) [http://www.ncbi.nlm.nih.gov/pubmed/23063126?dopt=AbstractPlus]
Channel blockers	http://www.guidetopharmacology.org/GRAC/LigandDisplayForward?ligandId=2406 (Inhibition) (pIC_50_ 4.6) [http://www.ncbi.nlm.nih.gov/pubmed/24776928?dopt=AbstractPlus], http://www.guidetopharmacology.org/GRAC/LigandDisplayForward?ligandId=2440 (Inhibition) (pIC_50_ 3.7) [http://www.ncbi.nlm.nih.gov/pubmed/24776928?dopt=AbstractPlus]	http://www.guidetopharmacology.org/GRAC/LigandDisplayForward?ligandId=2406 (Inhibition) (pIC_50_ 5) [http://www.ncbi.nlm.nih.gov/pubmed/23063126?dopt=AbstractPlus]
Functional Characteristics	Organelle voltage‐gated Na^+^‐selective channel (Na^+^K^+^Ca^2+^); Required for the generation of action potential‐like long depolarization in lysosomes. Voltage‐dependence of activation is sensitive to luminal pH (determined from lysosomal recordings). ^*ψ*^_1_/2 @ pH4.6 = +91 mV; ^*ψ*^_1_/2 @ pH6.5 = +2.6 mV. Maximum activity requires PI(3,5)P2 and reduced [ATP], or depletion of extracellular amino acids.	Organelle voltage‐independent Na^+^‐selective channel (Na^+^K^+^Ca^2+^). Sensitive to the levels of PI(3,5)P2. Activated by decreases in [ATP] or depletion of extracellular amino acids

### Comments

CatSper channel subunits expressed singly, or in combination, fail to functionally express in heterologous expression systems [http://www.ncbi.nlm.nih.gov/pubmed/11675491?dopt=AbstractPlus, http://www.ncbi.nlm.nih.gov/pubmed/11595941?dopt=AbstractPlus]. The properties of CatSper1 tabulated above are derived from whole cell voltage‐clamp recordings comparing currents endogenous to spermatozoa isolated from the *corpus epididymis* of wild‐type and*Catsper1^(‐/‐)^* mice [http://www.ncbi.nlm.nih.gov/pubmed/16467839?dopt=AbstractPlus] and also mature human sperm [http://www.ncbi.nlm.nih.gov/pubmed/21412339?dopt=AbstractPlus, http://www.ncbi.nlm.nih.gov/pubmed/21412338?dopt=AbstractPlus]. I_CatSper_ is also undetectable in the spermatozoa of *Catsper2^(−/−)^*, *Catsper3^(−/−)^*, *Catsper4^(−/−),^* or CatSper*δ* ^(−/−)^ mice, and CatSper 1 associates with CatSper 2, 3, 4, β, γ, and *δ* [http://www.ncbi.nlm.nih.gov/pubmed/21224844?dopt=AbstractPlus, http://www.ncbi.nlm.nih.gov/pubmed/17478420?dopt=AbstractPlus, http://www.ncbi.nlm.nih.gov/pubmed/17227845?dopt=AbstractPlus]. Moreover, targeted disruption of *Catsper1, 2, 3, 4*, or *δ* genes results in an identical phenotype in which spermatozoa fail to exhibit the hyperactive movement (whip‐like flagellar beats) necessary for penetration of the egg *cumulus* and *zona pellucida* and subsequent fertilization. Such disruptions are associated with a deficit in alkalinization and depolarization‐evoked Ca^2+^ entry into spermatozoa [http://www.ncbi.nlm.nih.gov/pubmed/16036917?dopt=AbstractPlus,http://www.ncbi.nlm.nih.gov/pubmed/16036917?dopt=AbstractPlus, http://www.ncbi.nlm.nih.gov/pubmed/17227845?dopt=AbstractPlus]. Thus, it is likely that the CatSper pore is formed by a heterotetramer of CatSpers1‐4 [http://www.ncbi.nlm.nih.gov/pubmed/17227845?dopt=AbstractPlus] in association with the auxiliary subunits (β, γ, *δ*) that are also essential for function [http://www.ncbi.nlm.nih.gov/pubmed/21224844?dopt=AbstractPlus]. CatSper channels are required for the increase in intracellular Ca^2+^ concentration in sperm evoked by egg *zona pellucida* glycoproteins [http://www.ncbi.nlm.nih.gov/pubmed/19211808?dopt=AbstractPlus]. Mouse and human sperm swim against the fluid flow and Ca^2+^ signaling through CatSper is required for the rheotaxis [http://www.ncbi.nlm.nih.gov/pubmed/23453951?dopt=AbstractPlus]. *In vivo*, CatSper1‐null spermatozoa cannot ascend the female reproductive tracts efficiently [http://www.ncbi.nlm.nih.gov/pubmed/24813608?dopt=AbstractPlus, http://www.ncbi.nlm.nih.gov/pubmed/19210926?dopt=AbstractPlus]. It has been shown that CatSper channels form four linear Ca^2+^ signaling domains along the flagella, which orchestrate capacitation‐associated tyrosine phosphorylation [http://www.ncbi.nlm.nih.gov/pubmed/24813608?dopt=AbstractPlus].The driving force for Ca^2+^ entry is principally determined by a mildly outwardly rectifying K^+^ channel (KSper) that, like CatSpers, is activated by intracellular alkalinization [http://www.ncbi.nlm.nih.gov/pubmed/17460039?dopt=AbstractPlus]. Mouse KSper is encoded by *mSlo3*, a protein detected only in testis [http://www.ncbi.nlm.nih.gov/pubmed/19338774?dopt=AbstractPlus, http://www.ncbi.nlm.nih.gov/pubmed/17460039?dopt=AbstractPlus, http://www.ncbi.nlm.nih.gov/pubmed/21427226?dopt=AbstractPlus]. In human sperm, such alkalinization may result from the activation of Hv1, a proton channel [http://www.ncbi.nlm.nih.gov/pubmed/20679352?dopt=AbstractPlus]. Mutations in CatSpers are associated with syndromic and non‐syndromic male infertility [http://www.ncbi.nlm.nih.gov/pubmed/20648059?dopt=AbstractPlus]. In human ejaculated spermatozoa, progesterone (*<*50 nM) potentiates the CatSper current by a non‐genomic mechanism and acts synergistically with intracellular alkalinisation [http://www.ncbi.nlm.nih.gov/pubmed/21412339?dopt=AbstractPlus]. Spermcells frominfertile patients witha deletion inCatSper2gene lack I_CatSper_ and the progesterone response [http://www.ncbi.nlm.nih.gov/pubmed/23530196?dopt=AbstractPlus]. In addition, certain prostaglandins (*e.g.* http://www.guidetopharmacology.org/GRAC/LigandDisplayForward?ligandId=5412, http://www.guidetopharmacology.org/GRAC/LigandDisplayForward?ligandId=1882) also potentiate CatSper mediated currents [http://www.ncbi.nlm.nih.gov/pubmed/21412339?dopt=AbstractPlus,http://www.ncbi.nlm.nih.gov/pubmed/21412339?dopt=AbstractPlus].

In human sperm, CatSper channels are also activated by various small molecules including endocrine disrupting chemicals (EDC) and proposed as a polymodal sensor [http://www.ncbi.nlm.nih.gov/pubmed/22354039?dopt=AbstractPlus].

TPCs are the major Na^+^ conductance in lysosomes; knocking out TPC1 and TPC2 eliminates the Na^+^ conductance and renders the organelle's membrane potential insensitive to changes in [Na^+^] (31). The channels are regulated by luminal pH [http://www.ncbi.nlm.nih.gov/pubmed/24776928?dopt=AbstractPlus], PI(3,5)P2 [http://www.ncbi.nlm.nih.gov/pubmed/23063126?dopt=AbstractPlus], intracellular ATP and extracellular amino acids [http://www.ncbi.nlm.nih.gov/pubmed/23394946?dopt=AbstractPlus]. TPCs are also involved in the NAADP‐activated Ca^2+^ release from lysosomal Ca^2+^ stores [http://www.ncbi.nlm.nih.gov/pubmed/19387438?dopt=AbstractPlus, http://www.ncbi.nlm.nih.gov/pubmed/24277557?dopt=AbstractPlus]. Mice lacking TPCs are viable but have phenotypes including compromised lysosomal pH stability, reduced physical endurance [http://www.ncbi.nlm.nih.gov/pubmed/23394946?dopt=AbstractPlus], resistance to Ebola viral infection [http://www.ncbi.nlm.nih.gov/pubmed/25722412?dopt=AbstractPlus] and fatty liver [http://www.ncbi.nlm.nih.gov/pubmed/25144390?dopt=AbstractPlus]. No major human diseaseassociated TPC mutation has been reported.

### Further reading on CatSper and Two‐Pore channels

Clapham DE *et al.* (2005) International Union of Pharmacology. L. Nomenclature and structure‐Kintzer AF *et al.* (2018) On the structure and mechanism of two‐pore channels. *FEBS J.* **285**: 233‐243 function relationships of CatSper and two‐pore channels. *Pharmacol. Rev.* **57**: 451‐4 [https://www.ncbi.nlm.nih.gov/pubmed/28656706?dopt=AbstractPlus] [https://www.ncbi.nlm.nih.gov/pubmed/16382101?dopt=AbstractPlus]

Grimm C *et al.* (2017) Two‐Pore Channels: Catalyzers of Endolysosomal Transport and Function. *Front Pharmacol* **8**: 45 [https://www.ncbi.nlm.nih.gov/pubmed/28223936?dopt=AbstractPlus]

## http://www.guidetopharmacology.org/GRAC/FamilyDisplayForward?familyId=71

### Overview

**Cyclic nucleotide‐gated (CNG) channels** are responsible for signalling in the primary sensory cells of the vertebrate visual and olfactory systems.

CNG channels arevoltage‐independent cation channels formed as tetramers. Each subunit has 6TM, with the pore‐forming domain between TM5 and TM6. CNG channels were first found in rod photoreceptors [http://www.ncbi.nlm.nih.gov/pubmed/2578616?dopt=AbstractPlus, http://www.ncbi.nlm.nih.gov/pubmed/2481236?dopt=AbstractPlus], where light signals through rhodopsin and transducin to stimulate phosphodiesterase and reduce intracellular http://www.guidetopharmacology.org/GRAC/LigandDisplayForward?ligandId=2347level. This results ina closure ofCNG channels and a reduced ‘dark current’. Similar channels were found in the cilia of olfactory neurons [http://www.ncbi.nlm.nih.gov/pubmed/3027574?dopt=AbstractPlus] and the pineal gland [http://www.ncbi.nlm.nih.gov/pubmed/1719422?dopt=AbstractPlus]. The cyclic nucleotides bind to a domain in the C terminus of the subunit protein: other channels directly binding cyclic nucleotides include HCN, eag and certain plant potassium channels.

**A standardised nomenclature for CNG and HCN channels has been proposed by the NC‐IUPHAR subcommittee on voltage‐gated ion channels** [http://www.ncbi.nlm.nih.gov/pubmed/16382102?dopt=AbstractPlus].

**Table:** 

Nomenclature	http://www.guidetopharmacology.org/GRAC/ObjectDisplayForward?objectId=394	http://www.guidetopharmacology.org/GRAC/ObjectDisplayForward?objectId=395	http://www.guidetopharmacology.org/GRAC/ObjectDisplayForward?objectId=396	http://www.guidetopharmacology.org/GRAC/ObjectDisplayForward?objectId=399
HGNC, UniProt	https://www.genenames.org/data/gene‐symbol‐report/#!/hgnc_id/HGNC:2148, http://www.uniprot.org/uniprot/P29973	https://www.genenames.org/data/gene‐symbol‐report/#!/hgnc_id/HGNC:2149, http://www.uniprot.org/uniprot/Q16280	https://www.genenames.org/data/gene‐symbol‐report/#!/hgnc_id/HGNC:2150, http://www.uniprot.org/uniprot/Q16281	https://www.genenames.org/data/gene‐symbol‐report/#!/hgnc_id/HGNC:2153, http://www.uniprot.org/uniprot/Q9NQW8
Activators	http://www.guidetopharmacology.org/GRAC/LigandDisplayForward?ligandId=2347 (EC_50_ 30 *μ*M) http://www.guidetopharmacology.org/GRAC/LigandDisplayForward?ligandId=2352	http://www.guidetopharmacology.org/GRAC/LigandDisplayForward?ligandId=2347 > http://www.guidetopharmacology.org/GRAC/LigandDisplayForward?ligandId=2352 (EC50 1 *μ*M)	http://www.guidetopharmacology.org/GRAC/LigandDisplayForward?ligandId=2347 (EC_50_ 30 *μ*M) http://www.guidetopharmacology.org/GRAC/LigandDisplayForward?ligandId=2352	–
Inhibitors	–	–	http://www.guidetopharmacology.org/GRAC/LigandDisplayForward?ligandId=2349 (high affinity binding requires presence of CNGB subunits)	–
Channel blockers	http://www.guidetopharmacology.org/GRAC/LigandDisplayForward?ligandId=2313 (Antagonist) (pIC_50_ 6.7) [0mV] [http://www.ncbi.nlm.nih.gov/pubmed/12508052?dopt=AbstractPlus], http://www.guidetopharmacology.org/GRAC/LigandDisplayForward?ligandId=2349 (Antagonist) (p*K*_i_ 4) [−80mV – 80mV] [http://www.ncbi.nlm.nih.gov/pubmed/7682292?dopt=AbstractPlus]	http://www.guidetopharmacology.org/GRAC/LigandDisplayForward?ligandId=2313 (Antagonist) (pIC_50_ 5.6) [0mV] [http://www.ncbi.nlm.nih.gov/pubmed/14981138?dopt=AbstractPlus]	–	http://www.guidetopharmacology.org/GRAC/LigandDisplayForward?ligandId=2349 (Antagonist) (pIC_50_ 5.5) [0mV] [http://www.ncbi.nlm.nih.gov/pubmed/10662822?dopt=AbstractPlus] – Mouse
Functional Characteristics	γ = 25‐30 pS *P*Ca/*P*Na = 3.1	γ = 35 pS *P*Ca/*P*Na = 6.8	γ = 40 pS *P*_Ca_/*P*_Na_ = 10.9	–

### Comments

CNGA1, CNGA2 and CNGA3 express functional channels as homomers. Three additional subunits https://www.genenames.org/data/gene‐symbol‐report/#!/hgnc_id/HGNC:2152 (http://www.uniprot.org/uniprot/Q8IV77), https://www.genenames.org/data/gene‐symbol‐report/#!/hgnc_id/HGNC:2151 (http://www.uniprot.org/uniprot/Q14028) and https://www.genenames.org/data/gene‐symbol‐report/#!/hgnc_id/HGNC:2153 (http://www.uniprot.org/uniprot/Q9NQW8) do not, and are referred to as auxiliary subunits. The subunit composition of the native channels is believed to be as follows. Rod: CNGA1_3_/CNGB1a; Cone: CNGA3_2_/CNGB3_2_; Olfactory neurons: CNGA2_2_/CNGA4/CNGB1b [http://www.ncbi.nlm.nih.gov/pubmed/15134637?dopt=AbstractPlus, http://www.ncbi.nlm.nih.gov/pubmed/12467591?dopt=AbstractPlus, http://www.ncbi.nlm.nih.gov/pubmed/12467592?dopt=AbstractPlus, http://www.ncbi.nlm.nih.gov/pubmed/15134638?dopt=AbstractPlus, http://www.ncbi.nlm.nih.gov/pubmed/12432397?dopt=AbstractPlus].

### Hyperpolarisation‐activated, cyclic nucleotide‐gated (HCN)

The hyperpolarisation‐activated, cyclic nucleotide‐gated (HCN) channels are cation channels that are activated by hyperpolarisation at voltages negative to ˜‐50 mV. The cyclic nucleotides http://www.guidetopharmacology.org/GRAC/LigandDisplayForward?ligandId=2352 and http://www.guidetopharmacology.org/GRAC/LigandDisplayForward?ligandId=2347 directly activate the channels and shift the activation curves of HCN channels to more positive voltages, thereby enhancing channel activity. HCN channels underlie pacemaker currents found in many excitable cells including cardiac cells and neurons [http://www.ncbi.nlm.nih.gov/pubmed/7682045?dopt=AbstractPlus, http://www.ncbi.nlm.nih.gov/pubmed/8815797?dopt=AbstractPlus]. In native cells, these currents have a variety of names, such as *I*_h_, *I*q and*I*_f_. The four known HCN channels have six transmembrane domains and form tetramers.

It is believed that the channels can form heteromers with each other, as has been shown for HCN1 and HCN4 [http://www.ncbi.nlm.nih.gov/pubmed/12702747?dopt=AbstractPlus]. High resolution structural studies of CNG and HCN channels has provided insight into the the gating processes of these channels [http://www.ncbi.nlm.nih.gov/pubmed/28086084?dopt=AbstractPlus, http://www.ncbi.nlm.nih.gov/pubmed/28099415?dopt=AbstractPlus].

**Table:** 

Nomenclature	http://www.guidetopharmacology.org/GRAC/ObjectDisplayForward?objectId=400	http://www.guidetopharmacology.org/GRAC/ObjectDisplayForward?objectId=401	http://www.guidetopharmacology.org/GRAC/ObjectDisplayForward?objectId=402	http://www.guidetopharmacology.org/GRAC/ObjectDisplayForward?objectId=403
HGNC, UniProt	https://www.genenames.org/data/gene‐symbol‐report/#!/hgnc_id/HGNC:4845, http://www.uniprot.org/uniprot/O60741	https://www.genenames.org/data/gene‐symbol‐report/#!/hgnc_id/HGNC:4846, http://www.uniprot.org/uniprot/Q9UL51	https://www.genenames.org/data/gene‐symbol‐report/#!/hgnc_id/HGNC:19183, http://www.uniprot.org/uniprot/Q9P1Z3	https://www.genenames.org/data/gene‐symbol‐report/#!/hgnc_id/HGNC:16882, http://www.uniprot.org/uniprot/Q9Y3Q4
Activators	http://www.guidetopharmacology.org/GRAC/LigandDisplayForward?ligandId=2352 > http://www.guidetopharmacology.org/GRAC/LigandDisplayForward?ligandId=2347 (both weak)	http://www.guidetopharmacology.org/GRAC/LigandDisplayForward?ligandId=2352 > http://www.guidetopharmacology.org/GRAC/LigandDisplayForward?ligandId=2347	–	http://www.guidetopharmacology.org/GRAC/LigandDisplayForward?ligandId=2352 > http://www.guidetopharmacology.org/GRAC/LigandDisplayForward?ligandId=2347
Channel blockers	http://www.guidetopharmacology.org/GRAC/LigandDisplayForward?ligandId=2357 (Antagonist) (pIC_50_ 5.7) [http://www.ncbi.nlm.nih.gov/pubmed/16387796?dopt=AbstractPlus], http://www.guidetopharmacology.org/GRAC/LigandDisplayForward?ligandId=2359 (Antagonist) (pIC_50_ 4.7) [http://www.ncbi.nlm.nih.gov/pubmed/16043489?dopt=AbstractPlus], http://www.guidetopharmacology.org/GRAC/LigandDisplayForward?ligandId=2356 (Antagonist) (pIC_50_ 3.7) [−40mV] [http://www.ncbi.nlm.nih.gov/pubmed/16043489?dopt=AbstractPlus]	http://www.guidetopharmacology.org/GRAC/LigandDisplayForward?ligandId=2357 (Antagonist) (pIC_50_ 5.6) [http://www.ncbi.nlm.nih.gov/pubmed/16387796?dopt=AbstractPlus] – Mouse, http://www.guidetopharmacology.org/GRAC/LigandDisplayForward?ligandId=2359 (Antagonist) (pIC_50_ 4.4) [http://www.ncbi.nlm.nih.gov/pubmed/16043489?dopt=AbstractPlus], http://www.guidetopharmacology.org/GRAC/LigandDisplayForward?ligandId=2356 (Antagonist) (pIC_50_ 3.7) [−40mV] [http://www.ncbi.nlm.nih.gov/pubmed/16043489?dopt=AbstractPlus]	http://www.guidetopharmacology.org/GRAC/LigandDisplayForward?ligandId=2357 (Antagonist) (pIC_50_ 5.7) [http://www.ncbi.nlm.nih.gov/pubmed/16387796?dopt=AbstractPlus], http://www.guidetopharmacology.org/GRAC/LigandDisplayForward?ligandId=2359 (Antagonist) (pIC_50_ 4.5) [http://www.ncbi.nlm.nih.gov/pubmed/16043489?dopt=AbstractPlus], http://www.guidetopharmacology.org/GRAC/LigandDisplayForward?ligandId=2356 (Antagonist) (pIC_50_ 3.8) [−40mV] [http://www.ncbi.nlm.nih.gov/pubmed/16043489?dopt=AbstractPlus]	http://www.guidetopharmacology.org/GRAC/LigandDisplayForward?ligandId=2357 (Antagonist) (pIC_50_ 5.7) [http://www.ncbi.nlm.nih.gov/pubmed/16387796?dopt=AbstractPlus], http://www.guidetopharmacology.org/GRAC/LigandDisplayForward?ligandId=2359 (Antagonist) (pIC_50_ 4.7) [http://www.ncbi.nlm.nih.gov/pubmed/16043489?dopt=AbstractPlus], http://www.guidetopharmacology.org/GRAC/LigandDisplayForward?ligandId=2356 (Antagonist) (pIC_50_ 3.8) [−40mV] [http://www.ncbi.nlm.nih.gov/pubmed/16043489?dopt=AbstractPlus]

### Comments

HCN channels are permeable to both Na^+^ and K^+^ ions, with a Na^+^/K^+^ permeability ratio of about 0.2. Functionally, they differ from each other in terms of time constant of activation with HCN1 the fastest, HCN4 the slowest and HCN2 and HCN3 intermediate. The compounds http://www.guidetopharmacology.org/GRAC/LigandDisplayForward?ligandId=2359 [http://www.ncbi.nlm.nih.gov/pubmed/7693281?dopt=AbstractPlus] and http://www.guidetopharmacology.org/GRAC/LigandDisplayForward?ligandId=2357 [http://www.ncbi.nlm.nih.gov/pubmed/12084770?dopt=AbstractPlus] have proven useful in identifying and studying functional HCN channels in native cells. http://www.guidetopharmacology.org/GRAC/LigandDisplayForward?ligandId=2358 and http://www.guidetopharmacology.org/GRAC/LigandDisplayForward?ligandId=2355 are also useful blocking agents.

### Further reading on Cyclic nucleotide‐regulated channels

James ZM *et al.* (2018) Structural insights into the mechanisms of CNBD channel function. *J. Gen. Physiol.* **150**: 225–244 [https://www.ncbi.nlm.nih.gov/pubmed/29233886?dopt=AbstractPlus]

Michalakis S *et al.* (2018) Retinal Cyclic Nucleotide‐Gated Channels: From Pathophysiology to Ther‐ apy. *Int J Mol Sci* **19**: [https://www.ncbi.nlm.nih.gov/pubmed/29518895?dopt=AbstractPlus]

Podda MV *et al.* (2014) New perspectives in cyclic nucleotide‐mediated functions in the CNS: the emerging role of cyclic nucleotide‐gated (CNG) channels. *Pflugers Arch.* **466**: 1241‐57 [https://www.ncbi.nlm.nih.gov/pubmed/24142069?dopt=AbstractPlus]

Sartiani L *et al.* (2017) The Hyperpolarization‐Activated Cyclic Nucleotide‐Gated Channels: from Biophysics to Pharmacology of a Unique Family of Ion Channels. *Pharmacol. Rev.* **69**: 354‐395 [https://www.ncbi.nlm.nih.gov/pubmed/28878030?dopt=AbstractPlus]

Wahl‐Schott C *et al.* (2014) HCN channels: new roles in sinoatrial node function. *Curr Opin Pharmacol* **15**: 83‐90 [https://www.ncbi.nlm.nih.gov/pubmed/24441197?dopt=AbstractPlus]

## http://www.guidetopharmacology.org/GRAC/FamilyDisplayForward?familyId=133

### Overview

Activation of potassium channels regulates excitability and can control the shape of the action potential waveform. They are present in all cells within the body and can influence processes asdiverse ascognition, muscle contraction and hormone secretion. Potassium channels aresubdivided into families, based on their structural and functional properties. The largest family consists of potassium channels that activated by membrane depolarization, with other families consisting of channels that are either activated by a riseof intracellular calcium ions orareconstitutively active. A standardised nomenclature for potassium channels has been proposed by the **NC‐IUPHAR subcommittees** on potassium channels [http://www.ncbi.nlm.nih.gov/pubmed/16382106?dopt=AbstractPlus, http://www.ncbi.nlm.nih.gov/pubmed/16382104?dopt=AbstractPlus, http://www.ncbi.nlm.nih.gov/pubmed/16382105?dopt=AbstractPlus, http://www.ncbi.nlm.nih.gov/pubmed/16382103?dopt=AbstractPlus], which has placed cloned channels into groups based on gene family and structure of channels that exhibit 6, 4 or 2 transmembrane domains (TM).

## http://www.guidetopharmacology.org/GRAC/FamilyDisplayForward?familyId=69

### Overview

Calcium‐ and sodium‐ activated potassium channels are members of the 6TM family of K channels which comprises the voltage‐gated KV subfamilies, including the KCNQ subfamily, the EAG subfamily (which includes herg channels), the Ca^2+^‐ activated Slo subfamily (actually with 6 or 7TM) and the Ca^2+^and Na^+^‐activated SK subfamily (**nomenclature as agreed by the NC‐IUPHAR Subcommittee on Calcium‐ and sodiumactivated potassium channels** [http://www.ncbi.nlm.nih.gov/pubmed/28267675?dopt=AbstractPlus]). As for the 2TM family, the pore‐forming a subunits form tetramers and heteromeric channels may be formed within subfamilies (*e.g.* KV1.1 with KV1.2; KCNQ2 with KCNQ3).

**Table:** 

Nomenclature	http://www.guidetopharmacology.org/GRAC/ObjectDisplayForward?objectId=380
HGNC, UniProt	https://www.genenames.org/data/gene‐symbol‐report/#!/hgnc_id/HGNC:6284, http://www.uniprot.org/uniprot/Q12791
Activators	http://www.guidetopharmacology.org/GRAC/LigandDisplayForward?ligandId=4271, http://www.guidetopharmacology.org/GRAC/LigandDisplayForward?ligandId=4272
Inhibitors	http://www.guidetopharmacology.org/GRAC/LigandDisplayForward?ligandId=2309 (p*K*_i_ 8.7) [0mV] [http://www.ncbi.nlm.nih.gov/pubmed/8938726?dopt=AbstractPlus] – Mouse
Channel blockers	http://www.guidetopharmacology.org/GRAC/LigandDisplayForward?ligandId=2328, http://www.guidetopharmacology.org/GRAC/LigandDisplayForward?ligandId=4218, http://www.guidetopharmacology.org/GRAC/LigandDisplayForward?ligandId=2343
Functional Characteristics	Maxi K_Ca_

**Table:** 

Nomenclature	http://www.guidetopharmacology.org/GRAC/ObjectDisplayForward?objectId=381	http://www.guidetopharmacology.org/GRAC/ObjectDisplayForward?objectId=382	http://www.guidetopharmacology.org/GRAC/ObjectDisplayForward?objectId=383
HGNC, UniProt	https://www.genenames.org/data/gene‐symbol‐report/#!/hgnc_id/HGNC:6290, http://www.uniprot.org/uniprot/Q92952	https://www.genenames.org/data/gene‐symbol‐report/#!/hgnc_id/HGNC:6291, http://www.uniprot.org/uniprot/Q9H2S1	https://www.genenames.org/data/gene‐symbol‐report/#!/hgnc_id/HGNC:6292, http://www.uniprot.org/uniprot/Q9UGI6
Activators	http://www.guidetopharmacology.org/GRAC/LigandDisplayForward?ligandId=2314 (Agonist) Concentration range: 2×10−^3^M [−80mV] [http://www.ncbi.nlm.nih.gov/pubmed/11134030?dopt=AbstractPlus, http://www.ncbi.nlm.nih.gov/pubmed/20359520?dopt=AbstractPlus], http://www.guidetopharmacology.org/GRAC/LigandDisplayForward?ligandId=2317 (Agonist) Concentration range: 3×10−^8^M‐1×10−^7^M [‐90mV] [http://www.ncbi.nlm.nih.gov/pubmed/15471565?dopt=AbstractPlus]	http://www.guidetopharmacology.org/GRAC/LigandDisplayForward?ligandId=2317 (Agonist) (pEC_50_ 6.2) Concentration range: 3×10−^8^M‐1×10−^7^M [http://www.ncbi.nlm.nih.gov/pubmed/16239218?dopt=AbstractPlus, http://www.ncbi.nlm.nih.gov/pubmed/15471565?dopt=AbstractPlus, http://www.ncbi.nlm.nih.gov/pubmed/20359520?dopt=AbstractPlus], http://www.guidetopharmacology.org/GRAC/LigandDisplayForward?ligandId=2314 (Agonist) (pEC_50_ 3.3) [http://www.ncbi.nlm.nih.gov/pubmed/16239218?dopt=AbstractPlus], http://www.guidetopharmacology.org/GRAC/LigandDisplayForward?ligandId=2314 (Agonist) (pEC_50_ 3) Concentration range: 2×10−^3^M [http://www.ncbi.nlm.nih.gov/pubmed/11181893?dopt=AbstractPlus, http://www.ncbi.nlm.nih.gov/pubmed/11134030?dopt=AbstractPlus] – Rat	http://www.guidetopharmacology.org/GRAC/LigandDisplayForward?ligandId=2314 (Agonist) (pEC_50_ 3.8) [http://www.ncbi.nlm.nih.gov/pubmed/20359520?dopt=AbstractPlus, http://www.ncbi.nlm.nih.gov/pubmed/14978258?dopt=AbstractPlus], http://www.guidetopharmacology.org/GRAC/LigandDisplayForward?ligandId=2317 (Agonist) Concentration range: 3×10−^8^M [http://www.ncbi.nlm.nih.gov/pubmed/15471565?dopt=AbstractPlus]
Inhibitors	http://www.guidetopharmacology.org/GRAC/LigandDisplayForward?ligandId=2320 (pIC_50_ 9.1) [http://www.ncbi.nlm.nih.gov/pubmed/10696100?dopt=AbstractPlus, http://www.ncbi.nlm.nih.gov/pubmed/20359520?dopt=AbstractPlus], http://www.guidetopharmacology.org/GRAC/LigandDisplayForward?ligandId=2311 (pIC_50_ 7.9–8.5) [http://www.ncbi.nlm.nih.gov/pubmed/10683185?dopt=AbstractPlus, http://www.ncbi.nlm.nih.gov/pubmed/15208027?dopt=AbstractPlus]	http://www.guidetopharmacology.org/GRAC/LigandDisplayForward?ligandId=2320 (pIC_50_ 9.6) [http://www.ncbi.nlm.nih.gov/pubmed/11278890?dopt=AbstractPlus, http://www.ncbi.nlm.nih.gov/pubmed/20359520?dopt=AbstractPlus], http://www.guidetopharmacology.org/GRAC/LigandDisplayForward?ligandId=2311 (p*K*_d_ 9.4) [http://www.ncbi.nlm.nih.gov/pubmed/10713270?dopt=AbstractPlus]	http://www.guidetopharmacology.org/GRAC/LigandDisplayForward?ligandId=2311 (pIC_50_ 7.9–9.1) [http://www.ncbi.nlm.nih.gov/pubmed/11369031?dopt=AbstractPlus, http://www.ncbi.nlm.nih.gov/pubmed/14978258?dopt=AbstractPlus], http://www.guidetopharmacology.org/GRAC/LigandDisplayForward?ligandId=2320 (pIC_50_ 8–9) [http://www.ncbi.nlm.nih.gov/pubmed/11278890?dopt=AbstractPlus, http://www.ncbi.nlm.nih.gov/pubmed/20359520?dopt=AbstractPlus]
Channel blockers	http://www.guidetopharmacology.org/GRAC/LigandDisplayForward?ligandId=2343 (pIC_50_ 2.7) [http://www.ncbi.nlm.nih.gov/pubmed/20359520?dopt=AbstractPlus]	http://www.guidetopharmacology.org/GRAC/LigandDisplayForward?ligandId=2343 (pIC_50_ 2.7) [http://www.ncbi.nlm.nih.gov/pubmed/20359520?dopt=AbstractPlus]	http://www.guidetopharmacology.org/GRAC/LigandDisplayForward?ligandId=2343 (pIC_50_ 2.7) [http://www.ncbi.nlm.nih.gov/pubmed/20359520?dopt=AbstractPlus]
Functional Characteristics	SK_Ca_	SK_Ca_	SK_Ca_
Comments	The rat isoform does not form functional channels when expressed alone in cell lines. N‐ or C‐terminal chimeric constructs permit functional channels that are insensitive to http://www.guidetopharmacology.org/GRAC/LigandDisplayForward?ligandId=2311 [http://www.ncbi.nlm.nih.gov/pubmed/20359520?dopt=AbstractPlus]. Heteromeric channels are formed between KCa2.1 and 2.2 subunits that show intermediate sensitivity to http://www.guidetopharmacology.org/GRAC/LigandDisplayForward?ligandId=2311 [http://www.ncbi.nlm.nih.gov/pubmed/25421315?dopt=AbstractPlus].	–	–

**Table:** 

Nomenclature	http://www.guidetopharmacology.org/GRAC/ObjectDisplayForward?objectId=384	http://www.guidetopharmacology.org/GRAC/ObjectDisplayForward?objectId=385	http://www.guidetopharmacology.org/GRAC/ObjectDisplayForward?objectId=386	http://www.guidetopharmacology.org/GRAC/ObjectDisplayForward?objectId=387
HGNC, UniProt	https://www.genenames.org/data/gene‐symbol‐report/#!/hgnc_id/HGNC:6293, http://www.uniprot.org/uniprot/O15554	https://www.genenames.org/data/gene‐symbol‐report/#!/hgnc_id/HGNC:18865, http://www.uniprot.org/uniprot/Q5JUK3	https://www.genenames.org/data/gene‐symbol‐report/#!/hgnc_id/HGNC:18866, http://www.uniprot.org/uniprot/Q6UVM3	https://www.genenames.org/data/gene‐symbol‐report/#!/hgnc_id/HGNC:18867, http://www.uniprot.org/uniprot/A8MYU2
Activators	http://www.guidetopharmacology.org/GRAC/LigandDisplayForward?ligandId=2317 (Agonist) (pEC_50_ 8) [‐90mV] [http://www.ncbi.nlm.nih.gov/pubmed/15471565?dopt=AbstractPlus, http://www.ncbi.nlm.nih.gov/pubmed/20359520?dopt=AbstractPlus], http://www.guidetopharmacology.org/GRAC/LigandDisplayForward?ligandId=8492 (Agonist) (pEC_50_ 7) [http://www.ncbi.nlm.nih.gov/pubmed/24958817?dopt=AbstractPlus], http://www.guidetopharmacology.org/GRAC/LigandDisplayForward?ligandId=2314 (Agonist) (pEC_50_ 4.1–4.5) [‐100mV – ‐50mV] [http://www.ncbi.nlm.nih.gov/pubmed/11134030?dopt=AbstractPlus, http://www.ncbi.nlm.nih.gov/pubmed/10712246?dopt=AbstractPlus]	http://www.guidetopharmacology.org/GRAC/LigandDisplayForward?ligandId=2338 (Agonist) (pEC_50_ 5–6) [http://www.ncbi.nlm.nih.gov/pubmed/16876206?dopt=AbstractPlus] – Rat, http://www.guidetopharmacology.org/GRAC/LigandDisplayForward?ligandId=8494 (Agonist) (pEC_50_ 5.5) [http://www.ncbi.nlm.nih.gov/pubmed/22171093?dopt=AbstractPlus], http://www.guidetopharmacology.org/GRAC/LigandDisplayForward?ligandId=205 (Agonist) (pEC_50_ 5.4) [http://www.ncbi.nlm.nih.gov/pubmed/22171093?dopt=AbstractPlus]	http://www.guidetopharmacology.org/GRAC/LigandDisplayForward?ligandId=2439 (Agonist) (pEC_50_ 8.7) [http://www.ncbi.nlm.nih.gov/pubmed/20176855?dopt=AbstractPlus, http://www.ncbi.nlm.nih.gov/pubmed/22851714?dopt=AbstractPlus]	–
Inhibitors	–	–	–	–
Gating inhibitors	–	http://www.guidetopharmacology.org/GRAC/LigandDisplayForward?ligandId=2337 (pIC_50_ 5–6) [http://www.ncbi.nlm.nih.gov/pubmed/16876206?dopt=AbstractPlus] – Rat	–	–
Channel blockers	http://www.guidetopharmacology.org/GRAC/LigandDisplayForward?ligandId=2328 (pIC_50_ 7.6–8.7) [http://www.ncbi.nlm.nih.gov/pubmed/9730970?dopt=AbstractPlus, http://www.ncbi.nlm.nih.gov/pubmed/9380751?dopt=AbstractPlus]	http://www.guidetopharmacology.org/GRAC/LigandDisplayForward?ligandId=2342 (pIC_50_ 4) [http://www.ncbi.nlm.nih.gov/pubmed/14684870?dopt=AbstractPlus, http://www.ncbi.nlm.nih.gov/pubmed/16876206?dopt=AbstractPlus] – Rat	http://www.guidetopharmacology.org/GRAC/LigandDisplayForward?ligandId=2344 (Inhibition) (pIC_50_ 3) [http://www.ncbi.nlm.nih.gov/pubmed/14684870?dopt=AbstractPlus], http://www.guidetopharmacology.org/GRAC/LigandDisplayForward?ligandId=2342 (Inhibition) Concentration range: 1×10−^3^M [http://www.ncbi.nlm.nih.gov/pubmed/14684870?dopt=AbstractPlus] – Rat	http://www.guidetopharmacology.org/GRAC/LigandDisplayForward?ligandId=2342 Concentration range: 2×10−^5^M [http://www.ncbi.nlm.nih.gov/pubmed/19934650?dopt=AbstractPlus, http://www.ncbi.nlm.nih.gov/pubmed/26045093?dopt=AbstractPlus] – Mouse
Selective channel blockers	http://www.guidetopharmacology.org/GRAC/LigandDisplayForward?ligandId=2336 (Inhibition) (p*K*_d_ 7.6–8) [http://www.ncbi.nlm.nih.gov/pubmed/12939222?dopt=AbstractPlus, http://www.ncbi.nlm.nih.gov/pubmed/10884437?dopt=AbstractPlus], http://www.guidetopharmacology.org/GRAC/LigandDisplayForward?ligandId=2331 (Inhibition) (pIC_50_ 8) [http://www.ncbi.nlm.nih.gov/pubmed/12433690?dopt=AbstractPlus]	–	–	–
Functional Characteristics	IK_Ca_	K_Na_	K_Na_	Sperm pH‐regulated K^+^ current, KSPER

### Further reading on Calcium‐ and sodium‐activated potassium channels

Dopico AM *et al.* (2018) Calcium‐ and voltage‐gated BK channels in vascular smooth muscle. *Pflugers Arch.* **470**: 1271‐1289 [https://www.ncbi.nlm.nih.gov/pubmed/29748711?dopt=AbstractPlus]

Kaczmarek LK *et al.* (2017) International Union of Basic and Clinical Pharmacology. C. Nomenclature and Properties of Calcium‐Activated and Sodium‐Activated Potassium Channels. *Pharmacol. Rev.* **69**: 1‐11 [https://www.ncbi.nlm.nih.gov/pubmed/28267675?dopt=AbstractPlus]

Kshatri AS *et al.* (2018) Physiological Roles and Therapeutic Potential of Ca^2+^ Activated Potassium Channels in the Nervous System. *Front Mol Neurosci* **11**: 258 [https://www.ncbi.nlm.nih.gov/pubmed/30104956?dopt=AbstractPlus]

## http://www.guidetopharmacology.org/GRAC/FamilyDisplayForward?familyId=74

### Overview

The 2TM domain family of K channels are also known as the inward‐rectifier K channel family. This family includes the strong inward‐rectifier K channels (K_ir_2.x) that are constitutively active, the G‐protein‐activated inward‐rectifier K channels (K_ir_3.x) and the ATP‐sensitive K channels (K_ir_6.x, which combine with sulphonylurea receptors (SUR1‐3)). The pore‐forming α subunits form tetramers, and heteromeric channels may be formed within subfamilies (*e.g.* K_ir_3.2 with K_ir_3.3).

**Table:** 

Nomenclature	http://www.guidetopharmacology.org/GRAC/ObjectDisplayForward?objectId=429
HGNC, UniProt	https://www.genenames.org/data/gene‐symbol‐report/#!/hgnc_id/HGNC:6255, http://www.uniprot.org/uniprot/P48048
Ion Selectivity and Conductance	NH_4_^+^ [62pS] > K^+^ [38. pS] > Tl^+^ [21pS] > Rb^+^ [15pS] (Rat) [http://www.ncbi.nlm.nih.gov/pubmed/10736307?dopt=AbstractPlus, http://www.ncbi.nlm.nih.gov/pubmed/7680431?dopt=AbstractPlus]
Channel blockers	http://www.guidetopharmacology.org/GRAC/LigandDisplayForward?ligandId=2383 (Inhibition) (pIC_50_ 8.9) [http://www.ncbi.nlm.nih.gov/pubmed/10572003?dopt=AbstractPlus], http://www.guidetopharmacology.org/GRAC/LigandDisplayForward?ligandId=2344 (Antagonist) (pIC_50_ 2.3–4.2) Concentration range: 1×10^−4^M [*voltage dependent* 0mV – ‐100mV] [http://www.ncbi.nlm.nih.gov/pubmed/7680431?dopt=AbstractPlus, http://www.ncbi.nlm.nih.gov/pubmed/8997197?dopt=AbstractPlus] – Rat, http://www.guidetopharmacology.org/GRAC/LigandDisplayForward?ligandId=2356 (Antagonist) (pIC_50_ 2.9) [*voltage dependent* ‐120mV] [http://www.ncbi.nlm.nih.gov/pubmed/8997197?dopt=AbstractPlus] – Rat
Functional Characteristics	K_ir_1.1 is weakly inwardly rectifying, as compared to classical (strong) inward rectifiers.
Comments	–

**Table:** 

Nomenclature	http://www.guidetopharmacology.org/GRAC/ObjectDisplayForward?objectId=430	http://www.guidetopharmacology.org/GRAC/ObjectDisplayForward?objectId=431	http://www.guidetopharmacology.org/GRAC/ObjectDisplayForward?objectId=432	http://www.guidetopharmacology.org/GRAC/ObjectDisplayForward?objectId=433
HGNC, UniProt	https://www.genenames.org/data/gene‐symbol‐report/#!/hgnc_id/HGNC:6263, http://www.uniprot.org/uniprot/P63252	https://www.genenames.org/data/gene‐symbol‐report/#!/hgnc_id/HGNC:6258, http://www.uniprot.org/uniprot/Q14500	https://www.genenames.org/data/gene‐symbol‐report/#!/hgnc_id/HGNC:6265, http://www.uniprot.org/uniprot/P48050	https://www.genenames.org/data/gene‐symbol‐report/#!/hgnc_id/HGNC:6260, http://www.uniprot.org/uniprot/Q9UNX9
Endogenous activators	http://www.guidetopharmacology.org/GRAC/LigandDisplayForward?ligandId=2387 (Agonist) Concentration range: 1×10^−5^M‐5×10^−5^M [‐30mV] [http://www.ncbi.nlm.nih.gov/pubmed/9486652?dopt=AbstractPlus, http://www.ncbi.nlm.nih.gov/pubmed/15980413?dopt=AbstractPlus, http://www.ncbi.nlm.nih.gov/pubmed/11172809?dopt=AbstractPlus] – Mouse	–	–	Intracellular http://www.guidetopharmacology.org/GRAC/LigandDisplayForward?ligandId=708
Endogenous inhibitors	–	Intracellular http://www.guidetopharmacology.org/GRAC/LigandDisplayForward?ligandId=708 (pIC_50_ 5) [40mV] [http://www.ncbi.nlm.nih.gov/pubmed/8735700?dopt=AbstractPlus]	–	–
Gating inhibitors	–	http://www.guidetopharmacology.org/GRAC/LigandDisplayForward?ligandId=2344 (Antagonist) Concentration range: 5×10^−5^M [‐150mV – ‐50mV] [http://www.ncbi.nlm.nih.gov/pubmed/8083233?dopt=AbstractPlus] – Mouse, http://www.guidetopharmacology.org/GRAC/LigandDisplayForward?ligandId=2356 (Antagonist) Concentration range: 5×10^−6^M‐5×10^−5^M [‐150mV – ‐50mV] [http://www.ncbi.nlm.nih.gov/pubmed/8083233?dopt=AbstractPlus] – Mouse	–	–
Endogenous channel blockers	http://www.guidetopharmacology.org/GRAC/LigandDisplayForward?ligandId=710 (Antagonist) (p*K*_d_ 9.1) [*voltage dependent* 40mV] [http://www.ncbi.nlm.nih.gov/pubmed/8866861?dopt=AbstractPlus, http://www.ncbi.nlm.nih.gov/pubmed/7748552?dopt=AbstractPlus] – Mouse, http://www.guidetopharmacology.org/GRAC/LigandDisplayForward?ligandId=2390 (Antagonist) (p*K*_d_ 8.1) [*voltage dependent* 40mV] [http://www.ncbi.nlm.nih.gov/pubmed/7748552?dopt=AbstractPlus] – Mouse, http://www.guidetopharmacology.org/GRAC/LigandDisplayForward?ligandId=2388 (Antagonist) (p*K*_d_ 5.1) [*voltage dependent* 40mV] [http://www.ncbi.nlm.nih.gov/pubmed/8866861?dopt=AbstractPlus, http://www.ncbi.nlm.nih.gov/pubmed/7748552?dopt=AbstractPlus] – Mouse, Intracellular http://www.guidetopharmacology.org/GRAC/LigandDisplayForward?ligandId=708 (Antagonist) (p*K*_d_ 4.8) [*voltage dependent* 40mV] [http://www.ncbi.nlm.nih.gov/pubmed/7748552?dopt=AbstractPlus] – Mouse	–	Intracellular http://www.guidetopharmacology.org/GRAC/LigandDisplayForward?ligandId=708 (Antagonist) (p*K*_d_ 5) [*voltage dependent* 50mV] [http://www.ncbi.nlm.nih.gov/pubmed/7969496?dopt=AbstractPlus], http://www.guidetopharmacology.org/GRAC/LigandDisplayForward?ligandId=2388 (Antagonist) Concentration range: 5×10^−5^M‐1×10^−3^M [‐80mV – 80mV] [http://www.ncbi.nlm.nih.gov/pubmed/7969496?dopt=AbstractPlus], http://www.guidetopharmacology.org/GRAC/LigandDisplayForward?ligandId=2390 (Antagonist) Concentration range: 2.5×10^−5^M‐1×10^−3^M [‐80mV – 80mV] [http://www.ncbi.nlm.nih.gov/pubmed/7969496?dopt=AbstractPlus], http://www.guidetopharmacology.org/GRAC/LigandDisplayForward?ligandId=710 (Antagonist) Concentration range: 5×10^−5^M‐1×10^−3^M [‐80mV – 80mV] [http://www.ncbi.nlm.nih.gov/pubmed/7969496?dopt=AbstractPlus]	–
Channel blockers	http://www.guidetopharmacology.org/GRAC/LigandDisplayForward?ligandId=2344 (Antagonist) (p*K*_d_ 3.9–5.6) Concentration range: 1×10^−6^M‐1×10^−4^M [*voltage dependent* 0mV – ‐80mV] [http://www.ncbi.nlm.nih.gov/pubmed/11454958?dopt=AbstractPlus] – Mouse, http://www.guidetopharmacology.org/GRAC/LigandDisplayForward?ligandId=2356 (Antagonist) (p*K*_d_ 1.3–4) Concentration range: 3×10^−5^M‐3×10^−4^M [*voltage dependent* 0mV – ‐102mV] [http://www.ncbi.nlm.nih.gov/pubmed/8799888?dopt=AbstractPlus] – Mouse	–	http://www.guidetopharmacology.org/GRAC/LigandDisplayForward?ligandId=2344 (Antagonist) (pIC_50_ 5) Concentration range: 3×10^−6^M‐5×10^−4^M [‐60mV] [http://www.ncbi.nlm.nih.gov/pubmed/8051145?dopt=AbstractPlus, http://www.ncbi.nlm.nih.gov/pubmed/12032359?dopt=AbstractPlus, http://www.ncbi.nlm.nih.gov/pubmed/8034048?dopt=AbstractPlus], http://www.guidetopharmacology.org/GRAC/LigandDisplayForward?ligandId=2356 (Antagonist) (p*K*_i_ 1.3–4.5) Concentration range: 3×10^−6^M‐3×10^−4^M [0mV – ‐130mV] [http://www.ncbi.nlm.nih.gov/pubmed/8051145?dopt=AbstractPlus]	http://www.guidetopharmacology.org/GRAC/LigandDisplayForward?ligandId=2356 (Antagonist) (p*K*_d_ 3–4.1) [*voltage dependent* ‐60mV – ‐100mV] [http://www.ncbi.nlm.nih.gov/pubmed/10942728?dopt=AbstractPlus], http://www.guidetopharmacology.org/GRAC/LigandDisplayForward?ligandId=2344 (Antagonist) (p*K*_d_ 3.3) [*voltage dependent* 0mV] [http://www.ncbi.nlm.nih.gov/pubmed/10942728?dopt=AbstractPlus]
Functional Characteristics	IK_1_ in heart, ‘strong’ inward‐rectifier current	IK_1_ in heart, ‘strong’ inward‐rectifier current	IK_1_ in heart, ‘strong’ inward‐rectifier current	IK_1_ in heart, ‘strong’ inward‐rectifier current
Comments	K_ir_2.1 is also inhibited by intracellular polyamines	K_ir_2.2 is also inhibited by intracellular polyamines	K_ir_2.3 is also inhibited by intracellular polyamines	K_ir_2.4 is also inhibited by intracellular polyamines

**Table:** 

Nomenclature	http://www.guidetopharmacology.org/GRAC/ObjectDisplayForward?objectId=434	http://www.guidetopharmacology.org/GRAC/ObjectDisplayForward?objectId=435	http://www.guidetopharmacology.org/GRAC/ObjectDisplayForward?objectId=436	http://www.guidetopharmacology.org/GRAC/ObjectDisplayForward?objectId=437
HGNC, UniProt	https://www.genenames.org/data/gene‐symbol‐report/#!/hgnc_id/HGNC:6264, http://www.uniprot.org/uniprot/P48549	https://www.genenames.org/data/gene‐symbol‐report/#!/hgnc_id/HGNC:6267, http://www.uniprot.org/uniprot/P48051	https://www.genenames.org/data/gene‐symbol‐report/#!/hgnc_id/HGNC:6270, http://www.uniprot.org/uniprot/Q92806	https://www.genenames.org/data/gene‐symbol‐report/#!/hgnc_id/HGNC:6266, http://www.uniprot.org/uniprot/P48544
Endogenous activators	http://www.guidetopharmacology.org/GRAC/LigandDisplayForward?ligandId=2387 (Agonist) (p*K*_d_ 6.3) Concentration range: 5×10^−5^M [physiological voltage] [http://www.ncbi.nlm.nih.gov/pubmed/9486652?dopt=AbstractPlus]	http://www.guidetopharmacology.org/GRAC/LigandDisplayForward?ligandId=2387 (Agonist) (p*K*_d_ 6.3) Concentration range: 5×10^−5^M [physiological voltage] [http://www.ncbi.nlm.nih.gov/pubmed/9486652?dopt=AbstractPlus]	http://www.guidetopharmacology.org/GRAC/LigandDisplayForward?ligandId=2387 [http://www.ncbi.nlm.nih.gov/pubmed/8688080?dopt=AbstractPlus]	http://www.guidetopharmacology.org/GRAC/LigandDisplayForward?ligandId=2387 [http://www.ncbi.nlm.nih.gov/pubmed/9804555?dopt=AbstractPlus, http://www.ncbi.nlm.nih.gov/pubmed/8688080?dopt=AbstractPlus]
Gating inhibitors	–	http://www.guidetopharmacology.org/GRAC/LigandDisplayForward?ligandId=90 (Antagonist) (pEC_50_ 5.5) [‐70mV] [http://www.ncbi.nlm.nih.gov/pubmed/10780978?dopt=AbstractPlus] – Mouse	–	–
Endogenous channel blockers	–	–		
Channel blockers	http://www.guidetopharmacology.org/GRAC/LigandDisplayForward?ligandId=2383 (Antagonist) (pIC_50_ 7.9) [http://www.ncbi.nlm.nih.gov/pubmed/10572004?dopt=AbstractPlus], http://www.guidetopharmacology.org/GRAC/LigandDisplayForward?ligandId=2344 (Antagonist) (pIC_50_ 4.7) [http://www.ncbi.nlm.nih.gov/pubmed/8234283?dopt=AbstractPlus] – Rat	http://www.guidetopharmacology.org/GRAC/LigandDisplayForward?ligandId=2399 (Antagonist) (pIC_50_ 4.4) [‐70mV] [http://www.ncbi.nlm.nih.gov/pubmed/15150531?dopt=AbstractPlus] – Mouse	–	http://www.guidetopharmacology.org/GRAC/LigandDisplayForward?ligandId=2383 (Antagonist) (pIC_50_ 7.9) [http://www.ncbi.nlm.nih.gov/pubmed/10572004?dopt=AbstractPlus]
Functional Characteristics	G protein‐activated inward‐rectifier current	G protein‐activated inward‐rectifier current	G protein‐activated inward‐rectifier current	G protein‐activated inward‐rectifier current
Comments	K_ir_3.1 is also activated by G_βγ_. K_ir_3.1 is not functional alone. The functional expression of Kir3.1 in *Xenopus oocytes* requires coassembly with the endogenous *Xenopus* K_ir_3.5 subunit. The major functional assembly in the heart is the K_ir_3.1/3.4 heteromultimer, while in the brain it is K_ir_3.1/3.2, K_ir_3.1/3.3 and K_ir_3.2/3.3.	K_ir_3.2 is also activated by G_βγ_. K_ir_3.2 forms functional heteromers with K_ir_3.1/3.3.	K_ir_3.3 is also activated by G_βγ_	K_ir_3.4 is also activated by G_βγ_

**Table:** 

Nomenclature	http://www.guidetopharmacology.org/GRAC/ObjectDisplayForward?objectId=438	http://www.guidetopharmacology.org/GRAC/ObjectDisplayForward?objectId=439	http://www.guidetopharmacology.org/GRAC/ObjectDisplayForward?objectId=440
HGNC, UniProt	https://www.genenames.org/data/gene‐symbol‐report/#!/hgnc_id/HGNC:6256, http://www.uniprot.org/uniprot/P78508	https://www.genenames.org/data/gene‐symbol‐report/#!/hgnc_id/HGNC:6261, http://www.uniprot.org/uniprot/Q99712	https://www.genenames.org/data/gene‐symbol‐report/#!/hgnc_id/HGNC:6262, http://www.uniprot.org/uniprot/Q9NPI9
Channel blockers	http://www.guidetopharmacology.org/GRAC/LigandDisplayForward?ligandId=2344 (Antagonist) Concentration range: 3×10^−6^M‐1×10^−3^M [‐160mV – 60mV] [http://www.ncbi.nlm.nih.gov/pubmed/12456399?dopt=AbstractPlus, http://www.ncbi.nlm.nih.gov/pubmed/7608203?dopt=AbstractPlus, http://www.ncbi.nlm.nih.gov/pubmed/10856114?dopt=AbstractPlus] – Rat, http://www.guidetopharmacology.org/GRAC/LigandDisplayForward?ligandId=2356 (Antagonist) Concentration range: 3×10^−5^M‐3×10^−4^M [‐160mV – 50mV] [http://www.ncbi.nlm.nih.gov/pubmed/7608203?dopt=AbstractPlus] – Rat	http://www.guidetopharmacology.org/GRAC/LigandDisplayForward?ligandId=2344 (Antagonist) Concentration range: 1×10^−5^M‐1×10^−4^M [‐120mV – 100mV] [http://www.ncbi.nlm.nih.gov/pubmed/9882736?dopt=AbstractPlus] – Mouse, http://www.guidetopharmacology.org/GRAC/LigandDisplayForward?ligandId=2356 (Antagonist) Concentration range: 1×10^−5^M‐1×10^−4^M [‐120mV – 100mV] [http://www.ncbi.nlm.nih.gov/pubmed/9882736?dopt=AbstractPlus] – Mouse	http://www.guidetopharmacology.org/GRAC/LigandDisplayForward?ligandId=2344 (Antagonist) Concentration range: 3×10^−3^M [‐120mV – 20mV] [http://www.ncbi.nlm.nih.gov/pubmed/11988170?dopt=AbstractPlus] – Rat
Functional Characteristics	Inward‐rectifier current	Inward‐rectifier current	Weakly inwardly rectifying
Comments	K_ir_3.3 is also activated by G_βγ_	K_ir_3.4 is also activated by G_βγ_	–

**Table:** 

Nomenclature	http://www.guidetopharmacology.org/GRAC/ObjectDisplayForward?objectId=441	http://www.guidetopharmacology.org/GRAC/ObjectDisplayForward?objectId=442	http://www.guidetopharmacology.org/GRAC/ObjectDisplayForward?objectId=443
HGNC, UniProt	https://www.genenames.org/data/gene‐symbol‐report/#!/hgnc_id/HGNC:6269, http://www.uniprot.org/uniprot/Q15842	https://www.genenames.org/data/gene‐symbol‐report/#!/hgnc_id/HGNC:6257, http://www.uniprot.org/uniprot/Q14654	https://www.genenames.org/data/gene‐symbol‐report/#!/hgnc_id/HGNC:6259, http://www.uniprot.org/uniprot/O60928
Associated subunits	SUR1, SUR2A, SUR2B	SUR1, SUR2A, SUR2B	–
Activators	http://www.guidetopharmacology.org/GRAC/LigandDisplayForward?ligandId=2413, http://www.guidetopharmacology.org/GRAC/LigandDisplayForward?ligandId=2409 (Agonist) Concentration range: 2×10^−4^M [‐60mV] [http://www.ncbi.nlm.nih.gov/pubmed/9130167?dopt=AbstractPlus] – Mouse, http://www.guidetopharmacology.org/GRAC/LigandDisplayForward?ligandId=4254, http://www.guidetopharmacology.org/GRAC/LigandDisplayForward?ligandId=2411 (Agonist) Concentration range: 3×10^−4^M [‐60mV – 60mV] [http://www.ncbi.nlm.nih.gov/pubmed/9130167?dopt=AbstractPlus] – Mouse	http://www.guidetopharmacology.org/GRAC/LigandDisplayForward?ligandId=2409 (Agonist) (pEC_50_ 4.2) [physiological voltage] [http://www.ncbi.nlm.nih.gov/pubmed/7502040?dopt=AbstractPlus] – Mouse, http://www.guidetopharmacology.org/GRAC/LigandDisplayForward?ligandId=2413 (Agonist) Concentration range: 3×10^−5^M [‐60mV] [http://www.ncbi.nlm.nih.gov/pubmed/8630239?dopt=AbstractPlus] – Mouse, http://www.guidetopharmacology.org/GRAC/LigandDisplayForward?ligandId=4254, http://www.guidetopharmacology.org/GRAC/LigandDisplayForward?ligandId=2411	–
Inhibitors	http://www.guidetopharmacology.org/GRAC/LigandDisplayForward?ligandId=2414, http://www.guidetopharmacology.org/GRAC/LigandDisplayForward?ligandId=6848	http://www.guidetopharmacology.org/GRAC/LigandDisplayForward?ligandId=2414, http://www.guidetopharmacology.org/GRAC/LigandDisplayForward?ligandId=6848	–
Channel blockers	–	–	http://www.guidetopharmacology.org/GRAC/LigandDisplayForward?ligandId=2344 (Antagonist) (p*K*_i_ 3.2) [*voltage dependent* ‐100mV] [http://www.ncbi.nlm.nih.gov/pubmed/9786970?dopt=AbstractPlus, http://www.ncbi.nlm.nih.gov/pubmed/9620703?dopt=AbstractPlus, http://www.ncbi.nlm.nih.gov/pubmed/11179389?dopt=AbstractPlus, http://www.ncbi.nlm.nih.gov/pubmed/9738472?dopt=AbstractPlus], http://www.guidetopharmacology.org/GRAC/LigandDisplayForward?ligandId=2356 (Antagonist) (p*K*_i_ 1.6) [*voltage dependent* ‐100mV] [http://www.ncbi.nlm.nih.gov/pubmed/9786970?dopt=AbstractPlus, http://www.ncbi.nlm.nih.gov/pubmed/9620703?dopt=AbstractPlus,http://www.ncbi.nlm.nih.gov/pubmed/9738472?dopt=AbstractPlus]
Functional Characteristics	ATP‐sensitive, inward‐rectifier current	ATP‐sensitive, inward‐rectifier current	Inward‐rectifier current

## http://www.guidetopharmacology.org/GRAC/FamilyDisplayForward?familyId=79

### Overview

The 4TM family of K channels mediate many of the background potassium currents observed in native cells. They are open across the physiological voltage‐range and are regulated by a wide array of neurotransmitters and biochemical mediators. The pore‐forming α‐subunit contains two pore loop (P) domains and two subunits assemble to form one ion conduction pathway lined by four P domains. It is important to note that single channels do not have two pores but that each subunit has two P domains in its primary sequence; hence the name two P domain, or K_2_P channels (and not two‐pore channels). Some of the K_2P_ subunits can form heterodimers across subfamilies (*e.g.* K_2P_3.1 with K_2P_9.1). The nomenclature of 4TM K channels in the literature is still a mixture of IUPHAR and common names. The suggested division into subfamilies, described in the http://www.guidetopharmacology.org/GRAC/FamilyIntroductionForward?familyId=79, is based on similarities in both structural and functional properties within subfamilies and this explains the “common abbreviation” nomenclature in the tables below.

**Table:** 

Nomenclature	http://www.guidetopharmacology.org/GRAC/ObjectDisplayForward?objectId=513	http://www.guidetopharmacology.org/GRAC/ObjectDisplayForward?objectId=514	http://www.guidetopharmacology.org/GRAC/ObjectDisplayForward?objectId=515	http://www.guidetopharmacology.org/GRAC/ObjectDisplayForward?objectId=516
Common abbreviation	TWIK1	TREK1	TASK1	TRAAK
HGNC, UniProt	https://www.genenames.org/data/gene‐symbol‐report/#!/hgnc_id/HGNC:6272, http://www.uniprot.org/uniprot/O00180	https://www.genenames.org/data/gene‐symbol‐report/#!/hgnc_id/HGNC:6277, http://www.uniprot.org/uniprot/O95069	https://www.genenames.org/data/gene‐symbol‐report/#!/hgnc_id/HGNC:6278, http://www.uniprot.org/uniprot/O14649	https://www.genenames.org/data/gene‐symbol‐report/#!/hgnc_id/HGNC:6279, http://www.uniprot.org/uniprot/Q9NYG8
Endogenous activators	–	http://www.guidetopharmacology.org/GRAC/LigandDisplayForward?ligandId=2391 (studied at 1‐10 *μ*M) (pEC_50_ 5) [http://www.ncbi.nlm.nih.gov/pubmed/9687497?dopt=AbstractPlus]	–	http://www.guidetopharmacology.org/GRAC/LigandDisplayForward?ligandId=2391 (studied at 1‐10 *μ*M) [http://www.ncbi.nlm.nih.gov/pubmed/9628867?dopt=AbstractPlus]
Activators	–	http://www.guidetopharmacology.org/GRAC/LigandDisplayForward?ligandId=10337 (pEC_50_ 6.1) [http://www.ncbi.nlm.nih.gov/pubmed/29150838?dopt=AbstractPlus], http://www.guidetopharmacology.org/GRAC/LigandDisplayForward?ligandId=10336 (pEC_50_ 5.3) [http://www.ncbi.nlm.nih.gov/pubmed/30089357?dopt=AbstractPlus], http://www.guidetopharmacology.org/GRAC/LigandDisplayForward?ligandId=2503 (studied at 1‐5 mM) Concentration range: 8×10−^3^M [http://www.ncbi.nlm.nih.gov/pubmed/10321245?dopt=AbstractPlus], http://www.guidetopharmacology.org/GRAC/LigandDisplayForward?ligandId=2401 (studied at 1‐5 mM) [http://www.ncbi.nlm.nih.gov/pubmed/10321245?dopt=AbstractPlus], http://www.guidetopharmacology.org/GRAC/LigandDisplayForward?ligandId=2505 (studied at 1‐5 mM) [http://www.ncbi.nlm.nih.gov/pubmed/10321245?dopt=AbstractPlus]	http://www.guidetopharmacology.org/GRAC/LigandDisplayForward?ligandId=2401 (studied at 1‐10 mM) [http://www.ncbi.nlm.nih.gov/pubmed/20519544?dopt=AbstractPlus]	http://www.guidetopharmacology.org/GRAC/LigandDisplayForward?ligandId=2326 (studied at 1‐100 *μ*M) [http://www.ncbi.nlm.nih.gov/pubmed/10779373?dopt=AbstractPlus]
Inhibitors	–	http://www.guidetopharmacology.org/GRAC/LigandDisplayForward?ligandId=208 (pIC_50_ 5.1) [http://www.ncbi.nlm.nih.gov/pubmed/15685212?dopt=AbstractPlus]	–	–
Channel blockers	–	–	http://www.guidetopharmacology.org/GRAC/LigandDisplayForward?ligandId=2506 (pIC_50_ ∼6.2) [http://www.ncbi.nlm.nih.gov/pubmed/11226154?dopt=AbstractPlus], http://www.guidetopharmacology.org/GRAC/LigandDisplayForward?ligandId=2364 (pIC_50_ ∼6.2) [http://www.ncbi.nlm.nih.gov/pubmed/11226154?dopt=AbstractPlus]	–
Functional Characteristics	Background current	Background current	Background current	Background current
Comments	K_2P_1.1 is inhibited by acid pH_o_ external acidification with a pK_a_ 6.7 [http://www.ncbi.nlm.nih.gov/pubmed/20498050?dopt=AbstractPlus]. K_2P_1 forms heterodimers with K_2P_3 and K_2P_9 [http://www.ncbi.nlm.nih.gov/pubmed/23169818?dopt=AbstractPlus].	K_2P_2.1 is also activated by membrane stretch, heat and acid pH_i_ [http://www.ncbi.nlm.nih.gov/pubmed/10835347?dopt=AbstractPlus, http://www.ncbi.nlm.nih.gov/pubmed/10480871?dopt=AbstractPlus]. >K_2P_2 can heterodimerize with >K_2P_4 [http://www.ncbi.nlm.nih.gov/pubmed/27035965?dopt=AbstractPlus] and K_2P_10 [http://www.ncbi.nlm.nih.gov/pubmed/27035963?dopt=AbstractPlus].	Knock‐out of the *kcnk3* gene leads to a prolonged QT interval in mice [http://www.ncbi.nlm.nih.gov/pubmed/21865850?dopt=AbstractPlus] and disrupted development of the adrenal cortex [http://www.ncbi.nlm.nih.gov/pubmed/18034154?dopt=AbstractPlus]. >K_2P_3.1 is inhibited by acid pH_o_ with a pK_a_ of 6.4 [http://www.ncbi.nlm.nih.gov/pubmed/10748056?dopt=AbstractPlus]. >K_2P_3 forms heterodimers with K_2_P1 [http://www.ncbi.nlm.nih.gov/pubmed/23169818?dopt=AbstractPlus] and K_2_P9 [http://www.ncbi.nlm.nih.gov/pubmed/11733509?dopt=AbstractPlus].	K_2P_4 is activated by membrane stretch [http://www.ncbi.nlm.nih.gov/pubmed/9880510?dopt=AbstractPlus], and increased temperature (12 to 20‐fold between 17 and 40°C [http://www.ncbi.nlm.nih.gov/pubmed/15677687?dopt=AbstractPlus]) and can heterodimerize with >K_2P_2 [http://www.ncbi.nlm.nih.gov/pubmed/27035965?dopt=AbstractPlus].

**Table:** 

Nomenclature	http://www.guidetopharmacology.org/GRAC/ObjectDisplayForward?objectId=517	http://www.guidetopharmacology.org/GRAC/ObjectDisplayForward?objectId=518	http://www.guidetopharmacology.org/GRAC/ObjectDisplayForward?objectId=519	http://www.guidetopharmacology.org/GRAC/ObjectDisplayForward?objectId=520	http://www.guidetopharmacology.org/GRAC/ObjectDisplayForward?objectId=521
Common abbreviation	TASK2	TWIK2	–	TASK3	TREK2
HGNC, UniProt	https://www.genenames.org/data/gene‐symbol‐report/#!/hgnc_id/HGNC:6280, http://www.uniprot.org/uniprot/O95279	https://www.genenames.org/data/gene‐symbol‐report/#!/hgnc_id/HGNC:6281, http://www.uniprot.org/uniprot/Q9Y257	https://www.genenames.org/data/gene‐symbol‐report/#!/hgnc_id/HGNC:6282, http://www.uniprot.org/uniprot/Q9Y2U2	https://www.genenames.org/data/gene‐symbol‐report/#!/hgnc_id/HGNC:6283, http://www.uniprot.org/uniprot/Q9NPC2	https://www.genenames.org/data/gene‐symbol‐report/#!/hgnc_id/HGNC:6273, http://www.uniprot.org/uniprot/P57789
Endogenous activators	–	–	–	–	http://www.guidetopharmacology.org/GRAC/LigandDisplayForward?ligandId=2391 (studied at 1‐10 *μ*M) [http://www.ncbi.nlm.nih.gov/pubmed/10880510?dopt=AbstractPlus]
Activators	–	–	–	http://www.guidetopharmacology.org/GRAC/LigandDisplayForward?ligandId=2401 (studied at 1‐5 mM) [http://www.ncbi.nlm.nih.gov/pubmed/11886861?dopt=AbstractPlus]	http://www.guidetopharmacology.org/GRAC/LigandDisplayForward?ligandId=10337 [http://www.ncbi.nlm.nih.gov/pubmed/29150838?dopt=AbstractPlus], http://www.guidetopharmacology.org/GRAC/LigandDisplayForward?ligandId=2401 (studied at 1‐5 mM) [http://www.ncbi.nlm.nih.gov/pubmed/10880510?dopt=AbstractPlus]
Inhibitors	–	–	–	http://www.guidetopharmacology.org/GRAC/LigandDisplayForward?ligandId=2506 (studied at 1‐10 *μ*M) [http://www.ncbi.nlm.nih.gov/pubmed/10747866?dopt=AbstractPlus], http://www.guidetopharmacology.org/GRAC/LigandDisplayForward?ligandId=2364 (studied at 1‐10 *μ*M) [http://www.ncbi.nlm.nih.gov/pubmed/10747866?dopt=AbstractPlus]	–
Functional Characteristics	Background current	Unknown	Unknown	Background current	Background current
Comments	K_2P_5.1 is activated by alkaline pH_o_ [http://www.ncbi.nlm.nih.gov/pubmed/9812978?dopt=AbstractPlus]. Knockout of the kcnk5 gene in mice is associated with metabolic acidosis, hyponatremia and hypotension due to impaired bicarbonate handling in the kidney [http://www.ncbi.nlm.nih.gov/pubmed/15141089?dopt=AbstractPlus], as well as deafness [http://www.ncbi.nlm.nih.gov/pubmed/26549439?dopt=AbstractPlus]. The T108P mutation is associated with Balkan Endemic Nephropathy in humans [http://www.ncbi.nlm.nih.gov/pubmed/24949484?dopt=AbstractPlus].	–	–	K_2P_9.1 is also inhibited by acid pH_o_ with a pK_a_ of 6 [http://www.ncbi.nlm.nih.gov/pubmed/10747866?dopt=AbstractPlus]. Imprinting of the *KCNK9* gene is associated with Birk Barel syndrome [http://www.ncbi.nlm.nih.gov/pubmed/18678320?dopt=AbstractPlus]. K_2P_9 can form heterodimers with >K_2P_1 [http://www.ncbi.nlm.nih.gov/pubmed/23169818?dopt=AbstractPlus] or K_2P_3 [http://www.ncbi.nlm.nih.gov/pubmed/11733509?dopt=AbstractPlus].	K_2P_10.1 is also activated by membrane stretch [http://www.ncbi.nlm.nih.gov/pubmed/10880510?dopt=AbstractPlus] and can heterodimerize with K_2P_2 [http://www.ncbi.nlm.nih.gov/pubmed/27035963?dopt=AbstractPlus].

**Table:** 

Nomenclature	http://www.guidetopharmacology.org/GRAC/ObjectDisplayForward?objectId=522	http://www.guidetopharmacology.org/GRAC/ObjectDisplayForward?objectId=523	http://www.guidetopharmacology.org/GRAC/ObjectDisplayForward?objectId=524	http://www.guidetopharmacology.org/GRAC/ObjectDisplayForward?objectId=525	http://www.guidetopharmacology.org/GRAC/ObjectDisplayForward?objectId=526	http://www.guidetopharmacology.org/GRAC/ObjectDisplayForward?objectId=527
Common abbreviation	THIK2	THIK1	TASK5	TALK1	TALK2	TRESK
HGNC, UniProt	https://www.genenames.org/data/gene‐symbol‐report/#!/hgnc_id/HGNC:6274, http://www.uniprot.org/uniprot/Q9HB15	https://www.genenames.org/data/gene‐symbol‐report/#!/hgnc_id/HGNC:6275, http://www.uniprot.org/uniprot/Q9HB14	https://www.genenames.org/data/gene‐symbol‐report/#!/hgnc_id/HGNC:13814, http://www.uniprot.org/uniprot/Q9H427	https://www.genenames.org/data/gene‐symbol‐report/#!/hgnc_id/HGNC:14464, http://www.uniprot.org/uniprot/Q96T55	https://www.genenames.org/data/gene‐symbol‐report/#!/hgnc_id/HGNC:14465, http://www.uniprot.org/uniprot/Q96T54	https://www.genenames.org/data/gene‐symbol‐report/#!/hgnc_id/HGNC:19439, http://www.uniprot.org/uniprot/Q7Z418
Endogenous inhibitors	–	–	–	–	–	http://www.guidetopharmacology.org/GRAC/LigandDisplayForward?ligandId=2391 (studied at 10‐50 *μ*M) [http://www.ncbi.nlm.nih.gov/pubmed/12754259?dopt=AbstractPlus]
Inhibitors	–	http://www.guidetopharmacology.org/GRAC/LigandDisplayForward?ligandId=2401 (studied at 5 mM) [http://www.ncbi.nlm.nih.gov/pubmed/25148687?dopt=AbstractPlus]	–	–	–	–
Functional Characteristics	Does not function as a homodimer [http://www.ncbi.nlm.nih.gov/pubmed/11060316?dopt=AbstractPlus] but can form a functional heterodimer with K_2P_13 [http://www.ncbi.nlm.nih.gov/pubmed/25148687?dopt=AbstractPlus].	Background current	Unknown	Background current	Background current	Background current
Comments	–	Forms a heterodimer with K_2P_12 [http://www.ncbi.nlm.nih.gov/pubmed/25148687?dopt=AbstractPlus].	–	K_2P_16.1 current is increased by alkaline pH_o_ with a pK_a_ of 7.8 [http://www.ncbi.nlm.nih.gov/pubmed/14985088?dopt=AbstractPlus].	K_2P_17.1 current is increased by alkaline pH_o_ with a pK_a_ of 8.8 [http://www.ncbi.nlm.nih.gov/pubmed/14985088?dopt=AbstractPlus].	A frame‐shift mutation (F139WfsX24) in the *KCNK18* gene, is associated with migraine with aura in humans [http://www.ncbi.nlm.nih.gov/pubmed/20871611?dopt=AbstractPlus].

### Comments

The K_2P_6, K_2P_7.1, K_2P_15.1and K_2P_12.1 subtypes, when expressed in isolation, are nonfunctional. All 4TM channels are insensitive to the classical potassium channel blockers http://www.guidetopharmacology.org/GRAC/LigandDisplayForward?ligandId=2343 and http://www.guidetopharmacology.org/GRAC/LigandDisplayForward?ligandId=2416, but are blocked to varying degrees by Ba^2+^ ions.

## http://www.guidetopharmacology.org/GRAC/FamilyDisplayForward?familyId=81

### Overview

The 6TM family of K channels comprises the voltage‐gated K_V_ subfamilies, the EAG subfamily (which includes hERG channels), the Ca^2+^‐activated Slo subfamily (actually with 7TM termed BK) and the Ca^2+^‐activated SK subfamily. These channels possess a pore‐forming α subunit that comprise tetramers of identical subunits (homomeric) or of different subunits (heteromeric). Heteromeric channels can only be formed within subfamilies (e.g. ‐ K_v_1.1 with K_v_1.2; K_v_7.2 with K_v_7.3). The pharmacology largely, reflects the subunit composition of the functional channel.

**Table:** 

Nomenclature	http://www.guidetopharmacology.org/GRAC/ObjectDisplayForward?objectId=538	http://www.guidetopharmacology.org/GRAC/ObjectDisplayForward?objectId=539	http://www.guidetopharmacology.org/GRAC/ObjectDisplayForward?objectId=540	http://www.guidetopharmacology.org/GRAC/ObjectDisplayForward?objectId=541	http://www.guidetopharmacology.org/GRAC/ObjectDisplayForward?objectId=542	http://www.guidetopharmacology.org/GRAC/ObjectDisplayForward?objectId=543	http://www.guidetopharmacology.org/GRAC/ObjectDisplayForward?objectId=544
HGNC, UniProt	https://www.genenames.org/data/gene‐symbol‐report/#!/hgnc_id/HGNC:6218, http://www.uniprot.org/uniprot/Q09470	https://www.genenames.org/data/gene‐symbol‐report/#!/hgnc_id/HGNC:6220, http://www.uniprot.org/uniprot/P16389	https://www.genenames.org/data/gene‐symbol‐report/#!/hgnc_id/HGNC:6221, http://www.uniprot.org/uniprot/P22001	https://www.genenames.org/data/gene‐symbol‐report/#!/hgnc_id/HGNC:6222, http://www.uniprot.org/uniprot/P22459	https://www.genenames.org/data/gene‐symbol‐report/#!/hgnc_id/HGNC:6224, http://www.uniprot.org/uniprot/P22460	https://www.genenames.org/data/gene‐symbol‐report/#!/hgnc_id/HGNC:6225, http://www.uniprot.org/uniprot/P17658	https://www.genenames.org/data/gene‐symbol‐report/#!/hgnc_id/HGNC:6226, http://www.uniprot.org/uniprot/Q96RP8
Associated subunits	K_v_1.2, K_v_1.4, K_v_β1 and K_v_β2 [http://www.ncbi.nlm.nih.gov/pubmed/10428084?dopt=AbstractPlus]	K_v_1.1, K_v_1.4, K_v_ β1 and K_v_ β2 [http://www.ncbi.nlm.nih.gov/pubmed/10428084?dopt=AbstractPlus]	K_v_1.1, K_v_1.2, K_v_1.4, K_v_1.6, K_v_ β1 and K_v_ β2 [http://www.ncbi.nlm.nih.gov/pubmed/10428084?dopt=AbstractPlus]	K_v_1.1, K_v_1.2, K_v_β1 and K_v_β2 [http://www.ncbi.nlm.nih.gov/pubmed/10428084?dopt=AbstractPlus]	K_v_ β1 and K_v_ β2	K_v_ β1 and K_v_ β2	K_v_ β1 and K_v_ β2
Channel blockers	http://www.guidetopharmacology.org/GRAC/LigandDisplayForward?ligandId=2563 (pEC_50_ 7.7–9) [http://www.ncbi.nlm.nih.gov/pubmed/7517498?dopt=AbstractPlus, http://www.ncbi.nlm.nih.gov/pubmed/1921987?dopt=AbstractPlus] – Rat, http://www.guidetopharmacology.org/GRAC/LigandDisplayForward?ligandId=2547 (Inhibition) (pIC_50_ 8.4) [http://www.ncbi.nlm.nih.gov/pubmed/24878374?dopt=AbstractPlus], http://www.guidetopharmacology.org/GRAC/LigandDisplayForward?ligandId=2343 (Inhibition) (p*K*_d_ 3.5) [http://www.ncbi.nlm.nih.gov/pubmed/7517498?dopt=AbstractPlus] – Mouse	http://www.guidetopharmacology.org/GRAC/LigandDisplayForward?ligandId=2547 (Inhibition) (pIC_50_ 11.2) [http://www.ncbi.nlm.nih.gov/pubmed/24878374?dopt=AbstractPlus], http://www.guidetopharmacology.org/GRAC/LigandDisplayForward?ligandId=2563 (pIC_50_ 7.8–9.4) [http://www.ncbi.nlm.nih.gov/pubmed/7517498?dopt=AbstractPlus, http://www.ncbi.nlm.nih.gov/pubmed/1921987?dopt=AbstractPlus] – Rat, http://www.guidetopharmacology.org/GRAC/LigandDisplayForward?ligandId=2552 (p*K*_d_ 8.7) [http://www.ncbi.nlm.nih.gov/pubmed/7517498?dopt=AbstractPlus] – Rat	http://www.guidetopharmacology.org/GRAC/LigandDisplayForward?ligandId=2547 (pIC_50_ 10–10.3) [http://www.ncbi.nlm.nih.gov/pubmed/8360176?dopt=AbstractPlus, http://www.ncbi.nlm.nih.gov/pubmed/10961988?dopt=AbstractPlus], http://www.guidetopharmacology.org/GRAC/LigandDisplayForward?ligandId=2552 (p*K*_d_ 9) [http://www.ncbi.nlm.nih.gov/pubmed/7517498?dopt=AbstractPlus] – Mouse, http://www.guidetopharmacology.org/GRAC/LigandDisplayForward?ligandId=2332 (pIC_50_ 6.8) [http://www.ncbi.nlm.nih.gov/pubmed/9792177?dopt=AbstractPlus], http://www.guidetopharmacology.org/GRAC/LigandDisplayForward?ligandId=2343 (p*K*_d_ 2) [http://www.ncbi.nlm.nih.gov/pubmed/7517498?dopt=AbstractPlus] – Mouse	http://www.guidetopharmacology.org/GRAC/LigandDisplayForward?ligandId=2416 (pIC_50_ 1.9) [http://www.ncbi.nlm.nih.gov/pubmed/2555158?dopt=AbstractPlus] – Rat	http://www.guidetopharmacology.org/GRAC/LigandDisplayForward?ligandId=2416 (pIC_50_ 4.3) [http://www.ncbi.nlm.nih.gov/pubmed/8508531?dopt=AbstractPlus]	http://www.guidetopharmacology.org/GRAC/LigandDisplayForward?ligandId=2563 (pIC_50_ 7.7) [http://www.ncbi.nlm.nih.gov/pubmed/2347305?dopt=AbstractPlus], http://www.guidetopharmacology.org/GRAC/LigandDisplayForward?ligandId=2343 (pIC_50_ 2.2) [http://www.ncbi.nlm.nih.gov/pubmed/2347305?dopt=AbstractPlus]	http://www.guidetopharmacology.org/GRAC/LigandDisplayForward?ligandId=2552 (pIC_50_ 7.7) [http://www.ncbi.nlm.nih.gov/pubmed/9488722?dopt=AbstractPlus] – Mouse, http://www.guidetopharmacology.org/GRAC/LigandDisplayForward?ligandId=2416 (pIC_50_ 3.6) [http://www.ncbi.nlm.nih.gov/pubmed/9488722?dopt=AbstractPlus] – Mouse
Selective channel blockers	–	–	http://www.guidetopharmacology.org/GRAC/LigandDisplayForward?ligandId=2555 (pIC_50_ 7.1) [http://www.ncbi.nlm.nih.gov/pubmed/10213593?dopt=AbstractPlus]	–	–	–	–
Functional Characteristics	K_V_	K_V_	K_V_	K_A_	K_v_	K_V_	K_V_
Comments	–	–	Resistant to dendrotoxins	Resistant to dendrotoxins	Resistant to external TEA	–	–

**Table:** 

Nomenclature	http://www.guidetopharmacology.org/GRAC/ObjectDisplayForward?objectId=545	http://www.guidetopharmacology.org/GRAC/ObjectDisplayForward?objectId=546	http://www.guidetopharmacology.org/GRAC/ObjectDisplayForward?objectId=547	http://www.guidetopharmacology.org/GRAC/ObjectDisplayForward?objectId=548	http://www.guidetopharmacology.org/GRAC/ObjectDisplayForward?objectId=549	http://www.guidetopharmacology.org/GRAC/ObjectDisplayForward?objectId=550	http://www.guidetopharmacology.org/GRAC/ObjectDisplayForward?objectId=551
HGNC, UniProt	https://www.genenames.org/data/gene‐symbol‐report/#!/hgnc_id/HGNC:6219, http://www.uniprot.org/uniprot/Q16322	https://www.genenames.org/data/gene‐symbol‐report/#!/hgnc_id/HGNC:6231, http://www.uniprot.org/uniprot/Q14721	https://www.genenames.org/data/gene‐symbol‐report/#!/hgnc_id/HGNC:6232, http://www.uniprot.org/uniprot/Q92953	https://www.genenames.org/data/gene‐symbol‐report/#!/hgnc_id/HGNC:6233, http://www.uniprot.org/uniprot/P48547	https://www.genenames.org/data/gene‐symbol‐report/#!/hgnc_id/HGNC:6234, http://www.uniprot.org/uniprot/Q96PR1	https://www.genenames.org/data/gene‐symbol‐report/#!/hgnc_id/HGNC:6235, http://www.uniprot.org/uniprot/Q14003	https://www.genenames.org/data/gene‐symbol‐report/#!/hgnc_id/HGNC:6236, http://www.uniprot.org/uniprot/Q03721
Associated subunits	K_v_ β1 and K_v_ β2	K_v_5.1, K_v_6.1‐6.4, K_v_8.1‐8.2 and K_v_9.1‐9.3	K_v_5.1, K_v_6.1‐6.4, K_v_8.1‐8.2 and K_v_9.1‐9.3	–	–	–	MiRP2 is an associated subunit for K_v_3.4
Channel blockers	http://www.guidetopharmacology.org/GRAC/LigandDisplayForward?ligandId=2416 (pIC_50_ 2.8) [http://www.ncbi.nlm.nih.gov/pubmed/10836990?dopt=AbstractPlus]	http://www.guidetopharmacology.org/GRAC/LigandDisplayForward?ligandId=2343 (Pore blocker) (pIC_50_ 2) [http://www.ncbi.nlm.nih.gov/pubmed/16478442?dopt=AbstractPlus] – Rat	http://www.guidetopharmacology.org/GRAC/LigandDisplayForward?ligandId=2416 (pIC_50_ 2.8) [http://www.ncbi.nlm.nih.gov/pubmed/9612272?dopt=AbstractPlus], http://www.guidetopharmacology.org/GRAC/LigandDisplayForward?ligandId=2343 (pIC_50_ 2.6) [http://www.ncbi.nlm.nih.gov/pubmed/9612272?dopt=AbstractPlus]	http://www.guidetopharmacology.org/GRAC/LigandDisplayForward?ligandId=2416 (pIC_50_ 4.5) [http://www.ncbi.nlm.nih.gov/pubmed/7517498?dopt=AbstractPlus] – Mouse, http://www.guidetopharmacology.org/GRAC/LigandDisplayForward?ligandId=2343 (pIC_50_ 3.7) [http://www.ncbi.nlm.nih.gov/pubmed/7517498?dopt=AbstractPlus] – Mouse	http://www.guidetopharmacology.org/GRAC/LigandDisplayForward?ligandId=2416 (pIC_50_ 4.6) [http://www.ncbi.nlm.nih.gov/pubmed/11790809?dopt=AbstractPlus] – Rat, http://www.guidetopharmacology.org/GRAC/LigandDisplayForward?ligandId=2343 (pIC_50_ 4.2) [http://www.ncbi.nlm.nih.gov/pubmed/11790809?dopt=AbstractPlus] – Rat	http://www.guidetopharmacology.org/GRAC/LigandDisplayForward?ligandId=2343 (pIC_50_ 3.9) [http://www.ncbi.nlm.nih.gov/pubmed/1381835?dopt=AbstractPlus] – Rat	http://www.guidetopharmacology.org/GRAC/LigandDisplayForward?ligandId=2343 (pIC_50_ 3.5) [http://www.ncbi.nlm.nih.gov/pubmed/1378392?dopt=AbstractPlus, http://www.ncbi.nlm.nih.gov/pubmed/1840526?dopt=AbstractPlus] – Rat
Selective channel blockers	–	–	–	–	–	–	http://www.guidetopharmacology.org/GRAC/LigandDisplayForward?ligandId=2578 (pIC_50_ 7.3) [http://www.ncbi.nlm.nih.gov/pubmed/9506974?dopt=AbstractPlus] – Rat
Functional Characteristics	K_V_	K_V_	–	K_V_	K_V_	K_A_	K_A_

**Table:** 

Nomenclature	http://www.guidetopharmacology.org/GRAC/ObjectDisplayForward?objectId=552	http://www.guidetopharmacology.org/GRAC/ObjectDisplayForward?objectId=553	http://www.guidetopharmacology.org/GRAC/ObjectDisplayForward?objectId=554
HGNC, UniProt	https://www.genenames.org/data/gene‐symbol‐report/#!/hgnc_id/HGNC:6237, http://www.uniprot.org/uniprot/Q9NSA2	https://www.genenames.org/data/gene‐symbol‐report/#!/hgnc_id/HGNC:6238, http://www.uniprot.org/uniprot/Q9NZV8	https://www.genenames.org/data/gene‐symbol‐report/#!/hgnc_id/HGNC:6239, http://www.uniprot.org/uniprot/Q9UK17
Associated subunits	KChIP 1‐4, DP66, DPP10	KChIP 1‐4, DPP6, DPP10, K_v_β1, NCS‐1, Navβ1	KChIP 1‐4, DPP6 and DPP10, MinK, MiRPs
Channel blockers	http://www.guidetopharmacology.org/GRAC/LigandDisplayForward?ligandId=2416 (pIC_50_ 2) [http://www.ncbi.nlm.nih.gov/pubmed/10729221?dopt=AbstractPlus]	–	–
Functional Characteristics	K_A_	K_A_	K_A_

**Table:** 

Nomenclature	http://www.guidetopharmacology.org/GRAC/ObjectDisplayForward?objectId=555	http://www.guidetopharmacology.org/GRAC/ObjectDisplayForward?objectId=556	http://www.guidetopharmacology.org/GRAC/ObjectDisplayForward?objectId=557	http://www.guidetopharmacology.org/GRAC/ObjectDisplayForward?objectId=558	http://www.guidetopharmacology.org/GRAC/ObjectDisplayForward?objectId=559
HGNC, UniProt	https://www.genenames.org/data/gene‐symbol‐report/#!/hgnc_id/HGNC:6246, http://www.uniprot.org/uniprot/Q9H3M0	https://www.genenames.org/data/gene‐symbol‐report/#!/hgnc_id/HGNC:6248, http://www.uniprot.org/uniprot/Q9UIX4	https://www.genenames.org/data/gene‐symbol‐report/#!/hgnc_id/HGNC:6249, http://www.uniprot.org/uniprot/Q9UJ96	https://www.genenames.org/data/gene‐symbol‐report/#!/hgnc_id/HGNC:18306, http://www.uniprot.org/uniprot/Q8TAE7	https://www.genenames.org/data/gene‐symbol‐report/#!/hgnc_id/HGNC:19697, http://www.uniprot.org/uniprot/Q8TDN1

**Table:** 

Nomenclature	http://www.guidetopharmacology.org/GRAC/ObjectDisplayForward?objectId=560	http://www.guidetopharmacology.org/GRAC/ObjectDisplayForward?objectId=561	http://www.guidetopharmacology.org/GRAC/ObjectDisplayForward?objectId=562	http://www.guidetopharmacology.org/GRAC/ObjectDisplayForward?objectId=563	http://www.guidetopharmacology.org/GRAC/ObjectDisplayForward?objectId=564
HGNC, UniProt	https://www.genenames.org/data/gene‐symbol‐report/#!/hgnc_id/HGNC:6294, http://www.uniprot.org/uniprot/P51787	https://www.genenames.org/data/gene‐symbol‐report/#!/hgnc_id/HGNC:6296, http://www.uniprot.org/uniprot/O43526	https://www.genenames.org/data/gene‐symbol‐report/#!/hgnc_id/HGNC:6297, http://www.uniprot.org/uniprot/O43525	https://www.genenames.org/data/gene‐symbol‐report/#!/hgnc_id/HGNC:6298, http://www.uniprot.org/uniprot/P56696	https://www.genenames.org/data/gene‐symbol‐report/#!/hgnc_id/HGNC:6299, http://www.uniprot.org/uniprot/Q9NR82
Activators	–	http://www.guidetopharmacology.org/GRAC/LigandDisplayForward?ligandId=2601 (pEC_50_ 5.6) [http://www.ncbi.nlm.nih.gov/pubmed/11466425?dopt=AbstractPlus]	http://www.guidetopharmacology.org/GRAC/LigandDisplayForward?ligandId=5483 (pEC_50_ 8.3) [http://www.ncbi.nlm.nih.gov/pubmed/30021858?dopt=AbstractPlus], http://www.guidetopharmacology.org/GRAC/LigandDisplayForward?ligandId=2601 (pEC_50_ 6.2) [http://www.ncbi.nlm.nih.gov/pubmed/11466425?dopt=AbstractPlus]	http://www.guidetopharmacology.org/GRAC/LigandDisplayForward?ligandId=2601 (pEC_50_ 5.2) [http://www.ncbi.nlm.nih.gov/pubmed/11466425?dopt=AbstractPlus]	http://www.guidetopharmacology.org/GRAC/LigandDisplayForward?ligandId=2601 (pEC_50_ 5) [http://www.ncbi.nlm.nih.gov/pubmed/11890900?dopt=AbstractPlus]
Selective activators	–	–	–	–	http://www.guidetopharmacology.org/GRAC/LigandDisplayForward?ligandId=5483 (pEC_50_ 8.7) [http://www.ncbi.nlm.nih.gov/pubmed/30021858?dopt=AbstractPlus]
Inhibitors	http://www.guidetopharmacology.org/GRAC/LigandDisplayForward?ligandId=2596 (p*K*_d_ 6.1) [http://www.ncbi.nlm.nih.gov/pubmed/10825393?dopt=AbstractPlus], http://www.guidetopharmacology.org/GRAC/LigandDisplayForward?ligandId=2599 (pIC_50_ 4.4) [http://www.ncbi.nlm.nih.gov/pubmed/12690036?dopt=AbstractPlus] – Mouse	http://www.guidetopharmacology.org/GRAC/LigandDisplayForward?ligandId=2596 (pIC_50_ 6.2) [http://www.ncbi.nlm.nih.gov/pubmed/9836639?dopt=AbstractPlus], http://www.guidetopharmacology.org/GRAC/LigandDisplayForward?ligandId=2599 (pIC_50_ 5.3) [http://www.ncbi.nlm.nih.gov/pubmed/9836639?dopt=AbstractPlus]	http://www.guidetopharmacology.org/GRAC/LigandDisplayForward?ligandId=2599 (pIC_50_ 5.4) [http://www.ncbi.nlm.nih.gov/pubmed/9836639?dopt=AbstractPlus] – Rat	http://www.guidetopharmacology.org/GRAC/LigandDisplayForward?ligandId=2596 (pIC_50_ 5.3) [http://www.ncbi.nlm.nih.gov/pubmed/11245603?dopt=AbstractPlus], http://www.guidetopharmacology.org/GRAC/LigandDisplayForward?ligandId=2599 (pIC_50_ 4.9) [http://www.ncbi.nlm.nih.gov/pubmed/11245603?dopt=AbstractPlus]	http://www.guidetopharmacology.org/GRAC/LigandDisplayForward?ligandId=2599 (p*K*_d_ 4.8) [http://www.ncbi.nlm.nih.gov/pubmed/10787416?dopt=AbstractPlus]
Sub/family‐selective inhibitors	–	–	–	–	http://www.guidetopharmacology.org/GRAC/LigandDisplayForward?ligandId=2596 (pIC_50_ 4.2) [http://www.ncbi.nlm.nih.gov/pubmed/10816588?dopt=AbstractPlus]
Channel blockers	–	http://www.guidetopharmacology.org/GRAC/LigandDisplayForward?ligandId=2343 (pIC_50_ 3.5–3.9) [http://www.ncbi.nlm.nih.gov/pubmed/10711337?dopt=AbstractPlus, http://www.ncbi.nlm.nih.gov/pubmed/10953053?dopt=AbstractPlus]	–	http://www.guidetopharmacology.org/GRAC/LigandDisplayForward?ligandId=2343 (pIC_50_ 1.3) [http://www.ncbi.nlm.nih.gov/pubmed/18786918?dopt=AbstractPlus]	–
Functional Characteristics	cardiac IK_5_	M current as a heteromer between K_V_7.2 and K_V_7.3	M current as heteromeric K_V_7.2/K_V_7.3 or K_V_7.3/K_V_7.5	–	M current as heteromeric K_V_7.3/K_V_7.5

**Table:** 

Nomenclature	http://www.guidetopharmacology.org/GRAC/ObjectDisplayForward?objectId=565	http://www.guidetopharmacology.org/GRAC/ObjectDisplayForward?objectId=566	http://www.guidetopharmacology.org/GRAC/ObjectDisplayForward?objectId=567	http://www.guidetopharmacology.org/GRAC/ObjectDisplayForward?objectId=568	http://www.guidetopharmacology.org/GRAC/ObjectDisplayForward?objectId=569	http://www.guidetopharmacology.org/GRAC/ObjectDisplayForward?objectId=570	http://www.guidetopharmacology.org/GRAC/ObjectDisplayForward?objectId=571
HGNC, UniProt	https://www.genenames.org/data/gene‐symbol‐report/#!/hgnc_id/HGNC:18861, http://www.uniprot.org/uniprot/Q6PIU1	https://www.genenames.org/data/gene‐symbol‐report/#!/hgnc_id/HGNC:19698, http://www.uniprot.org/uniprot/Q8TDN2	https://www.genenames.org/data/gene‐symbol‐report/#!/hgnc_id/HGNC:6300, http://www.uniprot.org/uniprot/Q96KK3	https://www.genenames.org/data/gene‐symbol‐report/#!/hgnc_id/HGNC:6301, http://www.uniprot.org/uniprot/Q9ULS6	https://www.genenames.org/data/gene‐symbol‐report/#!/hgnc_id/HGNC:6302, http://www.uniprot.org/uniprot/Q9BQ31	https://www.genenames.org/data/gene‐symbol‐report/#!/hgnc_id/HGNC:6250, http://www.uniprot.org/uniprot/O95259	https://www.genenames.org/data/gene‐symbol‐report/#!/hgnc_id/HGNC:6254, http://www.uniprot.org/uniprot/Q8NCM2

**Table:** 

Nomenclature	http://www.guidetopharmacology.org/GRAC/ObjectDisplayForward?objectId=572	http://www.guidetopharmacology.org/GRAC/ObjectDisplayForward?objectId=573	http://www.guidetopharmacology.org/GRAC/ObjectDisplayForward?objectId=574	http://www.guidetopharmacology.org/GRAC/ObjectDisplayForward?objectId=575	http://www.guidetopharmacology.org/GRAC/ObjectDisplayForward?objectId=576	http://www.guidetopharmacology.org/GRAC/ObjectDisplayForward?objectId=577
HGNC, UniProt	https://www.genenames.org/data/gene‐symbol‐report/#!/hgnc_id/HGNC:6251, http://www.uniprot.org/uniprot/Q12809	https://www.genenames.org/data/gene‐symbol‐report/#!/hgnc_id/HGNC:18862, http://www.uniprot.org/uniprot/Q9H252	https://www.genenames.org/data/gene‐symbol‐report/#!/hgnc_id/HGNC:18863, http://www.uniprot.org/uniprot/Q9NS40	https://www.genenames.org/data/gene‐symbol‐report/#!/hgnc_id/HGNC:18864, http://www.uniprot.org/uniprot/Q96L42	https://www.genenames.org/data/gene‐symbol‐report/#!/hgnc_id/HGNC:6252, http://www.uniprot.org/uniprot/Q9ULD8	https://www.genenames.org/data/gene‐symbol‐report/#!/hgnc_id/HGNC:6253, http://www.uniprot.org/uniprot/Q9UQ05
Associated subunits	minK (KCNE1) and MiRP1 (KCNE2)	minK (KCNE1)	minK (KCNE1)	minK (KCNE1)	minK (KCNE1) and MiRP2 (KCNE3)	–
Channel blockers	http://www.guidetopharmacology.org/GRAC/LigandDisplayForward?ligandId=2603 (pIC_50_ 9) [http://www.ncbi.nlm.nih.gov/pubmed/10376921?dopt=AbstractPlus], http://www.guidetopharmacology.org/GRAC/LigandDisplayForward?ligandId=2608 (pIC_50_ 7.3) [http://www.ncbi.nlm.nih.gov/pubmed/9395068?dopt=AbstractPlus], http://www.guidetopharmacology.org/GRAC/LigandDisplayForward?ligandId=7167 (Inhibition) (pIC_50_ 4) [http://www.ncbi.nlm.nih.gov/pubmed/12873512?dopt=AbstractPlus]	–	–	–	–	–
Sub/family‐selective channel blockers	http://www.guidetopharmacology.org/GRAC/LigandDisplayForward?ligandId=2605 (pIC_50_ 8.1) [http://www.ncbi.nlm.nih.gov/pubmed/9449325?dopt=AbstractPlus]	–	–	–	–	–
Selective channel blockers	http://www.guidetopharmacology.org/GRAC/LigandDisplayForward?ligandId=2604 (Inhibition) (p*K*i 8.2) [http://www.ncbi.nlm.nih.gov/pubmed/17536794?dopt=AbstractPlus], http://www.guidetopharmacology.org/GRAC/LigandDisplayForward?ligandId=7200 (pIC_50_ 7.6–8) [http://www.ncbi.nlm.nih.gov/pubmed/12873512?dopt=AbstractPlus, http://www.ncbi.nlm.nih.gov/pubmed/15266014?dopt=AbstractPlus]	–	–	–	–	–
Functional Characteristics	cardiac IK_R_	–	–	–	–	–
Comments	http://www.guidetopharmacology.org/GRAC/LigandDisplayForward?ligandId=7657 is an activator of K_v_11.1 [http://www.ncbi.nlm.nih.gov/pubmed/15548764?dopt=AbstractPlus].	–	–	–	–	–

### Further reading on Potassium channels

Aguilar‐Bryan L *et al.* (1998) Toward understanding the assembly and structure of KATP channels. *Physiol. Rev.* **78**: 227‐45 [https://www.ncbi.nlm.nih.gov/pubmed/9457174?dopt=AbstractPlus]

Borsotto M *et al.* (2015) Targeting two‐pore domain K(+) channels TREK‐1 and TASK‐3 for the treatment of depression: a new therapeutic concept. *Br. J. Pharmacol.* **172**: 771‐84 [https://www.ncbi.nlm.nih.gov/pubmed/25263033?dopt=AbstractPlus]

Chang PC *et al.* (2015) SK channels and ventricular arrhythmias in heart failure. *Trends Cardiovasc. Med.* **25**: 508‐14 [https://www.ncbi.nlm.nih.gov/pubmed/25743622?dopt=AbstractPlus]

Decher N *et al.* (2017) Stretch‐activated potassium currents in the heart: Focus on TREK‐1 and arrhythmias. *Prog. Biophys. Mol. Biol.* **130**: 223‐232 [https://www.ncbi.nlm.nih.gov/pubmed/28526352?dopt=AbstractPlus]

Feliciangeli S *et al.* (2015) The family of >K_2P_ channels: salient structural and functional properties. *J. Physiol. (Lond.)* **593**: 2587‐603 [https://www.ncbi.nlm.nih.gov/pubmed/25530075?dopt=AbstractPlus]

Foster MN *et al.* (2016) KATP Channels in the Cardiovascular System. *Physiol. Rev.* **96**: 177‐252

[https://www.ncbi.nlm.nih.gov/pubmed/26660852?dopt=AbstractPlus]

Gada K *et al.* (2019) Two‐pore domain potassium channels: emerging targets for novel analgesic drugs: IUPHAR Review 26. *Br J Pharmacol* **176**: 256‐266 [https://www.ncbi.nlm.nih.gov/pubmed/30325008?dopt=AbstractPlus]

Goldstein SA *et al.* (2005) International Union of Pharmacology. LV. Nomenclature and molecular relationships of two‐P potassium channels. *Pharmacol. Rev.* **57**: 527‐40 [https://www.ncbi.nlm.nih.gov/pubmed/16382106?dopt=AbstractPlus]

Greene DL *et al.* (2017) Modulation of Kv7 channels and excitability in the brain. *Cell. Mol. Life Sci.*

**74**: 495‐508 [https://www.ncbi.nlm.nih.gov/pubmed/27645822?dopt=AbstractPlus]

Gutman GA *et al.* (2003) International Union of Pharmacology. XLI. Compendium of voltage‐gated ion channels: potassium channels. *Pharmacol. Rev.* **55**: 583‐6 [https://www.ncbi.nlm.nih.gov/pubmed/14657415?dopt=AbstractPlus]

Kaczmarek LK *et al.* (2017) International Union of Basic and Clinical Pharmacology. C. Nomenclature and Properties of Calcium‐Activated and Sodium‐Activated Potassium Channels. *Pharmacol. Rev.* **69**: 1‐11 [https://www.ncbi.nlm.nih.gov/pubmed/28267675?dopt=AbstractPlus]

Kubo Y *et al.* (2005) International Union of Pharmacology. LIV. Nomenclature and molecular relationships of inwardly rectifying potassium channels. *Pharmacol. Rev.* **57**: 509‐26 [https://www.ncbi.nlm.nih.gov/pubmed/16382105?dopt=AbstractPlus]

Latorre R *et al.* (2017) Molecular Determinants of BK Channel Functional Diversity and Functioning. *Physiol. Rev.* **97**: 39‐87 [https://www.ncbi.nlm.nih.gov/pubmed/27807200?dopt=AbstractPlus]

Niemeyer MI *et al.* (2016) Gating, Regulation, and Structure in >K_2P_ K+ Channels: In Varietate Concordia? *Mol. Pharmacol.* **90**: 309‐17 [https://www.ncbi.nlm.nih.gov/pubmed/27268784?dopt=AbstractPlus]

Poveda JA *et al.* (2017) Towards understanding the molecular basis of ion channel modulation by lipids: Mechanistic models and current paradigms. *Biochim. Biophys. Acta* **1859**: 1507‐1516 [https://www.ncbi.nlm.nih.gov/pubmed/28408206?dopt=AbstractPlus]

Rifkin RA *et al.* (2017) G Protein‐Gated Potassium Channels: A Link to Drug Addiction. *Trends Pharmacol. Sci.* **38**: 378‐392 [https://www.ncbi.nlm.nih.gov/pubmed/28188005?dopt=AbstractPlus]

Taylor KC *et al.* (2017) Regulation of KCNQ/Kv7 family voltage‐gated K^+^ channels by lipids. *Biochim. Biophys. Acta* **1859**: 586‐597 [https://www.ncbi.nlm.nih.gov/pubmed/27818172?dopt=AbstractPlus]

Vivier D *et al.* (2016) Perspectives on the Two‐Pore Domain Potassium Channel TREK‐1 (TWIKRelated K(+) Channel 1). A Novel Therapeutic Target? *J. Med. Chem.* **59**: 5149‐57 [https://www.ncbi.nlm.nih.gov/pubmed/26588045?dopt=AbstractPlus]

Wei AD *et al.* (2005) International Union of Pharmacology. LII. Nomenclature and molecular relationships of calcium‐activated potassium channels. *Pharmacol. Rev.* **57**: 463‐72 [https://www.ncbi.nlm.nih.gov/pubmed/16382103?dopt=AbstractPlus]

Yang KC *et al.* (2016) Mechanisms contributing to myocardial potassium channel diversity, regulation and remodeling. *Trends Cardiovasc. Med.* **26**: 209‐18 [https://www.ncbi.nlm.nih.gov/pubmed/26391345?dopt=AbstractPlus]

## http://www.guidetopharmacology.org/GRAC/FamilyDisplayForward?familyId=125

### Overview

The ryanodine receptors (RyRs) are found on intracellular Ca^2+^ storage/release organelles. The family of RyR genes encodes three highly related Ca^2+^ release channels: RyR1, RyR2 and RyR3, which assemble as large tetrameric structures. These RyR channels are ubiquitously expressed in many types of cells and participate in a variety of important Ca^2+^ signaling phenomena (neurotransmission, secretion, *etc*.). In addition to the three mammalian isoforms described below, various nonmammalian isoforms of the ryanodine receptor have been identified [http://www.ncbi.nlm.nih.gov/pubmed/8874493?dopt=AbstractPlus]. The function of the ryanodine receptor channels may also be influenced by closely associated proteins such as the tacrolimus (FK506)‐binding protein, calmodulin [http://www.ncbi.nlm.nih.gov/pubmed/12707260?dopt=AbstractPlus], triadin, calsequestrin, junctin and sorcin, and by protein kinases and phosphatases. Recent studies solving the structure of the ryanodine receptor have shed light on the structural basis of ryanodine receptor function [see, for example, Samso (2017) [http://www.ncbi.nlm.nih.gov/pubmed/27671094?dopt=AbstractPlus] and Meissner (2017) [http://www.ncbi.nlm.nih.gov/pubmed/29122978?dopt=AbstractPlus]].

**Table:** 

Nomenclature	http://www.guidetopharmacology.org/GRAC/ObjectDisplayForward?objectId=747	http://www.guidetopharmacology.org/GRAC/ObjectDisplayForward?objectId=748	http://www.guidetopharmacology.org/GRAC/ObjectDisplayForward?objectId=749
HGNC, UniProt	https://www.genenames.org/data/gene‐symbol‐report/#!/hgnc_id/HGNC:10483, http://www.uniprot.org/uniprot/P21817	https://www.genenames.org/data/gene‐symbol‐report/#!/hgnc_id/HGNC:10484, http://www.uniprot.org/uniprot/Q92736	https://www.genenames.org/data/gene‐symbol‐report/#!/hgnc_id/HGNC:10485, http://www.uniprot.org/uniprot/Q15413
Endogenous activators	cytosolic http://www.guidetopharmacology.org/GRAC/LigandDisplayForward?ligandId=1713 (endogenous; mM range), cytosolic http://www.guidetopharmacology.org/GRAC/LigandDisplayForward?ligandId=707 (endogenous; *μ*M range), luminal http://www.guidetopharmacology.org/GRAC/LigandDisplayForward?ligandId=707 (endogenous)	cytosolic http://www.guidetopharmacology.org/GRAC/LigandDisplayForward?ligandId=1713 (endogenous; mM range), cytosolic http://www.guidetopharmacology.org/GRAC/LigandDisplayForward?ligandId=707 (endogenous; *μ*M range), luminal http://www.guidetopharmacology.org/GRAC/LigandDisplayForward?ligandId=707 (endogenous)	cytosolic http://www.guidetopharmacology.org/GRAC/LigandDisplayForward?ligandId=1713 (endogenous; mM range), cytosolic http://www.guidetopharmacology.org/GRAC/LigandDisplayForward?ligandId=707 (endogenous; *μ*M range)
Activators	http://www.guidetopharmacology.org/GRAC/LigandDisplayForward?ligandId=407 (pharmacological; mM range), http://www.guidetopharmacology.org/GRAC/LigandDisplayForward?ligandId=4303 (pharmacological; nM ‐ *μ*M range), http://www.guidetopharmacology.org/GRAC/LigandDisplayForward?ligandId=1728 (pharmacological; *μ*M range)	http://www.guidetopharmacology.org/GRAC/LigandDisplayForward?ligandId=407 (pharmacological; mM range), http://www.guidetopharmacology.org/GRAC/LigandDisplayForward?ligandId=4303 (pharmacological; nM ‐ *μ*M range), http://www.guidetopharmacology.org/GRAC/LigandDisplayForward?ligandId=1728 (pharmacological; *μ*M range)	http://www.guidetopharmacology.org/GRAC/LigandDisplayForward?ligandId=407 (pharmacological; mM range), http://www.guidetopharmacology.org/GRAC/LigandDisplayForward?ligandId=4303 (pharmacological; nM ‐ *μ*M range)
Endogenous antagonists	cytosolic http://www.guidetopharmacology.org/GRAC/LigandDisplayForward?ligandId=707 Concentration range: >1×10^−4^M, cytosolic http://www.guidetopharmacology.org/GRAC/LigandDisplayForward?ligandId=708 (mM range)	cytosolic http://www.guidetopharmacology.org/GRAC/LigandDisplayForward?ligandId=707 Concentration range: >1×10^−3^M, cytosolic http://www.guidetopharmacology.org/GRAC/LigandDisplayForward?ligandId=708 (mM range)	cytosolic http://www.guidetopharmacology.org/GRAC/LigandDisplayForward?ligandId=707 Concentration range: >1×10^−3^M, cytosolic http://www.guidetopharmacology.org/GRAC/LigandDisplayForward?ligandId=708 (mM range)
Antagonists	http://www.guidetopharmacology.org/GRAC/LigandDisplayForward?ligandId=4172	–	http://www.guidetopharmacology.org/GRAC/LigandDisplayForward?ligandId=4172
Channel blockers	http://www.guidetopharmacology.org/GRAC/LigandDisplayForward?ligandId=4291, http://www.guidetopharmacology.org/GRAC/LigandDisplayForward?ligandId=2432, http://www.guidetopharmacology.org/GRAC/LigandDisplayForward?ligandId=4303 Concentration range: >1×10^−4^M	http://www.guidetopharmacology.org/GRAC/LigandDisplayForward?ligandId=4291, http://www.guidetopharmacology.org/GRAC/LigandDisplayForward?ligandId=2432, http://www.guidetopharmacology.org/GRAC/LigandDisplayForward?ligandId=4303 Concentration range: >1×^10−4^M	http://www.guidetopharmacology.org/GRAC/LigandDisplayForward?ligandId=2432
Functional Characteristics	Ca^2+^: (*P*_Ca_/*P*_K_6) single‐channel conductance: 90 pS (50mM Ca^2+^), 770 pS (200 mM K^+^)	Ca^2+^: (*P*_Ca_/*P*_K_6) single‐channel conductance: 90 pS (50mM Ca^2+^), 720 pS (210 mM K^+^)	Ca^2+^: (*P*_Ca_/*P*_K_6) single‐channel conductance: 140 pS (50mM Ca^2+^), 777 pS (250 mM K^+^)
Comments	RyR1 is also activated by depolarisation *via* DHP receptor, calmodulin at low cytosolic Ca^2+^ concentrations, CaM kinase and PKA; antagonised by calmodulin at high cytosolic Ca^2+^ concentrations	RyR2 is also activated by CaM kinase and PKA; antagonised by calmodulin at high cytosolic Ca^2+^ concentrations	RyR3 is also activated by calmodulin at low cytosolic Ca^2+^ concentrations; antagonised by calmodulin at high cytosolic Ca^2+^ concentrations

### Comments

The modulators of channel function included in this table are those most commonly used to identify ryanodine‐sensitive Ca^2+^ release pathways. Numerous other modulators of ryanodine receptor/channel function can be found in the reviews listed below. The absence of a modulator of a particular isoform of receptor indicates that the action of that modulator has not been determined, not that it is without effect. The potential role of cyclic ADP ribose as an endogenous regulator of ryanodine receptor channels is controversial. A region of RyR likely to be involved in ion translocation and selection has been identified [http://www.ncbi.nlm.nih.gov/pubmed/10920015?dopt=AbstractPlus, http://www.ncbi.nlm.nih.gov/pubmed/10473538?dopt=AbstractPlus].

### Further reading on Ryanodine receptors

Dulhunty AF *et al.* (2017) Physiology and Pharmacology of Ryanodine Receptor Calcium Release Channels. *Adv. Pharmacol.* **79**: 287‐324 https://www.ncbi.nlm.nih.gov/pubmed/28528672?dopt=AbstractPlus

Meissner G. (2017) The structural basis of ryanodine receptor ion channel function. *J. Gen. Physiol.* **149**: 1065‐1089 https://www.ncbi.nlm.nih.gov/pubmed/29122978?dopt=AbstractPlus

O'Brien F *et al.* (2015) The ryanodine receptor provides high throughput Ca2+‐release but is precisely regulated by networks of associated proteins: a focus on proteins relevant to phosphorylation. *Biochem. Soc. Trans.* **43**: 426‐33 https://www.ncbi.nlm.nih.gov/pubmed/26009186?dopt=AbstractPlus

Samsó M. (2017) A guide to the 3D structure of the ryanodine receptor type 1 by cryoEM. *Protein Sci.* **26**: 52‐68 [https://www.ncbi.nlm.nih.gov/pubmed/27671094?dopt=AbstractPlus

Van Petegem F. (2015) Ryanodine receptors: allosteric ion channel giants. *J. Mol. Biol.* **427**: 31‐53 [https://www.ncbi.nlm.nih.gov/pubmed/25134758?dopt=AbstractPlus

Zalk R *et al.* (2017) Ca^2+^ Release Channels Join the ’Resolution Revolution’. *Trends Biochem. Sci.* **42**: 543‐555 https://www.ncbi.nlm.nih.gov/pubmed/28499500?dopt=AbstractPlus

## http://www.guidetopharmacology.org/GRAC/FamilyDisplayForward?familyId=78

### Overview

The TRP superfamily of channels (**nomenclature as agreed by NC‐IUPHAR** [http://www.ncbi.nlm.nih.gov/pubmed/14657417?dopt=AbstractPlus, http://www.ncbi.nlm.nih.gov/pubmed/20716668?dopt=AbstractPlus]), whose founder member is the *Drosophila* Trp channel, exists in mammals as six families; TRPC, TRPM, TRPV, TRPA, TRPP and TRPML based on amino acid homologies. TRP subunits contain six putative transmembrane domains and assemble as homo‐ or hetero‐tetramers to form cation selective channels with diverse modes of activation and varied permeation properties (reviewed by [http://www.ncbi.nlm.nih.gov/pubmed/16460288?dopt=AbstractPlus]). Established, or potential, physiological functions of the individual members of the TRP families are discussed in detail in the recommended reviews and in a number of books [http://www.ncbi.nlm.nih.gov/pubmed/29356469?dopt=AbstractPlus, http://www.ncbi.nlm.nih.gov/pubmed/21290328?dopt=AbstractPlus, http://www.ncbi.nlm.nih.gov/pubmed/25296415?dopt=AbstractPlus, http://www.ncbi.nlm.nih.gov/pubmed/22593967?dopt=AbstractPlus]. The established, or potential, involvement of TRP channels in disease is reviewed in [http://www.ncbi.nlm.nih.gov/pubmed/17138610?dopt=AbstractPlus, http://www.ncbi.nlm.nih.gov/pubmed/17368864?dopt=AbstractPlus] and [http://www.ncbi.nlm.nih.gov/pubmed/20127491?dopt=AbstractPlus], together with a special edition of *Biochemica et Biophysica Acta* on the subject [http://www.ncbi.nlm.nih.gov/pubmed/17368864?dopt=AbstractPlus]. Additional disease related reviews, for pain [http://www.ncbi.nlm.nih.gov/pubmed/28924972?dopt=AbstractPlus], stroke [http://www.ncbi.nlm.nih.gov/pubmed/25502473?dopt=AbstractPlus], sensation and inflammation [http://www.ncbi.nlm.nih.gov/pubmed/25361914?dopt=AbstractPlus], itch [http://www.ncbi.nlm.nih.gov/pubmed/24830011?dopt=AbstractPlus], and airway disease [http://www.ncbi.nlm.nih.gov/pubmed/24286227?dopt=AbstractPlus, http://www.ncbi.nlm.nih.gov/pubmed/22820171?dopt=AbstractPlus], are available. The pharmacology of most TRP channels has been advanced in recent years. Broad spectrum agents are listed in the tables along with more selective, or recently recognised, ligands that are flagged by the inclusion of a primary reference. See Rubaiy (2019) for a review of pharmacological tools for TRPC1/C4/C5 channels [http://www.ncbi.nlm.nih.gov/pubmed/30656647?dopt=AbstractPlus]. Most TRP channels are regulated by phosphoinostides such as PtIns(4,5)P_2_ although the effects reported are often complex, occasionally contradictory, and likely to be dependent upon experimental conditions, such as intracellular ATP levels (reviewed by [http://www.ncbi.nlm.nih.gov/pubmed/18923420?dopt=AbstractPlus, http://www.ncbi.nlm.nih.gov/pubmed/19376575?dopt=AbstractPlus, http://www.ncbi.nlm.nih.gov/pubmed/17395625?dopt=AbstractPlus]). Such regulation is generally not included in the tables.When thermosensitivity is mentioned, it refers specifically to a high Q10 of gating, often in the range of 10‐ 30, but does not necessarily imply that the channel's function is to act as a ’hot’ or ’cold’ sensor. In general, the search for TRP activators has led to many claims for temperature sensing, mechanosensation, and lipid sensing. All proteins are of course sensitive to energies of binding, mechanical force, and temperature, but the issue is whether the proposed input is within a physiologically relevant range resulting in a response.

### TRPA (ankyrin) family

TRPA1 is the sole mammalian member of this group (reviewed by [http://www.ncbi.nlm.nih.gov/pubmed/17217068?dopt=AbstractPlus]). TRPA1 activation of sensory neurons contribute to nociception [http://www.ncbi.nlm.nih.gov/pubmed/14712238?dopt=AbstractPlus, http://www.ncbi.nlm.nih.gov/pubmed/17686976?dopt=AbstractPlus, http://www.ncbi.nlm.nih.gov/pubmed/12654248?dopt=AbstractPlus]. Pungent chemicals such as mustard oil (AITC), http://www.guidetopharmacology.org/GRAC/LigandDisplayForward?ligandId=2419, and http://www.guidetopharmacology.org/GRAC/LigandDisplayForward?ligandId=2423 activate TRPA1 by modification of free thiol groups of cysteine side chains, especially those located in its amino terminus [http://www.ncbi.nlm.nih.gov/pubmed/16564016?dopt=AbstractPlus, http://www.ncbi.nlm.nih.gov/pubmed/17164327?dopt=AbstractPlus, http://www.ncbi.nlm.nih.gov/pubmed/15916949?dopt=AbstractPlus, http://www.ncbi.nlm.nih.gov/pubmed/17942735?dopt=AbstractPlus]. Alkenals with α, β‐unsaturated bonds, such as propenal (http://www.guidetopharmacology.org/GRAC/LigandDisplayForward?ligandId=2418), butenal (http://www.guidetopharmacology.org/GRAC/LigandDisplayForward?ligandId=6288), and http://www.guidetopharmacology.org/GRAC/LigandDisplayForward?ligandId=2417 can react with free thiols *via* Michael addition and can activate TRPA1. However, potency appears to weaken as carbon chain length increases [http://www.ncbi.nlm.nih.gov/pubmed/18568077?dopt=AbstractPlus, http://www.ncbi.nlm.nih.gov/pubmed/16564016?dopt=AbstractPlus]. Covalent modification leads to sustained activation of TRPA1. Chemicals including http://www.guidetopharmacology.org/GRAC/LigandDisplayForward?ligandId=2497, menthol, and local anesthetics reversibly activate TRPA1 by non‐covalent binding [http://www.ncbi.nlm.nih.gov/pubmed/17855602?dopt=AbstractPlus, http://www.ncbi.nlm.nih.gov/pubmed/21861907?dopt=AbstractPlus, http://www.ncbi.nlm.nih.gov/pubmed/16192383?dopt=AbstractPlus, http://www.ncbi.nlm.nih.gov/pubmed/16617338?dopt=AbstractPlus]. TRPA1 is not mechanosensitive under physiological conditions, but can be activated by cold temperatures [http://www.ncbi.nlm.nih.gov/pubmed/21068322?dopt=AbstractPlus, http://www.ncbi.nlm.nih.gov/pubmed/19144922?dopt=AbstractPlus]. The electron cryo‐EM structure of TRPA1 [http://www.ncbi.nlm.nih.gov/pubmed/25855297?dopt=AbstractPlus] indicates that it is a 6‐TM homotetramer. Each subunit of the channel contains two short ‘pore helices’ pointing into the ion selectivity filter, which is big enough to allow permeation of partially hydrated Ca^2+^ ions.

**Table:** 

Nomenclature	http://www.guidetopharmacology.org/GRAC/ObjectDisplayForward?objectId=485
HGNC, UniProt	https://www.genenames.org/data/gene‐symbol‐report/#!/hgnc_id/HGNC:497, http://www.uniprot.org/uniprot/O75762
Chemical activators	Isothiocyanates (covalent) and 1,4‐dihydropyridines (non‐covalent)
Oxidative stress compounds	4‐oxo‐nonenal: pEC_50_ 5.7, H2O2 : pEC_50_ 3.6 (Mouse) [http://www.ncbi.nlm.nih.gov/pubmed/18322093?dopt=AbstractPlus, http://www.ncbi.nlm.nih.gov/pubmed/18364033?dopt=AbstractPlus]
Physical activators	Cooling (*<*17°C) (disputed) [http://www.ncbi.nlm.nih.gov/pubmed/14712238?dopt=AbstractPlus, http://www.ncbi.nlm.nih.gov/pubmed/15843607?dopt=AbstractPlus, http://www.ncbi.nlm.nih.gov/pubmed/29539642?dopt=AbstractPlus]
Activators	http://www.guidetopharmacology.org/GRAC/LigandDisplayForward?ligandId=10293 (pEC_50_ 6.4) [http://www.ncbi.nlm.nih.gov/pubmed/18550530?dopt=AbstractPlus], http://www.guidetopharmacology.org/GRAC/LigandDisplayForward?ligandId=2418 (Agonist) (pEC_50_ 5.3) [physiological voltage] [http://www.ncbi.nlm.nih.gov/pubmed/16564016?dopt=AbstractPlus], http://www.guidetopharmacology.org/GRAC/LigandDisplayForward?ligandId=2419 (Agonist) (pEC_50_ 5.1) [physiological voltage] [http://www.ncbi.nlm.nih.gov/pubmed/16103371?dopt=AbstractPlus], http://www.guidetopharmacology.org/GRAC/LigandDisplayForward?ligandId=2424‐http://www.guidetopharmacology.org/GRAC/LigandDisplayForward?ligandId=2424 (Agonist) (pEC_50_ 4.9) [‐60mV] [http://www.ncbi.nlm.nih.gov/pubmed/14712238?dopt=AbstractPlus], http://www.guidetopharmacology.org/GRAC/LigandDisplayForward?ligandId=2585 (non‐covalent) (pEC_50_ 4.8) [‐75mV] [http://www.ncbi.nlm.nih.gov/pubmed/19749751?dopt=AbstractPlus], http://www.guidetopharmacology.org/GRAC/LigandDisplayForward?ligandId=2499 (non‐covalent) (pEC_50_ 4.7) Concentration range: 6.2×10^−6^M‐2.5×10^−5^M [http://www.ncbi.nlm.nih.gov/pubmed/18334983?dopt=AbstractPlus], http://www.guidetopharmacology.org/GRAC/LigandDisplayForward?ligandId=4339 (Agonist) (pEC_50_ 4.6) [http://www.ncbi.nlm.nih.gov/pubmed/17314320?dopt=AbstractPlus], (‐)http://www.guidetopharmacology.org/GRAC/LigandDisplayForward?ligandId=2430 (Partial agonist) (pEC_50_ 4–4.5) [http://www.ncbi.nlm.nih.gov/pubmed/17855602?dopt=AbstractPlus, http://www.ncbi.nlm.nih.gov/pubmed/18815250?dopt=AbstractPlus], http://www.guidetopharmacology.org/GRAC/LigandDisplayForward?ligandId=2423 (Agonist) (pEC_50_ 4.2) [physiological voltage] [http://www.ncbi.nlm.nih.gov/pubmed/15046718?dopt=AbstractPlus] – Mouse, http://www.guidetopharmacology.org/GRAC/LigandDisplayForward?ligandId=2429 (Agonist) Concentration range: 1×10^−4^M [physiological voltage] [http://www.ncbi.nlm.nih.gov/pubmed/12654248?dopt=AbstractPlus] – Mouse
Selective activators	http://www.guidetopharmacology.org/GRAC/LigandDisplayForward?ligandId=10275 (pEC_50_ 9.2) [http://www.ncbi.nlm.nih.gov/pubmed/26630251?dopt=AbstractPlus], http://www.guidetopharmacology.org/GRAC/LigandDisplayForward?ligandId=4158 (covalent) (pEC_50_ 6.7) [http://www.ncbi.nlm.nih.gov/pubmed/18501939?dopt=AbstractPlus], http://www.guidetopharmacology.org/GRAC/LigandDisplayForward?ligandId=10274 (pEC_50_ 6.3) [http://www.ncbi.nlm.nih.gov/pubmed/24291101?dopt=AbstractPlus], http://www.guidetopharmacology.org/GRAC/LigandDisplayForward?ligandId=4196 (covalent. This level of activity is also observed for rat TRPA1) (pEC_50_ 3.4) [http://www.ncbi.nlm.nih.gov/pubmed/17942735?dopt=AbstractPlus, http://www.ncbi.nlm.nih.gov/pubmed/17686976?dopt=AbstractPlus] – Mouse
Channel blockers	http://www.guidetopharmacology.org/GRAC/LigandDisplayForward?ligandId=4134 (Inhibition) (pIC_50_ 5.5) [http://www.ncbi.nlm.nih.gov/pubmed/18086313?dopt=AbstractPlus], http://www.guidetopharmacology.org/GRAC/LigandDisplayForward?ligandId=2432 (Inhibition) (pIC_50_ 5.5) [‐80mV] [http://www.ncbi.nlm.nih.gov/pubmed/15843607?dopt=AbstractPlus] – Mouse, http://www.guidetopharmacology.org/GRAC/LigandDisplayForward?ligandId=4211 (Inhibition) (pIC_50_ 5.2) [http://www.ncbi.nlm.nih.gov/pubmed/17686976?dopt=AbstractPlus]
Selective channel blockers	http://www.guidetopharmacology.org/GRAC/LigandDisplayForward?ligandId=10276 (Antagonist) (pIC_50_ 7.7) [http://www.ncbi.nlm.nih.gov/pubmed/26942860?dopt=AbstractPlus]
Functional Characteristics	γ = 87–100 pS; conducts mono‐ and di‐valent cations non‐selectively (P_Ca_/PN_a_ = 0.84); outward rectification; activated by elevated intracellular Ca^2+^
Comments	miRNA‐711 is a selective activator of TRPA1 (pEC_50_ 5.0) [http://www.ncbi.nlm.nih.gov/pubmed/30033153?dopt=AbstractPlus].

### Comments

Agents activating TRPA1 in a covalent manner are thiol reactive electrophiles that bind to cysteine and lysine residues within the cytoplasmic domain of the channel [http://www.ncbi.nlm.nih.gov/pubmed/17164327?dopt=AbstractPlus, http://www.ncbi.nlm.nih.gov/pubmed/17237762?dopt=AbstractPlus]. TRPA1 is activated by a wide range of endogenous and exogenous compounds and only a few representative examples arementioned in the table: an exhaustive listing can be found in [http://www.ncbi.nlm.nih.gov/pubmed/20356305?dopt=AbstractPlus]. In addition, TRPA1 is potently activated by intracellular zinc (EC_50_ = 8 nM) [http://www.ncbi.nlm.nih.gov/pubmed/19416844?dopt=AbstractPlus, http://www.ncbi.nlm.nih.gov/pubmed/19202543?dopt=AbstractPlus]. A gain‐of‐function mutation in TRPA1 was found to cause familial episodic pain syndrome [http://www.ncbi.nlm.nih.gov/pubmed/20547126?dopt=AbstractPlus].

### TRPC (canonical) family

Members of the TRPC subfamily (reviewed by [http://www.ncbi.nlm.nih.gov/pubmed/18940894?dopt=AbstractPlus, http://www.ncbi.nlm.nih.gov/pubmed/17486362?dopt=AbstractPlus, http://www.ncbi.nlm.nih.gov/pubmed/21624095?dopt=AbstractPlus, http://www.ncbi.nlm.nih.gov/pubmed/19281310?dopt=AbstractPlus, http://www.ncbi.nlm.nih.gov/pubmed/15975974?dopt=AbstractPlus, http://www.ncbi.nlm.nih.gov/pubmed/19273053?dopt=AbstractPlus, http://www.ncbi.nlm.nih.gov/pubmed/20490539?dopt=AbstractPlus, http://www.ncbi.nlm.nih.gov/pubmed/16133266?dopt=AbstractPlus]) fall into the subgroups outlined below. TRPC2 is a pseudogene in humans. It is generally accepted that all TRPC channels are activated downstream of G_q/11_‐coupled receptors, or receptor tyrosine kinases (reviewed by [http://www.ncbi.nlm.nih.gov/pubmed/12765689?dopt=AbstractPlus, http://www.ncbi.nlm.nih.gov/pubmed/17217081?dopt=AbstractPlus, http://www.ncbi.nlm.nih.gov/pubmed/20716668?dopt=AbstractPlus]). A comprehensive listing of G‐protein coupled receptors that activate TRPC channels is given in [http://www.ncbi.nlm.nih.gov/pubmed/18940894?dopt=AbstractPlus]. Hetero‐oligomeric complexes of TRPC channels and their association with proteins to form signalling complexes are detailed in [http://www.ncbi.nlm.nih.gov/pubmed/17486362?dopt=AbstractPlus] and [http://www.ncbi.nlm.nih.gov/pubmed/17217079?dopt=AbstractPlus]. TRPC channels have frequently been proposed to act as store‐operated channels (SOCs) (or compenents of mulimeric complexes that form SOCs), activated by depletion of intracellular calcium stores (reviewed by [http://www.ncbi.nlm.nih.gov/pubmed/17486362?dopt=AbstractPlus, http://www.ncbi.nlm.nih.gov/pubmed/28900918?dopt=AbstractPlus, http://www.ncbi.nlm.nih.gov/pubmed/21290310?dopt=AbstractPlus, http://www.ncbi.nlm.nih.gov/pubmed/23890115?dopt=AbstractPlus, http://www.ncbi.nlm.nih.gov/pubmed/28900914?dopt=AbstractPlus, http://www.ncbi.nlm.nih.gov/pubmed/16098585?dopt=AbstractPlus, http://www.ncbi.nlm.nih.gov/pubmed/18536932?dopt=AbstractPlus, http://www.ncbi.nlm.nih.gov/pubmed/19061922?dopt=AbstractPlus, http://www.ncbi.nlm.nih.gov/pubmed/19574740?dopt=AbstractPlus]). However, the weight of the evidence is that they are not directly gated by conventional store‐operated mechanisms, as established for Stim‐gated Orai channels. TRPC channels are not mechanically gated in physiologically relevant ranges of force. All members of the TRPC family are blocked by http://www.guidetopharmacology.org/GRAC/LigandDisplayForward?ligandId=2433 and http://www.guidetopharmacology.org/GRAC/LigandDisplayForward?ligandId=2441 [http://www.ncbi.nlm.nih.gov/pubmed/15843919?dopt=AbstractPlus, http://www.ncbi.nlm.nih.gov/pubmed/20932261?dopt=AbstractPlus]. Activation of TRPC channels by lipids is discussed by [http://www.ncbi.nlm.nih.gov/pubmed/21624095?dopt=AbstractPlus]. Important progress has been recently made in TRPC pharmacology [http://www.ncbi.nlm.nih.gov/pubmed/23763262?dopt=AbstractPlus, http://www.ncbi.nlm.nih.gov/pubmed/26030081?dopt=AbstractPlus, http://www.ncbi.nlm.nih.gov/pubmed/29865154?dopt=AbstractPlus, http://www.ncbi.nlm.nih.gov/pubmed/30656647?dopt=AbstractPlus]. TRPC channels regulate a variety of physiological functions and are implicated in many human diseases [http://www.ncbi.nlm.nih.gov/pubmed/23412755?dopt=AbstractPlus, http://www.ncbi.nlm.nih.gov/pubmed/27289383?dopt=AbstractPlus, http://www.ncbi.nlm.nih.gov/pubmed/23054893?dopt=AbstractPlus, http://www.ncbi.nlm.nih.gov/pubmed/26648074?dopt=AbstractPlus].

### TRPC1/C4/C5 subgroup

TRPC1 alone may not form a functional ion channel [http://www.ncbi.nlm.nih.gov/pubmed/25268281?dopt=AbstractPlus]. TRPC4/C5 may be distinguished from other TRP channels by their potentiation by micromolar concentrations of La^3+^. TRPC2 is a pseudogene in humans, but in other mammals appears to be an ion channel localized to microvilli of the vomeronasal organ. It is required for normal sexual behavior in response to pheromones in mice. It may also function in the main olfactory epithelia in mice [571, http://www.ncbi.nlm.nih.gov/pubmed/25001287?dopt=AbstractPlus, http://www.ncbi.nlm.nih.gov/pubmed/25701815?dopt=AbstractPlus, http://www.ncbi.nlm.nih.gov/pubmed/12601176?dopt=AbstractPlus, http://www.ncbi.nlm.nih.gov/pubmed/26157356?dopt=AbstractPlus, http://www.ncbi.nlm.nih.gov/pubmed/20142439?dopt=AbstractPlus, http://www.ncbi.nlm.nih.gov/pubmed/15971083?dopt=AbstractPlus].

### TRPC3/C6/C7 subgroup

All members are activated by diacylglycerol independent of protein kinase C stimulation [http://www.ncbi.nlm.nih.gov/pubmed/20932261?dopt=AbstractPlus].

**Table:** 

Nomenclature	http://www.guidetopharmacology.org/GRAC/ObjectDisplayForward?objectId=486	http://www.guidetopharmacology.org/GRAC/ObjectDisplayForward?objectId=487	http://www.guidetopharmacology.org/GRAC/ObjectDisplayForward?objectId=488	http://www.guidetopharmacology.org/GRAC/ObjectDisplayForward?objectId=489
HGNC, UniProt	https://www.genenames.org/data/gene‐symbol‐report/#!/hgnc_id/HGNC:12333, http://www.uniprot.org/uniprot/P48995	https://www.genenames.org/data/gene‐symbol‐report/#!/hgnc_id/HGNC:12334, –	https://www.genenames.org/data/gene‐symbol‐report/#!/hgnc_id/HGNC:12335, http://www.uniprot.org/uniprot/Q13507	https://www.genenames.org/data/gene‐symbol‐report/#!/hgnc_id/HGNC:12336, http://www.uniprot.org/uniprot/Q9UBN4
Chemical activators	NO‐mediated cysteine S‐nitrosylation	Diacylglycerol (SAG, OAG, DOG): strongly inhibited by Ca^2+^/CaM once activated by DAG [http://www.ncbi.nlm.nih.gov/pubmed/19228965?dopt=AbstractPlus]	diacylglycerols	NO‐mediated cysteine S‐nitrosylation, potentiation by extracellular protons
Physical activators	–	DAG kinase; regulates DAG concentration in vomeronasal sensory neurons	–	–
Endogenous activators	membrane stretch (likely direct)	Intracellular http://www.guidetopharmacology.org/GRAC/LigandDisplayForward?ligandId=707	–	–
Activators		http://www.guidetopharmacology.org/GRAC/LigandDisplayForward?ligandId=2435 (Agonist) Concentration range: 1×10^−4^M [‐80mV] [http://www.ncbi.nlm.nih.gov/pubmed/14642279?dopt=AbstractPlus] – Mouse, http://www.guidetopharmacology.org/GRAC/LigandDisplayForward?ligandId=2437 (Agonist) Concentration range: 1×10^−4^M [‐80mV] [http://www.ncbi.nlm.nih.gov/pubmed/14642279?dopt=AbstractPlus] – Mouse	http://www.guidetopharmacology.org/GRAC/LigandDisplayForward?ligandId=10281 (pEC_50_ 7.7) [http://www.ncbi.nlm.nih.gov/pubmed/28395140?dopt=AbstractPlus], http://www.guidetopharmacology.org/GRAC/LigandDisplayForward?ligandId=10278 (Agonist) (pEC_50_ 7.1) [1099]	(‐)http://www.guidetopharmacology.org/GRAC/LigandDisplayForward?ligandId=8372 (Agonist) (pEC_50_ 7.9) [http://www.ncbi.nlm.nih.gov/pubmed/25707820?dopt=AbstractPlus], http://www.guidetopharmacology.org/GRAC/LigandDisplayForward?ligandId=10287 (pEC_50_ 6.9) [http://www.ncbi.nlm.nih.gov/pubmed/29859013?dopt=AbstractPlus], http://www.guidetopharmacology.org/GRAC/LigandDisplayForward?ligandId=2434 (*μ*M range)
Channel blockers	http://www.guidetopharmacology.org/GRAC/LigandDisplayForward?ligandId=2433 (Antagonist) [‐70mV] [http://www.ncbi.nlm.nih.gov/pubmed/11301024?dopt=AbstractPlus], http://www.guidetopharmacology.org/GRAC/LigandDisplayForward?ligandId=2426 (Antagonist) Concentration range: 2×10^−5^M [‐70mV] [http://www.ncbi.nlm.nih.gov/pubmed/8663995?dopt=AbstractPlus], http://www.guidetopharmacology.org/GRAC/LigandDisplayForward?ligandId=2434 (Antagonist) Concentration range: 1×10^−4^M [‐70mV] [http://www.ncbi.nlm.nih.gov/pubmed/11301024?dopt=AbstractPlus]	http://www.guidetopharmacology.org/GRAC/LigandDisplayForward?ligandId=2433 (Antagonist) Concentration range: 5×10^−5^M [‐70mV – 80mV] [http://www.ncbi.nlm.nih.gov/pubmed/14642279?dopt=AbstractPlus] – Mouse, http://www.guidetopharmacology.org/GRAC/LigandDisplayForward?ligandId=5283 (Antagonist) Concentration range: 1×10^−5^M – Mouse	http://www.guidetopharmacology.org/GRAC/LigandDisplayForward?ligandId=10284 (pIC_50_ 7.7) [80mV] [http://www.ncbi.nlm.nih.gov/pubmed/24453217?dopt=AbstractPlus], http://www.guidetopharmacology.org/GRAC/LigandDisplayForward?ligandId=10279 (Antagonist) (pIC_50_ 7.1) [http://www.ncbi.nlm.nih.gov/pubmed/23886683?dopt=AbstractPlus], http://www.guidetopharmacology.org/GRAC/LigandDisplayForward?ligandId=2426 (Antagonist) (pEC_50_ 7) [‐60mV] [http://www.ncbi.nlm.nih.gov/pubmed/10970899?dopt=AbstractPlus], http://www.guidetopharmacology.org/GRAC/LigandDisplayForward?ligandId=10283 (pIC_50_ 6.6) [http://www.ncbi.nlm.nih.gov/pubmed/25847402?dopt=AbstractPlus], http://www.guidetopharmacology.org/GRAC/LigandDisplayForward?ligandId=2438 (Antagonist) (pIC_50_ 6.5) [‐80mV] [http://www.ncbi.nlm.nih.gov/pubmed/15647288?dopt=AbstractPlus], http://www.guidetopharmacology.org/GRAC/LigandDisplayForward?ligandId=4293 (pIC_50_ 6.2) [http://www.ncbi.nlm.nih.gov/pubmed/19289841?dopt=AbstractPlus], http://www.guidetopharmacology.org/GRAC/LigandDisplayForward?ligandId=10282 (Antagonist) (pIC_50_ 6.1) [http://www.ncbi.nlm.nih.gov/pubmed/22862290?dopt=AbstractPlus], http://www.guidetopharmacology.org/GRAC/LigandDisplayForward?ligandId=7091 (p*K*_i_ 5.5) [http://www.ncbi.nlm.nih.gov/pubmed/22530015?dopt=AbstractPlus], http://www.guidetopharmacology.org/GRAC/LigandDisplayForward?ligandId=2434 (Antagonist) (pIC_50_ 5.4) [‐60mV] [http://www.ncbi.nlm.nih.gov/pubmed/10970899?dopt=AbstractPlus], http://www.guidetopharmacology.org/GRAC/LigandDisplayForward?ligandId=10285 (pIC_50_ 5) [http://www.ncbi.nlm.nih.gov/pubmed/25140002?dopt=AbstractPlus], http://www.guidetopharmacology.org/GRAC/LigandDisplayForward?ligandId=2433 (Antagonist) (pIC_50_ 5) [physiological voltage] [http://www.ncbi.nlm.nih.gov/pubmed/15933213?dopt=AbstractPlus], http://www.guidetopharmacology.org/GRAC/LigandDisplayForward?ligandId=2476, http://www.guidetopharmacology.org/GRAC/LigandDisplayForward?ligandId=2441	http://www.guidetopharmacology.org/GRAC/LigandDisplayForward?ligandId=10286 (Antagonist) (pIC_50_ 7.3) [http://www.ncbi.nlm.nih.gov/pubmed/29385160?dopt=AbstractPlus], http://www.guidetopharmacology.org/GRAC/LigandDisplayForward?ligandId=4255 (pIC_50_ 5.5) [http://www.ncbi.nlm.nih.gov/pubmed/21795696?dopt=AbstractPlus], http://www.guidetopharmacology.org/GRAC/LigandDisplayForward?ligandId=10288 (Inhibition) (pIC_50_ 5.3) [http://www.ncbi.nlm.nih.gov/pubmed/25816897?dopt=AbstractPlus], http://www.guidetopharmacology.org/GRAC/LigandDisplayForward?ligandId=10285 (pIC_50_ 5.2) [http://www.ncbi.nlm.nih.gov/pubmed/25140002?dopt=AbstractPlus], http://www.guidetopharmacology.org/GRAC/LigandDisplayForward?ligandId=2434 (mM range), http://www.guidetopharmacology.org/GRAC/LigandDisplayForward?ligandId=2441, http://www.guidetopharmacology.org/GRAC/LigandDisplayForward?ligandId=2439 (Antagonist) Concentration range: 3×10^−5^M [‐60mV] [http://www.ncbi.nlm.nih.gov/pubmed/12388058?dopt=AbstractPlus] – Mouse
Functional Characteristics	It is not yet clear that TRPC1 forms a homomer. It does form heteromers with TRPC4 and TRPC5	γ = 42 pS linear single channel conductance in 150 mM symmetrical Na^+^ in vomeronasal sensory neurons. P_Ca_/P_Na_ = 2.7; permeant to Na^+^, Cs^+^, Ca^2+^, but not NMDG [http://www.ncbi.nlm.nih.gov/pubmed/25001287?dopt=AbstractPlus, http://www.ncbi.nlm.nih.gov/pubmed/26157356?dopt=AbstractPlus]	γ = 66 pS; conducts mono and di‐valent cations non‐selectively (P_Ca_/P_Na_ = 1.6); monovalent cation current suppressed by extracellular Ca^2+^; dual (inward and outward) rectification	γ = 30 –41 pS, conducts mono and di‐valent cations non‐selectively (P_Ca_/P_Na_ = 1.1 – 7.7); dual (inward and outward) rectification

**Table:** 

Nomenclature	http://www.guidetopharmacology.org/GRAC/ObjectDisplayForward?objectId=490	http://www.guidetopharmacology.org/GRAC/ObjectDisplayForward?objectId=491	http://www.guidetopharmacology.org/GRAC/ObjectDisplayForward?objectId=492
HGNC, UniProt	https://www.genenames.org/data/gene‐symbol‐report/#!/hgnc_id/HGNC:12337, http://www.uniprot.org/uniprot/Q9UL62	https://www.genenames.org/data/gene‐symbol‐report/#!/hgnc_id/HGNC:12338, http://www.uniprot.org/uniprot/Q9Y210	https://www.genenames.org/data/gene‐symbol‐report/#!/hgnc_id/HGNC:20754, http://www.uniprot.org/uniprot/Q9HCX4
Chemical activators	NO‐mediated cysteine S‐nitrosylation (disputed), potentiation by extracellular protons	Diacylglycerols	diacylglycerols
Physical activators	Membrane stretch	Membrane stretch	–
Endogenous activators	intracellular http://www.guidetopharmacology.org/GRAC/LigandDisplayForward?ligandId=707 (at negative potentials) (pEC_50_ 6.2), http://www.guidetopharmacology.org/GRAC/LigandDisplayForward?ligandId=2508	http://www.guidetopharmacology.org/GRAC/LigandDisplayForward?ligandId=4103, http://www.guidetopharmacology.org/GRAC/LigandDisplayForward?ligandId=2391, http://www.guidetopharmacology.org/GRAC/LigandDisplayForward?ligandId=2508	–
Activators	(‐)http://www.guidetopharmacology.org/GRAC/LigandDisplayForward?ligandId=8372 (Agonist) (pEC_50_ 8.1) [http://www.ncbi.nlm.nih.gov/pubmed/25707820?dopt=AbstractPlus], http://www.guidetopharmacology.org/GRAC/LigandDisplayForward?ligandId=10287 (pEC_50_ 7.1) [http://www.ncbi.nlm.nih.gov/pubmed/29859013?dopt=AbstractPlus], http://www.guidetopharmacology.org/GRAC/LigandDisplayForward?ligandId=10289 (pEC_50_ 5.8) [http://www.ncbi.nlm.nih.gov/pubmed/28807145?dopt=AbstractPlus], http://www.guidetopharmacology.org/GRAC/LigandDisplayForward?ligandId=2326 (pEC_50_ 5) [http://www.ncbi.nlm.nih.gov/pubmed/24117252?dopt=AbstractPlus], http://www.guidetopharmacology.org/GRAC/LigandDisplayForward?ligandId=7088 (pEC_50_ 4.9) [http://www.ncbi.nlm.nih.gov/pubmed/28807145?dopt=AbstractPlus], http://www.guidetopharmacology.org/GRAC/LigandDisplayForward?ligandId=1056 (pEC_50_ 4.5) [http://www.ncbi.nlm.nih.gov/pubmed/21406603?dopt=AbstractPlus], http://www.guidetopharmacology.org/GRAC/LigandDisplayForward?ligandId=2426 Concentration range: 1×10^−4^M, http://www.guidetopharmacology.org/GRAC/LigandDisplayForward?ligandId=2434 (*μ*M range), http://www.guidetopharmacology.org/GRAC/LigandDisplayForward?ligandId=2525 Concentration range: 5×10^−6^M, http://www.guidetopharmacology.org/GRAC/LigandDisplayForward?ligandId=2826 (independent of tyrosine kinase inhibition) [http://www.ncbi.nlm.nih.gov/pubmed/20233211?dopt=AbstractPlus]	http://www.guidetopharmacology.org/GRAC/LigandDisplayForward?ligandId=10278 (Agonist) (pIC_50_ 6.4) [1099], http://www.guidetopharmacology.org/GRAC/LigandDisplayForward?ligandId=10281 (pEC_50_ 5.9) [http://www.ncbi.nlm.nih.gov/pubmed/28395140?dopt=AbstractPlus], http://www.guidetopharmacology.org/GRAC/LigandDisplayForward?ligandId=4191, http://www.guidetopharmacology.org/GRAC/LigandDisplayForward?ligandId=4101 [http://www.ncbi.nlm.nih.gov/pubmed/20008516?dopt=AbstractPlus], http://www.guidetopharmacology.org/GRAC/LigandDisplayForward?ligandId=2764 [http://www.ncbi.nlm.nih.gov/pubmed/17666455?dopt=AbstractPlus]	http://www.guidetopharmacology.org/GRAC/LigandDisplayForward?ligandId=10281 (pIC_50_ 6.1) [http://www.ncbi.nlm.nih.gov/pubmed/28395140?dopt=AbstractPlus]
Channel blockers	http://www.guidetopharmacology.org/GRAC/LigandDisplayForward?ligandId=10291 (Inhibition) (pIC_50_ 8.9) [http://www.ncbi.nlm.nih.gov/pubmed/28325835?dopt=AbstractPlus], http://www.guidetopharmacology.org/GRAC/LigandDisplayForward?ligandId=10286 (Antagonist) (pIC_50_ 8) [http://www.ncbi.nlm.nih.gov/pubmed/29385160?dopt=AbstractPlus], http://www.guidetopharmacology.org/GRAC/LigandDisplayForward?ligandId=10292 (Inhibition) (pIC_50_ 6.6) [http://www.ncbi.nlm.nih.gov/pubmed/26565375?dopt=AbstractPlus], http://www.guidetopharmacology.org/GRAC/LigandDisplayForward?ligandId=410 (p*K*_i_ 6.3) [http://www.ncbi.nlm.nih.gov/pubmed/26565375?dopt=AbstractPlus], http://www.guidetopharmacology.org/GRAC/LigandDisplayForward?ligandId=10285 (pIC_50_ 6) [http://www.ncbi.nlm.nih.gov/pubmed/25140002?dopt=AbstractPlus], http://www.guidetopharmacology.org/GRAC/LigandDisplayForward?ligandId=4232 (Inhibition) (pIC_50_ 5.9) [http://www.ncbi.nlm.nih.gov/pubmed/17658472?dopt=AbstractPlus], http://www.guidetopharmacology.org/GRAC/LigandDisplayForward?ligandId=10288 (Inhibition) (pIC_50_ 5.1) [http://www.ncbi.nlm.nih.gov/pubmed/25816897?dopt=AbstractPlus], http://www.guidetopharmacology.org/GRAC/LigandDisplayForward?ligandId=4255 (pIC_50_ ∼5) [http://www.ncbi.nlm.nih.gov/pubmed/21795696?dopt=AbstractPlus], http://www.guidetopharmacology.org/GRAC/LigandDisplayForward?ligandId=2433 (Antagonist) (pIC_50_ 4.7) [‐80mV] [http://www.ncbi.nlm.nih.gov/pubmed/15806115?dopt=AbstractPlus], http://www.guidetopharmacology.org/GRAC/LigandDisplayForward?ligandId=2434 (Antagonist) Concentration range: 5×10^−3^M [‐60mV] [http://www.ncbi.nlm.nih.gov/pubmed/12456670?dopt=AbstractPlus] – Mouse	http://www.guidetopharmacology.org/GRAC/LigandDisplayForward?ligandId=10284 (pIC_50_ 8.5) [80mV] [http://www.ncbi.nlm.nih.gov/pubmed/24453217?dopt=AbstractPlus], http://www.guidetopharmacology.org/GRAC/LigandDisplayForward?ligandId=10283 (pIC_50_ 8) [http://www.ncbi.nlm.nih.gov/pubmed/25847402?dopt=AbstractPlus], http://www.guidetopharmacology.org/GRAC/LigandDisplayForward?ligandId=10295 (Inhibition) (pIC_50_ 7) [http://www.ncbi.nlm.nih.gov/pubmed/26500253?dopt=AbstractPlus], http://www.guidetopharmacology.org/GRAC/LigandDisplayForward?ligandId=10279 (Antagonist) (pIC_50_ 6.4) [http://www.ncbi.nlm.nih.gov/pubmed/23886683?dopt=AbstractPlus], http://www.guidetopharmacology.org/GRAC/LigandDisplayForward?ligandId=10285 (pIC_50_ 5.9) [http://www.ncbi.nlm.nih.gov/pubmed/25140002?dopt=AbstractPlus], http://www.guidetopharmacology.org/GRAC/LigandDisplayForward?ligandId=2426 (Antagonist) (pIC_50_ 5.7) [‐60mV] [http://www.ncbi.nlm.nih.gov/pubmed/11179201?dopt=AbstractPlus] – Mouse, http://www.guidetopharmacology.org/GRAC/LigandDisplayForward?ligandId=2441 (Antagonist) (pIC_50_ 5.4) [‐60mV] [http://www.ncbi.nlm.nih.gov/pubmed/11179201?dopt=AbstractPlus] – Mouse, http://www.guidetopharmacology.org/GRAC/LigandDisplayForward?ligandId=7091 (pIC_50_ 5.3) [http://www.ncbi.nlm.nih.gov/pubmed/22530015?dopt=AbstractPlus], http://www.guidetopharmacology.org/GRAC/LigandDisplayForward?ligandId=2434 (pIC_50_ ∼5.2), http://www.guidetopharmacology.org/GRAC/LigandDisplayForward?ligandId=2421 (Antagonist) (pIC_50_ 3.9) [‐60mV] [http://www.ncbi.nlm.nih.gov/pubmed/11179201?dopt=AbstractPlus] – Mouse, http://www.guidetopharmacology.org/GRAC/LigandDisplayForward?ligandId=2440 (Antagonist) (pIC_50_ 3.6) [‐60mV] [http://www.ncbi.nlm.nih.gov/pubmed/11179201?dopt=AbstractPlus] – Mouse, http://www.guidetopharmacology.org/GRAC/LigandDisplayForward?ligandId=2433, http://www.guidetopharmacology.org/GRAC/LigandDisplayForward?ligandId=2443, http://www.guidetopharmacology.org/GRAC/LigandDisplayForward?ligandId=4206, Extracellular http://www.guidetopharmacology.org/GRAC/LigandDisplayForward?ligandId=2346, http://www.guidetopharmacology.org/GRAC/LigandDisplayForward?ligandId=4232, http://www.guidetopharmacology.org/GRAC/LigandDisplayForward?ligandId=4256	http://www.guidetopharmacology.org/GRAC/LigandDisplayForward?ligandId=10283 (pIC_50_ 6.7) [http://www.ncbi.nlm.nih.gov/pubmed/25847402?dopt=AbstractPlus], http://www.guidetopharmacology.org/GRAC/LigandDisplayForward?ligandId=2433, http://www.guidetopharmacology.org/GRAC/LigandDisplayForward?ligandId=2434 (Antagonist) Concentration range: 1×10^−4^M [‐60mV] [http://www.ncbi.nlm.nih.gov/pubmed/10488066?dopt=AbstractPlus] – Mouse, http://www.guidetopharmacology.org/GRAC/LigandDisplayForward?ligandId=2441 (Antagonist) Concentration range: 2.5×10^−5^M [‐60mV] [http://www.ncbi.nlm.nih.gov/pubmed/10488066?dopt=AbstractPlus] – Mouse, http://www.guidetopharmacology.org/GRAC/LigandDisplayForward?ligandId=2421
Selective channel blockers	http://www.guidetopharmacology.org/GRAC/LigandDisplayForward?ligandId=10290 (Inhibition) (pIC_50_ 4.8) [http://www.ncbi.nlm.nih.gov/pubmed/29217578?dopt=AbstractPlus]	–	–
Functional Characteristics	γ = 41‐63 pS; conducts mono‐and di‐valent cations non‐selectively (P_Ca_/P_Na_ = 1.8 – 9.5); dual rectification (inward and outward) as a homomer, outwardly rectifying when expressed with TRPC1 or TRPC4	γ = 28‐37 pS; conducts mono and divalent cations with a preference for divalents (P_Ca_/P_Na_ = 4.5–5.0); monovalent cation current suppressed by extracellular Ca^2+^ and Mg^2+^, dual rectification (inward and outward), or inward rectification	γ = 25–75 pS; conducts mono and divalent cations with a preference for divalents (P_Ca_/ P_Cs_ = 5.9); modest outward rectification (monovalent cation current recorded in the absence of extracellular divalents); monovalent cation current suppressed by extracellular Ca^2+^ and Mg^2+^

### TRPM (melastatin) family

Members of the TRPM subfamily (reviewed by [http://www.ncbi.nlm.nih.gov/pubmed/15530641?dopt=AbstractPlus, http://www.ncbi.nlm.nih.gov/pubmed/15843919?dopt=AbstractPlus, http://www.ncbi.nlm.nih.gov/pubmed/16098585?dopt=AbstractPlus, http://www.ncbi.nlm.nih.gov/pubmed/20233227?dopt=AbstractPlus]) fall into the five subgroups outlined below.

### TRPM1/M3 subgroup

In darkness, glutamate released by the photoreceptors and ON‐bipolar cells binds to the metabotropic glutamate receptor 6, leading to activation of Go . This results in the closure of TRPM1. When the photoreceptors are stimulated by light, glutamate release is reduced, and TRPM1 channels are more active, resulting in cell membrane depolarization. Human TRPM1 mutations are associated with congenital stationary night blindness (CSNB), whose patients lack rod function. TRPM1 is also found melanocytes. Isoforms of TRPM1 may present in melanocytes, melanoma, brain, and retina. In melanoma cells, TRPM1 is prevalent in highly dynamic intracellular vesicular structures [http://www.ncbi.nlm.nih.gov/pubmed/24756714?dopt=AbstractPlus, http://www.ncbi.nlm.nih.gov/pubmed/19436059?dopt=AbstractPlus]. TRPM3 (reviewed by [http://www.ncbi.nlm.nih.gov/pubmed/17217062?dopt=AbstractPlus]) exists as multiple splice variants which differ significantly in their biophysical properties. TRPM3 is expressed in somatosensory neurons and may be important in development of heat hyperalgesia during inflammation (see review [http://www.ncbi.nlm.nih.gov/pubmed/28720517?dopt=AbstractPlus]). TRPM3 is frequently coexpressed with TRPA1 and TRPV1 in these neurons. TRPM3 is expressed in pancreatic beta cells as well as brain, pituitary gland, eye, kidney, and adipose tissue [http://www.ncbi.nlm.nih.gov/pubmed/24756716?dopt=AbstractPlus, http://www.ncbi.nlm.nih.gov/pubmed/23511953?dopt=AbstractPlus]. TRPM3 may contribute to the detection of noxious heat [http://www.ncbi.nlm.nih.gov/pubmed/21555074?dopt=AbstractPlus].

### TRPM2

TRPM2 is activated under conditions of oxidative stress (respiratory burst of phagocytic cells) and ischemic conditions. However, the direct activators are ADPR(P) and calcium. As for many ion channels, PIP_2_ must also be present (reviewed by [http://www.ncbi.nlm.nih.gov/pubmed/20553742?dopt=AbstractPlus]). Numerous splice variants of TRPM2 exist which differ in their activation mechanisms [http://www.ncbi.nlm.nih.gov/pubmed/19372375?dopt=AbstractPlus]. The C‐terminal domain contains a TRP motif, a coiled‐coil region, and an enzymatic NUDT9 homologous domain. TRPM2 appears not to be activated by NAD, NAAD, or NAADP, but is directly activated by ADPRP (adenosine5′‐O‐disphosphoribose phosphate) [http://www.ncbi.nlm.nih.gov/pubmed/25918360?dopt=AbstractPlus]. TRPM2 is involved in warmth sensation [http://www.ncbi.nlm.nih.gov/pubmed/2955270?dopt=AbstractPlus], and contributes to neurological diseases [http://www.ncbi.nlm.nih.gov/pubmed/29671419?dopt=AbstractPlus]. Recent study shows that 2′‐deoxy‐ADPR is an endogenous TRPM2 superagonist [http://www.ncbi.nlm.nih.gov/pubmed/28671679?dopt=AbstractPlus].

### TRPM4/5 subgroup

TRPM4 and TRPM5 have the distinction within all TRP channels of being impermeable to Ca^2+^ [http://www.ncbi.nlm.nih.gov/pubmed/20716668?dopt=AbstractPlus]. A splice variant of TRPM4 (*i.e.*TRPM4b) and TRPM5 are molecular candidates for endogenous calcium‐activated cation (CAN) channels [http://www.ncbi.nlm.nih.gov/pubmed/21290294?dopt=AbstractPlus]. TRPM4 is active in the late phase of repolarization of the cardiac ventricular action potential. TRPM4 deletion or knockout enhances beta adrenergic‐mediated inotropy [http://www.ncbi.nlm.nih.gov/pubmed/24226423?dopt=AbstractPlus]. Mutations are associated with conduction defects [http://www.ncbi.nlm.nih.gov/pubmed/25600961?dopt=AbstractPlus, http://www.ncbi.nlm.nih.gov/pubmed/24226423?dopt=AbstractPlus, http://www.ncbi.nlm.nih.gov/pubmed/21887725?dopt=AbstractPlus]. TRPM4 has been shown to be an important regulator of Ca^2+^ entry in to mast cells [http://www.ncbi.nlm.nih.gov/pubmed/17217063?dopt=AbstractPlus] and dendritic cell migration [http://www.ncbi.nlm.nih.gov/pubmed/18758465?dopt=AbstractPlus]. TRPM5 in taste receptor cells of the tongue appears essential for the transduction of sweet, amino acid and bitter stimuli [http://www.ncbi.nlm.nih.gov/pubmed/17217064?dopt=AbstractPlus] TRPM5 contributes to the slow afterdepolarization of layer 5 neurons in mouse prefrontal cortex [http://www.ncbi.nlm.nih.gov/pubmed/25237295?dopt=AbstractPlus]. Both TRPM4 and TRPM5 are required transduction of taste stimuli [http://www.ncbi.nlm.nih.gov/pubmed/29311301?dopt=AbstractPlus].

### TRPM6/7 subgroup

TRPM6 and 7 combine channel and enzymatic activities (’chanzymes’). These channels have the unusual property of permeation by divalent (Ca^2+^, Mg^2+^, Zn^2+^) and monovalent cations, high single channel conductances, but overall extremely small inward conductance when expressed to the plasma membrane. They are inhibited by internal Mg^2+^ at 0.6 mM, around the free level of Mg^2+^ in cells. Whether they contribute to Mg^2+^ homeostasis is a contentious issue. When either gene is deleted in mice, the result is embryonic lethality. The C‐terminal kinase region is cleaved under unknown stimuli, and the kinase phosphorylates nuclear histones. TRPM7 is responsible for oxidant‐ induced Zn^2+^ release from intracellular vesicles [http://www.ncbi.nlm.nih.gov/pubmed/28696294?dopt=AbstractPlus] and contributes to intestinal mineral absorption essential for postnatal survival [http://www.ncbi.nlm.nih.gov/pubmed/30770447?dopt=AbstractPlus].

### TRPM8

Is a channel activated by cooling and pharmacological agents evoking a ’cool’ sensation and participates in the thermosensation of cold temperatures [http://www.ncbi.nlm.nih.gov/pubmed/17538622?dopt=AbstractPlus, http://www.ncbi.nlm.nih.gov/pubmed/17481392?dopt=AbstractPlus, http://www.ncbi.nlm.nih.gov/pubmed/17481391?dopt=AbstractPlus] reviewed by [http://www.ncbi.nlm.nih.gov/pubmed/20932257?dopt=AbstractPlus, http://www.ncbi.nlm.nih.gov/pubmed/21290296?dopt=AbstractPlus, http://www.ncbi.nlm.nih.gov/pubmed/20932258?dopt=AbstractPlus, http://www.ncbi.nlm.nih.gov/pubmed/17217067?dopt=AbstractPlus].

**Table:** 

Nomenclature	http://www.guidetopharmacology.org/GRAC/ObjectDisplayForward?objectId=493	http://www.guidetopharmacology.org/GRAC/ObjectDisplayForward?objectId=494
HGNC, UniProt	https://www.genenames.org/data/gene‐symbol‐report/#!/hgnc_id/HGNC:7146, http://www.uniprot.org/uniprot/Q7Z4N2	https://www.genenames.org/data/gene‐symbol‐report/#!/hgnc_id/HGNC:12339, http://www.uniprot.org/uniprot/O94759
Chemical activators	–	Agents producing reactive oxygen (e.g. H_2_O_2_) and nitrogen (e.g. GEA 3162) species
Physical activators	–	Heat 35°C
Endogenous activators	http://www.guidetopharmacology.org/GRAC/LigandDisplayForward?ligandId=4290 [http://www.ncbi.nlm.nih.gov/pubmed/21278253?dopt=AbstractPlus]	intracellular http://www.guidetopharmacology.org/GRAC/LigandDisplayForward?ligandId=2445 (Agonist) (pEC_50_ 5) [‐80mV – ‐60mV] [http://www.ncbi.nlm.nih.gov/pubmed/16585058?dopt=AbstractPlus, http://www.ncbi.nlm.nih.gov/pubmed/15808509?dopt=AbstractPlus, http://www.ncbi.nlm.nih.gov/pubmed/16601673?dopt=AbstractPlus], intracellular http://www.guidetopharmacology.org/GRAC/LigandDisplayForward?ligandId=2444 (Agonist) (pEC_50_ 3.9–4.4) [‐80mV] [http://www.ncbi.nlm.nih.gov/pubmed/11385575?dopt=AbstractPlus], intracellular http://www.guidetopharmacology.org/GRAC/LigandDisplayForward?ligandId=707 (perhaps *via* calmodulin), http://www.guidetopharmacology.org/GRAC/LigandDisplayForward?ligandId=2448 (Agonist) Concentration range: 5×10^−7^M‐5×10^−5^M [physiological voltage] [http://www.ncbi.nlm.nih.gov/pubmed/15302683?dopt=AbstractPlus, http://www.ncbi.nlm.nih.gov/pubmed/11804595?dopt=AbstractPlus, http://www.ncbi.nlm.nih.gov/pubmed/14512294?dopt=AbstractPlus, http://www.ncbi.nlm.nih.gov/pubmed/12562896?dopt=AbstractPlus, http://www.ncbi.nlm.nih.gov/pubmed/11960981?dopt=AbstractPlus], membrane http://www.guidetopharmacology.org/GRAC/LigandDisplayForward?ligandId=2387 [http://www.ncbi.nlm.nih.gov/pubmed/22847436?dopt=AbstractPlus], http://www.guidetopharmacology.org/GRAC/LigandDisplayForward?ligandId=2391 (Potentiation) Concentration range: 1×10^−5^M‐3×10^−5^M [physiological voltage] [http://www.ncbi.nlm.nih.gov/pubmed/11804595?dopt=AbstractPlus]
Activators	–	http://www.guidetopharmacology.org/GRAC/LigandDisplayForward?ligandId=4200
Endogenous channel blockers	http://www.guidetopharmacology.org/GRAC/LigandDisplayForward?ligandId=566 (pIC_50_ 6)	http://www.guidetopharmacology.org/GRAC/LigandDisplayForward?ligandId=566 (pIC_50_ 6), extracellular http://www.guidetopharmacology.org/GRAC/LigandDisplayForward?ligandId=2346
Channel blockers	–	http://www.guidetopharmacology.org/GRAC/LigandDisplayForward?ligandId=2433 (Antagonist) (pIC_50_ 6.1) [‐60mV] [http://www.ncbi.nlm.nih.gov/pubmed/18204483?dopt=AbstractPlus], http://www.guidetopharmacology.org/GRAC/LigandDisplayForward?ligandId=2443 (Antagonist) (pIC_50_ 5.8) [physiological voltage] [http://www.ncbi.nlm.nih.gov/pubmed/16604090?dopt=AbstractPlus], http://www.guidetopharmacology.org/GRAC/LigandDisplayForward?ligandId=2330 (Antagonist) Concentration range: 3×10^−6^M‐3×10^−5^M [‐60mV – ‐15mV] [http://www.ncbi.nlm.nih.gov/pubmed/15549272?dopt=AbstractPlus], http://www.guidetopharmacology.org/GRAC/LigandDisplayForward?ligandId=2446 (Antagonist) Concentration range: 3×10^−6^M‐3×10^−5^M [‐60mV – ‐15mV] [http://www.ncbi.nlm.nih.gov/pubmed/15549272?dopt=AbstractPlus], http://www.guidetopharmacology.org/GRAC/LigandDisplayForward?ligandId=2447 (Antagonist) Concentration range: 5×10^−5^M‐1×10^−3^M [‐60mV – ‐50mV] [http://www.ncbi.nlm.nih.gov/pubmed/15275834?dopt=AbstractPlus, http://www.ncbi.nlm.nih.gov/pubmed/18204483?dopt=AbstractPlus], http://www.guidetopharmacology.org/GRAC/LigandDisplayForward?ligandId=2449 (Antagonist) Concentration range: 1×10^−5^M [‐60mV] [http://www.ncbi.nlm.nih.gov/pubmed/18204483?dopt=AbstractPlus]
Functional Characteristics	Conducts mono‐ and di‐valent cations non‐selectively, dual rectification (inward and outward)	γ = 52‐60 pS at negative potentials, 76 pS at positive potentials; conducts mono‐ and di‐valent cations non‐selectively (P_Ca_/P_Na_ = 0.6‐0.7); non‐rectifying; inactivation at negative potentials; activated by oxidative stress probably *via* PARP‐1, PARP inhibitors reduce activation by oxidative stress, activation inhibited by suppression of APDR formation by glycohydrolase inhibitors.
Comments	–	Additional endogenous activators include 2′‐deoxy‐ADPR, 3′‐deoxy‐ADPR, 2′‐phospho‐ADPR, 2‐F‐ADPR and ADP‐ribose‐2′‐phosphate (ADPRP) [http://www.ncbi.nlm.nih.gov/pubmed/28671679?dopt=AbstractPlus, http://www.ncbi.nlm.nih.gov/pubmed/25918360?dopt=AbstractPlus]. 8‐Br‐cADPR acts as a gating inhibitor [http://www.ncbi.nlm.nih.gov/pubmed/15808509?dopt=AbstractPlus].

**Table:** 

Nomenclature	http://www.guidetopharmacology.org/GRAC/ObjectDisplayForward?objectId=495	http://www.guidetopharmacology.org/GRAC/ObjectDisplayForward?objectId=496
HGNC, UniProt	https://www.genenames.org/data/gene‐symbol‐report/#!/hgnc_id/HGNC:17992, http://www.uniprot.org/uniprot/Q9HCF6	https://www.genenames.org/data/gene‐symbol‐report/#!/hgnc_id/HGNC:17993, http://www.uniprot.org/uniprot/Q8TD43
Other channel blockers	–	Intracellular nucleotides including http://www.guidetopharmacology.org/GRAC/LigandDisplayForward?ligandId=1713, http://www.guidetopharmacology.org/GRAC/LigandDisplayForward?ligandId=1712, http://www.guidetopharmacology.org/GRAC/LigandDisplayForward?ligandId=2455 and http://www.guidetopharmacology.org/GRAC/LigandDisplayForward?ligandId=2456 with an IC_50_ range of 1.3‐1.9 *μ*M
Physical activators	heat (Q_10_ = 7.2 between 15 ‐ 25°C), hypotonic cell swelling [http://www.ncbi.nlm.nih.gov/pubmed/12672799?dopt=AbstractPlus, http://www.ncbi.nlm.nih.gov/pubmed/21555074?dopt=AbstractPlus, http://www.ncbi.nlm.nih.gov/pubmed/29305649?dopt=AbstractPlus]	Membrane depolarization (V_½_ = ‐20 mV to + 60 mV dependent upon conditions) in the presence of elevated [Ca^2+^]_i_, heat (Q_10_ = 8.5 @ +25 mV between 15 and 25°C)
Endogenous activators	http://www.guidetopharmacology.org/GRAC/LigandDisplayForward?ligandId=4290 (pEC_50_ 4.9) [http://www.ncbi.nlm.nih.gov/pubmed/18978782?dopt=AbstractPlus], http://www.guidetopharmacology.org/GRAC/LigandDisplayForward?ligandId=2452 (Agonist) (pEC_50_ 4.9) [physiological voltage] [http://www.ncbi.nlm.nih.gov/pubmed/15550678?dopt=AbstractPlus], http://www.guidetopharmacology.org/GRAC/LigandDisplayForward?ligandId=2453 (Agonist) (pEC_50_ 4.7) [http://www.ncbi.nlm.nih.gov/pubmed/15550678?dopt=AbstractPlus], http://www.guidetopharmacology.org/GRAC/LigandDisplayForward?ligandId=4188 [http://www.ncbi.nlm.nih.gov/pubmed/20735426?dopt=AbstractPlus]	intracellular http://www.guidetopharmacology.org/GRAC/LigandDisplayForward?ligandId=707 (Agonist) (pEC_50_ 3.9–6.3) [‐100mV – 100mV] [http://www.ncbi.nlm.nih.gov/pubmed/16424899?dopt=AbstractPlus, http://www.ncbi.nlm.nih.gov/pubmed/15331675?dopt=AbstractPlus, http://www.ncbi.nlm.nih.gov/pubmed/15590641?dopt=AbstractPlus, http://www.ncbi.nlm.nih.gov/pubmed/16407466?dopt=AbstractPlus]
Activators	http://www.guidetopharmacology.org/GRAC/LigandDisplayForward?ligandId=10297 (pEC_50_ 6.1) [http://www.ncbi.nlm.nih.gov/pubmed/25733887?dopt=AbstractPlus, http://www.ncbi.nlm.nih.gov/pubmed/28720517?dopt=AbstractPlus], http://www.guidetopharmacology.org/GRAC/LigandDisplayForward?ligandId=2514	http://www.guidetopharmacology.org/GRAC/LigandDisplayForward?ligandId=2438 (Agonist) (pEC_50_ 8.1) [‐80mV] [http://www.ncbi.nlm.nih.gov/pubmed/16407466?dopt=AbstractPlus], http://www.guidetopharmacology.org/GRAC/LigandDisplayForward?ligandId=2459 (Agonist) (pEC_50_ 5.7) [‐100mV] [http://www.ncbi.nlm.nih.gov/pubmed/15331675?dopt=AbstractPlus]
Gating inhibitors	http://www.guidetopharmacology.org/GRAC/LigandDisplayForward?ligandId=2433 (Antagonist) (pIC_50_ 4) [http://www.ncbi.nlm.nih.gov/pubmed/15806115?dopt=AbstractPlus]	http://www.guidetopharmacology.org/GRAC/LigandDisplayForward?ligandId=2447 (Antagonist) (pIC_50_ 5.6) [100mV] [http://www.ncbi.nlm.nih.gov/pubmed/15670874?dopt=AbstractPlus] – Mouse, http://www.guidetopharmacology.org/GRAC/LigandDisplayForward?ligandId=2330 (Antagonist) Concentration range: 1×10^−6^M‐1×10^−5^M [100mV] [720]
Endogenous channel blockers	http://www.guidetopharmacology.org/GRAC/LigandDisplayForward?ligandId=708 (Antagonist) (pIC_50_ 2) [http://www.ncbi.nlm.nih.gov/pubmed/15824111?dopt=AbstractPlus] – Mouse, extracellular http://www.guidetopharmacology.org/GRAC/LigandDisplayForward?ligandId=2340 (TRPM3α2 only)	–
Channel blockers	http://www.guidetopharmacology.org/GRAC/LigandDisplayForward?ligandId=10299 (pIC_50_ 6.3) [http://www.ncbi.nlm.nih.gov/pubmed/24006495?dopt=AbstractPlus], http://www.guidetopharmacology.org/GRAC/LigandDisplayForward?ligandId=5338 (pIC_50_ 6.2) [http://www.ncbi.nlm.nih.gov/pubmed/28106668?dopt=AbstractPlus], http://www.guidetopharmacology.org/GRAC/LigandDisplayForward?ligandId=2402 (pIC_50_ 5.8) [http://www.ncbi.nlm.nih.gov/pubmed/28106668?dopt=AbstractPlus], http://www.guidetopharmacology.org/GRAC/LigandDisplayForward?ligandId=2714 (pIC_50_ 5.2) [http://www.ncbi.nlm.nih.gov/pubmed/27433342?dopt=AbstractPlus], http://www.guidetopharmacology.org/GRAC/LigandDisplayForward?ligandId=8899 (pIC_50_ 5.2) [http://www.ncbi.nlm.nih.gov/pubmed/24006495?dopt=AbstractPlus], http://www.guidetopharmacology.org/GRAC/LigandDisplayForward?ligandId=10298 (pIC_50_ 5.2) [http://www.ncbi.nlm.nih.gov/pubmed/24006495?dopt=AbstractPlus, http://www.ncbi.nlm.nih.gov/pubmed/23190005?dopt=AbstractPlus], http://www.guidetopharmacology.org/GRAC/LigandDisplayForward?ligandId=2426 (Antagonist) (pIC_50_ 4) [http://www.ncbi.nlm.nih.gov/pubmed/12672799?dopt=AbstractPlus, http://www.ncbi.nlm.nih.gov/pubmed/12672827?dopt=AbstractPlus], http://www.guidetopharmacology.org/GRAC/LigandDisplayForward?ligandId=2434 (Antagonist) (pIC_50_ 4) [http://www.ncbi.nlm.nih.gov/pubmed/12672799?dopt=AbstractPlus,http://www.ncbi.nlm.nih.gov/pubmed/12672827?dopt=AbstractPlus]	http://www.guidetopharmacology.org/GRAC/LigandDisplayForward?ligandId=4114 (pIC_50_ 4.6–4.8) [http://www.ncbi.nlm.nih.gov/pubmed/18297105?dopt=AbstractPlus], http://www.guidetopharmacology.org/GRAC/LigandDisplayForward?ligandId=710 (Antagonist) (pIC_50_ 4.2) [100mV] [http://www.ncbi.nlm.nih.gov/pubmed/14758478?dopt=AbstractPlus], http://www.guidetopharmacology.org/GRAC/LigandDisplayForward?ligandId=2844 (pIC_50_ 3.2)
Functional Characteristics	TRPM3_1235_: γ = 83 pS (Na^+^ current), 65 pS (Ca^2+^ current); conducts mono and di‐valent cations non‐selectively (P_Ca_/P_Na_ = 1.6) TRPM3α1: selective for monovalent cations (P_Ca_/P_Cs_0.1); TRPM3α2: conducts mono‐ and di‐valent cations non‐selectively (P_Ca_/P_Cs_ = 1‐10); In‐ and outwardly rectifying currents by co‐application of pregnenolone sulphate and clotrimazole or single application of CIM0216 [http://www.ncbi.nlm.nih.gov/pubmed/25733887?dopt=AbstractPlus, http://www.ncbi.nlm.nih.gov/pubmed/24390427?dopt=AbstractPlus].	γ = 23 pS (within the range 60 to +60 mV); permeable to monovalent cations; impermeable to Ca^2+^; strong outward rectification; slow activation at positive potentials, rapid deactivation at negative potentials, deactivation blocked by decavanadate
Comments	G protein βγ subunits can act as endogenous inhibitors of TRPM3 channel activity [http://www.ncbi.nlm.nih.gov/pubmed/28829742?dopt=AbstractPlus, http://www.ncbi.nlm.nih.gov/pubmed/28826482?dopt=AbstractPlus, http://www.ncbi.nlm.nih.gov/pubmed/28826490?dopt=AbstractPlus].	–

**Table:** 

Nomenclature	http://www.guidetopharmacology.org/GRAC/ObjectDisplayForward?objectId=497	http://www.guidetopharmacology.org/GRAC/ObjectDisplayForward?objectId=498
HGNC, UniProt	https://www.genenames.org/data/gene‐symbol‐report/#!/hgnc_id/HGNC:14323, http://www.uniprot.org/uniprot/Q9NZQ8	https://www.genenames.org/data/gene‐symbol‐report/#!/hgnc_id/HGNC:17995, http://www.uniprot.org/uniprot/Q9BX84
EC number	–	http://www.genome.jp/dbget‐bin/www_bget?ec:2.7.11.1
Other chemical activators	–	constitutively active, activated by reduction of intracellular Mg^2+^
Physical activators	membrane depolarization (V_½_ = 0 to + 120 mV dependent upon conditions), heat (Q_10_ = 10.3 @ ‐75 mV between 15 and 25°C)	–
Endogenous activators	intracellular http://www.guidetopharmacology.org/GRAC/LigandDisplayForward?ligandId=707 (Agonist) (pEC_50_ 4.5–6.2) [‐80mV – 80mV] [http://www.ncbi.nlm.nih.gov/pubmed/12842017?dopt=AbstractPlus, http://www.ncbi.nlm.nih.gov/pubmed/14657398?dopt=AbstractPlus, http://www.ncbi.nlm.nih.gov/pubmed/15670874?dopt=AbstractPlus] – Mouse	extracellular http://www.guidetopharmacology.org/GRAC/LigandDisplayForward?ligandId=2346 (Potentiation), intracellular http://www.guidetopharmacology.org/GRAC/LigandDisplayForward?ligandId=708
Activators	–	http://www.guidetopharmacology.org/GRAC/LigandDisplayForward?ligandId=2433 (Agonist) (pEC_50_ 3.4–3.7) [‐120mV – 100mV] [http://www.ncbi.nlm.nih.gov/pubmed/16636202?dopt=AbstractPlus]
Endogenous channel blockers	–	http://www.guidetopharmacology.org/GRAC/LigandDisplayForward?ligandId=708 (inward current mediated by monovalent cations is blocked) (pIC_50_ 5.5–6), http://www.guidetopharmacology.org/GRAC/LigandDisplayForward?ligandId=707 (inward current mediated by monovalent cations is blocked) (pIC_50_ 5.3–5.3)
Channel blockers	http://www.guidetopharmacology.org/GRAC/LigandDisplayForward?ligandId=2447 (pIC_50_ 4.6), intracellular http://www.guidetopharmacology.org/GRAC/LigandDisplayForward?ligandId=710 (pIC_50_ 4.4), Extracellular http://www.guidetopharmacology.org/GRAC/LigandDisplayForward?ligandId=2346 (pIC_50_ 3.2)	http://www.guidetopharmacology.org/GRAC/LigandDisplayForward?ligandId=2432 (pIC_50_ 7) [*voltage dependent* ‐120mV]
Functional Characteristics	γ = 15‐25 pS; conducts monovalent cations selectively (P_Ca_/P_Na_ = 0.05); strong outward rectification; slow activation at positive potentials, rapid inactivation at negative potentials; activated and subsequently desensitized by [Ca^2+^]_I_	γ= 40–87 pS; permeable to mono‐ and di‐valent cations with a preference for divalents (Mg^2+^ > Ca^2+^; P_Ca_/P_Na_ = 6.9), conductance sequence Zn^2+^ > Ba^2+^ > Mg^2+^= Ca^2+^ = Mn^2+^ > Sr^2+^ > Cd^2+^> Ni^2+^; strong outward rectification abolished by removal of extracellular divalents, inhibited by intracellular Mg^2+^ (IC_50_ = 0.5 mM) and ATP
Comments	TRPM5 is not blocked by http://www.guidetopharmacology.org/GRAC/LigandDisplayForward?ligandId=1713	–

**Table:** 

Nomenclature	http://www.guidetopharmacology.org/GRAC/ObjectDisplayForward?objectId=499	http://www.guidetopharmacology.org/GRAC/ObjectDisplayForward?objectId=500
HGNC, UniProt	https://www.genenames.org/data/gene‐symbol‐report/#!/hgnc_id/HGNC:17994, http://www.uniprot.org/uniprot/Q96QT4	https://www.genenames.org/data/gene‐symbol‐report/#!/hgnc_id/HGNC:17961, http://www.uniprot.org/uniprot/Q7Z2W7
EC number	http://www.genome.jp/dbget‐bin/www_bget?ec:2.7.11.1	–
Chemical activators	–	agonist activities are temperature dependent and potentiated by cooling
Physical activators	–	depolarization (V_½_ +50 mV at 15°C), cooling (*<* 22‐26°C)
Endogenous activators	Extracellular http://www.guidetopharmacology.org/GRAC/LigandDisplayForward?ligandId=2346 (Potentiation)	–
Activators	http://www.guidetopharmacology.org/GRAC/LigandDisplayForward?ligandId=2433 Concentration range: >1×10^−3^M [http://www.ncbi.nlm.nih.gov/pubmed/11385574?dopt=AbstractPlus] – Mouse, http://www.guidetopharmacology.org/GRAC/LigandDisplayForward?ligandId=1640 [http://www.ncbi.nlm.nih.gov/pubmed/24633576?dopt=AbstractPlus]	http://www.guidetopharmacology.org/GRAC/LigandDisplayForward?ligandId=2429 (Agonist) (pEC_50_ 6.7–6.9) [physiological voltage] [http://www.ncbi.nlm.nih.gov/pubmed/15190109?dopt=AbstractPlus, http://www.ncbi.nlm.nih.gov/pubmed/14757700?dopt=AbstractPlus] – Mouse, (‐)http://www.guidetopharmacology.org/GRAC/LigandDisplayForward?ligandId=2430 (inhibited by intracellular Ca^2+^) (pEC_50_ 4.6) [‐120mV – 160mV] [http://www.ncbi.nlm.nih.gov/pubmed/15306801?dopt=AbstractPlus]
Selective activators	–	http://www.guidetopharmacology.org/GRAC/LigandDisplayForward?ligandId=4343 (Full agonist) (pEC_50_ 4.9) [physiological voltage] [http://www.ncbi.nlm.nih.gov/pubmed/18930858?dopt=AbstractPlus, http://www.ncbi.nlm.nih.gov/pubmed/20816009?dopt=AbstractPlus] – Rat
Channel blockers	http://www.guidetopharmacology.org/GRAC/LigandDisplayForward?ligandId=710 (Inhibition) (p*K*_i_ 5.6) [‐110mV – 80mV] [http://www.ncbi.nlm.nih.gov/pubmed/12149283?dopt=AbstractPlus] – Rat, http://www.guidetopharmacology.org/GRAC/LigandDisplayForward?ligandId=2433 (Inhibition) (pIC_50_ 3.8) [‐100mV – 100mV] [http://www.ncbi.nlm.nih.gov/pubmed/16636202?dopt=AbstractPlus] – Mouse, http://www.guidetopharmacology.org/GRAC/LigandDisplayForward?ligandId=2497 (Inhibition) (pIC_50_ 3.5) [‐100mV – 100mV] [http://www.ncbi.nlm.nih.gov/pubmed/19135721?dopt=AbstractPlus] – Mouse, http://www.guidetopharmacology.org/GRAC/LigandDisplayForward?ligandId=708 (Antagonist) (pIC_50_ 2.5) [80mV] [http://www.ncbi.nlm.nih.gov/pubmed/11385574?dopt=AbstractPlus] – Mouse, http://www.guidetopharmacology.org/GRAC/LigandDisplayForward?ligandId=2434 (Antagonist) Concentration range: 2×10^−3^M [‐100mV – 100mV] [http://www.ncbi.nlm.nih.gov/pubmed/11161216?dopt=AbstractPlus] – Mouse	http://www.guidetopharmacology.org/GRAC/LigandDisplayForward?ligandId=2460 (Antagonist) (pIC_50_ 6.1) [physiological voltage] [http://www.ncbi.nlm.nih.gov/pubmed/14757700?dopt=AbstractPlus] – Mouse, http://www.guidetopharmacology.org/GRAC/LigandDisplayForward?ligandId=2433 (Antagonist) (pIC_50_ 4.9–5.1) [100mV – ‐100mV] [http://www.ncbi.nlm.nih.gov/pubmed/15194687?dopt=AbstractPlus, http://www.ncbi.nlm.nih.gov/pubmed/21964764?dopt=AbstractPlus] – Mouse, http://www.guidetopharmacology.org/GRAC/LigandDisplayForward?ligandId=2461 (Antagonist) (pIC_50_ 4.7) [physiological voltage] [http://www.ncbi.nlm.nih.gov/pubmed/14757700?dopt=AbstractPlus] – Mouse
Selective channel blockers	–	http://www.guidetopharmacology.org/GRAC/LigandDisplayForward?ligandId=10300 (Antagonist) (pIC_50_ 7) [*voltage dependent*] [http://www.ncbi.nlm.nih.gov/pubmed/25893043?dopt=AbstractPlus]
Functional Characteristics	γ = 40‐105 pS at negative and positive potentials respectively; conducts mono‐and di‐valent cations with a preference for monovalents (P_Ca_/P_Na_ = 0.34); conductance sequence Ni^2+^ > Zn^2+^ > Ba^2+^ = Mg^2+^ > Ca^2+^ = Mn^2+^ > Sr^2+^ > Cd^2+^; outward rectification, decreased by removal of extracellular divalent cations; inhibited by intracellular Mg^2+^, Ba^2+^, Sr^2+^, Zn^2+^, Mn^2+^ and Mg.ATP (disputed); activated by and intracellular alkalinization; sensitive to osmotic gradients	γ = 40‐83 pS at positive potentials; conducts mono‐ and di‐valent cations non‐selectively (P_Ca_/P_Na_ = 1.0–3.3); pronounced outward rectification; demonstrates densensitization to chemical agonists and adaptation to a cold stimulus in the presence of Ca^2+^; modulated by lysophospholipids and PUFAs
Comments	http://www.guidetopharmacology.org/GRAC/LigandDisplayForward?ligandId=2433 acts as a channel blocker in the *μ*M range. Recent study shows cAMP inhibits TRPM7‐mediated Ca^2+^ influx [http://www.ncbi.nlm.nih.gov/pubmed/30615643?dopt=AbstractPlus]. Waixenicin‐A specifically inhibits TRPM7 [http://www.ncbi.nlm.nih.gov/pubmed/21926172?dopt=AbstractPlus].	http://www.guidetopharmacology.org/GRAC/LigandDisplayForward?ligandId=4150 and http://www.guidetopharmacology.org/GRAC/LigandDisplayForward?ligandId=2424 are examples of cannabinoid activators. TRPM8 is insensitive to http://www.guidetopharmacology.org/GRAC/LigandDisplayForward?ligandId=2432. http://www.guidetopharmacology.org/GRAC/LigandDisplayForward?ligandId=2429 requires intracellular Ca^2+^ for full agonist activity.

### Comments

Ca^2+^ activates all splice variants of TRPM2, but other activators listed are effective only at the full length isoform [http://www.ncbi.nlm.nih.gov/pubmed/19372375?dopt=AbstractPlus]. Inhibition of TRPM2 by http://www.guidetopharmacology.org/GRAC/LigandDisplayForward?ligandId=2330, http://www.guidetopharmacology.org/GRAC/LigandDisplayForward?ligandId=2449, http://www.guidetopharmacology.org/GRAC/LigandDisplayForward?ligandId=2446, http://www.guidetopharmacology.org/GRAC/LigandDisplayForward?ligandId=2447 is largely irreversible. Co‐application of pregnenolone sulphate and clotrimazole caused TRPM3 currents to acquire an inwardly rectifying component at negative voltages, resulting in a biphasic conductance‐voltage relationship. Evidence was presented that the inward current might reflect the permeation of cations through the opening of a non‐canonical pore [http://www.ncbi.nlm.nih.gov/pubmed/24390427?dopt=AbstractPlus]. TRPM3 activity is impaired in chronic fatigue syndrome/myalgic encephalomyelitis patients suggesting changes in intracellular Ca^2+^ concentration, which may impact natural killer cellular functions [http://www.ncbi.nlm.nih.gov/pubmed/30134818?dopt=AbstractPlus]. TRPM4 exists as multiple spice variants: data listed are for TRPM4b. The sensitivity of TRPM4b and TRPM5 to activation by [Ca^2+^]_i_ demonstrates a pronounced and time‐dependent reduction following excision of inside‐out membrane patches [http://www.ncbi.nlm.nih.gov/pubmed/15670874?dopt=AbstractPlus]. The V_½_ for activation of TRPM4 and TRPM5 demonstrates a pronounced negative shift with increasing temperature. Activation of TRPM8 by depolarization is strongly temperature‐dependent via a channel‐closing rate that decreases with decreasing temperature. The V_½_ is shifted in the hyperpolarizing direction both by decreasing temperature and by exogenous agonists, such as http://www.guidetopharmacology.org/GRAC/LigandDisplayForward?ligandId=2430 [http://www.ncbi.nlm.nih.gov/pubmed/15306801?dopt=AbstractPlus] whereas antagonists produce depolarizing shifts in V_½_ [http://www.ncbi.nlm.nih.gov/pubmed/17317754?dopt=AbstractPlus]. The V_½_ for the native channel is far more positive than that of heterologously expressed TRPM8 [http://www.ncbi.nlm.nih.gov/pubmed/17317754?dopt=AbstractPlus]. It should be noted that http://www.guidetopharmacology.org/GRAC/LigandDisplayForward?ligandId=2430 and structurally related compounds can elicit release of Ca^2+^ from the endoplasmic reticulum independent of activation of TRPM8 [http://www.ncbi.nlm.nih.gov/pubmed/17142461?dopt=AbstractPlus]. Intracellular pH modulates activation of TRPM8 by cold and http://www.guidetopharmacology.org/GRAC/LigandDisplayForward?ligandId=2429, but not http://www.guidetopharmacology.org/GRAC/LigandDisplayForward?ligandId=2430 [http://www.ncbi.nlm.nih.gov/pubmed/15190109?dopt=AbstractPlus].

### TRPML (mucolipin) family

The TRPML family [http://www.ncbi.nlm.nih.gov/pubmed/26336837?dopt=AbstractPlus, http://www.ncbi.nlm.nih.gov/pubmed/19158345?dopt=AbstractPlus, http://www.ncbi.nlm.nih.gov/pubmed/15971078?dopt=AbstractPlus, http://www.ncbi.nlm.nih.gov/pubmed/25668017?dopt=AbstractPlus, http://www.ncbi.nlm.nih.gov/pubmed/17306511?dopt=AbstractPlus] consists of three mammalian members (TRPML1‐3). TRPML channels are probably restricted to intracellular vesicles and mutations in the gene (*MCOLN1*) encoding TRPML1 (mucolipin‐1) cause the neurodegenerative disorder mucolipidosis type IV (MLIV) in man. TRPML1 is a cation selective ion channel that is important for sorting/transport of endosomes in the late endocytotic pathway and specifically, fission from late endosome‐lysosome hybrid vesicles and lysosomal exocytosis [http://www.ncbi.nlm.nih.gov/pubmed/23993788?dopt=AbstractPlus]. TRPML2 and TRPML3 show increased channel activity in low extracellular sodium and are activated by similar small molecules [http://www.ncbi.nlm.nih.gov/pubmed/22753890?dopt=AbstractPlus]. A naturally occurring gain of function mutation in TRPML3 (*i.e.* A419P) results in the varitint waddler (V*a*) mouse phenotype (reviewed by [http://www.ncbi.nlm.nih.gov/pubmed/17237345?dopt=AbstractPlus,http://www.ncbi.nlm.nih.gov/pubmed/15971078?dopt=AbstractPlus]).

**Table:** 

Nomenclature	http://www.guidetopharmacology.org/GRAC/ObjectDisplayForward?objectId=501	http://www.guidetopharmacology.org/GRAC/ObjectDisplayForward?objectId=502	http://www.guidetopharmacology.org/GRAC/ObjectDisplayForward?objectId=503
HGNC, UniProt	https://www.genenames.org/data/gene‐symbol‐report/#!/hgnc_id/HGNC:13356, http://www.uniprot.org/uniprot/Q9GZU1	https://www.genenames.org/data/gene‐symbol‐report/#!/hgnc_id/HGNC:13357, http://www.uniprot.org/uniprot/Q8IZK6	https://www.genenames.org/data/gene‐symbol‐report/#!/hgnc_id/HGNC:13358, http://www.uniprot.org/uniprot/Q8TDD5
Endogenous activators	http://www.guidetopharmacology.org/GRAC/LigandDisplayForward?ligandId=2794 (Also activates other TRPMLs) (pEC_50_ 7.3) [http://www.ncbi.nlm.nih.gov/pubmed/20802798?dopt=AbstractPlus]	–	–
Activators	http://www.guidetopharmacology.org/GRAC/LigandDisplayForward?ligandId=6386 (pEC_50_ 7.3) [‐140mV] [http://www.ncbi.nlm.nih.gov/pubmed/22415822?dopt=AbstractPlus], http://www.guidetopharmacology.org/GRAC/LigandDisplayForward?ligandId=9783 (pEC_50_ 7) [‐200mV] [http://www.ncbi.nlm.nih.gov/pubmed/25119295?dopt=AbstractPlus], http://www.guidetopharmacology.org/GRAC/LigandDisplayForward?ligandId=6389 (pEC_50_ 6.3) [‐200mV] [http://www.ncbi.nlm.nih.gov/pubmed/25119295?dopt=AbstractPlus], http://www.guidetopharmacology.org/GRAC/LigandDisplayForward?ligandId=6387 (pEC_50_ 4.5) [http://www.ncbi.nlm.nih.gov/pubmed/22415822?dopt=AbstractPlus] TRPML1^Va^: Constitutively active, current potentiated by extracellular acidification (equivalent to intralysosomal acidification)	http://www.guidetopharmacology.org/GRAC/LigandDisplayForward?ligandId=6386 Concentration range: 1×10^−5^M [‐140mV] [http://www.ncbi.nlm.nih.gov/pubmed/22415822?dopt=AbstractPlus], http://www.guidetopharmacology.org/GRAC/LigandDisplayForward?ligandId=2794 Concentration range: 1×10^−6^M [‐140mV] [http://www.ncbi.nlm.nih.gov/pubmed/20802798?dopt=AbstractPlus] TRPML2^Va^: Constitutively active, current potentiated by extracellular acidification (equivalent to intralysosomal acidification)	http://www.guidetopharmacology.org/GRAC/LigandDisplayForward?ligandId=6386 Concentration range: 1×10^−5^M [‐140mV] [http://www.ncbi.nlm.nih.gov/pubmed/22415822?dopt=AbstractPlus], http://www.guidetopharmacology.org/GRAC/LigandDisplayForward?ligandId=2794 Concentration range: 1×10^−6^M [‐140mV] [http://www.ncbi.nlm.nih.gov/pubmed/20802798?dopt=AbstractPlus]
Selective activators Channel blockers	–	http://www.guidetopharmacology.org/GRAC/LigandDisplayForward?ligandId=2426 (Antagonist) (pIC_50_ 4.7) [‐80mV] [http://www.ncbi.nlm.nih.gov/pubmed/18162548?dopt=AbstractPlus] – Mouse	
	–	http://www.guidetopharmacology.org/GRAC/LigandDisplayForward?ligandId=10301 (Agonist) (pEC_50_ 5.9) [‐100mV] [http://www.ncbi.nlm.nih.gov/pubmed/30479274?dopt=AbstractPlus]	
Functional Characteristics	TRPML1^Va^: γ = 40 pS and 76‐86 pS at very negative holding potentials with Fe^2+^ and monovalent cations as charge carriers, respectively; conducts Na^+^≅ K^+^>Cs^+^ and divalent cations (Ba^2+^>Mn2^+^>Fe^2+^>Ca^2+^> Mg^2+^ > Ni^2+^ > Co^2+^ > Cd^2+^ > Zn^2+^≫Cu^2+^); monovalent cation flux suppressed by divalent cations (*e.g.* Ca^2+^, Fe^2+^); inwardly rectifying	Conducts Na^+^; monovalent cation flux suppressed by divalent cations; inwardly rectifying	TRPML3^Va^: γ = 49 pS at very negative holding potentials with monovalent cations as charge carrier; conducts Na^+^ > K^+^ > Cs^+^ with maintained current in the presence of Na^+^, conducts Ca^2+^ and Mg^2+^, but not Fe^2+^, impermeable to protons; inwardly rectifying Wild type TRPML3: γ = 59 pS at negative holding potentials with monovalent cations as charge carrier; conducts Na^+^ > K^+^ > Cs^+^ and Ca^2+^ (P_Ca_/P_K_^∼^ = 350), slowly inactivates in the continued presence of Na^+^ within the extracellular (extracytosolic) solution; outwardly rectifying
Comments	TRPML1 current is potentiated by acidic pH and sphingosine [http://www.ncbi.nlm.nih.gov/pubmed/22415822?dopt=AbstractPlus].	–	Current is activated by Na^+^‐free extracellular (extracytosolic) solution, and is inhibited by extracellular acidification (equivalent to intra‐lysosomal acidification). Channel blockers include the ML‐SI series of compounds (*e.g*. ML‐SI1; concentration range 5x10^‐5^ M; ‐120mV) [http://www.ncbi.nlm.nih.gov/pubmed/26027738?dopt=AbstractPlus].

### Comments

Data in the table are for TRPML proteins mutated (*i.e* TRPML1^Va^, TRPML2^Va^ and TRPML3^Va^) at loci equivalent to TRPML3 A419P to allow plasma membrane expression when expressed in HEK‐293 cells and subsequent characterisation by patch‐clamp recording [http://www.ncbi.nlm.nih.gov/pubmed/18794901?dopt=AbstractPlus, http://www.ncbi.nlm.nih.gov/pubmed/18048323?dopt=AbstractPlus, http://www.ncbi.nlm.nih.gov/pubmed/17962195?dopt=AbstractPlus, http://www.ncbi.nlm.nih.gov/pubmed/18162548?dopt=AbstractPlus, http://www.ncbi.nlm.nih.gov/pubmed/17989217?dopt=AbstractPlus]. Data for wild type TRPML3 are also tabulated [http://www.ncbi.nlm.nih.gov/pubmed/17962195?dopt=AbstractPlus, http://www.ncbi.nlm.nih.gov/pubmed/18369318?dopt=AbstractPlus, http://www.ncbi.nlm.nih.gov/pubmed/18162548?dopt=AbstractPlus, http://www.ncbi.nlm.nih.gov/pubmed/17989217?dopt=AbstractPlus]. It should be noted that alternative methodologies, particularly in the case of TRPML1, have resulted in channels with differing biophysical characteristics (reviewed by [http://www.ncbi.nlm.nih.gov/pubmed/19158345?dopt=AbstractPlus]). Initial functional characteristics of TRPML channels are performed on their Va mutations of TRPMLs at loci equivalent to TRPML3 A419P. Current pharmacological characterization of channel activators and blockers are conducted on wild‐type channel proteins using endolysosomal patch‐clamp [http://www.ncbi.nlm.nih.gov/pubmed/25119295?dopt=AbstractPlus, http://www.ncbi.nlm.nih.gov/pubmed/20802798?dopt=AbstractPlus, http://www.ncbi.nlm.nih.gov/pubmed/30479274?dopt=AbstractPlus, http://www.ncbi.nlm.nih.gov/pubmed/22415822?dopt=AbstractPlus].

### TRPP (polycystin) family

The TRPP family (reviewed by [http://www.ncbi.nlm.nih.gov/pubmed/15889307?dopt=AbstractPlus, http://www.ncbi.nlm.nih.gov/pubmed/15336986?dopt=AbstractPlus, http://www.ncbi.nlm.nih.gov/pubmed/16880824?dopt=AbstractPlus, http://www.ncbi.nlm.nih.gov/pubmed/21290302?dopt=AbstractPlus, http://www.ncbi.nlm.nih.gov/pubmed/17217069?dopt=AbstractPlus]) or PKD2 family is comprised of PKD2 (PC2), PKD2L1 (PC2L1), PKD2L2 (PC2L2), which have been renamed TRPP1, TRPP2 and TRPP3, respectively [http://www.ncbi.nlm.nih.gov/pubmed/20716668?dopt=AbstractPlus]. It should also be noted that the nomenclature of PC2 was TRPP2 in old literature. However, PC2 has been uniformed to be called TRPP2 [http://www.ncbi.nlm.nih.gov/pubmed/29149325?dopt=AbstractPlus]. PKD2 family channels are clearly distinct from the PKD1 family, whose function is unknown. PKD1 and PKD2 form a hetero‐oligomeric complex with a 1:3 ratio. [http://www.ncbi.nlm.nih.gov/pubmed/30093605?dopt=AbstractPlus]. Although still being sorted out, TRPP family members appear to be 6TM spanning nonselective cation channels.

**Table:** 

Nomenclature	http://www.guidetopharmacology.org/GRAC/ObjectDisplayForward?objectId=504	http://www.guidetopharmacology.org/GRAC/ObjectDisplayForward?objectId=505	http://www.guidetopharmacology.org/GRAC/ObjectDisplayForward?objectId=506
HGNC, UniProt	https://www.genenames.org/data/gene‐symbol‐report/#!/hgnc_id/HGNC:9009, http://www.uniprot.org/uniprot/Q13563	https://www.genenames.org/data/gene‐symbol‐report/#!/hgnc_id/HGNC:9011, http://www.uniprot.org/uniprot/Q9P0L9	https://www.genenames.org/data/gene‐symbol‐report/#!/hgnc_id/HGNC:9012, http://www.uniprot.org/uniprot/Q9NZM6
Activators	–	Calmidazolium (in primary cilia): 10 *μ*M	–
Channel blockers	–	http://www.guidetopharmacology.org/GRAC/LigandDisplayForward?ligandId=4281 (pIC_50_ 6.9), http://www.guidetopharmacology.org/GRAC/LigandDisplayForward?ligandId=4145 (pIC_50_ 6), http://www.guidetopharmacology.org/GRAC/LigandDisplayForward?ligandId=4186 (pIC_50_ 5), http://www.guidetopharmacology.org/GRAC/LigandDisplayForward?ligandId=2421 (pIC_50_ 3.8), http://www.guidetopharmacology.org/GRAC/LigandDisplayForward?ligandId=2426 Concentration range: 1×10^−4^M [‐50mV] [http://www.ncbi.nlm.nih.gov/pubmed/10517637?dopt=AbstractPlus], http://www.guidetopharmacology.org/GRAC/LigandDisplayForward?ligandId=2434 Concentration range: 1×10^−4^M [‐50mV] [http://www.ncbi.nlm.nih.gov/pubmed/10517637?dopt=AbstractPlus], http://www.guidetopharmacology.org/GRAC/LigandDisplayForward?ligandId=4191	–
Functional Characteristics	TRPP1 (PKD2) is a cation channel found in primary cilia of inner medullary collecting duct (IMCD) cells, but not at the plasma membrane; conducts K^+^>Na^+^>Ca^2+^ (1:0.4:0.025). PKD2 shows a large outward conductance (90‐117 pS) [http://www.ncbi.nlm.nih.gov/pubmed/29443690?dopt=AbstractPlus, http://www.ncbi.nlm.nih.gov/pubmed/22760766?dopt=AbstractPlus]. Activators or channel blockers remain to be investigated.	Currents have been measured directly from primary cilia and also when expressed on plasma membranes. Primary cilia appear to contain heteromeric TRPP2 + PKD1‐L1, underlying a gently outwardly rectifying nonselective conductance (P_Ca_/P_Na_ 6: PKD1‐L1 is a 12 TM protein of unknown topology). Primary cilia heteromeric channels have an inward single channel conductance of 80 pS and an outward single channel conductance of 95 pS. Presumed homomeric TRPP2 channels are gently outwardly rectifying. Single channel conductance is 120 pS inward, 200 pS outward [http://www.ncbi.nlm.nih.gov/pubmed/24336289?dopt=AbstractPlus]. TRPP2 (PKD2L1) displays calcium dependent activation. Calcium accumulation due to prolonged channel activity may lead to outward‐moving Ca^2+^ ions to close the channel [http://www.ncbi.nlm.nih.gov/pubmed/27348301?dopt=AbstractPlus].	The functional characteristics of PKD2L2(PC2L2) has not been established yet.

### Comments

Data in the table are extracted from [http://www.ncbi.nlm.nih.gov/pubmed/17804601?dopt=AbstractPlus, http://www.ncbi.nlm.nih.gov/pubmed/15336986?dopt=AbstractPlus] and [http://www.ncbi.nlm.nih.gov/pubmed/18663466?dopt=AbstractPlus]. Broadly similar single channel conductance, mono‐ and di‐valent cation selectivity and sensitivity to blockers are observed for TRPP2 co‐expressed with TRPP1 [http://www.ncbi.nlm.nih.gov/pubmed/14766803?dopt=AbstractPlus]. Ca^2+^, Ba^2+^ and Sr^2+^ permeate TRPP3, but reduce inward currents carried by Na^+^. Mg^2^+ is largely impermeant and exerts a voltage dependent inhibition that increases with hyperpolarization.

### TRPV (vanilloid) family

Members of the TRPV family (reviewed by [http://www.ncbi.nlm.nih.gov/pubmed/18220815?dopt=AbstractPlus]) can broadly be divided into the non‐selective cation channels, TRPV1‐4 and the more calcium selective channels TRPV5 and TRPV6.

### TRPV1‐V4 subfamily

TRPV1 is involved in the development of thermal hyperalgesia following inflammation and may contribute to the detection of noxius heat (reviewed by [http://www.ncbi.nlm.nih.gov/pubmed/17217056?dopt=AbstractPlus, http://www.ncbi.nlm.nih.gov/pubmed/17349697?dopt=AbstractPlus, http://www.ncbi.nlm.nih.gov/pubmed/17464295?dopt=AbstractPlus]). Numerous splice variants of TRPV1 have been described, some of which modulate the activity of TRPV1, or act in a dominant negative manner when co‐expressed with TRPV1 [http://www.ncbi.nlm.nih.gov/pubmed/20515731?dopt=AbstractPlus]. The pharmacology of TRPV1 channels is discussed in detail in [http://www.ncbi.nlm.nih.gov/pubmed/19063991?dopt=AbstractPlus] and [http://www.ncbi.nlm.nih.gov/pubmed/19297520?dopt=AbstractPlus]. TRPV2 is probably not a thermosensor in man [http://www.ncbi.nlm.nih.gov/pubmed/21832173?dopt=AbstractPlus], but has recently been implicated in innate immunity [http://www.ncbi.nlm.nih.gov/pubmed/20118928?dopt=AbstractPlus]. TRPV3 and TRPV4 are both thermosensitive. There are claims that TRPV4 is also mechanosensitive, but this has not been established to be within a physiological range in a native environment [http://www.ncbi.nlm.nih.gov/pubmed/24305161?dopt=AbstractPlus, http://www.ncbi.nlm.nih.gov/pubmed/24305160?dopt=AbstractPlus].

### TRPV5/V6 subfamily

TRPV5 and TRPV6 are highly expressed in placenta, bone, and kidney. Under physiological conditions, TRPV5 and TRPV6 are calcium selective channels involved in the absorption and reabsorption of calcium across intestinal and kidney tubule epithelia (reviewed by [http://www.ncbi.nlm.nih.gov/pubmed/18596722?dopt=AbstractPlus, http://www.ncbi.nlm.nih.gov/pubmed/24756713?dopt=AbstractPlus, http://www.ncbi.nlm.nih.gov/pubmed/24756712?dopt=AbstractPlus, http://www.ncbi.nlm.nih.gov/pubmed/17217060?dopt=AbstractPlus]).

**Table:** 

Nomenclature	http://www.guidetopharmacology.org/GRAC/ObjectDisplayForward?objectId=507	http://www.guidetopharmacology.org/GRAC/ObjectDisplayForward?objectId=508
HGNC, UniProt	https://www.genenames.org/data/gene‐symbol‐report/#!/hgnc_id/HGNC:12716, http://www.uniprot.org/uniprot/Q8NER1	https://www.genenames.org/data/gene‐symbol‐report/#!/hgnc_id/HGNC:18082, http://www.uniprot.org/uniprot/Q9Y5S1
Other chemical activators	NO‐mediated cysteine S‐nitrosylation	–
Physical activators	depolarization (V_½_ 0 mV at 35°C), noxious heat (> 43°C at pH 7.4)	–
Endogenous activators	extracellular http://www.guidetopharmacology.org/GRAC/LigandDisplayForward?ligandId=2346 (at 37°C) (pEC_50_ 5.4), http://www.guidetopharmacology.org/GRAC/LigandDisplayForward?ligandId=2481 (Agonist) (pEC_50_ 5.1) [‐60mV] [http://www.ncbi.nlm.nih.gov/pubmed/10823958?dopt=AbstractPlus] – Rat, http://www.guidetopharmacology.org/GRAC/LigandDisplayForward?ligandId=2364 (pEC_50_ 5) [http://www.ncbi.nlm.nih.gov/pubmed/12761211?dopt=AbstractPlus], http://www.guidetopharmacology.org/GRAC/LigandDisplayForward?ligandId=2487 (Agonist) (pEC_50_ 4.9) [‐60mV] [http://www.ncbi.nlm.nih.gov/pubmed/10823958?dopt=AbstractPlus] – Rat, http://www.guidetopharmacology.org/GRAC/LigandDisplayForward?ligandId=3390	–
Activators	http://www.guidetopharmacology.org/GRAC/LigandDisplayForward?ligandId=2491 (Agonist) (pEC_50_ 8.4) [physiological voltage] [http://www.ncbi.nlm.nih.gov/pubmed/11301059?dopt=AbstractPlus], http://www.guidetopharmacology.org/GRAC/LigandDisplayForward?ligandId=2486 (Agonist) (pEC_50_ 7.5) [‐100mV – 160mV] [http://www.ncbi.nlm.nih.gov/pubmed/15306801?dopt=AbstractPlus], http://www.guidetopharmacology.org/GRAC/LigandDisplayForward?ligandId=2489 (Agonist) (pEC_50_ 4.4–5) [‐70mV] [http://www.ncbi.nlm.nih.gov/pubmed/15685214?dopt=AbstractPlus], http://www.guidetopharmacology.org/GRAC/LigandDisplayForward?ligandId=2422, http://www.guidetopharmacology.org/GRAC/LigandDisplayForward?ligandId=2494, http://www.guidetopharmacology.org/GRAC/LigandDisplayForward?ligandId=4283 [http://www.ncbi.nlm.nih.gov/pubmed/15356216?dopt=AbstractPlus]	http://www.guidetopharmacology.org/GRAC/LigandDisplayForward?ligandId=2433 (pEC_50_ 5) [http://www.ncbi.nlm.nih.gov/pubmed/17395593?dopt=AbstractPlus, http://www.ncbi.nlm.nih.gov/pubmed/18550765?dopt=AbstractPlus] – Rat, http://www.guidetopharmacology.org/GRAC/LigandDisplayForward?ligandId=2424 (pEC_50_ 4.8) [http://www.ncbi.nlm.nih.gov/pubmed/18550765?dopt=AbstractPlus] – Rat, http://www.guidetopharmacology.org/GRAC/LigandDisplayForward?ligandId=4150 (pEC_50_ 4.5) [http://www.ncbi.nlm.nih.gov/pubmed/18550765?dopt=AbstractPlus], http://www.guidetopharmacology.org/GRAC/LigandDisplayForward?ligandId=4357 (pEC_50_ 4.5) [http://www.ncbi.nlm.nih.gov/pubmed/17850966?dopt=AbstractPlus] – Rat, http://www.guidetopharmacology.org/GRAC/LigandDisplayForward?ligandId=2433 (Agonist) (pEC_50_ 3.8–3.9) [physiological voltage] [http://www.ncbi.nlm.nih.gov/pubmed/15194687?dopt=AbstractPlus, http://www.ncbi.nlm.nih.gov/pubmed/17673572?dopt=AbstractPlus] – Mouse, http://www.guidetopharmacology.org/GRAC/LigandDisplayForward?ligandId=2494 (Agonist) Concentration range: 1×10^−4^M [‐80mV] [http://www.ncbi.nlm.nih.gov/pubmed/15722340?dopt=AbstractPlus, http://www.ncbi.nlm.nih.gov/pubmed/17673572?dopt=AbstractPlus] – Mouse
Selective activators	http://www.guidetopharmacology.org/GRAC/LigandDisplayForward?ligandId=2488 (Agonist) (pEC_50_ 7.7) [physiological voltage] [http://www.ncbi.nlm.nih.gov/pubmed/11301059?dopt=AbstractPlus], http://www.guidetopharmacology.org/GRAC/LigandDisplayForward?ligandId=4178 (pEC_50_ 6.6) [physiological voltage] [http://www.ncbi.nlm.nih.gov/pubmed/20510930?dopt=AbstractPlus] – Rat	–
Channel blockers	http://www.guidetopharmacology.org/GRAC/LigandDisplayForward?ligandId=4109 (pIC_50_ 8.4), http://www.guidetopharmacology.org/GRAC/LigandDisplayForward?ligandId=4110 (pIC_50_ 8), http://www.guidetopharmacology.org/GRAC/LigandDisplayForward?ligandId=6347 (Inhibition) (pIC_50_ 7.8) [physiological voltage] [http://www.ncbi.nlm.nih.gov/pubmed/15615864?dopt=AbstractPlus], http://www.guidetopharmacology.org/GRAC/LigandDisplayForward?ligandId=2460 (Antagonist) (pIC_50_ 7.5) [http://www.ncbi.nlm.nih.gov/pubmed/24055075?dopt=AbstractPlus], http://www.guidetopharmacology.org/GRAC/LigandDisplayForward?ligandId=2461 (Antagonist) (pIC_50_ 7.4) [‐60mV] [http://www.ncbi.nlm.nih.gov/pubmed/11226139?dopt=AbstractPlus], http://www.guidetopharmacology.org/GRAC/LigandDisplayForward?ligandId=2432 (pIC_50_ 6.7–7)	http://www.guidetopharmacology.org/GRAC/LigandDisplayForward?ligandId=2432 (pIC_50_ 6.2), http://www.guidetopharmacology.org/GRAC/LigandDisplayForward?ligandId=4331 (Inhibition) Concentration range: 5×10^−4^M [http://www.ncbi.nlm.nih.gov/pubmed/17673572?dopt=AbstractPlus] – Mouse
Selective channel blockers	http://www.guidetopharmacology.org/GRAC/LigandDisplayForward?ligandId=4129 (pIC_50_ 9) [http://www.ncbi.nlm.nih.gov/pubmed/20307063?dopt=AbstractPlus], http://www.guidetopharmacology.org/GRAC/LigandDisplayForward?ligandId=4130 (pIC_50_ 8.4) [http://www.ncbi.nlm.nih.gov/pubmed/17585751?dopt=AbstractPlus] – Rat, http://www.guidetopharmacology.org/GRAC/LigandDisplayForward?ligandId=4117 (pIC_50_ 8.3) [http://www.ncbi.nlm.nih.gov/pubmed/15837819?dopt=AbstractPlus], http://www.guidetopharmacology.org/GRAC/LigandDisplayForward?ligandId=4120 (pIC_50_ 8.3) [http://www.ncbi.nlm.nih.gov/pubmed/17660385?dopt=AbstractPlus], http://www.guidetopharmacology.org/GRAC/LigandDisplayForward?ligandId=4309 (pIC_50_ 8.2) [http://www.ncbi.nlm.nih.gov/pubmed/14654105?dopt=AbstractPlus], http://www.guidetopharmacology.org/GRAC/LigandDisplayForward?ligandId=4230 (Antagonist) (pIC_50_ 8) [http://www.ncbi.nlm.nih.gov/pubmed/12237342?dopt=AbstractPlus] – Rat, http://www.guidetopharmacology.org/GRAC/LigandDisplayForward?ligandId=4228 (Antagonist) (pIC_50_ 7.8) [physiological voltage] [http://www.ncbi.nlm.nih.gov/pubmed/15771431?dopt=AbstractPlus], http://www.guidetopharmacology.org/GRAC/LigandDisplayForward?ligandId=4310 (Antagonist) (p*K*_B_ 7.7), http://www.guidetopharmacology.org/GRAC/LigandDisplayForward?ligandId=4311 (Antagonist) (pIC_50_ 7.1) [http://www.ncbi.nlm.nih.gov/pubmed/17392405?dopt=AbstractPlus]	–
Labelled ligands	http://www.guidetopharmacology.org/GRAC/LigandDisplayForward?ligandId=4076 (Channel blocker) (p*K*_d_ 8.5) [http://www.ncbi.nlm.nih.gov/pubmed/17660385?dopt=AbstractPlus], http://www.guidetopharmacology.org/GRAC/LigandDisplayForward?ligandId=2492 (Channel blocker, Antagonist) (pIC_50_ 8.4) [‐50mV] [http://www.ncbi.nlm.nih.gov/pubmed/11125018?dopt=AbstractPlus] – Rat, http://www.guidetopharmacology.org/GRAC/LigandDisplayForward?ligandId=4091 (Activator)	–
Functional Characteristics	γ = 35 pS at – 60 mV; 77 pS at + 60 mV, conducts mono and di‐valent cations with a selectivity for divalents (P_Ca_/P_Na_ = 9.6); voltage‐ and time‐ dependent outward rectification; potentiated by ethanol; activated/potentiated/upregulated by PKC stimulation; extracellular acidification facilitates activation by PKC; desensitisation inhibited by PKA; inhibited by Ca^2+^/ calmodulin; cooling reduces vanilloid‐evoked currents; may be tonically active at body temperature	Conducts mono‐ and di‐valent cations (P_Ca_/P_Na_ = 0.9–2.9); dual (inward and outward) rectification; current increases upon repetitive activation by heat; translocates to cell surface in response to IGF‐1 to induce a constitutively active conductance, translocates to the cell surface in response to membrane stretch

**Table:** 

Nomenclature	http://www.guidetopharmacology.org/GRAC/ObjectDisplayForward?objectId=509	http://www.guidetopharmacology.org/GRAC/ObjectDisplayForward?objectId=510
HGNC, UniProt	https://www.genenames.org/data/gene‐symbol‐report/#!/hgnc_id/HGNC:18084, http://www.uniprot.org/uniprot/Q8NET8	https://www.genenames.org/data/gene‐symbol‐report/#!/hgnc_id/HGNC:18083, http://www.uniprot.org/uniprot/Q9HBA0
Other chemical activators	NO‐mediated cysteine S‐nitrosylation	Epoxyeicosatrieonic acids and NO‐mediated cysteine S‐nitrosylation
Physical activators	depolarization (V_½_ +80 mV, reduced to more negative values following heat stimuli), heat (23°C ‐ 39°C, temperature threshold reduces with repeated heat challenge)	Constitutively active, heat (> 24°C ‐ 32°C), mechanical stimuli
Activators	http://www.guidetopharmacology.org/GRAC/LigandDisplayForward?ligandId=4220 (pEC_50_ 4.8) [http://www.ncbi.nlm.nih.gov/pubmed/18492727?dopt=AbstractPlus] – Mouse, http://www.guidetopharmacology.org/GRAC/LigandDisplayForward?ligandId=2433 (Full agonist) (pEC_50_ 4.6) [‐80mV – 80mV] [http://www.ncbi.nlm.nih.gov/pubmed/15175387?dopt=AbstractPlus] – Mouse, http://www.guidetopharmacology.org/GRAC/LigandDisplayForward?ligandId=2494 (Full agonist) (pEC_50_ 4.1–4.2) [*voltage dependent* ‐80mV – 80mV] [http://www.ncbi.nlm.nih.gov/pubmed/15722340?dopt=AbstractPlus] – Mouse, http://www.guidetopharmacology.org/GRAC/LigandDisplayForward?ligandId=2499 (Full agonist) (pEC_50_ 3.3) [http://www.ncbi.nlm.nih.gov/pubmed/16617338?dopt=AbstractPlus] – Mouse, http://www.guidetopharmacology.org/GRAC/LigandDisplayForward?ligandId=2425 (Full agonist) (pEC_50_ 2.5) [http://www.ncbi.nlm.nih.gov/pubmed/16617338?dopt=AbstractPlus] – Mouse, http://www.guidetopharmacology.org/GRAC/LigandDisplayForward?ligandId=2422 (Full agonist) (pEC_50_ 2) [http://www.ncbi.nlm.nih.gov/pubmed/15746429?dopt=AbstractPlus] – Mouse, http://www.guidetopharmacology.org/GRAC/LigandDisplayForward?ligandId=2430 (pEC_50_ 1.7) [‐80mV – 80mV] [http://www.ncbi.nlm.nih.gov/pubmed/16829128?dopt=AbstractPlus] – Mouse, http://www.guidetopharmacology.org/GRAC/LigandDisplayForward?ligandId=2497 (Full agonist) Concentration range: 5×10^−4^M [‐80mV – 80mV] [http://www.ncbi.nlm.nih.gov/pubmed/16617338?dopt=AbstractPlus] – Mouse	http://www.guidetopharmacology.org/GRAC/LigandDisplayForward?ligandId=2341 (Agonist) (pEC_50_ 7.9) [physiological voltage] [http://www.ncbi.nlm.nih.gov/pubmed/12970074?dopt=AbstractPlus], http://www.guidetopharmacology.org/GRAC/LigandDisplayForward?ligandId=2391 (pEC_50_ 5) [http://www.ncbi.nlm.nih.gov/pubmed/12879072?dopt=AbstractPlus] – Mouse
Selective activators	http://www.guidetopharmacology.org/GRAC/LigandDisplayForward?ligandId=4111 (pEC_50_ 3.4) [http://www.ncbi.nlm.nih.gov/pubmed/17420775?dopt=AbstractPlus] – Mouse	http://www.guidetopharmacology.org/GRAC/LigandDisplayForward?ligandId=4205 (pEC_50_ 8.7) [physiological voltage] [http://www.ncbi.nlm.nih.gov/pubmed/18499743?dopt=AbstractPlus], http://www.guidetopharmacology.org/GRAC/LigandDisplayForward?ligandId=4105 (pEC_50_ 7.1) [physiological voltage] [http://www.ncbi.nlm.nih.gov/pubmed/19361196?dopt=AbstractPlus] – Mouse, http://www.guidetopharmacology.org/GRAC/LigandDisplayForward?ligandId=2500 (Agonist) (pEC_50_ 6.5) [http://www.ncbi.nlm.nih.gov/pubmed/12970074?dopt=AbstractPlus], http://www.guidetopharmacology.org/GRAC/LigandDisplayForward?ligandId=4295 (pEC_50_ 6.1) [physiological voltage] [http://www.ncbi.nlm.nih.gov/pubmed/19737537?dopt=AbstractPlus], http://www.guidetopharmacology.org/GRAC/LigandDisplayForward?ligandId=2502 (Agonist) (pEC_50_ 6) [‐60mV] [http://www.ncbi.nlm.nih.gov/pubmed/16899456?dopt=AbstractPlus] – Mouse
Channel blockers	http://www.guidetopharmacology.org/GRAC/LigandDisplayForward?ligandId=2432 (Inhibition) (pIC_50_ 6) [http://www.ncbi.nlm.nih.gov/pubmed/12016205?dopt=AbstractPlus] – Mouse, http://www.guidetopharmacology.org/GRAC/LigandDisplayForward?ligandId=2498 (Antagonist) (pIC_50_ 5–5.2) [‐80mV – 80mV] [http://www.ncbi.nlm.nih.gov/pubmed/15722340?dopt=AbstractPlus] – Mouse, http://www.guidetopharmacology.org/GRAC/LigandDisplayForward?ligandId=10303 (Inhibition) (pIC_50_ 5.2) [http://www.ncbi.nlm.nih.gov/pubmed/30377214?dopt=AbstractPlus], http://www.guidetopharmacology.org/GRAC/LigandDisplayForward?ligandId=10302 (Inhibition) (pIC_50_ 4.4) [http://www.ncbi.nlm.nih.gov/pubmed/30108138?dopt=AbstractPlus]	http://www.guidetopharmacology.org/GRAC/LigandDisplayForward?ligandId=2432 (Inhibition) (pIC_50_ 6.7) [http://www.ncbi.nlm.nih.gov/pubmed/12151520?dopt=AbstractPlus] – Rat, http://www.guidetopharmacology.org/GRAC/LigandDisplayForward?ligandId=2426, http://www.guidetopharmacology.org/GRAC/LigandDisplayForward?ligandId=2434
Selective channel blockers	–	http://www.guidetopharmacology.org/GRAC/LigandDisplayForward?ligandId=4213 (Inhibition) (pIC_50_ 7.3) [‐40mV] [http://www.ncbi.nlm.nih.gov/pubmed/20956320?dopt=AbstractPlus], http://www.guidetopharmacology.org/GRAC/LigandDisplayForward?ligandId=10304 (Antagonist) (pIC_50_ 6.2) [http://www.ncbi.nlm.nih.gov/pubmed/26235950?dopt=AbstractPlus], http://www.guidetopharmacology.org/GRAC/LigandDisplayForward?ligandId=4294 (Inhibition) (pIC_50_ 5.6) [physiological voltage] [http://www.ncbi.nlm.nih.gov/pubmed/19737537?dopt=AbstractPlus]
Functional Characteristics	γ = 197 pS at = +40 to +80 mV, 48 pS at negative potentials; conducts mono‐ and di‐valent cations; outward rectification; potentiated by arachidonic acid	γ = 60 pS at –60 mV, 90‐100 pS at +60 mV; conducts mono‐ and di‐valent cations with a preference for divalents (P_Ca_/P_Na_ =6–10); dual (inward and outward) rectification; potentiated by intracellular Ca^2+^ *via* Ca^2+^/ calmodulin; inhibited by elevated intracellular Ca^2+^ *via* an unknown mechanism (IC_50_ = 0.4 *μ*M)

**Table:** 

Nomenclature	http://www.guidetopharmacology.org/GRAC/ObjectDisplayForward?objectId=511	http://www.guidetopharmacology.org/GRAC/ObjectDisplayForward?objectId=512
HGNC, UniProt	https://www.genenames.org/data/gene‐symbol‐report/#!/hgnc_id/HGNC:3145, http://www.uniprot.org/uniprot/Q9NQA5	https://www.genenames.org/data/gene‐symbol‐report/#!/hgnc_id/HGNC:14006, http://www.uniprot.org/uniprot/Q9H1D0
Other channel blockers	http://www.guidetopharmacology.org/GRAC/LigandDisplayForward?ligandId=2525 = http://www.guidetopharmacology.org/GRAC/LigandDisplayForward?ligandId=4164 = http://www.guidetopharmacology.org/GRAC/LigandDisplayForward?ligandId=2426 > http://www.guidetopharmacology.org/GRAC/LigandDisplayForward?ligandId=2440 > http://www.guidetopharmacology.org/GRAC/LigandDisplayForward?ligandId=566 > http://www.guidetopharmacology.org/GRAC/LigandDisplayForward?ligandId=2434 > http://www.guidetopharmacology.org/GRAC/LigandDisplayForward?ligandId=4160 > Fe^2^	–
Activators	constitutively active (with strong buffering of intracellular Ca^2+^)	http://www.guidetopharmacology.org/GRAC/LigandDisplayForward?ligandId=2433 (Potentiation) constitutively active (with strong buffering of intracellular Ca^2+^)
Channel blockers	http://www.guidetopharmacology.org/GRAC/LigandDisplayForward?ligandId=2432 (pIC_50_ 6.9), http://www.guidetopharmacology.org/GRAC/LigandDisplayForward?ligandId=708	http://www.guidetopharmacology.org/GRAC/LigandDisplayForward?ligandId=2432 (Antagonist) (pIC_50_ 5) [‐80mV] [http://www.ncbi.nlm.nih.gov/pubmed/11744752?dopt=AbstractPlus] – Mouse, http://www.guidetopharmacology.org/GRAC/LigandDisplayForward?ligandId=2440, http://www.guidetopharmacology.org/GRAC/LigandDisplayForward?ligandId=2434, http://www.guidetopharmacology.org/GRAC/LigandDisplayForward?ligandId=708
Functional Characteristics	γ = 59–78 pS for monovalent ions at negative potentials, conducts mono‐ and di‐valents with high selectivity for divalents (P_Ca_/P_Na_ > 107); voltage‐ and time‐ dependent inward rectification; inhibited by intracellular Ca^2+^ promoting fast inactivation and slow downregulation; feedback inhibition by Ca^2+^ reduced by calcium binding protein 80‐K‐H; inhibited by extracellular and intracellular acidosis; upregulated by 1,25‐dihydrovitamin D3	γ = 58–79 pS for monovalent ions at negative potentials, conducts mono‐ and di‐valents with high selectivity for divalents (P_Ca_/P_Na_ > 130); voltage‐ and time‐dependent inward rectification; inhibited by intracellular Ca^2+^ promoting fast and slow inactivation; gated by voltage‐dependent channel blockade by intracellular Mg^2+^; slow inactivation due to Ca^2+^‐dependent calmodulin binding; phosphorylation by PKC inhibits Ca^2+^‐calmodulin binding and slow inactivation; upregulated by 1,25‐dihydroxyvitamin D3

### Comments

Activation of TRPV1 by depolarisation is strongly temperature‐dependent via a channel opening rate that increases with increasing temperature. The V_½_ is shifted in the hyperpolarizing direction both by increasing temperature and by exogenous agonists [http://www.ncbi.nlm.nih.gov/pubmed/15306801?dopt=AbstractPlus]. TRPV3 channel dysfunction caused by genetic gain‐of‐function mutations is implicated in the pathogenesis of skin inflammation, dermatitis, and chronic itch. In rodents, a sponateous gain‐of‐function matation of the TRPV3 gene causes the development of skin lesions with pruritus and dermatitis [http://www.ncbi.nlm.nih.gov/pubmed/16858425?dopt=AbstractPlus, http://www.ncbi.nlm.nih.gov/pubmed/22405088?dopt=AbstractPlus]. In contrast to other thermoTRP channels, TRPV3 sensitizes rather than desensitizes, upon repeated stimulation with either heat or agonists [http://www.ncbi.nlm.nih.gov/pubmed/15175387?dopt=AbstractPlus, http://www.ncbi.nlm.nih.gov/pubmed/22006988?dopt=AbstractPlus, http://www.ncbi.nlm.nih.gov/pubmed/12077604?dopt=AbstractPlus]. The sensitivity of TRPV4 to heat, but not http://www.guidetopharmacology.org/GRAC/LigandDisplayForward?ligandId=2500 is lost upon patch excision. TRPV4 is activated by http://www.guidetopharmacology.org/GRAC/LigandDisplayForward?ligandId=2364 and http://www.guidetopharmacology.org/GRAC/LigandDisplayForward?ligandId=2391 following P450 epoxygenase‐dependent metabolism to http://www.guidetopharmacology.org/GRAC/LigandDisplayForward?ligandId=6304 (reviewed by [http://www.ncbi.nlm.nih.gov/pubmed/14707014?dopt=AbstractPlus]). Activation of TRPV4 by cell swelling, but not heat, or phorbol esters, is mediated via the formation of epoxyeicosatrieonic acids. Phorbol esters bind directly to TRPV4. Different TRPV4 mutations load to a broad spectrum of dominant skeletal dysplasias [http://www.ncbi.nlm.nih.gov/pubmed/19232556?dopt=AbstractPlus, http://www.ncbi.nlm.nih.gov/pubmed/18587396?dopt=AbstractPlus] and spinal muscular atrophies and hereditary motor and sensory neuropathies [http://www.ncbi.nlm.nih.gov/pubmed/20037588?dopt=AbstractPlus, http://www.ncbi.nlm.nih.gov/pubmed/20037587?dopt=AbstractPlus]. Similar mutations were also found in patients with Charcot‐Marie‐Tooth disease type 2C [http://www.ncbi.nlm.nih.gov/pubmed/20037586?dopt=AbstractPlus]. TRPV5 preferentially conducts Ca^2+^ under physiological conditions, but in the absence of extracellular Ca^2+^, conducts monovalent cations. Single channel conductances listed for TRPV5 and TRPV6 were determined in divalent cation‐free extracellular solution. Ca^2+^‐induced inactivation occurs at hyperpolarized potentials when Ca^2+^ is present extracellularly. Single channel events cannot be resolved (probably due to greatly reduced conductance) in the presence of extracellular divalent cations. Measurements of P_Ca_/P_Na_ for TRPV5 and TRPV6 are dependent upon ionic conditions due to anomalous mole fraction behaviour. Blockade of TRPV5 and TRPV6 by extracellular Mg^2+^ is voltage‐dependent. Intracellular Mg^2+^ also exerts a voltage dependent block that is alleviated by hyperpolarization and contributes to the time‐dependent activation and deactivation of TRPV6 mediated monovalent cation currents. TRPV5 and TRPV6 differ in their kinetics of Ca^2+^‐dependent inactivation and recovery from inactivation. TRPV5 and TRPV6 function as homo‐ and hetero‐tetramers.

### Further reading on Transient Receptor Potential channels

Aghazadeh Tabrizi M *et al.* (2017) Medicinal Chemistry, Pharmacology, and Clinical Implications of TRPV1 Receptor Antagonists. *Med Res Rev* **37**: 936‐983 https://www.ncbi.nlm.nih.gov/pubmed/27976413?dopt=AbstractPlus

Basso L *et al.* (2017) Transient Receptor Potential Channels in neuropathic pain. *Curr Opin Pharmacol* **32**: 9‐15 https://www.ncbi.nlm.nih.gov/pubmed/27835802?dopt=AbstractPlus

Ciardo MG *et al.* (2017) Lipids as central modulators of sensory TRP channels. *Biochim. Biophys. Acta* **1859**: 1615‐1628 [https://www.ncbi.nlm.nih.gov/pubmed/28432033?dopt=AbstractPlus

Clapham DE *et al.* (2003) International Union of Pharmacology. XLIII. Compendium of voltage‐gated ion channels: transient receptor potential channels. *Pharmacol. Rev.* **55**: 591‐6 https://www.ncbi.nlm.nih.gov/pubmed/14657417?dopt=AbstractPlus

Diaz‐Franulic I *et al.* (2016) Allosterism and Structure in Thermally Activated Transient Receptor Potential Channels. *Annu Rev Biophys* **45**: 371‐98 https://www.ncbi.nlm.nih.gov/pubmed/27297398?dopt=AbstractPlus

Emir TLR. (2017) Neurobiology of TRP Channels *Neurobiology of TRP Channels* https://www.ncbi.nlm.nih.gov/pubmed/29356487?dopt=AbstractPlus

Grayson TH *et al.* (2017) Transient receptor potential canonical type 3 channels: Interactions, role and relevance ‐ A vascular focus. *Pharmacol. Ther.* **174**: 79‐96 https://www.ncbi.nlm.nih.gov/pubmed/28223224?dopt=AbstractPlus

Nilius B *et al.* (2014) Mammalian transient receptor potential (TRP) cation channels. Preface. *Handb Exp Pharmacol* **223**: v ‐ vi https://www.ncbi.nlm.nih.gov/pubmed/25296415?dopt=AbstractPlus

Rubaiy HN. (2019) Treasure troves of pharmacological tools to study transient receptor potential canonical 1/4/5 channels. *Br. J. Pharmacol.* **176**: 832‐846 https://www.ncbi.nlm.nih.gov/pubmed/30656647?dopt=AbstractPlus

Wu LJ *et al*. (2010) International Union of Basic and Clinical Pharmacology. LXXVI. Current progress in the mammalian TRP ion channel family. *Pharmacol. Rev.* **62**: 381‐404 https://www.ncbi.nlm.nih.gov/pubmed/20716668?dopt=AbstractPlus

Zhu MX. (2011) Various *TRP Channels (CRC Press/Taylor & Francis)* [https://www.ncbi.nlm.nih.gov/pubmed/22593967?dopt=AbstractPlus

Zierler S *et al.* (2017) TRPM channels as potential therapeutic targets against pro‐inflammatory diseases. *Cell Calcium* **67**: 105‐115 https://www.ncbi.nlm.nih.gov/pubmed/28549569?dopt=AbstractPlus

## http://www.guidetopharmacology.org/GRAC/FamilyDisplayForward?familyId=80

### Overview

Calcium (Ca^2+^) channels are voltage‐gated ion channels present in the membrane of most excitable cells. The nomenclature for Ca^2+^channels was proposed by [http://www.ncbi.nlm.nih.gov/pubmed/10774722?dopt=AbstractPlus] and **approved by the NC‐IUPHAR Subcommittee on Ca^2+^ channels**[http://www.ncbi.nlm.nih.gov/pubmed/16382099?dopt=AbstractPlus]. Ca^2+^ channels form hetero‐oligomeric complexes. The α1 subunit is pore‐forming and provides the binding site(s) for practically all agonists and antagonists. The 10 cloned α1‐subunits can be grouped into three families: (1) the high‐voltage activated dihydropyridine‐sensitive (L‐type, CaV1.x) channels; (2) the highvoltage activated dihydropyridine‐insensitive (CaV2.x) channels and (3) the low‐voltage‐activated (T‐type, CaV3.x) channels. Each α1 subunit has four homologous repeats (I–IV),each repeat having six transmembrane domains and a pore‐forming region between transmembrane domains S5 and S6. Gating is thought to be associated with the membrane‐spanning S4 segment, which contains highly conserved positive charges. Many of the α1‐subunit genes give rise to alternatively spliced products. At least for high‐voltage activated channels, it is likely that native channels comprise coassemblies of α1, β and α2–*δ* subunits. The γ subunits have not been proven to associate with channels other than the α1s skeletal muscle Cav1.1 channel. The α2–*δ*1 and α2–*δ*2 subunits bind http://www.guidetopharmacology.org/GRAC/LigandDisplayForward?ligandId=5483 and http://www.guidetopharmacology.org/GRAC/LigandDisplayForward?ligandId=5484.

**Table:** 

Nomenclature	http://www.guidetopharmacology.org/GRAC/ObjectDisplayForward?objectId=528	http://www.guidetopharmacology.org/GRAC/ObjectDisplayForward?objectId=529	http://www.guidetopharmacology.org/GRAC/ObjectDisplayForward?objectId=530	http://www.guidetopharmacology.org/GRAC/ObjectDisplayForward?objectId=531
HGNC, UniProt	https://www.genenames.org/data/gene‐symbol‐report/#!/hgnc_id/HGNC:1397, http://www.uniprot.org/uniprot/Q13698	https://www.genenames.org/data/gene‐symbol‐report/#!/hgnc_id/HGNC:1390, http://www.uniprot.org/uniprot/Q13936	https://www.genenames.org/data/gene‐symbol‐report/#!/hgnc_id/HGNC:1391, http://www.uniprot.org/uniprot/Q01668	https://www.genenames.org/data/gene‐symbol‐report/#!/hgnc_id/HGNC:1393, http://www.uniprot.org/uniprot/O60840
Activators	http://www.guidetopharmacology.org/GRAC/LigandDisplayForward?ligandId=2518 (pEC_50_ ∼7.8), http://www.guidetopharmacology.org/GRAC/LigandDisplayForward?ligandId=4065 (pEC_50_ ∼7.8)	http://www.guidetopharmacology.org/GRAC/LigandDisplayForward?ligandId=4065 (pEC_50_ ∼7.8), http://www.guidetopharmacology.org/GRAC/LigandDisplayForward?ligandId=2518 Concentration range: 1×10−^6^M‐5×10−^6^M [http://www.ncbi.nlm.nih.gov/pubmed/12842134?dopt=AbstractPlus] – Rat	http://www.guidetopharmacology.org/GRAC/LigandDisplayForward?ligandId=2518 http://www.guidetopharmacology.org/GRAC/LigandDisplayForward?ligandId=4065 (pEC_50_ ∼7.8)	http://www.guidetopharmacology.org/GRAC/LigandDisplayForward?ligandId=4065 (pEC_50_ ∼7.8)
Gating inhibitors	http://www.guidetopharmacology.org/GRAC/LigandDisplayForward?ligandId=2514 (Antagonist) (pIC_50_ 6.3) Concentration range: 1×10−^7^M‐1×10−^4^M [*voltage dependent* ‐90mV] [http://www.ncbi.nlm.nih.gov/pubmed/2451745?dopt=AbstractPlus] – Rat, http://www.guidetopharmacology.org/GRAC/LigandDisplayForward?ligandId=2523 (Antagonist) (pIC_50_ ∼6) [‐70mV], http://www.guidetopharmacology.org/GRAC/LigandDisplayForward?ligandId=2334 (Antagonist) (pIC_50_ 6) [‐80mV] [http://www.ncbi.nlm.nih.gov/pubmed/2458429?dopt=AbstractPlus] – Rat	http://www.guidetopharmacology.org/GRAC/LigandDisplayForward?ligandId=4488 (arterial smooth muscle‐like activity) (pIC_50_ 8.8) [http://www.ncbi.nlm.nih.gov/pubmed/28592699?dopt=AbstractPlus], http://www.guidetopharmacology.org/GRAC/LigandDisplayForward?ligandId=4488 (dopamine neuron neuron‐like activity) (pIC_50_ 8.5) [http://www.ncbi.nlm.nih.gov/pubmed/28592699?dopt=AbstractPlus], http://www.guidetopharmacology.org/GRAC/LigandDisplayForward?ligandId=2514 (Antagonist) (pIC_50_ 7.7) [‐80mV] [http://www.ncbi.nlm.nih.gov/pubmed/11045961?dopt=AbstractPlus, http://www.ncbi.nlm.nih.gov/pubmed/7506331?dopt=AbstractPlus] – Rat, http://www.guidetopharmacology.org/GRAC/LigandDisplayForward?ligandId=2523 (Antagonist) (pIC_50_ 6.8) [‐80mV] [http://www.ncbi.nlm.nih.gov/pubmed/11487617?dopt=AbstractPlus] – Rat	http://www.guidetopharmacology.org/GRAC/LigandDisplayForward?ligandId=2334 (Inhibition) (pIC_50_ 8.4) [http://www.ncbi.nlm.nih.gov/pubmed/19029287?dopt=AbstractPlus], http://www.guidetopharmacology.org/GRAC/LigandDisplayForward?ligandId=4488 (dopamine neuron‐like activity; splice variant‐dependent) (pIC_50_ 7.8–8.2) [http://www.ncbi.nlm.nih.gov/pubmed/28592699?dopt=AbstractPlus], http://www.guidetopharmacology.org/GRAC/LigandDisplayForward?ligandId=2514 (Antagonist) (pIC_50_ 7.7) [http://www.ncbi.nlm.nih.gov/pubmed/19029287?dopt=AbstractPlus], http://www.guidetopharmacology.org/GRAC/LigandDisplayForward?ligandId=2523 (Antagonist) (pIC_50_ 5.7–6.6) [‐80mV – ‐40mV] [http://www.ncbi.nlm.nih.gov/pubmed/11514547?dopt=AbstractPlus, http://www.ncbi.nlm.nih.gov/pubmed/11487617?dopt=AbstractPlus] – Rat	http://www.guidetopharmacology.org/GRAC/LigandDisplayForward?ligandId=2514 (Antagonist) (pIC_50_ 6) [‐100mV] [http://www.ncbi.nlm.nih.gov/pubmed/14973233?dopt=AbstractPlus], http://www.guidetopharmacology.org/GRAC/LigandDisplayForward?ligandId=2523 (Antagonist) (pIC_50_ ∼6) [‐70mV], http://www.guidetopharmacology.org/GRAC/LigandDisplayForward?ligandId=2334 (Antagonist) (pIC_50_ ∼6) [‐70mV]
Channel blockers	http://www.guidetopharmacology.org/GRAC/LigandDisplayForward?ligandId=2298 (Antagonist), http://www.guidetopharmacology.org/GRAC/LigandDisplayForward?ligandId=2406 (Antagonist)	http://www.guidetopharmacology.org/GRAC/LigandDisplayForward?ligandId=2298 (Antagonist), http://www.guidetopharmacology.org/GRAC/LigandDisplayForward?ligandId=2406 (Antagonist)	http://www.guidetopharmacology.org/GRAC/LigandDisplayForward?ligandId=2406 (Antagonist)	http://www.guidetopharmacology.org/GRAC/LigandDisplayForward?ligandId=2298 (pIC_50_ 4) [‐80mV] [http://www.ncbi.nlm.nih.gov/pubmed/14744918?dopt=AbstractPlus] – Mouse, http://www.guidetopharmacology.org/GRAC/LigandDisplayForward?ligandId=2406 Concentration range: 1×10−^4^M [‐80mV] [http://www.ncbi.nlm.nih.gov/pubmed/14744918?dopt=AbstractPlus] – Mouse
Sub/family‐selective channel blockers	http://www.guidetopharmacology.org/GRAC/LigandDisplayForward?ligandId=4149 (Antagonist)	http://www.guidetopharmacology.org/GRAC/LigandDisplayForward?ligandId=4149 (Antagonist)	–	–
Functional Characteristics	L‐type calcium current: High voltage‐activated, slow voltage dependent inactivation	L‐type calcium current: High voltage‐activated, slow voltage‐dependent inactivation, rapid calcium‐dependent inactivation	L‐type calcium current: moderate‐voltage, slow voltage‐dependent inactivation, more rapid calcium‐dependent inactivation	L‐type calcium current: Moderate voltage‐activated, slow voltage‐dependent inactivation
Comments	–	–	Ca_v_1.3 activates more negative potentials than Ca_v_1.2 and is about 5–10 fold less sensitive to dihydropyridine antagonists.	Ca_v_1.4 is less sensitive to dihydropyridine antagonists than other Cav1 channels

**Table:** 

Nomenclature	http://www.guidetopharmacology.org/GRAC/ObjectDisplayForward?objectId=532	http://www.guidetopharmacology.org/GRAC/ObjectDisplayForward?objectId=533	http://www.guidetopharmacology.org/GRAC/ObjectDisplayForward?objectId=534
HGNC, UniProt	https://www.genenames.org/data/gene‐symbol‐report/#!/hgnc_id/HGNC:1388, http://www.uniprot.org/uniprot/O00555	https://www.genenames.org/data/gene‐symbol‐report/#!/hgnc_id/HGNC:1389, http://www.uniprot.org/uniprot/Q00975	https://www.genenames.org/data/gene‐symbol‐report/#!/hgnc_id/HGNC:1392, http://www.uniprot.org/uniprot/Q15878
Gating inhibitors	–	http://www.guidetopharmacology.org/GRAC/LigandDisplayForward?ligandId=7765 (pIC_50_ 7) [‐80mV] [http://www.ncbi.nlm.nih.gov/pubmed/19815411?dopt=AbstractPlus] – Rat	–
Selective gating inhibitors	http://www.guidetopharmacology.org/GRAC/LigandDisplayForward?ligandId=2529 (P current component: Kd =2nM, Q component Kd= >100nM) (pIC_50_ 7–8.7) [‐100mV – ‐90mV] [http://www.ncbi.nlm.nih.gov/pubmed/10321243?dopt=AbstractPlus, http://www.ncbi.nlm.nih.gov/pubmed/1311418?dopt=AbstractPlus] – Rat, http://www.guidetopharmacology.org/GRAC/LigandDisplayForward?ligandId=2530 (p*K*_d_ 8.5) [‐80mV] [http://www.ncbi.nlm.nih.gov/pubmed/8232218?dopt=AbstractPlus] – Rat	–	http://www.guidetopharmacology.org/GRAC/LigandDisplayForward?ligandId=4315 (Antagonist) (pIC_50_ 7.5–8) [physiological voltage] [http://www.ncbi.nlm.nih.gov/pubmed/9799496?dopt=AbstractPlus]
Channel blockers	–	–	http://www.guidetopharmacology.org/GRAC/LigandDisplayForward?ligandId=2476 (Antagonist) (pIC_50_ 4.6) [‐90mV] [http://www.ncbi.nlm.nih.gov/pubmed/8071363?dopt=AbstractPlus]
Sub/family‐selective channel blockers	http://www.guidetopharmacology.org/GRAC/LigandDisplayForward?ligandId=2537 (pIC_50_ 8.2–9.2) Concentration range: 2×10−^6^M‐5×10−^6^M [physiological voltage] [http://www.ncbi.nlm.nih.gov/pubmed/10938268?dopt=AbstractPlus] – Rat	http://www.guidetopharmacology.org/GRAC/LigandDisplayForward?ligandId=2535 (Antagonist) (pIC_50_ 10.4) [‐80mV] [http://www.ncbi.nlm.nih.gov/pubmed/10938268?dopt=AbstractPlus] – Rat, http://www.guidetopharmacology.org/GRAC/LigandDisplayForward?ligandId=2537 (Antagonist) (pIC_50_ 6.1–8.5) [‐80mV] [http://www.ncbi.nlm.nih.gov/pubmed/1352986?dopt=AbstractPlus, http://www.ncbi.nlm.nih.gov/pubmed/10938268?dopt=AbstractPlus, http://www.ncbi.nlm.nih.gov/pubmed/8786437?dopt=AbstractPlus] – Rat	–
Functional Characteristics	P/Q‐type calcium current: Moderate voltage‐activated, moderate voltage‐dependent inactivation	N‐type calcium current: High voltage‐activated, moderate voltage‐dependent inactivation	R‐type calcium current: Moderate voltage‐activated, fast voltage‐dependent inactivation

**Table:** 

Nomenclature	http://www.guidetopharmacology.org/GRAC/ObjectDisplayForward?objectId=535	http://www.guidetopharmacology.org/GRAC/ObjectDisplayForward?objectId=536	http://www.guidetopharmacology.org/GRAC/ObjectDisplayForward?objectId=537
HGNC, UniProt	https://www.genenames.org/data/gene‐symbol‐report/#!/hgnc_id/HGNC:1394, http://www.uniprot.org/uniprot/O43497	https://www.genenames.org/data/gene‐symbol‐report/#!/hgnc_id/HGNC:1395, http://www.uniprot.org/uniprot/O95180	https://www.genenames.org/data/gene‐symbol‐report/#!/hgnc_id/HGNC:1396, http://www.uniprot.org/uniprot/Q9P0X4
Gating inhibitors	http://www.guidetopharmacology.org/GRAC/LigandDisplayForward?ligandId=2521 (Antagonist) (pIC_50_ 7.3–7.8) [‐90mV] [http://www.ncbi.nlm.nih.gov/pubmed/10196582?dopt=AbstractPlus, http://www.ncbi.nlm.nih.gov/pubmed/11896142?dopt=AbstractPlus] – Rat	http://www.guidetopharmacology.org/GRAC/LigandDisplayForward?ligandId=2521 (Antagonist) (pIC_50_ 7.3–7.6) [‐90mV] [http://www.ncbi.nlm.nih.gov/pubmed/10196582?dopt=AbstractPlus, http://www.ncbi.nlm.nih.gov/pubmed/11896142?dopt=AbstractPlus] – Rat	–
Channel blockers	http://www.guidetopharmacology.org/GRAC/LigandDisplayForward?ligandId=7718 (Pore blocker) (pIC_50_ 7.3) [‐80mV] [http://www.ncbi.nlm.nih.gov/pubmed/22344687?dopt=AbstractPlus], http://www.guidetopharmacology.org/GRAC/LigandDisplayForward?ligandId=7724 (Pore blocker) (pIC_50_ 7) [‐75mV] [http://www.ncbi.nlm.nih.gov/pubmed/23257507?dopt=AbstractPlus], http://www.guidetopharmacology.org/GRAC/LigandDisplayForward?ligandId=2522 (Antagonist) (pIC_50_ 6–6.6) [‐110mV – ‐100mV] [http://www.ncbi.nlm.nih.gov/pubmed/10991994?dopt=AbstractPlus], http://www.guidetopharmacology.org/GRAC/LigandDisplayForward?ligandId=2476 (Antagonist) (pIC_50_ 3.6–3.8) [*voltage dependent* ‐90mV] [http://www.ncbi.nlm.nih.gov/pubmed/10585925?dopt=AbstractPlus] – Rat	http://www.guidetopharmacology.org/GRAC/LigandDisplayForward?ligandId=7724 (Pore blocker) (pIC_50_ 8) [‐75mV] [http://www.ncbi.nlm.nih.gov/pubmed/23257507?dopt=AbstractPlus], http://www.guidetopharmacology.org/GRAC/LigandDisplayForward?ligandId=2522 (Antagonist) (pIC_50_ 5.9–7.2) [‐110mV – ‐80mV] [http://www.ncbi.nlm.nih.gov/pubmed/10991994?dopt=AbstractPlus], http://www.guidetopharmacology.org/GRAC/LigandDisplayForward?ligandId=7718 (Pore blocker) (pIC_50_ 6.8) [‐75mV] [http://www.ncbi.nlm.nih.gov/pubmed/22344687?dopt=AbstractPlus], http://www.guidetopharmacology.org/GRAC/LigandDisplayForward?ligandId=2476 (Antagonist) (pIC_50_ 4.9–5.2) [*voltage dependent* ‐90mV] [http://www.ncbi.nlm.nih.gov/pubmed/10585925?dopt=AbstractPlus]	http://www.guidetopharmacology.org/GRAC/LigandDisplayForward?ligandId=7724 (Pore blocker) (pIC_50_ 7.5) [‐75mV] [http://www.ncbi.nlm.nih.gov/pubmed/23257507?dopt=AbstractPlus], http://www.guidetopharmacology.org/GRAC/LigandDisplayForward?ligandId=2522 (Antagonist) (pIC_50_ 5.8) [‐110mV] [http://www.ncbi.nlm.nih.gov/pubmed/10991994?dopt=AbstractPlus], http://www.guidetopharmacology.org/GRAC/LigandDisplayForward?ligandId=2476 (Antagonist) (pIC_50_ 3.7–4.1) [*voltage dependent* ‐90mV] [http://www.ncbi.nlm.nih.gov/pubmed/10585925?dopt=AbstractPlus] – Rat
Functional Characteristics	T‐type calcium current: Low voltage‐activated, fast voltage‐dependent inactivation	T‐type calcium current: Low voltage‐activated, fast voltage‐dependent inactivation	T‐type calcium current: Low voltage‐activated, moderate voltage‐dependent inactivation

### Comments

In many cell types, P and Q current components cannot be adequately separated and many researchers in the field have adopted the terminology ‘P/Q‐type’ current when referring to either component. Both of these physiologically defined current types are conducted by alternative forms of Cav2.1. Ziconotide (a synthetic peptide equivalent to http://www.guidetopharmacology.org/GRAC/LigandDisplayForward?ligandId=2536) has been approved for the treatment of chronic pain [http://www.ncbi.nlm.nih.gov/pubmed/18518786?dopt=AbstractPlus].

### Further reading on Voltage‐gated calcium channels

Catterall WA *et al.* (2005) International Union of Pharmacology. XLVIII. Nomenclature and structure‐function relationships of voltage‐gated calcium channels. *Pharmacol. Rev.* **57**: 411–25 [https://www.ncbi.nlm.nih.gov/pubmed/16382099?dopt=AbstractPlus]

Catterall WA *et al.* (2015) Structural basis for pharmacology of voltage‐gated sodium and calcium channels. *Mol. Pharmacol.* **88**: 141–50 [https://www.ncbi.nlm.nih.gov/pubmed/25848093?dopt=AbstractPlus]

Catterall WA *et al.* (2015) Deciphering voltage‐gated Na(+) and Ca(2+) channels by studying prokaryotic ancestors. *Trends Biochem. Sci.* **40**: 526–34 [https://www.ncbi.nlm.nih.gov/pubmed/26254514?dopt=AbstractPlus]

Dolphin AC. (2018) Voltage‐gated calcium channels: their discovery, function and importance as drug targets. *Brain Neurosci Adv* **2**: [https://www.ncbi.nlm.nih.gov/pubmed/30320224?dopt=AbstractPlus]

Flucher BE *et al.* (2017) How and why are calcium currents curtailed in the skeletal muscle voltagegated calcium channels? *J. Physiol. (Lond.)* **595**: 1451–1463 [https://www.ncbi.nlm.nih.gov/pubmed/27896815?dopt=AbstractPlus]

Huang J *et al.* (2017) Regulation of voltage gated calcium channels by GPCRs and post‐translational modification. *Curr Opin Pharmacol* **32**: 1–8 [https://www.ncbi.nlm.nih.gov/pubmed/27768908?dopt=AbstractPlus]

Nanou E *et al.* (2018) Calcium Channels, Synaptic Plasticity, and Neuropsychiatric Disease. *Neuron* **98**: 466–481 [https://www.ncbi.nlm.nih.gov/pubmed/29723500?dopt=AbstractPlus]

Ortner NJ *et al.* (2016) L‐type calcium channels as drug targets in CNS disorders. *Channels (Austin)* **10**: 7–13 [https://www.ncbi.nlm.nih.gov/pubmed/26039257?dopt=AbstractPlus]

Rougier JS *et al.* (2016) Cardiac voltage‐gated calcium channel macromolecular complexes. *Biochim. Biophys. Acta* **1863**: 1806–12 [https://www.ncbi.nlm.nih.gov/pubmed/26707467?dopt=AbstractPlus]

Zamponi GW. (2016) Targeting voltage‐gated calcium channels in neurological and psychiatric diseases. *Nat Rev Drug Discov* **15**: 19–34 [https://www.ncbi.nlm.nih.gov/pubmed/26542451?dopt=AbstractPlus]

Zamponi GW *et al.* (2015) The Physiology, Pathology, and Pharmacology of Voltage‐Gated Calcium Channels and Their Future Therapeutic Potential. *Pharmacol. Rev.* **67**: 821–70 [https://www.ncbi.nlm.nih.gov/pubmed/26362469?dopt=AbstractPlus]

## http://www.guidetopharmacology.org/GRAC/FamilyDisplayForward?familyId=124

### Overview

The voltage‐gated proton channel (provisionally denoted Hv1) is a putative 4TM proton‐selective channel gated by membrane depolarization and which is sensitive to the transmembrane pH gradient [http://www.ncbi.nlm.nih.gov/pubmed/20961760?dopt=AbstractPlus, http://www.ncbi.nlm.nih.gov/pubmed/18463791?dopt=AbstractPlus, http://www.ncbi.nlm.nih.gov/pubmed/18801839?dopt=AbstractPlus, http://www.ncbi.nlm.nih.gov/pubmed/16554753?dopt=AbstractPlus, http://www.ncbi.nlm.nih.gov/pubmed/16556803?dopt=AbstractPlus]. The structure of Hv1 is homologous to the voltage sensing domain (VSD) of the superfamily of voltage‐gated ion channels (*i.e*. segments S1 to S4) and contains no discernable pore region [http://www.ncbi.nlm.nih.gov/pubmed/16554753?dopt=AbstractPlus, http://www.ncbi.nlm.nih.gov/pubmed/16556803?dopt=AbstractPlus]. Proton flux through Hv1 is instead most likely mediated by a water wire completed in a crevice of the protein whenthe voltage‐sensing S4 helix moves in response to a change in transmembrane potential [http://www.ncbi.nlm.nih.gov/pubmed/20543828?dopt=AbstractPlus, http://www.ncbi.nlm.nih.gov/pubmed/21843503?dopt=AbstractPlus]. Hv1 expresses largely as a dimer mediated by intracellular C‐terminal coiled‐coil interactions [http://www.ncbi.nlm.nih.gov/pubmed/20147290?dopt=AbstractPlus] but individual promoters nonetheless support gated H^+^ flux via separate conduction pathways [http://www.ncbi.nlm.nih.gov/pubmed/18583477?dopt=AbstractPlus, http://www.ncbi.nlm.nih.gov/pubmed/18509058?dopt=AbstractPlus, http://www.ncbi.nlm.nih.gov/pubmed/21124855?dopt=AbstractPlus, http://www.ncbi.nlm.nih.gov/pubmed/18498736?dopt=AbstractPlus]. Within dimeric structures, the two protomers do not function independently, but display co‐operative interactions during gating resulting in increased voltage sensitivity, but slower activation, of the dimeric,*versus* monomeric, complexes [http://www.ncbi.nlm.nih.gov/pubmed/20023639?dopt=AbstractPlus, http://www.ncbi.nlm.nih.gov/pubmed/20023640?dopt=AbstractPlus].

**Table:** 

Nomenclature	http://www.guidetopharmacology.org/GRAC/ObjectDisplayForward?objectId=746
HGNC, UniProt	https://www.genenames.org/data/gene‐symbol‐report/#!/hgnc_id/HGNC:28240, http://www.uniprot.org/uniprot/Q96D96
Channel blockers	http://www.guidetopharmacology.org/GRAC/LigandDisplayForward?ligandId=566 (pIC_50_ ∼5.7–6.3), http://www.guidetopharmacology.org/GRAC/LigandDisplayForward?ligandId=2440 (pIC_50_ ∼5)
Functional Characteristics	Activated by membrane depolarization mediating macroscopic currents with time‐, voltage‐ and pH‐dependence; outwardly rectifying; voltage dependent kinetics with relatively slow current activation sensitive to extracellular pH and temperature, relatively fast deactivation; voltage threshold for current activation determined by pH gradient (ΔpH = pH_o_ ‐pH_i_) across the membrane

### Comments

The voltage threshold (V_thr_) for activation of Hv1 is not fixed but is set by the pH gradient across the membrane such that V_thr_ is positive to the Nernst potential for H^+^, which ensures that only outwardly directed flux of H^+^ occurs under physiological conditions [http://www.ncbi.nlm.nih.gov/pubmed/20961760?dopt=AbstractPlus, http://www.ncbi.nlm.nih.gov/pubmed/18463791?dopt=AbstractPlus, http://www.ncbi.nlm.nih.gov/pubmed/18801839?dopt=AbstractPlus]. Phosphorylation of Hv1 within the N‐terminal domain by PKC enhances the gating of the channel [http://www.ncbi.nlm.nih.gov/pubmed/20037153?dopt=AbstractPlus]. Tabulated IC_50_ values for Zn^2^+ and Cd^2+^ are for heterologously expressed human and mouse Hv1 [http://www.ncbi.nlm.nih.gov/pubmed/16554753?dopt=AbstractPlus, http://www.ncbi.nlm.nih.gov/pubmed/16556803?dopt=AbstractPlus]. Zn^2+^ is not a conventional pore blocker, but is coordinated by two, or more, external protonation sites involving http://www.guidetopharmacology.org/GRAC/LigandDisplayForward?ligandId=1204 residues [http://www.ncbi.nlm.nih.gov/pubmed/16554753?dopt=AbstractPlus]. Zn^2+^ binding may occur at the dimer interface between pairs of http://www.guidetopharmacology.org/GRAC/LigandDisplayForward?ligandId=1204 residues from both monomers where it may interfere with channel opening [http://www.ncbi.nlm.nih.gov/pubmed/20231140?dopt=AbstractPlus]. Mouse knockout studies demonstrate that Hv1 participates in charge compensation in granulocytes during the respiratory burst of NADPH oxidasedependent reactive oxygen species production that assists in the clearance of bacterial pathogens [http://www.ncbi.nlm.nih.gov/pubmed/19372380?dopt=AbstractPlus]. Additional physiological functions of Hv1 are reviewed by [http://www.ncbi.nlm.nih.gov/pubmed/20961760?dopt=AbstractPlus].

### Further reading on Voltage‐gated proton channel

Castillo K *et al.* (2015) Voltage‐gated proton (H(v)1) channels, a singular voltage sensing domain. *FEBS Lett.* **589**: 3471–8 [https://www.ncbi.nlm.nih.gov/pubmed/26296320?dopt=AbstractPlus]

DeCoursey TE. (2018) Gating currents indicate complex gating of voltage‐gated proton channels. *Proc. Natl. Acad. Sci. U.S.A.* **115**: 9057–9059 [https://www.ncbi.nlm.nih.gov/pubmed/30135099?dopt=AbstractPlus]

DeCoursey TE. (2018) Voltage and pH sensing by the voltage‐gated proton channel, HV1. *J R Soc Interface* **15**: [https://www.ncbi.nlm.nih.gov/pubmed/29643227?dopt=AbstractPlus]

Fernández A *et al.* (2016) Pharmacological Modulation of Proton Channel Hv1 in Cancer Therapy: Future Perspectives. *Mol. Pharmacol.* **90**: 385–402 [https://www.ncbi.nlm.nih.gov/pubmed/27260771?dopt=AbstractPlus]

Okamura Y *et al.* (2015) Gating mechanisms of voltage‐gated proton channels. *Annu. Rev. Biochem.* **84**: 685–709 [https://www.ncbi.nlm.nih.gov/pubmed/26034892?dopt=AbstractPlus]

## http://www.guidetopharmacology.org/GRAC/FamilyDisplayForward?familyId=82

### Overview

Sodium channels are voltage‐gated sodium‐selective ion channels present in the membrane of most excitable cells. Sodium channels comprise of one pore‐forming α subunit, which may be associated with either one or two β subunits [http://www.ncbi.nlm.nih.gov/pubmed/11486343?dopt=AbstractPlus]. α‐Subunits consist of four homologous domains (I‐IV), each containing six transmembrane segments (S1‐S6) and a pore‐forming loop. The positively charged fourth transmembrane segment (S4) acts as a voltage sensor and is involved in channel gating. The crystal structure of the bacterial NavAb channel has revealed a number of novel structural features compared to earlier potassium channel structures including a short selectivity filter with ion selectivity determined by interactions with glutamate side chains [http://www.ncbi.nlm.nih.gov/pubmed/21743477?dopt=AbstractPlus]. Interestingly, the pore region is penetrated by fatty acyl chains that extend into the central cavity which may allow the entry of small, hydrophobic pore‐blocking drugs [http://www.ncbi.nlm.nih.gov/pubmed/21743477?dopt=AbstractPlus]. Auxiliary β1, β2, β3 and β4 subunits consist of a large extracellular N‐terminal domain, a single transmembrane segment and a shorter cytoplasmic domain.

**The nomenclature for sodium channels was proposed by Goldin *et al.*, (2000)** [http://www.ncbi.nlm.nih.gov/pubmed/11144347?dopt=AbstractPlus] **and approved by the NC‐IUPHAR Subcommittee on sodium channels (Catterall *et al.*, 2005,** [http://www.ncbi.nlm.nih.gov/pubmed/16382098?dopt=AbstractPlus]) .

**Table:** 

Nomenclature	http://www.guidetopharmacology.org/GRAC/ObjectDisplayForward?objectId=578	http://www.guidetopharmacology.org/GRAC/ObjectDisplayForward?objectId=579	http://www.guidetopharmacology.org/GRAC/ObjectDisplayForward?objectId=580	http://www.guidetopharmacology.org/GRAC/ObjectDisplayForward?objectId=581
HGNC, UniProt	https://www.genenames.org/data/gene‐symbol‐report/#!/hgnc_id/HGNC:10585, http://www.uniprot.org/uniprot/P35498	https://www.genenames.org/data/gene‐symbol‐report/#!/hgnc_id/HGNC:10588, http://www.uniprot.org/uniprot/Q99250	https://www.genenames.org/data/gene‐symbol‐report/#!/hgnc_id/HGNC:10590, http://www.uniprot.org/uniprot/Q9NY46	https://www.genenames.org/data/gene‐symbol‐report/#!/hgnc_id/HGNC:10591, http://www.uniprot.org/uniprot/P35499
Sub/family‐selective activators	http://www.guidetopharmacology.org/GRAC/LigandDisplayForward?ligandId=2619, http://www.guidetopharmacology.org/GRAC/LigandDisplayForward?ligandId=2626	http://www.guidetopharmacology.org/GRAC/LigandDisplayForward?ligandId=2619 (Agonist) (p*K*_d_ 9.1) [physiological voltage] [http://www.ncbi.nlm.nih.gov/pubmed/9811906?dopt=AbstractPlus] – Rat, http://www.guidetopharmacology.org/GRAC/LigandDisplayForward?ligandId=2626 (Partial agonist) (p*K*_d_ 5.2) [physiological voltage] [http://www.ncbi.nlm.nih.gov/pubmed/6114956?dopt=AbstractPlus] – Rat	http://www.guidetopharmacology.org/GRAC/LigandDisplayForward?ligandId=2619, http://www.guidetopharmacology.org/GRAC/LigandDisplayForward?ligandId=2626	http://www.guidetopharmacology.org/GRAC/LigandDisplayForward?ligandId=2619 (Full agonist) Concentration range: 5×10−^6^M [‐100mV] [http://www.ncbi.nlm.nih.gov/pubmed/9482942?dopt=AbstractPlus] – Rat, http://www.guidetopharmacology.org/GRAC/LigandDisplayForward?ligandId=2626 (Partial agonist) Concentration range: 2×10−^4^M [‐100mV] [http://www.ncbi.nlm.nih.gov/pubmed/9482942?dopt=AbstractPlus] – Rat
Channel blockers	http://www.guidetopharmacology.org/GRAC/LigandDisplayForward?ligandId=2616 (Pore blocker) (p*K*_d_ 8) [‐100mV] [http://www.ncbi.nlm.nih.gov/pubmed/9437003?dopt=AbstractPlus] – Rat	–	–	–
Sub/family‐selective channel blockers	http://www.guidetopharmacology.org/GRAC/LigandDisplayForward?ligandId=9553 [http://www.ncbi.nlm.nih.gov/pubmed/27281198?dopt=AbstractPlus] – Rat, http://www.guidetopharmacology.org/GRAC/LigandDisplayForward?ligandId=2625 (Pore blocker)	http://www.guidetopharmacology.org/GRAC/LigandDisplayForward?ligandId=2625 (Pore blocker) (pIC_50_ 8.8) [‐120mV] [http://www.ncbi.nlm.nih.gov/pubmed/15815630?dopt=AbstractPlus] – Rat, http://www.guidetopharmacology.org/GRAC/LigandDisplayForward?ligandId=2616 (Pore blocker) (pIC_50_ 8) [‐120mV] [http://www.ncbi.nlm.nih.gov/pubmed/15815630?dopt=AbstractPlus] – Rat, http://www.guidetopharmacology.org/GRAC/LigandDisplayForward?ligandId=7472 (Antagonist) (pIC_50_ 4.5) [‐80mV] [http://www.ncbi.nlm.nih.gov/pubmed/24065921?dopt=AbstractPlus] – Rat	http://www.guidetopharmacology.org/GRAC/LigandDisplayForward?ligandId=2616 (Pore blocker) (pIC_50_ 8.4) [http://www.ncbi.nlm.nih.gov/pubmed/11122339?dopt=AbstractPlus], http://www.guidetopharmacology.org/GRAC/LigandDisplayForward?ligandId=2625 (Pore blocker)	http://www.guidetopharmacology.org/GRAC/LigandDisplayForward?ligandId=2625 (Pore blocker) (pIC_50_ 8.4) [‐100mV] [http://www.ncbi.nlm.nih.gov/pubmed/11159437?dopt=AbstractPlus] – Rat, http://www.guidetopharmacology.org/GRAC/LigandDisplayForward?ligandId=2616 (Pore blocker) (pIC_50_ 7.6) [‐120mV] [http://www.ncbi.nlm.nih.gov/pubmed/8058462?dopt=AbstractPlus], http://www.guidetopharmacology.org/GRAC/LigandDisplayForward?ligandId=2630 (Pore blocker) (pIC_50_ 5.9) [‐100mV] [http://www.ncbi.nlm.nih.gov/pubmed/8058462?dopt=AbstractPlus]
Functional Characteristics	Activation V0.5 = ‐20 mV. Fast inactivation (*τ* = 0.7 ms for peak sodium current).	Activation V0.5 = ‐24 mV. Fast inactivation (*τ* = 0.8 ms for peak sodium current).	Activation V0.5 = ‐24 mV. Fast inactivation (0.8 ms)	Activation V0.5 = ‐30 mV. Fast inactivation (0.6 ms)

**Table:** 

Nomenclature	http://www.guidetopharmacology.org/GRAC/ObjectDisplayForward?objectId=582	http://www.guidetopharmacology.org/GRAC/ObjectDisplayForward?objectId=583	http://www.guidetopharmacology.org/GRAC/ObjectDisplayForward?objectId=584	http://www.guidetopharmacology.org/GRAC/ObjectDisplayForward?objectId=585	http://www.guidetopharmacology.org/GRAC/ObjectDisplayForward?objectId=586
HGNC, UniProt	https://www.genenames.org/data/gene‐symbol‐report/#!/hgnc_id/HGNC:10593, http://www.uniprot.org/uniprot/Q14524	https://www.genenames.org/data/gene‐symbol‐report/#!/hgnc_id/HGNC:10596, http://www.uniprot.org/uniprot/Q9UQD0	https://www.genenames.org/data/gene‐symbol‐report/#!/hgnc_id/HGNC:10597, http://www.uniprot.org/uniprot/Q15858	https://www.genenames.org/data/gene‐symbol‐report/#!/hgnc_id/HGNC:10582, http://www.uniprot.org/uniprot/Q9Y5Y9	https://www.genenames.org/data/gene‐symbol‐report/#!/hgnc_id/HGNC:10583, http://www.uniprot.org/uniprot/Q9UI33
Sub/family‐selective activators	http://www.guidetopharmacology.org/GRAC/LigandDisplayForward?ligandId=2619 (Full agonist) (p*K*_d_ 7.6) [physiological voltage] [http://www.ncbi.nlm.nih.gov/pubmed/2431264?dopt=AbstractPlus] – Rat, http://www.guidetopharmacology.org/GRAC/LigandDisplayForward?ligandId=2626 (Partial agonist) (pEC_50_ 6.3) [‐30mV] [http://www.ncbi.nlm.nih.gov/pubmed/2154667?dopt=AbstractPlus] – Rat	http://www.guidetopharmacology.org/GRAC/LigandDisplayForward?ligandId=2619, http://www.guidetopharmacology.org/GRAC/LigandDisplayForward?ligandId=2626	http://www.guidetopharmacology.org/GRAC/LigandDisplayForward?ligandId=2619, http://www.guidetopharmacology.org/GRAC/LigandDisplayForward?ligandId=2626	–	–
Sub/family‐selective channel blockers	http://www.guidetopharmacology.org/GRAC/LigandDisplayForward?ligandId=2616 (Pore blocker) (p*K*_d_ 5.8) [‐80mV] [http://www.ncbi.nlm.nih.gov/pubmed/2175715?dopt=AbstractPlus, http://www.ncbi.nlm.nih.gov/pubmed/8967455?dopt=AbstractPlus] – Rat	http://www.guidetopharmacology.org/GRAC/LigandDisplayForward?ligandId=2616 (Pore blocker) (pIC_50_ 9) [‐130mV] [http://www.ncbi.nlm.nih.gov/pubmed/9603190?dopt=AbstractPlus] – Rat, http://www.guidetopharmacology.org/GRAC/LigandDisplayForward?ligandId=2625 (Pore blocker)	http://www.guidetopharmacology.org/GRAC/LigandDisplayForward?ligandId=2616 (Pore blocker) (pIC_50_ 7.6) [‐100mV] [http://www.ncbi.nlm.nih.gov/pubmed/7720699?dopt=AbstractPlus], http://www.guidetopharmacology.org/GRAC/LigandDisplayForward?ligandId=2625 (Pore blocker) (pIC_50_ 6.2) [http://www.ncbi.nlm.nih.gov/pubmed/23077250?dopt=AbstractPlus]	http://www.guidetopharmacology.org/GRAC/LigandDisplayForward?ligandId=2616 (Pore blocker) (pIC_50_ 4.2) [‐60mV] [http://www.ncbi.nlm.nih.gov/pubmed/8538791?dopt=AbstractPlus] – Rat	http://www.guidetopharmacology.org/GRAC/LigandDisplayForward?ligandId=2616 (Pore blocker) (pIC_50_ 4.4) [‐120mV] [http://www.ncbi.nlm.nih.gov/pubmed/10594087?dopt=AbstractPlus] – Rat
Selective channel blockers	–	–	–	http://www.guidetopharmacology.org/GRAC/LigandDisplayForward?ligandId=8521 (Pore blocker) (pIC_50_ 6.7) [*voltage dependent*] [http://www.ncbi.nlm.nih.gov/pubmed/25625641?dopt=AbstractPlus]	–
Functional Characteristics	Activation V_0.5_ = ‐26 mV. Fast inactivation (*τ* = 1 ms for peak sodium current).	Activation V_0.5_ = ‐29 mV. Fast inactivation (1 ms)	Activation V_0.5_ = ‐27 mV. Fast inactivation (0.5 ms)	Activation V_0.5_ = ‐16 mV. Inactivation (6 ms)	Activation V_0.5_ = ‐32 mV. Slow inactivation (16 ms)

### Comments

Sodium channels are also blocked by local anaesthetic agents, antiarrythmic drugs and antiepileptic drugs. In general, these drugs are not highly selective among channel subtypes. There are two clear functional fingerprints for distinguishing different subtypes. These are sensitivity to http://www.guidetopharmacology.org/GRAC/LigandDisplayForward?ligandId=2616 (Na_V_1.5, Na_V_1.8 and Na_V_1.9 are much less sensitive to block) and rate of fast inactivation (Na_V_1.8 and particularly Na_V_1.9 inactivate more slowly). All sodium channels also have a slow inactivation process that is engaged during long depolarizations (>100 msec) or repetitive trains of stimuli. All sodium channel subtypes are blocked by intracellular http://www.guidetopharmacology.org/GRAC/LigandDisplayForward?ligandId=2405.

### Further reading on Voltage‐gated sodium channels

Catterall WA *et al.* (2005) International Union of Pharmacology. XLVII. Nomenclature and structure‐function relationships of voltage‐gated sodium channels. *Pharmacol. Rev.* **57**: 397409 [https://www.ncbi.nlm.nih.gov/pubmed/16382098?dopt=AbstractPlus]

Catterall WA *et al.* (2017) The chemical basis for electrical signaling. *Nat. Chem. Biol.* **13**: 455–463 [https://www.ncbi.nlm.nih.gov/pubmed/28406893?dopt=AbstractPlus]

Deuis JR *et al.* (2017) The pharmacology of voltage‐gated sodium channel activators. *Neuropharmacology* **127**: 87–108 [https://www.ncbi.nlm.nih.gov/pubmed/28416444?dopt=AbstractPlus]

Jiang D *et al.* (2018) Structural basis for gating pore current in periodic paralysis. *Nature* **557**: 590–594 [https://www.ncbi.nlm.nih.gov/pubmed/29769724?dopt=AbstractPlus]

Kanellopoulos AH *et al.* (2016) Voltage‐gated sodium channels and pain‐related disorders. *Clin. Sci.* **130**: 2257–2265 [https://www.ncbi.nlm.nih.gov/pubmed/27815510?dopt=AbstractPlus]

Shen H *et al.* (2018) Structural basis for the modulation of voltage‐gated sodium channels by animal toxins. *Science* **362**: [https://www.ncbi.nlm.nih.gov/pubmed/30049784?dopt=AbstractPlus]

Terragni B *et al.* (2018) Post‐translational dysfunctions in channelopathies of the nervous system. *Neuropharmacology* **132**: 31–42 [https://www.ncbi.nlm.nih.gov/pubmed/28571716?dopt=AbstractPlus]

## http://www.guidetopharmacology.org/GRAC/FamilyDisplayForward?familyId=119

A number of ion channels in the human genome do not fit readily into the classification of either ligand‐gated or voltage‐gated ion channels. These are identified below.

## http://www.guidetopharmacology.org/GRAC/FamilyDisplayForward?familyId=119

### Overview

Aquaporins and aquaglyceroporins are membrane channels that allow the permeation of water and certain other small solutes across the cell membrane, or in the case of AQP6, AQP11 and AQP12A, intracellular membranes, such as vesicles and the endoplasmic reticulum membrane [http://www.ncbi.nlm.nih.gov/pubmed/26342685?dopt=AbstractPlus]. Since the isolation and cloning of the first aquaporin (AQP1)[http://www.ncbi.nlm.nih.gov/pubmed/1373524?dopt=AbstractPlus], 12 additional mammalian membersof thefamily havebeenidentified, although little is known about the functional properties of one of these (https://www.genenames.org/data/gene‐symbol‐report/#!/hgnc_id/HGNC:19941; http://www.uniprot.org/uniprot/Q8IXF9) and it is thus not tabulated. The other 12 aquaporins can be broadly divided into three families: orthodox aquaporins (AQP0,‐1,‐2,‐4,‐5,‐6 and ‐8) permeable mainly to water, but for some additional solutes [http://www.ncbi.nlm.nih.gov/pubmed/24090884?dopt=AbstractPlus]; aquaglyceroporins (AQP3,‐7 ‐9 and ‐10), additionally permeableto glycerol and for some isoforms urea [http://www.ncbi.nlm.nih.gov/pubmed/26365508?dopt=AbstractPlus], and superaquaporins (AQP11 and 12) located within cells [http://www.ncbi.nlm.nih.gov/pubmed/24189537?dopt=AbstractPlus]. Some aquaporins also conduct ammonia and/or H_2_O_2_ giving rise to the terms ’ammoniaporins’ (’aquaammoniaporins’) and ’peroxiporins’, respectively. Aquaporins are impermeable to protons and other inorganic and organic cations, with the possible exception of AQP1 [http://www.ncbi.nlm.nih.gov/pubmed/26365508?dopt=AbstractPlus]. One or more members of this family of proteins have been found to be expressed in almost all tissues of the body [reviewed in Yang (2017) [1104]]. AQPs are involved in numerous processes that include systemic water homeostasis, adipocyte metabolism, brain oedema, cell migration and fluid secretion by epithelia and loss of function mutations of some human AQPs, or their disruption by autoantibodies further underscore their importance [reviewed by Verkman *et al.* (2014) [http://www.ncbi.nlm.nih.gov/pubmed/24625825?dopt=AbstractPlus], Kitchen *et al.* (2105) [http://www.ncbi.nlm.nih.gov/pubmed/26365508?dopt=AbstractPlus]].

Functional AQPs exist as homotetramers that are the water conducting units wherein individual AQP subunits (each a protomer) have six transmembrane helices and two half helices that constitute a seventh ’pseudotransmembrane domain’ that surrounds a narrow water conducting channel [http://www.ncbi.nlm.nih.gov/pubmed/26342685?dopt=AbstractPlus]. In addition to the four pores contributed by the protomers, an additional hydrophobic pore exists within the center of the complex [http://www.ncbi.nlm.nih.gov/pubmed/26342685?dopt=AbstractPlus] that may mediate the transport of gases (*e.g.* O_2_, CO_2_, NO) and cations (the central pore is the proposed transport pathway for cations through AQP1) by some AQPs [http://www.ncbi.nlm.nih.gov/pubmed/23485707?dopt=AbstractPlus, http://www.ncbi.nlm.nih.gov/pubmed/24141139?dopt=AbstractPlus]. Although numerous small molecule inhibitors of aquaporins, particularly APQ1, have been reported primarily from *Xenopus* oocyte swelling assays, the activity of most has subsequently been disputed upon retesting using assays of water transport that are less prone to various artifacts [http://www.ncbi.nlm.nih.gov/pubmed/26993802?dopt=AbstractPlus] and they are therefore excluded from the tables [see Tradtrantip *et al.* (2017) [http://www.ncbi.nlm.nih.gov/pubmed/28258578?dopt=AbstractPlus] for a review].

**Table:** 

Nomenclature	http://www.guidetopharmacology.org/GRAC/ObjectDisplayForward?objectId=687	http://www.guidetopharmacology.org/GRAC/ObjectDisplayForward?objectId=688	http://www.guidetopharmacology.org/GRAC/ObjectDisplayForward?objectId=689
HGNC, UniProt	https://www.genenames.org/data/gene‐symbol‐report/#!/hgnc_id/HGNC:7103, http://www.uniprot.org/uniprot/P30301	https://www.genenames.org/data/gene‐symbol‐report/#!/hgnc_id/HGNC:633, http://www.uniprot.org/uniprot/P29972	https://www.genenames.org/data/gene‐symbol‐report/#!/hgnc_id/HGNC:634, http://www.uniprot.org/uniprot/P41181
Endogenous activator	AQP0 is gated by calmodulin [http://www.ncbi.nlm.nih.gov/pubmed/26365508?dopt=AbstractPlus]	cGMP (see comment)	–
Permeability	water (rat single channel permeability 0.25 × 10^−14^cm^3^s^−1^) (Rat) [http://www.ncbi.nlm.nih.gov/pubmed/9195910?dopt=AbstractPlus]	water (rat single channel permeability 6.0 × 10^−14^cm^3^s^−1^), ammonia, H_2_O_2_ [http://www.ncbi.nlm.nih.gov/pubmed/23485707?dopt=AbstractPlus, http://www.ncbi.nlm.nih.gov/pubmed/9195910?dopt=AbstractPlus]	water (rat single channel permeability 3.3 × 10–14cm3s‐1) [http://www.ncbi.nlm.nih.gov/pubmed/9388476?dopt=AbstractPlus]
Inhibitors	http://www.guidetopharmacology.org/GRAC/LigandDisplayForward?ligandId=4215	http://www.guidetopharmacology.org/GRAC/LigandDisplayForward?ligandId=4126, http://www.guidetopharmacology.org/GRAC/LigandDisplayForward?ligandId=4215, http://www.guidetopharmacology.org/GRAC/LigandDisplayForward?ligandId=1773	http://www.guidetopharmacology.org/GRAC/LigandDisplayForward?ligandId=4215
Comments	AQP0 appears permeable to CO_2_ [http://www.ncbi.nlm.nih.gov/pubmed/23485707?dopt=AbstractPlus].	Human, but not mouse, AQP1 appears permeable to CO_2_, probably through the central pore of the tetrameric complex [http://www.ncbi.nlm.nih.gov/pubmed/23485707?dopt=AbstractPlus]. NO also appears permeable [http://www.ncbi.nlm.nih.gov/pubmed/16682607?dopt=AbstractPlus]. Permeability to H_2_0_2_ has been demonstrated for rat, but not human, AQP1 [http://www.ncbi.nlm.nih.gov/pubmed/24060746?dopt=AbstractPlus]. Numerous small molecule inhibitors of AQP1 have been proposed, but re‐evaluation indicates that they have no significant effect upon water permeability at concentrations in excess of their originally reported IC_50_ values [http://www.ncbi.nlm.nih.gov/pubmed/26313002?dopt=AbstractPlus]. A fifth pore located at the central axis of the tetrameric complex has, controversially, been described as a cation conductance activated by cGMP and phosphorylation by protein kinases A and C. Evidence in support and against this proposal is discussed in detail by Kitchen *et al.* (2015) [http://www.ncbi.nlm.nih.gov/pubmed/26365508?dopt=AbstractPlus].	–

**Table:** 

Nomenclature	http://www.guidetopharmacology.org/GRAC/ObjectDisplayForward?objectId=690	http://www.guidetopharmacology.org/GRAC/ObjectDisplayForward?objectId=691	http://www.guidetopharmacology.org/GRAC/ObjectDisplayForward?objectId=692	http://www.guidetopharmacology.org/GRAC/ObjectDisplayForward?objectId=693
HGNC, UniProt	https://www.genenames.org/data/gene‐symbol‐report/#!/hgnc_id/HGNC:636, http://www.uniprot.org/uniprot/Q92482	https://www.genenames.org/data/gene‐symbol‐report/#!/hgnc_id/HGNC:637, http://www.uniprot.org/uniprot/P55087	https://www.genenames.org/data/gene‐symbol‐report/#!/hgnc_id/HGNC:638, http://www.uniprot.org/uniprot/P55064	https://www.genenames.org/data/gene‐symbol‐report/#!/hgnc_id/HGNC:639, http://www.uniprot.org/uniprot/Q13520
Permeability	water (rat single channel permeability 2.1 × 10^−14^cm^3^s^−1^), glycerol, ammonia, H_2_O_2_ [http://www.ncbi.nlm.nih.gov/pubmed/24060746?dopt=AbstractPlus, http://www.ncbi.nlm.nih.gov/pubmed/23485707?dopt=AbstractPlus, http://www.ncbi.nlm.nih.gov/pubmed/9195910?dopt=AbstractPlus]	water (rat single channel permeability 24 × 10–14cm3s‐1 [http://www.ncbi.nlm.nih.gov/pubmed/9195910?dopt=AbstractPlus]	water (rat single channel permeability 5.0 × 10–14cm3s‐1), H_2_O_2_ [http://www.ncbi.nlm.nih.gov/pubmed/24141139?dopt=AbstractPlus]	water (zero, or very low basal, permeability is enhanced by low pH and in mouse and rat by Hg^2+^), glycerol, ammonia, urea, anions [http://www.ncbi.nlm.nih.gov/pubmed/23485707?dopt=AbstractPlus, http://www.ncbi.nlm.nih.gov/pubmed/14985982?dopt=AbstractPlus, http://www.ncbi.nlm.nih.gov/pubmed/26365508?dopt=AbstractPlus, http://www.ncbi.nlm.nih.gov/pubmed/25225485?dopt=AbstractPlus]
Activators	–	–	–	http://www.guidetopharmacology.org/GRAC/LigandDisplayForward?ligandId=4215
Inhibitors	http://www.guidetopharmacology.org/GRAC/LigandDisplayForward?ligandId=10188 (pIC_50_ 6.1) [http://www.ncbi.nlm.nih.gov/pubmed/22624030?dopt=AbstractPlus], http://www.guidetopharmacology.org/GRAC/LigandDisplayForward?ligandId=10189 (pIC_50_ 4.8) [http://www.ncbi.nlm.nih.gov/pubmed/22624030?dopt=AbstractPlus], http://www.guidetopharmacology.org/GRAC/LigandDisplayForward?ligandId=4215	–	http://www.guidetopharmacology.org/GRAC/LigandDisplayForward?ligandId=4215	–
Comments	AQP3 is also inhibited by acid pH: permeability to urea is controversial [http://www.ncbi.nlm.nih.gov/pubmed/26365508?dopt=AbstractPlus].	AQP4 is inhibited by PKC activation (although this is probably due to phosphorylation‐dependent protein localisation rather than inhibition of the channel *per se*), but not by HgCl_2_. An isoform of AQP4 (AQP4M23 *vs*. AQPM1) may conduct CO_2_ [http://www.ncbi.nlm.nih.gov/pubmed/23485707?dopt=AbstractPlus]. AQP4 is predicted to be permeable to NO [http://www.ncbi.nlm.nih.gov/pubmed/19842162?dopt=AbstractPlus].	AQP5 may conduct CO_2_ [http://www.ncbi.nlm.nih.gov/pubmed/23485707?dopt=AbstractPlus].	AQP6 is an intracellular channel that localises to acid secreting intercalated cells of the renal collecting ducts. Notably, AQP6 is activated by Hg^2+^ and by low pH and is unusually permeable to anions (with the permeability sequence NO_3_^‐^>I^‐^ *>>*Br^‐^>Cl^‐^ *>>*Fl^‐^) as well as water, both through the monomeric pore [http://www.ncbi.nlm.nih.gov/pubmed/26365508?dopt=AbstractPlus, http://www.ncbi.nlm.nih.gov/pubmed/25225485?dopt=AbstractPlus]. AQP6 may also conduct CO_2_ [http://www.ncbi.nlm.nih.gov/pubmed/23485707?dopt=AbstractPlus].

**Table:** 

Nomenclature	http://www.guidetopharmacology.org/GRAC/ObjectDisplayForward?objectId=694	http://www.guidetopharmacology.org/GRAC/ObjectDisplayForward?objectId=695	http://www.guidetopharmacology.org/GRAC/ObjectDisplayForward?objectId=696	http://www.guidetopharmacology.org/GRAC/ObjectDisplayForward?objectId=697
HGNC, UniProt	https://www.genenames.org/data/gene‐symbol‐report/#!/hgnc_id/HGNC:640, http://www.uniprot.org/uniprot/O14520	https://www.genenames.org/data/gene‐symbol‐report/#!/hgnc_id/HGNC:642, http://www.uniprot.org/uniprot/O94778	https://www.genenames.org/data/gene‐symbol‐report/#!/hgnc_id/HGNC:643, http://www.uniprot.org/uniprot/O43315	https://www.genenames.org/data/gene‐symbol‐report/#!/hgnc_id/HGNC:16029, http://www.uniprot.org/uniprot/Q96PS8
Permeability	water (high), glycerol, ammonia, urea [http://www.ncbi.nlm.nih.gov/pubmed/23485707?dopt=AbstractPlus, http://www.ncbi.nlm.nih.gov/pubmed/9252401?dopt=AbstractPlus]	water (mouse single channel permeability 8.2 × 10^−14^cm^3^s^−1^), ammonia, H_2_0_2_ [http://www.ncbi.nlm.nih.gov/pubmed/24060746?dopt=AbstractPlus, http://www.ncbi.nlm.nih.gov/pubmed/23485707?dopt=AbstractPlus, http://www.ncbi.nlm.nih.gov/pubmed/26365508?dopt=AbstractPlus, http://www.ncbi.nlm.nih.gov/pubmed/9388476?dopt=AbstractPlus]	water (low), glycerol, ammonia, urea, H202, monocarboxylates [http://www.ncbi.nlm.nih.gov/pubmed/23485707?dopt=AbstractPlus, http://www.ncbi.nlm.nih.gov/pubmed/15988592?dopt=AbstractPlus, http://www.ncbi.nlm.nih.gov/pubmed/25225485?dopt=AbstractPlus, http://www.ncbi.nlm.nih.gov/pubmed/26837049?dopt=AbstractPlus]	water (low), glycerol, urea [http://www.ncbi.nlm.nih.gov/pubmed/12084581?dopt=AbstractPlus]
Inhibitors	http://www.guidetopharmacology.org/GRAC/LigandDisplayForward?ligandId=10188 (Effective at 15 *μ*M), http://www.guidetopharmacology.org/GRAC/LigandDisplayForward?ligandId=4215	http://www.guidetopharmacology.org/GRAC/LigandDisplayForward?ligandId=4215	http://www.guidetopharmacology.org/GRAC/LigandDisplayForward?ligandId=4215, http://www.guidetopharmacology.org/GRAC/LigandDisplayForward?ligandId=4285	http://www.guidetopharmacology.org/GRAC/LigandDisplayForward?ligandId=4215
Comments	AQP7 also transports silicon [http://www.ncbi.nlm.nih.gov/pubmed/26313002?dopt=AbstractPlus].	Permeability to urea is controversial, but might be explained by differences between mouse and human caused by a pore‐lining amino acid residue that differs between species [http://www.ncbi.nlm.nih.gov/pubmed/26365508?dopt=AbstractPlus].	AQP9 may conduct CO2 [http://www.ncbi.nlm.nih.gov/pubmed/23485707?dopt=AbstractPlus] and also transport silicon [http://www.ncbi.nlm.nih.gov/pubmed/26313002?dopt=AbstractPlus].	It is not known if AQP10 is permeable to ammonia. Permeability to silicon has been described [http://www.ncbi.nlm.nih.gov/pubmed/26313002?dopt=AbstractPlus].

### Further reading on Aquaporins

Agre P. (2006) The aquaporin water channels. *Proc Am Thorac Soc* **3**: 5–13 [https://www.ncbi.nlm.nih.gov/pubmed/16493146?dopt=AbstractPlus]

Agre P *et al.* (2002) Aquaporin water channels–from atomic structure to clinical medicine. *J. Physiol. (Lond.)* **542**: 3–16 [https://www.ncbi.nlm.nih.gov/pubmed/12096044?dopt=AbstractPlus]

Beitz E *et al.* (2015) Challenges and achievements in the therapeutic modulation of aquaporin functionality. *Pharmacol. Ther.* **155**: 22–35 [https://www.ncbi.nlm.nih.gov/pubmed/26277280?dopt=AbstractPlus]

Carbrey JM *et al.* (2009) Discovery of the aquaporins and development of the field. *Handb Exp Pharmacol* 3–28 [https://www.ncbi.nlm.nih.gov/pubmed/19096770?dopt=AbstractPlus]

Geng X *et al.* (2017) Transport Characteristics of Aquaporins. *Adv. Exp. Med. Biol.* **969**: 51–62 [https://www.ncbi.nlm.nih.gov/pubmed/28258565?dopt=AbstractPlus]

Ishibashi K *et al.* (2009) Aquaporin water channels in mammals. *Clin. Exp. Nephrol.* **13**: 107–17 [https://www.ncbi.nlm.nih.gov/pubmed/19085041?dopt=AbstractPlus]

Kitchen P *et al.* (2015) Beyond water homeostasis: Diverse functional roles of mammalian aquaporins. *Biochim. Biophys. Acta* **1850**: 2410–21 [https://www.ncbi.nlm.nih.gov/pubmed/26365508?dopt=AbstractPlus]

Tesse A *et al.* (2018) Aquaporins as Targets of Dietary Bioactive Phytocompounds. *Front Mol Biosci* **5**: 30 [https://www.ncbi.nlm.nih.gov/pubmed/29721498?dopt=AbstractPlus]

Tradtrantip L *et al.* (2017) Aquaporin‐Targeted Therapeutics: State‐of‐the‐Field. *Adv. Exp. Med. Biol.* **969**: 239–250 [https://www.ncbi.nlm.nih.gov/pubmed/28258578?dopt=AbstractPlus]

Yang B.(2017) Aquaporins *InAdvancesin ExperimentalMedicineandBiology* Edited by Yang B:Springer: 1–276 [ISBN: 9789402410570]

## http://www.guidetopharmacology.org/GRAC/FamilyDisplayForward?familyId=120

### Overview

Chloride channels are a functionally and structurally diverse group of anion selective channels involved in processes including the regulation of the excitability of neurones, skeletal, cardiac and smooth muscle, cell volume regulation, transepithelial salt transport, the acidification of internal and extracellular compartments, the cell cycle and apoptosis (reviewed in [http://www.ncbi.nlm.nih.gov/pubmed/19827947?dopt=AbstractPlus]). Excluding the transmittergated GABA_A_ and glycine receptors (see separate tables), well characterised chloride channels can be classified as certain members of the voltage‐sensitive ClC subfamily, calcium‐activated channels, high (maxi) conductance channels, the cystic fibrosis transmembrane conductance regulator (CFTR) and volume regulated channels [http://www.ncbi.nlm.nih.gov/pubmed/19153558?dopt=AbstractPlus]. No official recommendation exists regarding the classification of chloride channels. Functional chloride channels that have been cloned from, or characterised within, mammalian tissues are listed with the exception of several classes of intracellular channels (*e.g.* CLIC) that are reviewed by in [http://www.ncbi.nlm.nih.gov/pubmed/20100480?dopt=AbstractPlus].

## http://www.guidetopharmacology.org/GRAC/FamilyDisplayForward?familyId=128

### Overview

The mammalian ClC family (reviewed in [http://www.ncbi.nlm.nih.gov/pubmed/20188062?dopt=AbstractPlus, http://www.ncbi.nlm.nih.gov/pubmed/15709979?dopt=AbstractPlus, http://www.ncbi.nlm.nih.gov/pubmed/19827947?dopt=AbstractPlus, http://www.ncbi.nlm.nih.gov/pubmed/17452037?dopt=AbstractPlus, http://www.ncbi.nlm.nih.gov/pubmed/18307107?dopt=AbstractPlus]) contains 9 members that fall, on the basis of sequence homology, into three groups; ClC‐1, ClC‐2, hClC‐Ka (rClC‐K1) and hClC‐Kb (rClC‐K2); ClC‐3 to ClC‐5, and ClC‐6 and ‐7. ClC‐1 and ClC‐2 are plasma membrane chloride channels. ClC‐Ka and ClC‐Kb are also plasma membrane channels (largely expressed in the kidney and inner ear) when associated with barttin (https://www.genenames.org/data/gene‐symbol‐report/#!/hgnc_id/HGNC:16512, http://www.uniprot.org/uniprot/Q8WZ55), a 320 amino acid 2TM protein [http://www.ncbi.nlm.nih.gov/pubmed/11734858?dopt=AbstractPlus]. The localisation of the remaining members of the ClC family is likely to be predominantly intracellular *in vivo*, although they may traffic to the plasma membrane in overexpression systems. Numerous recent reports indicate that ClC‐4, ClC‐5, ClC‐6 and ClC‐7 (and by inference ClC‐3) function as Cl^‐^/H^+^ antiporters (secondary active transport), rather than classical Cl^−^ channels [http://www.ncbi.nlm.nih.gov/pubmed/18449189?dopt=AbstractPlus, http://www.ncbi.nlm.nih.gov/pubmed/21527911?dopt=AbstractPlus, http://www.ncbi.nlm.nih.gov/pubmed/20466723?dopt=AbstractPlus, http://www.ncbi.nlm.nih.gov/pubmed/16034421?dopt=AbstractPlus, http://www.ncbi.nlm.nih.gov/pubmed/16034422?dopt=AbstractPlus]; reviewed in [http://www.ncbi.nlm.nih.gov/pubmed/20188062?dopt=AbstractPlus, http://www.ncbi.nlm.nih.gov/pubmed/16179405?dopt=AbstractPlus]). It has recently been reported that the activity of ClC‐5 as a Cl‐/H^+^ exchanger is important for renal endocytosis [http://www.ncbi.nlm.nih.gov/pubmed/20430975?dopt=AbstractPlus]. Alternative splicing increases the structural diversity within the ClC family. The crystal structure of two bacterial ClC proteins has been described [http://www.ncbi.nlm.nih.gov/pubmed/11796999?dopt=AbstractPlus] and a eukaryotic ClC transporter (CmCLC) has recently been described at 3.5 Å resolution [http://www.ncbi.nlm.nih.gov/pubmed/20929736?dopt=AbstractPlus]. Each ClC subunit, with a complex topology of 18 intramembrane segments, contributes a single pore to a dimeric ‘double‐barrelled’ ClC channel that contains two independently gatedpores, confirming the predictions of previous functional and structural investigations (reviewed in [http://www.ncbi.nlm.nih.gov/pubmed/15709979?dopt=AbstractPlus, http://www.ncbi.nlm.nih.gov/pubmed/17452037?dopt=AbstractPlus, http://www.ncbi.nlm.nih.gov/pubmed/18307107?dopt=AbstractPlus, http://www.ncbi.nlm.nih.gov/pubmed/16179405?dopt=AbstractPlus]). As found for ClC‐4, ClC‐5, ClC‐6 and ClC‐7, the prokaryotic ClC homologue (ClC‐ec1) and CmCLC function as H^+^/Cl antiporters, rather than as ion channels [http://www.ncbi.nlm.nih.gov/pubmed/14985752?dopt=AbstractPlus, http://www.ncbi.nlm.nih.gov/pubmed/20929736?dopt=AbstractPlus]. The generation of monomers from dimeric ClC‐ec1 has firmly established that each ClC subunit is afunctional unit fortransport and thatcross‐subunit interaction is not required for Cl^‐^/H^+^ exchange in ClC transporters [http://www.ncbi.nlm.nih.gov/pubmed/21048711?dopt=AbstractPlus].

**Table:** 

Nomenclature	http://www.guidetopharmacology.org/GRAC/ObjectDisplayForward?objectId=698	http://www.guidetopharmacology.org/GRAC/ObjectDisplayForward?objectId=699
HGNC, UniProt	https://www.genenames.org/data/gene‐symbol‐report/#!/hgnc_id/HGNC:2019, http://www.uniprot.org/uniprot/P35523	https://www.genenames.org/data/gene‐symbol‐report/#!/hgnc_id/HGNC:2020, http://www.uniprot.org/uniprot/P51788
Endogenous activators	–	http://www.guidetopharmacology.org/GRAC/LigandDisplayForward?ligandId=2391
Activators	–	http://www.guidetopharmacology.org/GRAC/LigandDisplayForward?ligandId=4242, http://www.guidetopharmacology.org/GRAC/LigandDisplayForward?ligandId=4279
Channel blockers	http://www.guidetopharmacology.org/GRAC/LigandDisplayForward?ligandId=4113, http://www.guidetopharmacology.org/GRAC/LigandDisplayForward?ligandId=4097, http://www.guidetopharmacology.org/GRAC/LigandDisplayForward?ligandId=4098, http://www.guidetopharmacology.org/GRAC/LigandDisplayForward?ligandId=2440, http://www.guidetopharmacology.org/GRAC/LigandDisplayForward?ligandId=566, http://www.guidetopharmacology.org/GRAC/LigandDisplayForward?ligandId=2662, http://www.guidetopharmacology.org/GRAC/LigandDisplayForward?ligandId=2439	http://www.guidetopharmacology.org/GRAC/LigandDisplayForward?ligandId=4199 (p*K*_d_ 10.8) [*voltage dependent* ‐100mV], http://www.guidetopharmacology.org/GRAC/LigandDisplayForward?ligandId=2440, http://www.guidetopharmacology.org/GRAC/LigandDisplayForward?ligandId=4270, http://www.guidetopharmacology.org/GRAC/LigandDisplayForward?ligandId=566, http://www.guidetopharmacology.org/GRAC/LigandDisplayForward?ligandId=4182
Functional Characteristics	γ = 1–1.5 pS; voltage‐activated (depolarization) (by fast gating of single protopores and a slower common gate allowing both pores to open simultaneously); inwardly rectifying; incomplete deactivation upon repolarization, ATP binding to cytoplasmic cystathionine β‐synthetase related (CBS) domains inhibits ClC‐1 (by closure of the common gate), depending on its redox status	γ = 2–3 pS; voltage‐activated by membrane hyperpolarization by fast protopore and slow cooperative gating; channels only open negative to E_Cl_ resulting in steady‐state inward rectification; voltage dependence modulated by permeant anions; activated by cell swelling, PKA, and weak extracellular acidosis; potentiated by SGK1; inhibited by phosphorylation by p34(cdc2)/cyclin B; cell surface expression and activity increased by association with Hsp90
Comments	CIC‐1 is constitutively active	CIC‐2 is also activated by amidation

**Table:** 

Nomenclature	http://www.guidetopharmacology.org/GRAC/ObjectDisplayForward?objectId=700	http://www.guidetopharmacology.org/GRAC/ObjectDisplayForward?objectId=701	http://www.guidetopharmacology.org/GRAC/ObjectDisplayForward?objectId=702	http://www.guidetopharmacology.org/GRAC/ObjectDisplayForward?objectId=703
HGNC, UniProt	https://www.genenames.org/data/gene‐symbol‐report/#!/hgnc_id/HGNC:2026, http://www.uniprot.org/uniprot/P51800	https://www.genenames.org/data/gene‐symbol‐report/#!/hgnc_id/HGNC:2027, http://www.uniprot.org/uniprot/P51801	https://www.genenames.org/data/gene‐symbol‐report/#!/hgnc_id/HGNC:2021, http://www.uniprot.org/uniprot/P51790	https://www.genenames.org/data/gene‐symbol‐report/#!/hgnc_id/HGNC:2022, http://www.uniprot.org/uniprot/P51793
Activators	http://www.guidetopharmacology.org/GRAC/LigandDisplayForward?ligandId=2439 (pEC_50_ 3–5)	http://www.guidetopharmacology.org/GRAC/LigandDisplayForward?ligandId=2439 (pEC_50_ 3–5)	–	–
Channel blockers	http://www.guidetopharmacology.org/GRAC/LigandDisplayForward?ligandId=4104, http://www.guidetopharmacology.org/GRAC/LigandDisplayForward?ligandId=4177, http://www.guidetopharmacology.org/GRAC/LigandDisplayForward?ligandId=2439	http://www.guidetopharmacology.org/GRAC/LigandDisplayForward?ligandId=4104, http://www.guidetopharmacology.org/GRAC/LigandDisplayForward?ligandId=4177	http://www.guidetopharmacology.org/GRAC/LigandDisplayForward?ligandId=4285 (pIC_50_ 4.5)	http://www.guidetopharmacology.org/GRAC/LigandDisplayForward?ligandId=566 (pIC_50_ 4.3) [http://www.ncbi.nlm.nih.gov/pubmed/18658230?dopt=AbstractPlus], http://www.guidetopharmacology.org/GRAC/LigandDisplayForward?ligandId=2440 (pIC_50_ 4.2) [http://www.ncbi.nlm.nih.gov/pubmed/18658230?dopt=AbstractPlus]
Functional Characteristics	γ = 26 pS; linear current‐voltage relationship except at very negative potentials; no time dependence; inhibited by extracellular protons (p*K* = 7.1); potentiated by extracellular Ca^2+^	Bidirectional rectification; no time dependence; inhibited by extracellular protons; potentiated by extracellular Ca^2+^	Cl‐/H^+^ antiporter [http://www.ncbi.nlm.nih.gov/pubmed/17977943?dopt=AbstractPlus]; pronounced outward rectification; slow activation, fast deactivation; activity enhanced by CaM kinase II; inhibited by intracellular Ins(3,4,5,6)P4 and extracellular acidosis	Cl‐/H^+^ antiporter (2Cl‐:1H^+^) [http://www.ncbi.nlm.nih.gov/pubmed/19364886?dopt=AbstractPlus, http://www.ncbi.nlm.nih.gov/pubmed/16034421?dopt=AbstractPlus, http://www.ncbi.nlm.nih.gov/pubmed/16034422?dopt=AbstractPlus]; extreme outward rectification; voltage‐dependent gating with midpoint of activation at +73 mV [http://www.ncbi.nlm.nih.gov/pubmed/21354396?dopt=AbstractPlus]; rapid activation and deactivation; inhibited by extracellular acidosis; non‐hydrolytic nucleotide binding required for full activity
Comments	CIC‐Ka is constitutively active (when co‐expressed with barttin), and can be blocked by benzofuran derivatives	CIC‐Kb is constitutively active (when co‐expressed with barttin), and can be blocked by benzofuran derivatives	insensitive to the channel blockers http://www.guidetopharmacology.org/GRAC/LigandDisplayForward?ligandId=4177, http://www.guidetopharmacology.org/GRAC/LigandDisplayForward?ligandId=4270 and http://www.guidetopharmacology.org/GRAC/LigandDisplayForward?ligandId=1016 (10 *μ*M)	–

**Table:** 

Nomenclature	http://www.guidetopharmacology.org/GRAC/ObjectDisplayForward?objectId=704	http://www.guidetopharmacology.org/GRAC/ObjectDisplayForward?objectId=705	http://www.guidetopharmacology.org/GRAC/ObjectDisplayForward?objectId=706
HGNC, UniProt	https://www.genenames.org/data/gene‐symbol‐report/#!/hgnc_id/HGNC:2023, http://www.uniprot.org/uniprot/P51795	https://www.genenames.org/data/gene‐symbol‐report/#!/hgnc_id/HGNC:2024, http://www.uniprot.org/uniprot/P51797	https://www.genenames.org/data/gene‐symbol‐report/#!/hgnc_id/HGNC:2025, http://www.uniprot.org/uniprot/P51798
Channel blockers	–	http://www.guidetopharmacology.org/GRAC/LigandDisplayForward?ligandId=4177 (pIC_50_ 3)	http://www.guidetopharmacology.org/GRAC/LigandDisplayForward?ligandId=4177 (pIC_50_ 4.4) [http://www.ncbi.nlm.nih.gov/pubmed/20830208?dopt=AbstractPlus], http://www.guidetopharmacology.org/GRAC/LigandDisplayForward?ligandId=4275 (pIC_50_ 4.3) [http://www.ncbi.nlm.nih.gov/pubmed/20830208?dopt=AbstractPlus], http://www.guidetopharmacology.org/GRAC/LigandDisplayForward?ligandId=4270 (pIC_50_ 3.8) [http://www.ncbi.nlm.nih.gov/pubmed/20830208?dopt=AbstractPlus]
Functional Characteristics	Cl‐/H^+^ antiporter (2Cl‐:1H^+^) [http://www.ncbi.nlm.nih.gov/pubmed/16034421?dopt=AbstractPlus, http://www.ncbi.nlm.nih.gov/pubmed/16034422?dopt=AbstractPlus, http://www.ncbi.nlm.nih.gov/pubmed/20501796?dopt=AbstractPlus, http://www.ncbi.nlm.nih.gov/pubmed/19131966?dopt=AbstractPlus]; extreme outward rectification; voltage‐dependent gating with midpoint of activation of 116.0 mV; rapid activation and deactivation; potentiated and inhibited by intracellular and extracellular acidosis, respectively; ATP binding to cytoplasmic cystathionine β‐synthetase related (CBS) domains activates ClC‐5	Cl‐/H^+^ antiporter (2Cl‐:1H^+^) [http://www.ncbi.nlm.nih.gov/pubmed/20466723?dopt=AbstractPlus]; outward rectification, rapid activation and deactivation	Cl‐/H^+^ antiporter (2Cl‐:1H^+^) [http://www.ncbi.nlm.nih.gov/pubmed/18449189?dopt=AbstractPlus, http://www.ncbi.nlm.nih.gov/pubmed/21527911?dopt=AbstractPlus, http://www.ncbi.nlm.nih.gov/pubmed/20830208?dopt=AbstractPlus]; strong outward rectification; voltage‐dependent gating with a threshold more positive than + 20 mV; very slow activation and deactivation
Comments	Insensitive to the channel blockers http://www.guidetopharmacology.org/GRAC/LigandDisplayForward?ligandId=4177 (1 mM), http://www.guidetopharmacology.org/GRAC/LigandDisplayForward?ligandId=4182 (1 mM), http://www.guidetopharmacology.org/GRAC/LigandDisplayForward?ligandId=4113 (2 mM), http://www.guidetopharmacology.org/GRAC/LigandDisplayForward?ligandId=4270 (0.5 mM) and http://www.guidetopharmacology.org/GRAC/LigandDisplayForward?ligandId=2439 (1 mM)	–	active when co‐expressed with Ostm1

### Comments

ClC channels display the permeability sequence Cl^−^> Br^−^ > I^−^ (at physiological pH). ClC‐1 has significant opening probability at resting membrane potential, accounting for 75% of the membrane conductance at rest in skeletal muscle, and is important for stabilization of the membrane potential. http://www.guidetopharmacology.org/GRAC/LigandDisplayForward?ligandId=4098, http://www.guidetopharmacology.org/GRAC/LigandDisplayForward?ligandId=4113 and http://www.guidetopharmacology.org/GRAC/LigandDisplayForward?ligandId=2439 act intracellularly and exhibit a strongly voltage‐dependent block with strong inhibition at negative voltages and relief of block at depolarized potentials ([http://www.ncbi.nlm.nih.gov/pubmed/17128287?dopt=AbstractPlus] and reviewed in [http://www.ncbi.nlm.nih.gov/pubmed/12512775?dopt=AbstractPlus]). Inhibition of ClC‐2 by the peptide http://www.guidetopharmacology.org/GRAC/LigandDisplayForward?ligandId=4199, from *Leiurus quinquestriatus herbareus* venom, is likely to occur through inhibition of channel gating, rather than direct open channel blockade [http://www.ncbi.nlm.nih.gov/pubmed/19574231?dopt=AbstractPlus]. Although ClC‐2 can be activated by cell swelling, it does not correspond to the VRAC channel (see below). Alternative potential physiological functions for ClC‐2 are reviewed in [http://www.ncbi.nlm.nih.gov/pubmed/19708126?dopt=AbstractPlus]. Functional expression of human ClC‐Ka and ClC‐Kb requires the presence of barttin [http://www.ncbi.nlm.nih.gov/pubmed/11734858?dopt=AbstractPlus, http://www.ncbi.nlm.nih.gov/pubmed/16849430?dopt=AbstractPlus] reviewed in [http://www.ncbi.nlm.nih.gov/pubmed/21423394?dopt=AbstractPlus]. The properties of ClC‐Ka/barttin and ClC‐Kb/barttin tabulated are those observed in mammalian expression systems: in oocytes the channels display time‐ and voltage‐dependent gating. The rodent homologue (ClC‐K1) of ClC‐Ka demonstrates limited expression as a homomer, but its function is enhanced by barttin which increases both channel opening probablility in the physiological range of potentials [http://www.ncbi.nlm.nih.gov/pubmed/11734858?dopt=AbstractPlus, http://www.ncbi.nlm.nih.gov/pubmed/20538786?dopt=AbstractPlus, http://www.ncbi.nlm.nih.gov/pubmed/16849430?dopt=AbstractPlus] reviewed in [http://www.ncbi.nlm.nih.gov/pubmed/21423394?dopt=AbstractPlus]). ClC‐Ka is approximately 5 to 6‐fold more sensitive to block by http://www.guidetopharmacology.org/GRAC/LigandDisplayForward?ligandId=4104 and http://www.guidetopharmacology.org/GRAC/LigandDisplayForward?ligandId=4177 than ClC‐Kb, while newly synthesized benzofuran derivatives showed the same blocking affinity (*<*10 *μ*M) on both CLC‐K isoforms [http://www.ncbi.nlm.nih.gov/pubmed/18216243?dopt=AbstractPlus]. The biophysical and pharmacological properties of ClC‐3, and the relationship of the protein to the endogenous volume‐regulated anion channel(s) VRAC [http://www.ncbi.nlm.nih.gov/pubmed/19036336?dopt=AbstractPlus, http://www.ncbi.nlm.nih.gov/pubmed/16697056?dopt=AbstractPlus] are controversial and further complicated by the possibility that ClC‐3 may function as both a Cl‐/H^+^ exchanger and an ion channel [http://www.ncbi.nlm.nih.gov/pubmed/19036336?dopt=AbstractPlus, http://www.ncbi.nlm.nih.gov/pubmed/16034421?dopt=AbstractPlus, http://www.ncbi.nlm.nih.gov/pubmed/17046694?dopt=AbstractPlus]. The functional properties tabulated are those most consistent with the close structural relationship between ClC‐3, ClC‐4 and ClC‐5. Activation of heterologously expressed ClC‐3 by cell swelling in response to hypotonic solutions is disputed, as are many other aspects of its regulation. Dependent upon the predominant extracellular anion (*e.g.* SCN^−^ versus Cl‐), CIC‐4 can operate in two transport modes: a slippage mode in which behaves as an ion channel and an exchanger mode in which unitary transport rate is 10‐fold lower [http://www.ncbi.nlm.nih.gov/pubmed/19364886?dopt=AbstractPlus]. Similar findings have been made for ClC‐5 [http://www.ncbi.nlm.nih.gov/pubmed/18063579?dopt=AbstractPlus]. ClC‐7 associates with a β subunit, Ostm1, which increases the stability of the former [http://www.ncbi.nlm.nih.gov/pubmed/16525474?dopt=AbstractPlus] and is essential for its function [http://www.ncbi.nlm.nih.gov/pubmed/21527911?dopt=AbstractPlus].

## http://www.guidetopharmacology.org/GRAC/FamilyDisplayForward?familyId=129

### Overview

CFTR, a 12TM, ABC transporter‐type protein, is a cAMP‐regulated epithelial cell membrane Cl^−^ channel involved in normal fluid transport across various epithelia. Of the 1700 mutations identified in CFTR, the most common is the deletion mutant F508 (a class 2 mutation) which results in impaired trafficking of CFTR and reduces its incorporation into the plasma membrane causing cystic fibrosis (reviewed in [http://www.ncbi.nlm.nih.gov/pubmed/21108631?dopt=AbstractPlus]). Channels carrying the F508 mutation that do traffic to the plasma membrane demonstrate gating defects. Thus, pharmacological restoration of the function of the ΔF508 mutant would require a compound that embodies ’corrector’ (*i.e.* facilitates folding and trafficking to the cell surface) and ’potentiator’ (*i.e.* promotes opening of channels at the cell surface) activities [http://www.ncbi.nlm.nih.gov/pubmed/21108631?dopt=AbstractPlus]. In addition to acting as an anion channel *per se*, CFTR may act as a regulator of several other conductances including inhibition of the epithelial Na channel (ENaC), calcium activated chloride channels (CaCC) and volume regulated anion channel (VRAC), activation of the outwardly rectifying chloride channel (ORCC), and enhancementof the sulphonylurea sensitivity of therenalouter medullary potassium channel (ROMK2), (reviewed in [http://www.ncbi.nlm.nih.gov/pubmed/12558550?dopt=AbstractPlus]). CFTR also regulates TRPV4, which provides the Ca^2+^ signal for regulatory volume decrease in airway epithelia [http://www.ncbi.nlm.nih.gov/pubmed/15489228?dopt=AbstractPlus]. The activities of CFTR and the chloride‐bicarbonate exchangers SLC26A3 (DRA) and SLC26A6 (PAT1) are mutually enhanced by a physical association between the regulatory (R) domain of CFTR and the STAS domain of the SCL26 transporters, an effect facilitated by PKA‐mediated phosphorylation of the R domain of CFTR [http://www.ncbi.nlm.nih.gov/pubmed/15048129?dopt=AbstractPlus].

**Table:** 

Nomenclature	http://www.guidetopharmacology.org/GRAC/ObjectDisplayForward?objectId=707
HGNC, UniProt	https://www.genenames.org/data/gene‐symbol‐report/#!/hgnc_id/HGNC:1884, http://www.uniprot.org/uniprot/P13569
Activators	http://www.guidetopharmacology.org/GRAC/LigandDisplayForward?ligandId=4190 (Potentiation) (p*K*_i_ 8.4) [http://www.ncbi.nlm.nih.gov/pubmed/17452495?dopt=AbstractPlus], http://www.guidetopharmacology.org/GRAC/LigandDisplayForward?ligandId=4152 (Potentiation), http://www.guidetopharmacology.org/GRAC/LigandDisplayForward?ligandId=4271 (Potentiation), http://www.guidetopharmacology.org/GRAC/LigandDisplayForward?ligandId=4335 (Potentiation), http://www.guidetopharmacology.org/GRAC/LigandDisplayForward?ligandId=4337 (Potentiation), http://www.guidetopharmacology.org/GRAC/LigandDisplayForward?ligandId=4338 (Potentiation), http://www.guidetopharmacology.org/GRAC/LigandDisplayForward?ligandId=4136 (Potentiation), http://www.guidetopharmacology.org/GRAC/LigandDisplayForward?ligandId=2486 (Potentiation), http://www.guidetopharmacology.org/GRAC/LigandDisplayForward?ligandId=2826 (Potentiation), http://www.guidetopharmacology.org/GRAC/LigandDisplayForward?ligandId=4342 (Potentiation), http://www.guidetopharmacology.org/GRAC/LigandDisplayForward?ligandId=2523 (Potentiation), http://www.guidetopharmacology.org/GRAC/LigandDisplayForward?ligandId=4284 (Potentiation), http://www.guidetopharmacology.org/GRAC/LigandDisplayForward?ligandId=4313 (Potentiation)
Selective inhibitors	http://www.guidetopharmacology.org/GRAC/LigandDisplayForward?ligandId=7453 (pIC_50_ 5.2) [http://www.ncbi.nlm.nih.gov/pubmed/19808995?dopt=AbstractPlus]
Channel blockers	http://www.guidetopharmacology.org/GRAC/LigandDisplayForward?ligandId=2414 (p*K*_i_ 4.7) [http://www.ncbi.nlm.nih.gov/pubmed/1281220?dopt=AbstractPlus], intracellular http://www.guidetopharmacology.org/GRAC/LigandDisplayForward?ligandId=4153 (intracellular application prolongs mean closed time), http://www.guidetopharmacology.org/GRAC/LigandDisplayForward?ligandId=4198, extracellular http://www.guidetopharmacology.org/GRAC/LigandDisplayForward?ligandId=4202
Functional Characteristics	γ = 6–10 pS; permeability sequence = Br^−^ ≥ Cl^−^ > I^−^ > F^‐^, (P_I_/P_Cl_ = 0.1–0.85); slight outward rectification; phosphorylation necessary for activation by ATP binding at binding nucleotide binding domains (NBD)1 and 2; positively regulated by PKC and PKGII (tissue specific); regulated by several interacting proteins including syntaxin 1A, Munc18 and PDZ domain proteins such as NHERF (EBP50) and CAP70
Comments	http://www.guidetopharmacology.org/GRAC/LigandDisplayForward?ligandId=4337, http://www.guidetopharmacology.org/GRAC/LigandDisplayForward?ligandId=4335, http://www.guidetopharmacology.org/GRAC/LigandDisplayForward?ligandId=4136 and http://www.guidetopharmacology.org/GRAC/LigandDisplayForward?ligandId=2826 are examples of flavones. http://www.guidetopharmacology.org/GRAC/LigandDisplayForward?ligandId=4338 and http://www.guidetopharmacology.org/GRAC/LigandDisplayForward?ligandId=4271 are examples of benzimidazolones. http://www.guidetopharmacology.org/GRAC/LigandDisplayForward?ligandId=4152 is an example of a benzoquinoline. http://www.guidetopharmacology.org/GRAC/LigandDisplayForward?ligandId=4190 and http://www.guidetopharmacology.org/GRAC/LigandDisplayForward?ligandId=2523 are examples of 1,4‐dihydropyridines. http://www.guidetopharmacology.org/GRAC/LigandDisplayForward?ligandId=4284 is an example of a phenylglycine. http://www.guidetopharmacology.org/GRAC/LigandDisplayForward?ligandId=4313 is an example of a sulfonamide. Malonic acid hydrazide conjugates are also CFTR channel blockers (see Verkman and Galietta, 2009 [http://www.ncbi.nlm.nih.gov/pubmed/19153558?dopt=AbstractPlus])

### Comments

In addition to the agents listed in the table, the novel small molecule, ataluren, induces translational read through of nonsense mutations in CFTR (reviewed in [http://www.ncbi.nlm.nih.gov/pubmed/20829696?dopt=AbstractPlus]). Corrector compounds that aid the folding of DF508CFTR to increase the amount of protein expressed and potentially delivered to the cell surface include VX‐532 (which is also a potentiator), http://www.guidetopharmacology.org/GRAC/LigandDisplayForward?ligandId=4340, http://www.guidetopharmacology.org/GRAC/LigandDisplayForward?ligandId=4234, Corr‐3a and Corr‐4a see [http://www.ncbi.nlm.nih.gov/pubmed/19153558?dopt=AbstractPlus] for details and structures of Corr3a and Corr‐4a). Inhibition of CFTR by intracellular application of the peptide http://www.guidetopharmacology.org/GRAC/LigandDisplayForward?ligandId=4198, from *Leiurus quinquestriatus herbareus* venom, occurs preferentially for the closed state of the channel [http://www.ncbi.nlm.nih.gov/pubmed/17951250?dopt=AbstractPlus]. CFTR contains two cytoplasmic nucleotide binding domains (NBDs) that bind http://www.guidetopharmacology.org/GRAC/LigandDisplayForward?ligandId=1713. A single open‐closing cycle is hypothesised to involve, in sequence: binding of http://www.guidetopharmacology.org/GRAC/LigandDisplayForward?ligandId=1713 at the Nterminal NBD1, http://www.guidetopharmacology.org/GRAC/LigandDisplayForward?ligandId=1713 binding to the C‐terminal NBD2 leading to the formation of an intramolecular NBD1‐NBD2 dimer associated with the open state, and subsequent http://www.guidetopharmacology.org/GRAC/LigandDisplayForward?ligandId=1713 hydrolysis at NBD2 facilitating dissociation of the dimer and channel closing, and the initiation of a new gating cycle [http://www.ncbi.nlm.nih.gov/pubmed/17021796?dopt=AbstractPlus, http://www.ncbi.nlm.nih.gov/pubmed/18957373?dopt=AbstractPlus]. Phosphorylation by PKA at sites within a cytoplasmic regulatory (R) domain facilitates the interaction of the two NBD domains. PKC (and PKGII within intestinal epithelial cells via guanylinstimulated http://www.guidetopharmacology.org/GRAC/LigandDisplayForward?ligandId=2347 formation) positively regulate CFTR activity.

## http://www.guidetopharmacology.org/GRAC/FamilyDisplayForward?familyId=130

### Overview

Chloride channels activated by intracellular calcium (CaCC) are widely expressed in excitable and non‐excitable cells where they perform diverse functions [http://www.ncbi.nlm.nih.gov/pubmed/15709976?dopt=AbstractPlus]. The molecular nature of CaCC has been uncertain with both *CLCA*, *TWEETY* and *BEST* genes having been considered as likely candidates [http://www.ncbi.nlm.nih.gov/pubmed/19827947?dopt=AbstractPlus, http://www.ncbi.nlm.nih.gov/pubmed/18391176?dopt=AbstractPlus, http://www.ncbi.nlm.nih.gov/pubmed/15987802?dopt=AbstractPlus]. It is now accepted that CLCA expression products are unlikely to form channels *per se* and probably function as cell adhesion proteins, or are secreted [http://www.ncbi.nlm.nih.gov/pubmed/18954282?dopt=AbstractPlus]. Similarly, *TWEETY* gene products do not recapictulate the properties of endogenous CaCC. The bestrophins encoded by genes *BEST1‐4* have a topology more consistent with ion channels [http://www.ncbi.nlm.nih.gov/pubmed/18391176?dopt=AbstractPlus] and form chloride channels that are activated by physiological concentrations of Ca^2+^, but whether such activation is direct is not known [http://www.ncbi.nlm.nih.gov/pubmed/18391176?dopt=AbstractPlus]. However, currents generated by bestrophin over‐expression do not resemble native CaCC currents. The evidence for and against bestrophin proteins forming CaCC is critically reviewed by Duran *et al.* [http://www.ncbi.nlm.nih.gov/pubmed/19827947?dopt=AbstractPlus]. Recently, a new gene family, TMEM16 (anoctamin) consisting of 10 members (TMEM16A‐K; anoctamin 1–10) has been identified and there is firm evidence that some of these members form chloride channels [http://www.ncbi.nlm.nih.gov/pubmed/21642943?dopt=AbstractPlus, http://www.ncbi.nlm.nih.gov/pubmed/21607626?dopt=AbstractPlus]. TMEM16A (anoctamin 1; Ano 1) produces Ca^2+^‐activated Cl^−^ currents with kinetics similar to native CaCC currents recorded from different cell types [http://www.ncbi.nlm.nih.gov/pubmed/18772398?dopt=AbstractPlus, http://www.ncbi.nlm.nih.gov/pubmed/19363029?dopt=AbstractPlus, http://www.ncbi.nlm.nih.gov/pubmed/18805094?dopt=AbstractPlus, http://www.ncbi.nlm.nih.gov/pubmed/18724360?dopt=AbstractPlus]. Knockdown of TMEM16A greatly reduces currents mediated by calcium‐activated chloride channels in submandibular gland cells [http://www.ncbi.nlm.nih.gov/pubmed/18724360?dopt=AbstractPlus] and smooth muscle cells from pulmonary artery [http://www.ncbi.nlm.nih.gov/pubmed/20421283?dopt=AbstractPlus]. In TMEM16A^(‐/‐)^ mice secretion of Ca^2+^‐dependent Cl^−^ secretion by several epithelia is reduced [http://www.ncbi.nlm.nih.gov/pubmed/19679661?dopt=AbstractPlus, http://www.ncbi.nlm.nih.gov/pubmed/19363029?dopt=AbstractPlus]. Alternative splicing regulates the voltage‐ and Ca^2+^‐ dependence of TMEM16A and such processing may be tissue‐specific manner and thus contribute to functional diversity [http://www.ncbi.nlm.nih.gov/pubmed/19819874?dopt=AbstractPlus]. There are also reports that TMEM16B (anoctamin 2; Ano 2) supports CaCC activity (*e.g*.[http://www.ncbi.nlm.nih.gov/pubmed/19475416?dopt=AbstractPlus]) and in TMEM16B^(‐/‐)^ mice Ca‐activated Cl^−^ currents in the main olfactory epithelium (MOE) and in the vomeronasal organ are virtually absent [http://www.ncbi.nlm.nih.gov/pubmed/21516098?dopt=AbstractPlus].

**Table:** 

Nomenclature	http://www.guidetopharmacology.org/GRAC/ObjectDisplayForward?objectId=708
HGNC, UniProt	https://www.genenames.org/data/gene‐symbol‐report/#!/hgnc_id/HGNC:21625, http://www.uniprot.org/uniprot/Q5XXA6
Endogenous activators	intracellular http://www.guidetopharmacology.org/GRAC/LigandDisplayForward?ligandId=707
Selective inhibitors	http://www.guidetopharmacology.org/GRAC/LigandDisplayForward?ligandId=7453 (pIC_50_ 5.2) [http://www.ncbi.nlm.nih.gov/pubmed/19808995?dopt=AbstractPlus]
Endogenous channel blockers	http://www.guidetopharmacology.org/GRAC/LigandDisplayForward?ligandId=4224
Channel blockers	http://www.guidetopharmacology.org/GRAC/LigandDisplayForward?ligandId=4113, http://www.guidetopharmacology.org/GRAC/LigandDisplayForward?ligandId=4173, http://www.guidetopharmacology.org/GRAC/LigandDisplayForward?ligandId=4177, http://www.guidetopharmacology.org/GRAC/LigandDisplayForward?ligandId=4270, http://www.guidetopharmacology.org/GRAC/LigandDisplayForward?ligandId=4314, http://www.guidetopharmacology.org/GRAC/LigandDisplayForward?ligandId=2447, http://www.guidetopharmacology.org/GRAC/LigandDisplayForward?ligandId=203, http://www.guidetopharmacology.org/GRAC/LigandDisplayForward?ligandId=2522, http://www.guidetopharmacology.org/GRAC/LigandDisplayForward?ligandId=2439, http://www.guidetopharmacology.org/GRAC/LigandDisplayForward?ligandId=4319
Functional Characteristics	γ = 0.5–5 pS; permeability sequence, SCN^−^ > NO_3_^−^> I^−^ > Br^−^ > Cl^−^ > F^−^; relative permeability of SCN^−^:Cl^−^ 8. I‐:Cl^−^ 3, aspartate:Cl^−^ 0.15, outward rectification (decreased by increasing [Ca^2+^]_i_); sensitivity to activation by [Ca^2+^]_i_ decreased at hyperpolarized potentials; slow activation at positive potentials (accelerated by increasing [Ca^2+^]_i_); rapid deactivation at negative potentials, deactivation kinetics modulated by anions binding to an external site; modulated by redox status

### Comments

Blockade of I_Cl(Ca)_ by http://www.guidetopharmacology.org/GRAC/LigandDisplayForward?ligandId=2439, http://www.guidetopharmacology.org/GRAC/LigandDisplayForward?ligandId=4177 and http://www.guidetopharmacology.org/GRAC/LigandDisplayForward?ligandId=4113 is voltage‐dependent whereas block by http://www.guidetopharmacology.org/GRAC/LigandDisplayForward?ligandId=4270 is voltage‐independent [http://www.ncbi.nlm.nih.gov/pubmed/15709976?dopt=AbstractPlus]. Extracellular http://www.guidetopharmacology.org/GRAC/LigandDisplayForward?ligandId=2439; http://www.guidetopharmacology.org/GRAC/LigandDisplayForward?ligandId=4173 and http://www.guidetopharmacology.org/GRAC/LigandDisplayForward?ligandId=4113 (but not http://www.guidetopharmacology.org/GRAC/LigandDisplayForward?ligandId=4177) exert a complex effect upon I_Cl(Ca)_ in vascular smooth muscle, enhancing and inhibiting inwardly and outwardly directed currents in a manner dependent upon [Ca^2+^]_i_ (see [http://www.ncbi.nlm.nih.gov/pubmed/16091780?dopt=AbstractPlus] for summary). Considerable crossover in pharmacology with large conductance Ca^2+^‐activated K^+^ channels also exists (see [http://www.ncbi.nlm.nih.gov/pubmed/17150263?dopt=AbstractPlus] for overview). Two novel compounds, CaCC_inh_‐A01 and CaCC_inh_‐B01 have recently been identified as blockers of calcium‐activated chloride channels in T84 human intestinal epithelial cells [http://www.ncbi.nlm.nih.gov/pubmed/18083779?dopt=AbstractPlus] for structures). Significantly, other novel compounds totally block currents mediated by TMEM116A, but have only a modest effect upon total current mediated by CaCC native to T84 cells or human bronchial epithelial cells, suggesting that TMEM16A is not the predominant CaCC in such cells [http://www.ncbi.nlm.nih.gov/pubmed/21084298?dopt=AbstractPlus]. CaMKII modulates CaCC in a tissue dependent manner (reviewed by [http://www.ncbi.nlm.nih.gov/pubmed/15709976?dopt=AbstractPlus, http://www.ncbi.nlm.nih.gov/pubmed/16091780?dopt=AbstractPlus]). CaMKII inhibitors block activation of I_Cl(Ca)_ in T84 cells but have no effect in parotid acinar cells. In tracheal and arterial smooth muscle cells, but not portal vein myocytes, inhibition of CaMKII reduces inactivation of I_Cl(Ca)_. Intracellular http://www.guidetopharmacology.org/GRAC/LigandDisplayForward?ligandId=4224 may act as an endogenous negative regulator of CaCC channels activated by Ca^2+^, or CaMKII. Smooth muscle CaCC are also regulated positively by Ca^2+^‐dependent phosphatase, calcineurin (see [http://www.ncbi.nlm.nih.gov/pubmed/16091780?dopt=AbstractPlus] for summary).

## http://www.guidetopharmacology.org/GRAC/FamilyDisplayForward?familyId=131

### Overview

Maxi Cl^‐^ channels are high conductance, anion selective, channels initially characterised in skeletal muscle and subsequently found in many cell types including neurones, glia, cardiac muscle, lymphocytes, secreting and absorbing epithelia, macula densa cells of the kidney and human placenta syncytiotrophoblasts [http://www.ncbi.nlm.nih.gov/pubmed/19340557?dopt=AbstractPlus]. The physiological significance of the maxi Cl^‐^ channel is uncertain, but roles in cell volume regulation and apoptosis have been claimed. Evidence suggests a role for maxi Clchannels as a conductive pathway in the swelling‐induced release of http://www.guidetopharmacology.org/GRAC/LigandDisplayForward?ligandId=1713 from mouse mammary C127i cells that may be important for autocrine and paracrine signalling by purines [http://www.ncbi.nlm.nih.gov/pubmed/12154180?dopt=AbstractPlus, http://www.ncbi.nlm.nih.gov/pubmed/11524456?dopt=AbstractPlus]. A similar channel mediates http://www.guidetopharmacology.org/GRAC/LigandDisplayForward?ligandId=1713 release from macula densa cells within the thick ascending of the loop of Henle in response to changes in luminal NaCl concentration [http://www.ncbi.nlm.nih.gov/pubmed/12655045?dopt=AbstractPlus]. A family of human high conductance Cl^‐^ channels (TTYH1‐3) that resemble Maxi Cl^‐^ channels has been cloned [http://www.ncbi.nlm.nih.gov/pubmed/15010458?dopt=AbstractPlus], but alternatively, Maxi Cl^‐^ channels have also been suggested to correspond to the voltage‐dependent anion channel, VDAC, expressed at the plasma membrane [http://www.ncbi.nlm.nih.gov/pubmed/12794078?dopt=AbstractPlus, http://www.ncbi.nlm.nih.gov/pubmed/15477379?dopt=AbstractPlus].

**Table:** 

Nomenclature	http://www.guidetopharmacology.org/GRAC/ObjectDisplayForward?objectId=709
Activators	cytosolic http://www.guidetopharmacology.org/GRAC/LigandDisplayForward?ligandId=4207, extracellular http://www.guidetopharmacology.org/GRAC/LigandDisplayForward?ligandId=83, extracellular http://www.guidetopharmacology.org/GRAC/LigandDisplayForward?ligandId=1016, extracellular http://www.guidetopharmacology.org/GRAC/LigandDisplayForward?ligandId=4325, extracellular http://www.guidetopharmacology.org/GRAC/LigandDisplayForward?ligandId=4330
Endogenous channel blockers	intracellular http://www.guidetopharmacology.org/GRAC/LigandDisplayForward?ligandId=2391
Channel blockers	http://www.guidetopharmacology.org/GRAC/LigandDisplayForward?ligandId=4177 (pIC_50_ 4.4) [http://www.ncbi.nlm.nih.gov/pubmed/20830208?dopt=AbstractPlus], extracellular http://www.guidetopharmacology.org/GRAC/LigandDisplayForward?ligandId=566 (pIC_50_ 4.3) [http://www.ncbi.nlm.nih.gov/pubmed/18658230?dopt=AbstractPlus], http://www.guidetopharmacology.org/GRAC/LigandDisplayForward?ligandId=4270 (pIC_50_ 3.8) [http://www.ncbi.nlm.nih.gov/pubmed/20830208?dopt=AbstractPlus], extracellular http://www.guidetopharmacology.org/GRAC/LigandDisplayForward?ligandId=2426, http://www.guidetopharmacology.org/GRAC/LigandDisplayForward?ligandId=4314, http://www.guidetopharmacology.org/GRAC/LigandDisplayForward?ligandId=4182
Functional Characteristics	γ = 280–430 pS (main state); permeability sequence, I > Br > Cl > F > gluconate (PCIP_Cl_ = 1.5); ATP is a voltage dependent permeant blocker of single channel activity (PATP/P_Cl_ = 0.08‐0.1); channel activity increased by patch‐excision; channel opening probability (at steady‐state) maximal within approximately ± 20 mV of 0 mV, opening probability decreased at more negative and (commonly) positive potentials yielding a bell‐shaped curve; channel conductance and opening probability regulated by annexin 6
Comments	Maxi Cl^‐^ is also activated by G protein‐coupled receptors and cell swelling. http://www.guidetopharmacology.org/GRAC/LigandDisplayForward?ligandId=1016 and http://www.guidetopharmacology.org/GRAC/LigandDisplayForward?ligandId=4325 are examples of triphenylethylene anti‐oestrogens

### Comments

Differing ionic conditions may contribute to variable estimates of γ reported in the literature. Inhibition by http://www.guidetopharmacology.org/GRAC/LigandDisplayForward?ligandId=2391 (and cis‐unsaturated fatty acids) is voltageindependent, occurs at an intracellular site, and involves both channel shut down (K*d* = 4‐5 *μ*M) and a reduction of γ (K*d* = 1314 *μ*M). Blockade of channel activity by http://www.guidetopharmacology.org/GRAC/LigandDisplayForward?ligandId=4314, http://www.guidetopharmacology.org/GRAC/LigandDisplayForward?ligandId=4177, Gd^3+^ and http://www.guidetopharmacology.org/GRAC/LigandDisplayForward?ligandId=2391 is paralleled by decreased swelling‐induced release of http://www.guidetopharmacology.org/GRAC/LigandDisplayForward?ligandId=1713 [http://www.ncbi.nlm.nih.gov/pubmed/12154180?dopt=AbstractPlus, http://www.ncbi.nlm.nih.gov/pubmed/11524456?dopt=AbstractPlus]. Channel activation by anti‐oestrogens in whole cell recordings requires the presence of intracellular nucleotides and is prevented by pre‐treatment with http://www.guidetopharmacology.org/GRAC/LigandDisplayForward?ligandId=1013, http://www.guidetopharmacology.org/GRAC/LigandDisplayForward?ligandId=5485, or intracellular dialysis with GDPβS [http://www.ncbi.nlm.nih.gov/pubmed/11579158?dopt=AbstractPlus]. Activation by http://www.guidetopharmacology.org/GRAC/LigandDisplayForward?ligandId=1016 is suppressed by low concentrations of http://www.guidetopharmacology.org/GRAC/LigandDisplayForward?ligandId=5349, suggesting that a dephosphorylation event by protein phosphatase PP2A occurs in the activation pathway [http://www.ncbi.nlm.nih.gov/pubmed/11579158?dopt=AbstractPlus]. In contrast, http://www.guidetopharmacology.org/GRAC/LigandDisplayForward?ligandId=1013 and http://www.guidetopharmacology.org/GRAC/LigandDisplayForward?ligandId=1016 appear to directly inhibit the maxi Cl^‐^ channel of human placenta reconstituted into giant liposomes and recorded in excised patches [http://www.ncbi.nlm.nih.gov/pubmed/19604577?dopt=AbstractPlus].

## http://www.guidetopharmacology.org/GRAC/FamilyDisplayForward?familyId=132

### Overview

Volume activated chloride channels (also termed VSOAC, volume‐sensitive organic osmolyte/anion channel; VRC, volume regulated channel and VSOR, volume expansion‐sensing outwardly rectifying anion channel) participate in regulatory volumedecrease(RVD) inresponse tocell swelling. VRAC may also be important for several other processes including the regulation of membrane excitability, transcellular Cl^‐^ transport, angiogenesis, cell proliferation, necrosis, apoptosis, glutamate release from astrocytes, http://www.guidetopharmacology.org/GRAC/LigandDisplayForward?ligandId=5012 (https://www.genenames.org/data/gene‐symbol‐report/#!/hgnc_id/HGNC:6081, http://www.uniprot.org/uniprot/P01308) release from pancreatic β cells and resistance to the anti‐cancer drug, http://www.guidetopharmacology.org/GRAC/LigandDisplayForward?ligandId=5343 (reviewed by [http://www.ncbi.nlm.nih.gov/pubmed/21099297?dopt=AbstractPlus, http://www.ncbi.nlm.nih.gov/pubmed/17047222?dopt=AbstractPlus, http://www.ncbi.nlm.nih.gov/pubmed/12558550?dopt=AbstractPlus, http://www.ncbi.nlm.nih.gov/pubmed/19171657?dopt=AbstractPlus]). VRAC may not be a single entity, but may instead represent a number of different channels that are expressed to a variable extent in different tissues and are differentially activated by cell swelling. In addition to ClC‐3 expression products (see above) several former VRAC candidates including *MDR1* (ABCB1 P‐glycoprotein), Icln, Band 3 anion exchanger and phospholemman are also no longer considered likely to fulfil this function (see reviews [http://www.ncbi.nlm.nih.gov/pubmed/12558550?dopt=AbstractPlus, http://www.ncbi.nlm.nih.gov/pubmed/14729152?dopt=AbstractPlus]).

**Table:** 

Nomenclature	http://www.guidetopharmacology.org/GRAC/ObjectDisplayForward?objectId=710
Activators	http://www.guidetopharmacology.org/GRAC/LigandDisplayForward?ligandId=4207
Endogenous channel blockers	intracellular http://www.guidetopharmacology.org/GRAC/LigandDisplayForward?ligandId=708, http://www.guidetopharmacology.org/GRAC/LigandDisplayForward?ligandId=2391
Channel blockers	http://www.guidetopharmacology.org/GRAC/LigandDisplayForward?ligandId=4100, http://www.guidetopharmacology.org/GRAC/LigandDisplayForward?ligandId=4113, http://www.guidetopharmacology.org/GRAC/LigandDisplayForward?ligandId=4174, http://www.guidetopharmacology.org/GRAC/LigandDisplayForward?ligandId=4177, http://www.guidetopharmacology.org/GRAC/LigandDisplayForward?ligandId=4217, http://www.guidetopharmacology.org/GRAC/LigandDisplayForward?ligandId=4270, http://www.guidetopharmacology.org/GRAC/LigandDisplayForward?ligandId=4273, http://www.guidetopharmacology.org/GRAC/LigandDisplayForward?ligandId=4151, http://www.guidetopharmacology.org/GRAC/LigandDisplayForward?ligandId=4159, http://www.guidetopharmacology.org/GRAC/LigandDisplayForward?ligandId=4176, http://www.guidetopharmacology.org/GRAC/LigandDisplayForward?ligandId=4204, http://www.guidetopharmacology.org/GRAC/LigandDisplayForward?ligandId=4252, http://www.guidetopharmacology.org/GRAC/LigandDisplayForward?ligandId=2522, http://www.guidetopharmacology.org/GRAC/LigandDisplayForward?ligandId=4263, http://www.guidetopharmacology.org/GRAC/LigandDisplayForward?ligandId=4265, http://www.guidetopharmacology.org/GRAC/LigandDisplayForward?ligandId=2342, http://www.guidetopharmacology.org/GRAC/LigandDisplayForward?ligandId=2510, http://www.guidetopharmacology.org/GRAC/LigandDisplayForward?ligandId=1016
Functional Characteristics	γ = 10–20 pS (negative potentials), 50–90 pS (positive potentials); permeability sequence SCN > I > NO_3_^‐^ >Br^‐^ > Cl^‐^ > F^‐^ > gluconate; outward rectification due to voltage dependence of γ; inactivates at positive potentials in many, but not all, cell types; time dependent inactivation at positive potentials; intracellular ionic strength modulates sensitivity to cell swelling and rate of channel activation; rate of swelling‐induced activation is modulated by intracellular ATP concentration; ATP dependence is independent of hydrolysis and modulated by rate of cell swelling; inhibited by increased intracellular free Mg^2+^ concentration; swelling induced activation of several intracellular signalling cascades may be permissive of, but not essential to, the activation of VRAC including: the Rho‐Rho kinase‐MLCK; Ras‐Raf‐MEK‐ERK; PIK3‐NOX‐H_2_O_2_ and Src‐PLCγ‐Ca^2+^ pathways; regulation by PKCα required for optimal activity; cholesterol depletion enhances activity; activated by direct stretch of β1‐integrin
Comments	VRAC is also activated by cell swelling and low intracellular ionic strength. VRAC is also blocked by chromones, extracellular nucleotides and nucleoside analogues

### Comments

In addition to conducting monovalent anions, in many cell types the activation of VRAC by a hypotonic stimulus can allow the efflux of organic osmolytes such as amino acids and polyols that may contribute to RVD.

### Comments on Chloride channels: Other chloride channels

In addition to some intracellular chloride channels that are not considered here, plasma membrane channels other than those listed have been functionally described. Many cells and tissues contain outwardly rectifying chloride channels (ORCC) that may correspond to VRAC active under isotonic conditions. A http://www.guidetopharmacology.org/GRAC/LigandDisplayForward?ligandId=2352‐activated Cl^‐^ channel that does not correspond to CFTR has been described in intestinal Paneth cells [http://www.ncbi.nlm.nih.gov/pubmed/9769420?dopt=AbstractPlus]. A Cl channel activated by http://www.guidetopharmacology.org/GRAC/LigandDisplayForward?ligandId=2347 with a dependence on raised intracellular Ca^2+^ has been recorded in various vascular smooth muscle cells types, which has a pharmacology and biophysical characteristics very different from the ‘conventional’ CaCC [http://www.ncbi.nlm.nih.gov/pubmed/14718479?dopt=AbstractPlus, http://www.ncbi.nlm.nih.gov/pubmed/14724180?dopt=AbstractPlus]. It has been proposed that http://www.guidetopharmacology.org/GRAC/LigandDisplayForward?ligandId=5377 (https://www.genenames.org/data/gene‐symbol‐report/#!/hgnc_id/HGNC:17105, http://www.uniprot.org/uniprot/Q8N1M1) is an essential component of the http://www.guidetopharmacology.org/GRAC/LigandDisplayForward?ligandId=2347‐activated channel [http://www.ncbi.nlm.nih.gov/pubmed/18776041?dopt=AbstractPlus]. A proton‐activated, outwardly rectifying anion channel has also been described [http://www.ncbi.nlm.nih.gov/pubmed/15961423?dopt=AbstractPlus].

### Further reading on Chloride channels

Adkins GB *et al.* (2015) Potential role of cardiac chloride channels and transporters as novel therapeutic targets. *Pharmacol. Ther.* **145**: 67–75 [https://www.ncbi.nlm.nih.gov/pubmed/25160469?dopt=AbstractPlus]

Huang F *et al.* (2012) International Union of Basic and Clinical Pharmacology. LXXXV: calciumactivated chloride channels. *Pharmacol. Rev.* **64**: 1–15 [https://www.ncbi.nlm.nih.gov/pubmed/22090471?dopt=AbstractPlus]

KamaleddinMA.(2018)Molecular, biophysical, and pharmacological properties of calcium‐activated chloride channels. *J. Cell. Physiol.* **233**: 787–798 [https://www.ncbi.nlm.nih.gov/pubmed/28121009?dopt=AbstractPlus]

Kunzelmann K. (2015) TMEM16, LRRC8A, bestrophin: chloride channels controlled by Ca(2+) and cell volume. *Trends Biochem. Sci.* **40**: 535–43 [https://www.ncbi.nlm.nih.gov/pubmed/26254230?dopt=AbstractPlus]

Pedersen SF *et al.* (2016) Biophysics and Physiology of the Volume‐Regulated Anion Channel (VRAC)/Volume‐Sensitive Outwardly Rectifying Anion Channel (VSOR). *Pflugers Arch.* **468**: 371–83 [https://www.ncbi.nlm.nih.gov/pubmed/26739710?dopt=AbstractPlus]

Peretti M *et al.* (2015) Chloride channels in cancer: Focus on chloride intracellular channel 1 and 4 (CLIC1 AND CLIC4) proteins in tumor development and as novel therapeutic targets. *Biochim. Biophys. Acta* **1848**: 2523–31 [https://www.ncbi.nlm.nih.gov/pubmed/25546839?dopt=AbstractPlus]

Sabirov RZ *et al.* (2016) The properties, functions, and pathophysiology of maxi‐anion channels. *Pflugers Arch.* **468**: 405–20 [https://www.ncbi.nlm.nih.gov/pubmed/26733413?dopt=AbstractPlus]

Zegarra‐Moran O *et al.* (2017) CFTR pharmacology. *Cell. Mol. Life Sci.* **74**: 117–128 [https://www.ncbi.nlm.nih.gov/pubmed/27704174?dopt=AbstractPlus]

## http://www.guidetopharmacology.org/GRAC/FamilyDisplayForward?familyId=121

### Overview

Gap junctions are essential for many physiological processes including cardiac and smooth muscle contraction, regulation of neuronal excitability and epithelial electrolyte transport [http://www.ncbi.nlm.nih.gov/pubmed/14597722?dopt=AbstractPlus, http://www.ncbi.nlm.nih.gov/pubmed/15217338?dopt=AbstractPlus, http://www.ncbi.nlm.nih.gov/pubmed/12126230?dopt=AbstractPlus]. Gap junction channels allow the passive diffusion of molecules of up to 1,000 Daltons which can include nutrients, metabolites and second messengers (such as IP_3_) as well as cations and anions. 21 connexin genes and 3 pannexin genes which are structurally related to the invertebrate innexin genes) code for gap junction proteins in humans. Each connexin gap junction comprises 2 hemichannels or ’connexons’ which are themselves formed from 6 connexin molecules. The various connexins have been observed to combine into both homomeric and heteromeric combinations, each of which may exhibit different functional properties. Itis also suggested that individual hemichannels formed by a number of different connexins might be functional in at least some cells [http://www.ncbi.nlm.nih.gov/pubmed/17507078?dopt=AbstractPlus]. Connexins have a common topology, with four α‐helical transmembrane domains, two extracellular loops, a cytoplasmic loop, and N‐ and C‐termini located on the cytoplasmic membrane face. In mice, the most abundant connexins in electrical synapses in the brain seem to be Cx36, Cx45 and Cx57 [http://www.ncbi.nlm.nih.gov/pubmed/15738956?dopt=AbstractPlus]. Mutations in connexin genes are associated with the occurrence of a number of pathologies, such as peripheral neuropathies, cardiovascular diseases and hereditary deafness. The pannexin genes Px1 and Px2 are widely expressed in the mammalian brain [http://www.ncbi.nlm.nih.gov/pubmed/16143426?dopt=AbstractPlus]. Like the connexins, at least some of the pannexins can form hemichannels [http://www.ncbi.nlm.nih.gov/pubmed/14597722?dopt=AbstractPlus, http://www.ncbi.nlm.nih.gov/pubmed/17121814?dopt=AbstractPlus].

**Table:** 

Nomenclature	http://www.guidetopharmacology.org/GRAC/ObjectDisplayForward?objectId=714	http://www.guidetopharmacology.org/GRAC/ObjectDisplayForward?objectId=715	http://www.guidetopharmacology.org/GRAC/ObjectDisplayForward?objectId=716	http://www.guidetopharmacology.org/GRAC/ObjectDisplayForward?objectId=717	http://www.guidetopharmacology.org/GRAC/ObjectDisplayForward?objectId=718	http://www.guidetopharmacology.org/GRAC/ObjectDisplayForward?objectId=719	http://www.guidetopharmacology.org/GRAC/ObjectDisplayForward?objectId=720
HGNC, UniProt	https://www.genenames.org/data/gene‐symbol‐report/#!/hgnc_id/HGNC:33251, http://www.uniprot.org/uniprot/A6NN92	https://www.genenames.org/data/gene‐symbol‐report/#!/hgnc_id/HGNC:16690, http://www.uniprot.org/uniprot/Q6PEY0	https://www.genenames.org/data/gene‐symbol‐report/#!/hgnc_id/HGNC:4284, http://www.uniprot.org/uniprot/P29033	https://www.genenames.org/data/gene‐symbol‐report/#!/hgnc_id/HGNC:4288, http://www.uniprot.org/uniprot/O95452	https://www.genenames.org/data/gene‐symbol‐report/#!/hgnc_id/HGNC:17495, http://www.uniprot.org/uniprot/Q8NFK1	https://www.genenames.org/data/gene‐symbol‐report/#!/hgnc_id/HGNC:4286, http://www.uniprot.org/uniprot/Q9NTQ9	https://www.genenames.org/data/gene‐symbol‐report/#!/hgnc_id/HGNC:4285, http://www.uniprot.org/uniprot/O75712
Endogenous inhibitors extracellular http://www.guidetopharmacology.org/GRAC/LigandDisplayForward?ligandId=707 (blocked by raising external Ca^2+^)							
Inhibitors http://www.guidetopharmacology.org/GRAC/LigandDisplayForward?ligandId=4151, http://www.guidetopharmacology.org/GRAC/LigandDisplayForward?ligandId=2447, http://www.guidetopharmacology.org/GRAC/LigandDisplayForward?ligandId=4278

**Table:** 

Nomenclature	http://www.guidetopharmacology.org/GRAC/ObjectDisplayForward?objectId=721	http://www.guidetopharmacology.org/GRAC/ObjectDisplayForward?objectId=722	http://www.guidetopharmacology.org/GRAC/ObjectDisplayForward?objectId=723	http://www.guidetopharmacology.org/GRAC/ObjectDisplayForward?objectId=724	http://www.guidetopharmacology.org/GRAC/ObjectDisplayForward?objectId=725	http://www.guidetopharmacology.org/GRAC/ObjectDisplayForward?objectId=726	http://www.guidetopharmacology.org/GRAC/ObjectDisplayForward?objectId=727
HGNC, UniProt	https://www.genenames.org/data/gene‐symbol‐report/#!/hgnc_id/HGNC:4287, http://www.uniprot.org/uniprot/O95377	https://www.genenames.org/data/gene‐symbol‐report/#!/hgnc_id/HGNC:19147, http://www.uniprot.org/uniprot/Q8N144	https://www.genenames.org/data/gene‐symbol‐report/#!/hgnc_id/HGNC:4283, http://www.uniprot.org/uniprot/P08034	https://www.genenames.org/data/gene‐symbol‐report/#!/hgnc_id/HGNC:19154, http://www.uniprot.org/uniprot/Q9UKL4	https://www.genenames.org/data/gene‐symbol‐report/#!/hgnc_id/HGNC:4278, http://www.uniprot.org/uniprot/P35212	https://www.genenames.org/data/gene‐symbol‐report/#!/hgnc_id/HGNC:4279, http://www.uniprot.org/uniprot/P36382	https://www.genenames.org/data/gene‐symbol‐report/#!/hgnc_id/HGNC:23296, http://www.uniprot.org/uniprot/Q96KN9
Endogenous inhibitors extracellular http://www.guidetopharmacology.org/GRAC/LigandDisplayForward?ligandId=707 (blocked by raising external Ca^2+^)							
Inhibitors http://www.guidetopharmacology.org/GRAC/LigandDisplayForward?ligandId=4151, http://www.guidetopharmacology.org/GRAC/LigandDisplayForward?ligandId=2447, http://www.guidetopharmacology.org/GRAC/LigandDisplayForward?ligandId=4278

**Table:** 

Nomenclature	http://www.guidetopharmacology.org/GRAC/ObjectDisplayForward?objectId=728	http://www.guidetopharmacology.org/GRAC/ObjectDisplayForward?objectId=729	http://www.guidetopharmacology.org/GRAC/ObjectDisplayForward?objectId=730	http://www.guidetopharmacology.org/GRAC/ObjectDisplayForward?objectId=731	http://www.guidetopharmacology.org/GRAC/ObjectDisplayForward?objectId=732	http://www.guidetopharmacology.org/GRAC/ObjectDisplayForward?objectId=733	http://www.guidetopharmacology.org/GRAC/ObjectDisplayForward?objectId=734
HGNC, UniProt	https://www.genenames.org/data/gene‐symbol‐report/#!/hgnc_id/HGNC:4274, http://www.uniprot.org/uniprot/P17302	https://www.genenames.org/data/gene‐symbol‐report/#!/hgnc_id/HGNC:4280, http://www.uniprot.org/uniprot/P36383	https://www.genenames.org/data/gene‐symbol‐report/#!/hgnc_id/HGNC:4277, http://www.uniprot.org/uniprot/Q9Y6H8	https://www.genenames.org/data/gene‐symbol‐report/#!/hgnc_id/HGNC:17494, http://www.uniprot.org/uniprot/Q5T442	https://www.genenames.org/data/gene‐symbol‐report/#!/hgnc_id/HGNC:4281, http://www.uniprot.org/uniprot/P48165	https://www.genenames.org/data/gene‐symbol‐report/#!/hgnc_id/HGNC:19155, http://www.uniprot.org/uniprot/P57773	https://www.genenames.org/data/gene‐symbol‐report/#!/hgnc_id/HGNC:16995, http://www.uniprot.org/uniprot/Q969M2
Endogenous inhibitors extracellular http://www.guidetopharmacology.org/GRAC/LigandDisplayForward?ligandId=707 (blocked by raising external Ca^2+^)							
Inhibitors http://www.guidetopharmacology.org/GRAC/LigandDisplayForward?ligandId=4151, http://www.guidetopharmacology.org/GRAC/LigandDisplayForward?ligandId=2447, http://www.guidetopharmacology.org/GRAC/LigandDisplayForward?ligandId=4278

**Table:** 

Nomenclature	http://www.guidetopharmacology.org/GRAC/ObjectDisplayForward?objectId=735	http://www.guidetopharmacology.org/GRAC/ObjectDisplayForward?objectId=736	http://www.guidetopharmacology.org/GRAC/ObjectDisplayForward?objectId=737
HGNC, UniProt	https://www.genenames.org/data/gene‐symbol‐report/#!/hgnc_id/HGNC:8599, http://www.uniprot.org/uniprot/Q96RD7	https://www.genenames.org/data/gene‐symbol‐report/#!/hgnc_id/HGNC:8600, http://www.uniprot.org/uniprot/Q96RD6	https://www.genenames.org/data/gene‐symbol‐report/#!/hgnc_id/HGNC:20573, http://www.uniprot.org/uniprot/Q96QZ0
Inhibitors	http://www.guidetopharmacology.org/GRAC/LigandDisplayForward?ligandId=4151, http://www.guidetopharmacology.org/GRAC/LigandDisplayForward?ligandId=2447 (little block by flufenamic acid)	http://www.guidetopharmacology.org/GRAC/LigandDisplayForward?ligandId=4151, http://www.guidetopharmacology.org/GRAC/LigandDisplayForward?ligandId=2447 (little block by flufenamic acid)	http://www.guidetopharmacology.org/GRAC/LigandDisplayForward?ligandId=4151, http://www.guidetopharmacology.org/GRAC/LigandDisplayForward?ligandId=2447 (little block by flufenamic acid)
Comments	Unaffected by raising external Ca^2+^	Unaffected by raising external Ca^2+^	Unaffected by raising external Ca^2+^

### Comments

Connexins are most commonly named according to their molecular weights, so, for example, Cx23 is the connexin protein of 23 kDa. This can cause confusion when comparing between species – for example, the mouse connexin Cx57 is orthologous to the human connexin Cx62. No natural toxin or specific inhibitor of junctional channels has been identified yet however two compounds often used experimentally to block connexins are http://www.guidetopharmacology.org/GRAC/LigandDisplayForward?ligandId=4151 and http://www.guidetopharmacology.org/GRAC/LigandDisplayForward?ligandId=2447 [http://www.ncbi.nlm.nih.gov/pubmed/16216217?dopt=AbstractPlus]. At least some pannexin hemichannels are more sensitive to http://www.guidetopharmacology.org/GRAC/LigandDisplayForward?ligandId=4151 than connexins but much less sensitive to http://www.guidetopharmacology.org/GRAC/LigandDisplayForward?ligandId=2447 [http://www.ncbi.nlm.nih.gov/pubmed/15715654?dopt=AbstractPlus]. It has been suggested that 2‐aminoethoxydiphenyl borate (http://www.guidetopharmacology.org/GRAC/LigandDisplayForward?ligandId=2433) may be a more effective blocker of some connexin channel subtypes (Cx26, Cx30, Cx36, Cx40, Cx45, Cx50) compared to others (Cx32, Cx43, Cx46, [http://www.ncbi.nlm.nih.gov/pubmed/16985167?dopt=AbstractPlus]).

### Further reading on Connexins and Pannexins

Decrock E *et al.* (2015) Connexin and pannexin signaling pathways, an architectural blueprint for CNS physiology and pathology? *Cell. Mol. Life Sci.* **72**: 2823–51 [https://www.ncbi.nlm.nih.gov/pubmed/26118660?dopt=AbstractPlus]

Esseltine JL *et al.* (2016) Next‐Generation Connexin and Pannexin Cell Biology. *Trends Cell Biol.* **26**: 944–955 [https://www.ncbi.nlm.nih.gov/pubmed/27339936?dopt=AbstractPlus]

Freund‐Michel V *et al.* (2016) Expression and role of connexin‐based gap junctions in pulmonary inflammatory diseases. *Pharmacol. Ther.* **164**: 105–19 [https://www.ncbi.nlm.nih.gov/pubmed/27126473?dopt=AbstractPlus]

Harris AL. (2018) Electrical coupling and its channels. *J. Gen. Physiol.* **150**: 1606–1639 [https://www.ncbi.nlm.nih.gov/pubmed/30389716?dopt=AbstractPlus]

Sáez JC *et al.* (2015) Regulation of pannexin and connexin channels and their functional role in skeletal muscles. *Cell. Mol. Life Sci.* **72**: 2929–35 [https://www.ncbi.nlm.nih.gov/pubmed/26084874?dopt=AbstractPlus]

Willebrords J *et al.* (2017) Inhibitors of connexin and pannexin channels as potential therapeutics. *Pharmacol. Ther.* **180**: 144–160 [https://www.ncbi.nlm.nih.gov/pubmed/28720428?dopt=AbstractPlus]

## http://www.guidetopharmacology.org/GRAC/FamilyDisplayForward?familyId=967

### Overview

Piezo proteins are the pore‐forming subunits of trimeric mechanosensitive ion channels that open in response to mechanical stimuli such as shear stress and membrane stretch, allowing positively charged ions, including calcium, to flow into the cell. Piezo orthologs have thus far been identified in numerous eukaryotes. Most vertebrates have two channel isoforms, Piezol and Piezo2. Across species, Piezos are very large proteins (2521 and 2752 amino acids for human Piezo1 and human Piezo2, respectively) with numerous (>14) predicted transmembrane (TM) domains per subunit and, strikingly, no homology to other known proteins [http://www.ncbi.nlm.nih.gov/pubmed/27743844?dopt=AbstractPlus]. Piezo channels play a critical role in sensory neuron transduction [http://www.ncbi.nlm.nih.gov/pubmed/30305457?dopt=AbstractPlus, http://www.ncbi.nlm.nih.gov/pubmed/30361375?dopt=AbstractPlus]

**Table:** 

Nomenclature	http://www.guidetopharmacology.org/GRAC/ObjectDisplayForward?objectId=2945	http://www.guidetopharmacology.org/GRAC/ObjectDisplayForward?objectId=2946
HGNC, UniProt	https://www.genenames.org/data/gene‐symbol‐report/#!/hgnc_id/HGNC:28993, http://www.uniprot.org/uniprot/Q92508	https://www.genenames.org/data/gene‐symbol‐report/#!/hgnc_id/HGNC:26270, http://www.uniprot.org/uniprot/Q9H5I5
Selective activators	http://www.guidetopharmacology.org/GRAC/LigandDisplayForward?ligandId=9817 (pEC_50_ 4.6) [http://www.ncbi.nlm.nih.gov/pubmed/26001275?dopt=AbstractPlus], http://www.guidetopharmacology.org/GRAC/LigandDisplayForward?ligandId=10111 (pEC_50_ 3.8) [http://www.ncbi.nlm.nih.gov/pubmed/29610524?dopt=AbstractPlus] – Mouse, http://www.guidetopharmacology.org/GRAC/LigandDisplayForward?ligandId=10249 [http://www.ncbi.nlm.nih.gov/pubmed/29610524?dopt=AbstractPlus] – Mouse	–
Inhibitors	http://www.guidetopharmacology.org/GRAC/LigandDisplayForward?ligandId=10112 (pIC_50_ 5.8) [http://www.ncbi.nlm.nih.gov/pubmed/29498036?dopt=AbstractPlus]	–
Functional Characteristics	Mechano‐activated	Mechano‐activated

### Comments

http://www.guidetopharmacology.org/GRAC/LigandDisplayForward?ligandId=9817 is a Piezo1 channel activator [http://www.ncbi.nlm.nih.gov/pubmed/29498036?dopt=AbstractPlus, http://www.ncbi.nlm.nih.gov/pubmed/27797339?dopt=AbstractPlus].

### Further reading on Piezo channels

Chesler AT *et al.* (2018) Portraits of a pressure sensor. *Elife* **7**: [https://www.ncbi.nlm.nih.gov/pubmed/29376828?dopt=AbstractPlus]

Ehmke H. (2018) The mechanotransduction of blood pressure. *Science* **362**: 398–399 [https://www.ncbi.nlm.nih.gov/pubmed/30361358?dopt=AbstractPlus]

Gottlieb PA*etal*. (2012)Piezo1: propertiesof a cation selective mechanical channel. *Channels(Austin)* **6**: 214–9 [https://www.ncbi.nlm.nih.gov/pubmed/22790400?dopt=AbstractPlus]

Parpaite T *et al.* (2017) Piezo channels. *Curr. Biol.* **27**: R250‐R252 [https://www.ncbi.nlm.nih.gov/pubmed/28376327?dopt=AbstractPlus]

Volkers L *et al.* (2015) Piezo channels: from structure to function. *Pflugers Arch.* **467**: 95–9 [https://www.ncbi.nlm.nih.gov/pubmed/25037583?dopt=AbstractPlus]

Wu J *et al.* (2017) Touch, Tension, and Transduction ‐ The Function and Regulation of Piezo Ion Channels. *Trends Biochem. Sci.* **42**: 57–71 [https://www.ncbi.nlm.nih.gov/pubmed/27743844?dopt=AbstractPlus]

Zeng WZ *etal*. (2018)PIEZOs mediate neuronal sensing of blood pressure and the baroreceptor reflex. *Science* **362**: 464–467 [https://www.ncbi.nlm.nih.gov/pubmed/30361375?dopt=AbstractPlus]

## http://www.guidetopharmacology.org/GRAC/FamilyDisplayForward?familyId=126

### Overview

The sodium leak channel, non selective (**NC‐IUPHAR tentatively recommends the nomenclature Na_Vi_2.1, W.A. Catterall, personal communication**) is structurally a member of the family of voltage‐gated sodium channel family (Na_v_1.1‐Na_v_1.9) [http://www.ncbi.nlm.nih.gov/pubmed/10094463?dopt=AbstractPlus, http://www.ncbi.nlm.nih.gov/pubmed/15467096?dopt=AbstractPlus]. In contrast to the latter, Na_Vi_2.1, is voltage‐insensitive (denoted in the subscript ’vi’ in the tentative nomenclature) and possesses distinctive ion selectivity and pharmacological properties. Na_Vi_2.1, which is insensitive to http://www.guidetopharmacology.org/GRAC/LigandDisplayForward?ligandId=2616 (10 *μ*M), has been proposed to mediate the http://www.guidetopharmacology.org/GRAC/LigandDisplayForward?ligandId=2616‐resistant and voltage‐insensitive Na^+^ leak current (I_L_‐Na) observed in many types of neurone [http://www.ncbi.nlm.nih.gov/pubmed/17448995?dopt=AbstractPlus]. However, whether Na_Vi_2.1 is constitutively active has been challenged [http://www.ncbi.nlm.nih.gov/pubmed/19575010?dopt=AbstractPlus]. Na_Vi_2.1 is widely distributed within the central nervous system and is also expressed in the heart and pancreas specifically, in rodents, within the islets of Langerhans [http://www.ncbi.nlm.nih.gov/pubmed/10094463?dopt=AbstractPlus, http://www.ncbi.nlm.nih.gov/pubmed/17448995?dopt=AbstractPlus]. Recently, Na_Vi_2.1 has been proposed to be a core effector for the action of inhibitory G proteins [http://www.ncbi.nlm.nih.gov/pubmed/30556810?dopt=AbstractPlus].

**Table:** 

Nomenclature	http://www.guidetopharmacology.org/GRAC/ObjectDisplayForward?objectId=750
HGNC, UniProt	https://www.genenames.org/data/gene‐symbol‐report/#!/hgnc_id/HGNC:19082, http://www.uniprot.org/uniprot/Q8IZF0
Activators	Constitutively active (Lu *et al*., 2007), or activated downstream of Src family tyrosine kinases (SFKs) (Lu *et al*.,2009; Swayne *et al*., 2009); positively modulated by decreased extracellular Ca^2+^ concentration (Lu *et al*., 2010) [http://www.ncbi.nlm.nih.gov/pubmed/17448995?dopt=AbstractPlus, http://www.ncbi.nlm.nih.gov/pubmed/19092807?dopt=AbstractPlus, http://www.ncbi.nlm.nih.gov/pubmed/21040849?dopt=AbstractPlus, http://www.ncbi.nlm.nih.gov/pubmed/19575010?dopt=AbstractPlus]
Channel blockers	http://www.guidetopharmacology.org/GRAC/LigandDisplayForward?ligandId=2426 (pIC_50_ 5.6), http://www.guidetopharmacology.org/GRAC/LigandDisplayForward?ligandId=2440 (pIC_50_ 3.8), http://www.guidetopharmacology.org/GRAC/LigandDisplayForward?ligandId=4160 (pIC_50_ 3.6), http://www.guidetopharmacology.org/GRAC/LigandDisplayForward?ligandId=2406 (pIC_50_ 3.4)
Functional Characteristics	γ = 27 pS (by fluctuation analysis), *P*_Na_/*P*_Cs_ = 1.3, *P*_K_/*P*_Cs_ = 1.2, *P*_Ca_/*P*_Cs_ = 0.5, linear current voltage‐relationship, voltage‐independent and non‐inactivating

### Comments

In native and recombinant expression systems Na_Vi_2.1 can be activated by stimulation of NK_1_ (in hippocampal neurones), neurotensin (in ventral tegmental area neurones) and M3 muscarinic acetylcholine receptors (in MIN6 pancreatic β‐cells) and in a manner that is independent of signalling through G proteins [http://www.ncbi.nlm.nih.gov/pubmed/19092807?dopt=AbstractPlus, http://www.ncbi.nlm.nih.gov/pubmed/19575010?dopt=AbstractPlus]. Pharmacological and molecular biological evidence indicates such modulation to occur though a pathway that involves the activation of Src family tyrosine kinases. It is suggested that Na_Vi_2.1 exists as a macromolecular complex with M3 receptors [http://www.ncbi.nlm.nih.gov/pubmed/19575010?dopt=AbstractPlus] and peptide receptors [http://www.ncbi.nlm.nih.gov/pubmed/19092807?dopt=AbstractPlus], in the latter instance in association with the protein UNC‐80, which recruits Src to the channel complex [http://www.ncbi.nlm.nih.gov/pubmed/19092807?dopt=AbstractPlus, http://www.ncbi.nlm.nih.gov/pubmed/19535918?dopt=AbstractPlus]. By contrast, stimulation of Na_Vi_2.1 by decreased extracellular Ca^2+^ concentration is G protein dependent and involves a Ca^2+^‐sensing G protein‐coupled receptor and UNC80 which links Na_Vi_2.1 to the protein UNC79 in the same complex [http://www.ncbi.nlm.nih.gov/pubmed/21040849?dopt=AbstractPlus]. Na_Vi_2.1 null mutant mice have severe disturbances in respiratory rhythm and die within 24 hours of birth [http://www.ncbi.nlm.nih.gov/pubmed/17448995?dopt=AbstractPlus]. Na_Vi_2.1 heterozygous knockout mice display increased serum sodium concentrations in comparison to wildtype littermates and a role for the channel in osmoregulation has been postulated [http://www.ncbi.nlm.nih.gov/pubmed/21177381?dopt=AbstractPlus].

### Further reading on Sodium leak channel, non‐selective

Cochet‐Bissuel M *et al*. (2014) The sodium leak channel, NALCN, in health and disease. *Front Cell Neurosci* **8**: 132 [https://www.ncbi.nlm.nih.gov/pubmed/24904279?dopt=AbstractPlus]

Lu TZ *et al*. (2012) NALCN: a regulator of pacemaker activity. *Mol. Neurobiol*. **45**: 415‐23 [https://www.ncbi.nlm.nih.gov/pubmed/22476981?dopt=AbstractPlus]

Philippart F *et al*. (2018) G_i/o protein‐coupled receptors in dopamine neurons inhibit the sodium leak channel NALCN. *Elife* **7**: [https://www.ncbi.nlm.nih.gov/pubmed/30556810?dopt=AbstractPlus]

Waxman SG *et al*. (2014) Regulating excitability of peripheral afferents: emerging ion channel targets. *Nat. Neurosci*. **17**: 153‐63 [https://www.ncbi.nlm.nih.gov/pubmed/24473263?dopt=AbstractPlus]
